# Reduced SNP Panels for Genetic Identification and Introgression Analysis in the Dark Honey Bee (*Apis mellifera mellifera*)

**DOI:** 10.1371/journal.pone.0124365

**Published:** 2015-04-13

**Authors:** Irene Muñoz, Dora Henriques, J. Spencer Johnston, Julio Chávez-Galarza, Per Kryger, M. Alice Pinto

**Affiliations:** 1 Mountain Research Centre (CIMO), Polytechnic Institute of Bragança, Campus de Sta. Apolónia, Apartado 1172, 5301–855, Bragança, Portugal; 2 Department of Entomology, Texas A&M University, College Station, Texas, 77843–2475, United States of America; 3 Aarhus University, Department of Agroecology, Forsøgsvej 1, 4200, Slagelse, Denmark; University of Cologne, GERMANY

## Abstract

Beekeeping activities, especially queen trading, have shaped the distribution of honey bee (*Apis mellifera*) subspecies in Europe, and have resulted in extensive introductions of two eastern European C-lineage subspecies (*A*. *m*. *ligustica* and *A*. *m*. *carnica*) into the native range of the M-lineage *A*. *m*. *mellifera* subspecies in Western Europe. As a consequence, replacement and gene flow between native and commercial populations have occurred at varying levels across western European populations. Genetic identification and introgression analysis using molecular markers is an important tool for management and conservation of honey bee subspecies. Previous studies have monitored introgression by using microsatellite, PCR-RFLP markers and most recently, high density assays using single nucleotide polymorphism (SNP) markers. While the latter are almost prohibitively expensive, the information gained to date can be exploited to create a reduced panel containing the most ancestry-informative markers (AIMs) for those purposes with very little loss of information. The objective of this study was to design reduced panels of AIMs to verify the origin of *A*. *m*. *mellifera* individuals and to provide accurate estimates of the level of C-lineage introgression into their genome. The discriminant power of the SNPs using a variety of metrics and approaches including the Weir & Cockerham’s F_ST_, an F_ST_-based outlier test, Delta, informativeness (I_n_), and PCA was evaluated. This study shows that reduced AIMs panels assign individuals to the correct origin and calculates the admixture level with a high degree of accuracy. These panels provide an essential tool in Europe for genetic stock identification and estimation of admixture levels which can assist management strategies and monitor honey bee conservation programs.

## Introduction

The role of introgression and admixture in conservation is a dilemma: While natural admixture may be an important evolutionary force in speciation and maintenance of genetic diversity [1–2], admixture induced by human activities may contribute, either directly or indirectly, to the extinction of many taxa [3]. Introduction of species, subspecies and habitat modifications has caused increased rates of admixture with native flora and fauna and introgression that can generate extinction and irretrievable loss of combinations of genotypes throughout the entire genome [4].

The honey bee, *Apis mellifera* L., represents a valuable model to study human-mediated change. Beekeeping has been practiced in Europe for many centuries [5], which has led to loss of native genetic diversity through three major mechanisms: (i) replacement of native populations by human-selected more docile and productive colonies, (ii) spread of honey bee pests and parasites, such as the mite *Varroa destructor* and the microsporidian *Nosema ceranae*, that have contributed to worldwide population declines [6–7], and (iii) recurrent introductions of commercial colonies (reviewed by De la Rúa et al. [8]).

The genetic diversity harbored in native honey bee subspecies is amongst the most important legacies that we can leave to future generations of beekeepers and farmers [9–10]. Native honey bee subspecies are important reservoirs of local adaptations; their extinction means the loss of unique combinations of traits shaped by natural selection over extended periods of time. These combinations can be important for a more sustainable beekeeping, as shown by a recent pan-European experiment [11].

In Europe, honey bees show considerable differences in morphological, behavioural and biological characters across their range as a result of historical patterns of isolation and adaptation to environmental conditions [8]. Those differences are materialized in 10 extant European subspecies, among the 30 subspecies currently recognized worldwide [12–16], representing thereby a substantial component of the total honey bee diversity. These 10 European subspecies have been grouped by morphological and molecular tools [12, 17–21] into two evolutionary lineages: the M-lineage, in Western Europe, and the C-lineage, in Eastern Europe.

Subspecies-specific genetic footprints can still be identified in Europe [22–28], in spite of centuries of beekeeping [5], although introgression and admixture events have also been detected in eastern [28–30] and western [9, 26, 31–32] European populations. The M-lineage *A*. *m*. *mellifera* (dark honey bee) has been recognized as the most threatened, with most of the threat due to introgression from the C-lineage [9, 31–32]. In addition to the documented intentional replacement of *A*. *m*. *mellifera* by *A*. *m*. *carnica* in Germany [33–34], the increasing trade of commercial breeds (mainly C-lineage *A*. *m*. *carnica*, *A*. *m*. *ligustica* and the hybrid buckfast) is threatening the genetic integrity of the native *A*. *m*. *mellifera* as many beekeepers prefer using commercial as opposed to native honey bees.

Increasing awareness that native honey bee diversity represents a valuable asset for sustainable beekeeping is fuelling local breeding and conservation efforts across Europe. One of the earliest, and until recently the single conservation program enacted by law, is that implemented by the Danish Beekeepers Association and the Læsø Beekeepers Association on behalf of the Danish Government in 1993 and the European Union in 1998 [35] to create a reserve and protect the *A*. *m*. *mellifera*. Following approval by the Scottish government of an order to protect the *A*. *m*. *mellifera* on the islands of Colonsay and Oronsay [The Bee Keeping (Colonsay and Oronsay) Order 2013], a second European reserve was recently created in the United Kingdom. Other *A*. *m*. *mellifera* conservation efforts, although not enacted by law, are underway in France, Holland, Norway, Switzerland, Ireland, and Belgium, among others (see the website “http://www.sicamm.org” run by the International Association for the Protection of the European Dark bee). The success or failure of all these efforts will be tightly linked to efforts that monitor the integrity of these protected populations.

Assessing introgression is an important activity in honey bee breeding programs, especially when conservation of native subspecies is a major concern. This activity requires molecular tools that are reliable, inexpensive and preferably automated. Previous studies have monitored introgression between the endemic *A*. *m*. *mellifera* and introduced C-lineage subspecies using microsatellite and PCR-RFLP markers [31–32, 36]. However, with the publication of the honey bee genome [37], development of single-nucleotide polymorphism (SNP) markers [20, 38], and next generation sequencing becoming fast and affordable, particularly for a small genome as that of the honey bee (236 Mb), increasingly powerful tools are available to measure genomic ancestry and admixture levels occurring in both native and introduced honey bee populations [21, 39–40]. However, the genomic approach is not always cost-effective and low quality and/or degraded DNA can be a handicap to using genomic re-sequencing. Alternatively, ancestry can be estimated using a subset of highly informative SNPs ranging in number from a few dozens to several hundreds. The selected SNPs, commonly known as Ancestry-Informative Markers (AIMs), are those that exhibit large allele frequency differences between populations. AIMs can be used for inferring geographic origin of individuals [41–43], detecting illegal trade and translocation of animals [44], food authentication [45], for estimating overall admixture proportions efficiently and inexpensively [43, 46], among others. It is possible, using a panel of AIMs distributed throughout the genome, to estimate the relative ancestral proportions in admixed individuals, and infer the time since the admixture process [47–48].

The ability of an AIMs panel to measure ancestry is generally evaluated empirically, by examining its performance on a given set of samples for which ancestry is known [49]. In this paper, we employed five analytical methods to select different combinations of SNPs to form five nested panels of 48-, 96-, 144-, 192- and 384-AIMs optimized to estimate admixture proportions of C-lineage (*A*. *m*. *ligustica* and *A*. *m*. *carnica*) into the M-lineage *A*. *m*. *mellifera*. This was done in two successive stages. In the first stage, we evaluated the performance of the five selection methods [Weir & Cockerham’s F_ST_, an F_ST_-based outlier test, Delta, informativeness (I_n_), and PCA] on a training dataset, in an effort to select AIMs and to rank them by decreasing level of informativeness. In the second stage, we tested the power of the reduced five designed panels and validated their performance on holdout and simulated sets, by comparing the admixture estimates produced by the panels with those produced by an initial dataset of 1183 SNPs.

## Material and Methods

### Samples, DNA Extraction and SNP Genotyping

A total of 113 honey bee haploid males were collected in 2010 and 2011 across the native range of *A*. *m*. *mellifera*, *A*. *m*. *ligustica* and *A*. *m*. *carnica* in Europe (see the sampling map in Pinto et al. [9]). The samples of *A*. *m*. *mellifera* (N = 77) were collected from apiaries located in England (N = 8), France (N = 15), Belgium (N = 3), Denmark (N = 10), Holland (N = 15), Switzerland (N = 6), Scotland (N = 10), and Norway (N = 10) from protected and unprotected populations [9]. Colonies of protected populations have been identified by morphological (B. Dahle, pers. comm.) and molecular tools (mtDNA tRNA^leu^-cox2 and microsatellites; [31, 32, 50–51]) as the best representatives of *A*. *m*. *mellifera* and have therefore been integrated into conservation programs. To prevent C-lineage introgression and assure pure breeding, these colonies have been maintained in islands or in isolated mating stations. Despite careful management to protect the threatened *A*. *m*. *mellifera* from C-lineage introgression, a recent SNP survey detected variable, although generally low, levels of introgression in these protected populations (see Pinto et al. [9] for details). A reference collection of 36 samples representing C-lineage diversity was obtained from the natural range of *A*. *m*. *carnica* in Serbia (N = 8) and Croatia (N = 11) and from the natural range *A*. *m*. *ligustica* in Italy (N = 17). The owners of all the sampled apiaries gave permission to collect honey bee individuals from the hives. In each location, samples were taken from the inner part of hives, placed into absolute ethanol and stored at −20°C until molecular analysis.

Using a phenol/chloroform isoamyl alcohol (25:24:1) protocol [52], total DNA was extracted from the thorax of the 113 individuals, each representing a single colony. A total of 1536 SNP loci were genotyped for those individuals using Illumina’s BeadArray Technology and the Illumina GoldenGate Assay with a custom Oligo Pool Assay (Illumina, San Diego, CA, USA) following manufacturer’s protocols. The Oligo Pool consisted of the 1536 SNPs, which included the 768 most informative SNPs of Whitfield et al. [20] and 768 newly developed SNPs employed by Chávez-Galarza et al [38]. The 1536 SNP array was used previously to study diversity and introgression levels in populations of *A*. *m*. *mellifera* sampled across Western Europe [9] and to detect signatures of selection in the Iberian honey bee genome [38]. Genotype calling was performed using Illumina’s GenomeStudio Data Analysis software. Of the initial 1536 SNPs, 353 did not meet the quality criteria for analysis and were therefore excluded from the dataset. The SNP filtering was as follows: 124 exhibited poorly separated intensity clusters or low signal intensity when visualized in the GenomeStudio software; 167 were monomorphic (defined by a cut-off criterion of >0.98 for the most common allele, as in Chávez-Galarza et al. [38]) across all populations; 54 did not map in the honey bee genome assembly Amel_4.0; and 8 hit two different genomic positions (the first with 100% identity and the second with 96–98%) in the honey bee genome assembly Amel_4.0 during the mapping process using the 100 bp flanking sequence. Allele frequencies were calculated for each of the remaining 1183 bi-allelic SNPs (S1 Table) in each population using the program Plink [53].

### Selection of AIMs

Five different methods were employed on the initial 1183 SNP dataset for estimating marker information content. The first method, which has been one of the most popular for selecting informative loci, was the pairwise F_ST_ of Weir & Cockerham [54] as calculated at each locus using Genepop software [55]. The second method was the F_ST_–based outlier test developed by Foll & Gaggiotti [56], which employs a Bayesian likelihood approach to detect loci deviating from neutral expectations (outliers). This outlier test was implemented in Bayescan 2.01 [56] using 20 pilot runs of 5 000 iterations (sample size of 5 000 and thinning interval of 10) and an additional burn-in of 50 000 iterations. The third method was based on the estimate of allele-frequency differential (Delta), which is one of the most straightforward ways to evaluate the information content of a SNP. For a bi-allelic marker, like a SNP, the Delta value is estimated as |*p*A_i_—*p*A_j_|, where *p*Ai and *p*A_j_ are the frequencies of allele A in the i^th^ and j^th^ populations, respectively. When more than two populations were analyzed, the Delta value for each SNP locus was estimated as the mean across all pair-wise comparisons. The fourth method was the informativeness for assignment (I_n_, natural logarithm of the number of populations) proposed by Rosenberg et al. [41]. I_n_ provides the amount of information gained about population assignment from observation of a single randomly chosen allele at a locus. This method assumes a uniform prior across K potential source populations for the origin of the allele. For a given set of populations, the minimum value of I_n_ (0) occurs when all alleles have equal frequencies in all populations whereas the maximum value (1) occurs when alleles are not shared among populations. I_n_ was calculated using the software Infocalc available at http://www.stanford.edu/group/rosenberglab/infocalc.html. Finally, the fifth selection method was principal component analysis (PCA), which was performed using the PAST software [57]. The first eight principal components were used to calculate the information content of each SNP following the approach of Paschou et al. [58]. The loadings for each SNP were squared and summed over the eight most significant principal components to produce an estimate of informativeness.

SNPs were ranked and panels of SNPs tested using reference populations and the Anderson’s Simple Training and Holdout method to reduce the potential for upward bias, which is introduced when loci are ranked and assessed using the same individuals [59]. To that end, a total of 34 pure (*sensu* Soland-Reckeweg et al. [32]) individuals of *A*. *m*. *mellifera*, previously identified in Pinto et al. [9], and all reference individuals (17 *A*. *m*. *ligustica* and 19 *A*. *m*. *carnica*) were used for SNP ranking (training set = 70) and the remaining 43 individuals of *A*. *m*. *mellifera* were reserved for panel testing (holdout set = 113). To minimize the effect of clusters of populations on the selection of the AIMs [41, 60–61], the five selection methods were tested using four training datasets. The first dataset consisted of 70 individuals: 34 pure *A*. *m*. *mellifera* and 36 C-lineage individuals, with no distinction between the *A*. *m*. *carnica* and *A*. *m*. *ligustica* subspecies (dataset I). The second dataset consisted of 51 individuals: 34 pure *A*. *m*. *mellifera* and 17 *A*. *m*. *ligustica* (dataset II). The third dataset consisted of 53 individuals: 34 pure *A*. *m*. *mellifera* and 19 *A*. *m*. *carnica* (dataset III). Finally, the fourth dataset consisted of 70 individuals: 34 pure *A*. *m*. *mellifera*, 17 *A*. *m*. *ligustica* and 19 of *A*. *m*. *carnica* (dataset IV).

### Ranking of SNPs

The five selection methods were implemented on the four training datasets producing a total of 20 information content values for each of the 1183 SNPs. These values were ranked and analyzed individually and then were averaged in two steps to obtain a single global value per SNP. In the first step the information content values were averaged across the four training datasets for each of the five selection methods. In the second step the information content values produced by each selection method were converted into a 0–1 scale and then averaged to obtain a global score for each of the 1183 SNPs. After standardizing the values produced by the five selection methods, the global ranking was obtained for the 1183 SNPs using the global score. Given that linked loci yield redundant information, having therefore similar resolving power, markers were excluded if they were within a predefined genetic distance (<1 cM) of higher ranking selected SNPs. The genetic distance of the remaining SNPs ranged from 1.01 to 24.25 cM with a mean of 4.64 cM. Prior to obtaining the global score for each SNP, pairwise associations between information content values produced by the five methods and between the four training datasets were calculated using the Spearman`s rank correlation coefficient, in order to compare the five selection methods and examine the effect of clusters of populations.

### Panel Testing

Five panels of 48-, 96-, 144-, 192- and 384-SNPs (sets defined by multiplex sizes of commercial assays) were designed from the top-ranked SNPs. These nested panels were tested against a holdout set and a simulated set to obtain the admixture proportions estimated by each SNP panel. The holdout set (113 individuals) consisted of 34 pure individuals plus 43 reserved individuals of *A*. *m*. *mellifera* and the reference *A*. *m*. *ligustica* (17 individuals) and *A*. *m*. *carnica* (19 individuals), as described above. The simulated set (1000 individuals) was generated with the program ONCOR [62] using the function “simulate a single mixture”. Ten populations, each with 100 simulated genotypes, were simulated using different levels of introgression (0, 1, 5, 10, 20, 30, 40, 50, 75, and 90%).

Two approaches were used to validate the five reduced AIMs panels. First, a PCA was performed with SNPs in each AIMs panel on the holdout set using the software PAST to generate two-dimensional PCA and to visualize the stability of population assignment produced by the panels. Second, ancestry and admixture was analyzed. Admixture proportions were estimated with SNPs in each AIMs panel for the holdout and simulated sets using a model-based maximum likelihood estimation of individual ancestries implemented in the software Admixture v1.23 [63]. Coancestry spanning 1–6 populations (K = 1–6, using the default termination criterion that stops the runs when the log-likelihood increases by less than Ɛ <0.0001 between iterations) was explored for each AIMs panel and the optimal K was identified with the inferred number of populations producing the lowest cross-validation error (CV) during the clustering analysis.

The performance of each reduced panel was examined using different approaches. First, the pairwise differences between admixture proportions inferred from the initial 1183 SNP dataset and the five panels were tested using a Mann-Whitney test. Second, the precision of each panel was tested against the initial 1183 SNP dataset by calculating linear regression coefficients (r^2^) and the standard deviations of the differences between admixture proportions. Finally, the accuracy of the reduced panels was estimated via percentage of absolute error of admixture estimates obtained with the five panels in relation to the initial 1183 SNP dataset.

## Results

### Identification and Ranking of AIMs

**Fig 1:**
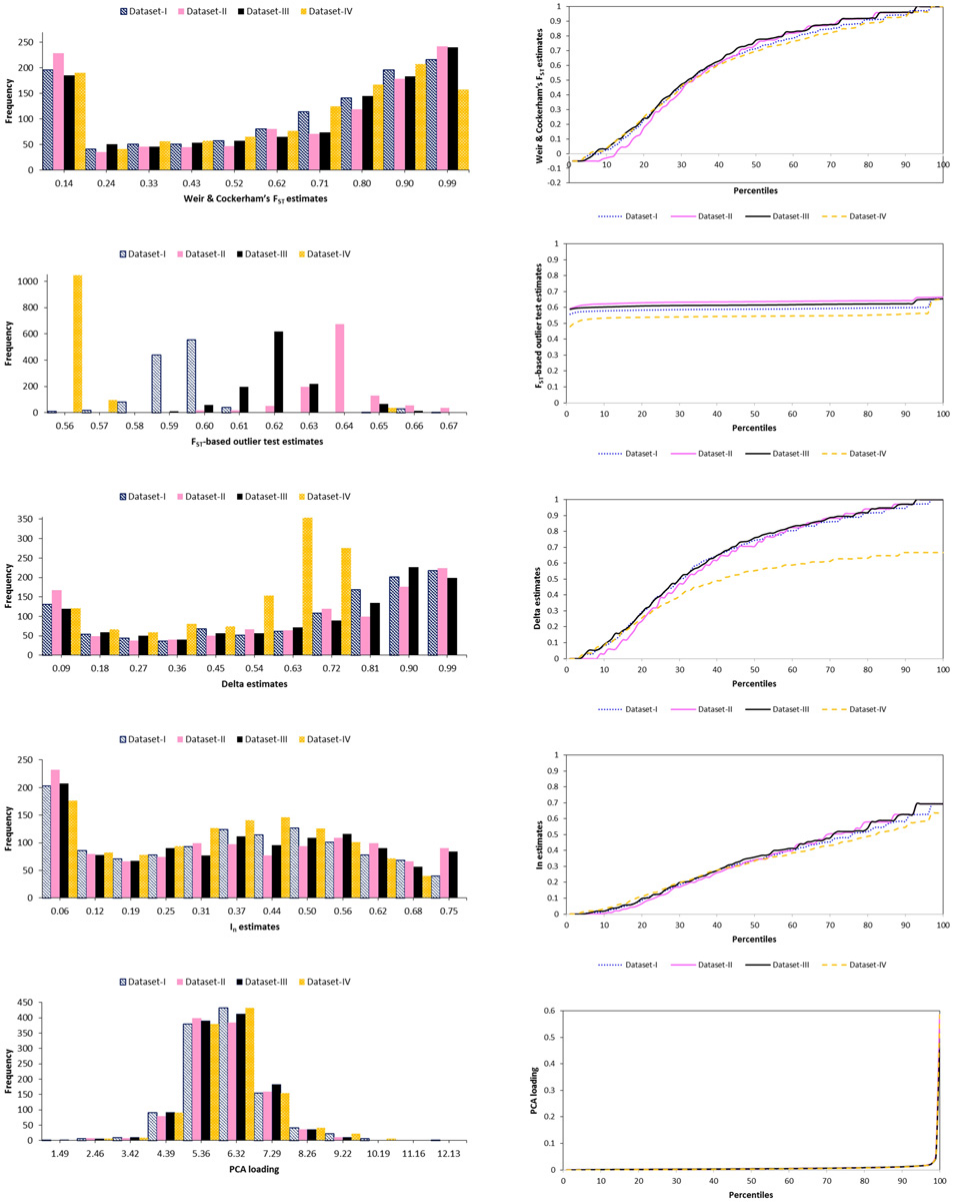
Frequency histograms and percentiles of the estimates of genetic information contained in the initial 1183 SNP dataset. Information content produced by the five selection methods (pairwise Weir & Cockerham’s F_ST_, F_ST_-based outlier test, Delta, I_n_ and PCA) is shown for the four training datasets (I, II, III and IV).

The majority of the 1183 SNPs assessed in this study using five selection methods (pairwise Weir & Cockerham’s F_ST_, F_ST_-based outlier test, Delta, I_n_ and PCA) contain high levels of information content (Fig 1, S2 Table), facilitating the design of reduced panels for genetic identification and introgression analysis in the dark honey bee, *A*. *m*. *mellifera*. The distribution of frequency histograms and percentiles of genetic information content of the 1183 SNPs estimated by each selection method and training dataset are shown in Fig 1. The 50^th^ percentile ranges of the four training datasets were 0.6974–0.7712, 0.5459–0.6362, 0.5532–0.7601, 0.3345–0.3583 and 0.0038–0.0040 for the Weir & Cockerham’s F_ST_, F_ST_-based outlier test, Delta, I_n_ and PCA, respectively, indicating a high level of information content for most SNPs and a similar pattern among the four training datasets (Fig 1).

**Fig 2:**
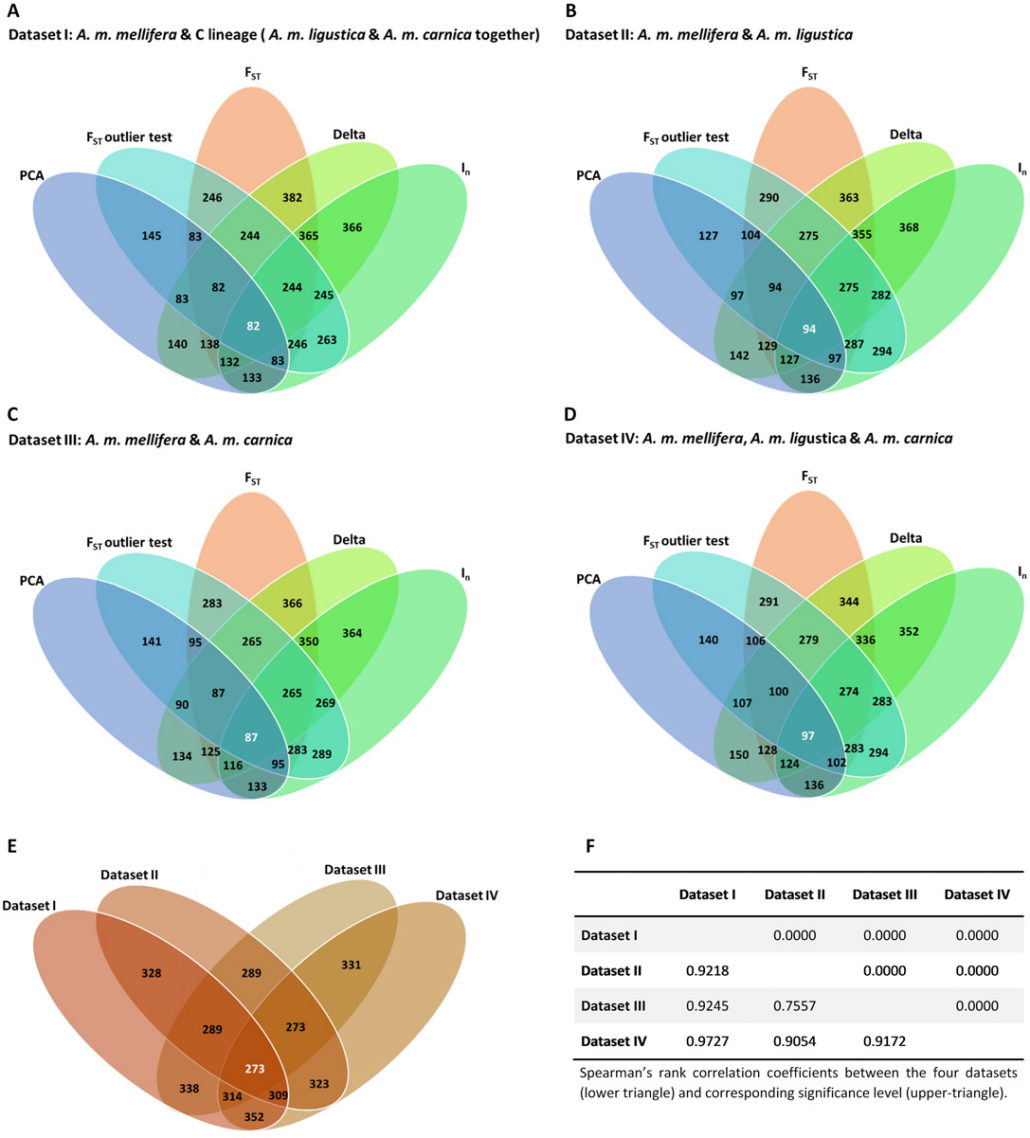
(A-E) Venn diagrams showing the extent of overlap of the top-ranked 384 AIMs. (A-D) Overlap among the five selection methods (pairwise Weir & Cockerham’s F_ST_, F_ST_-based outlier test, Delta, I_n_ and PCA) and the four training datasets (I, II, III and IV). (E) Overlap among the four training datasets, after averaging the information content obtained with the five selection methods, and (F) corresponding Spearman’s rank correlation coefficients.

The level of similarity (Spearman’s rank correlation, *r*_*s*_) between the different estimates of genetic information content produced by the five selection methods across the four training datasets is shown in Table 1. The highest correlation values were observed for Weir & Cockerham’s F_ST_, Delta and I_n_ (0.7648≤*r*_*s*_≤0.9985, P<0.001) whereas a moderate correlation was detected between the F_ST_-based outlier test and Weir & Cockerham’s F_ST_, Delta and I_n_ (0.2864≤*r*_*s*_≤0.6592, P<0.001). The lowest correlations were observed between PCA and the other four methods (-0.2228≤*r*_*s*_≤0.1025, 0.000≤P≤0.9412). Regarding the four training datasets (Fig 2), high correlation values were observed across selection methods (0.7557≤*r*_*s*_≤0.9727, P<0.001).

**Table 1:** Comparison of selection methods and training datasets.

		Dataset I: *A*. *m*. *mellifera* & C lineage (*A*. *m*. *ligustica* & *A*. *m*. *carnica together)*					Dataset II: *A*. *m*. *mellifera* & *A*. *m*. *ligustica*					Dataset III: *A*. *m*. *mellifera* & *A*. *m*. *carnica*					Dataset IV: *A*. *m*. *mellifera A*. *m*. *ligustica & A*. *m*. *carnica*				
		F_ST_	Delta	I_n_	PCA	F_ST_ outlier test	F_ST_	Delta	I_n_	PCA	F_ST_ outlier test	F_ST_	Delta	I_n_	PCA	F_ST_ outlier test	F_ST_	Delta	I_n_	PCA	F_ST_ outlier test
Dataset I	F_ST_		0.0000	0.0000	0.7134	0.0000	0.0000	0.0000	0.0000	0.0620	0.0000	0.0000	0.0000	0.0000	0.6130	0.0000	0.0000	0.0000	0.0000	0.7134	0.0000
	Delta	0.9985		0.0000	0.6139	0.0000	0.0000	0.0000	0.0000	0.0439	0.0000	0.0000	0.0000	0.0000	0.6772	0.0000	0.0000	0.0000	0.0000	0.6139	0.0000
	I_n_	0.9977	0.9957		0.7284	0.0000	0.0000	0.0000	0.0000	0.0753	0.0000	0.0000	0.0000	0.0000	0.4980	0.0000	0.0000	0.0000	0.0000	0.7284	0.0000
	PCA	0.0107	0.0147	0.0101		0.0000	0.9412	0.8104	0.7135	0.0000	0.0000	0.0390	0.0241	0.0357	0.0000	0.0711	0.4901	0.1065	0.5267	0.0000	0.0001
	F_ST_ outlier test	0.5974	0.5778	0.6280	-0.1195		0.0000	0.0000	0.0000	0.0002	0.0000	0.0000	0.0000	0.0000	0.0000	0.0000	0.0000	0.0000	0.0000	0.0000	0.0000
Dataset II	F_ST_	0.9364	0.9370	0.9392	-0.0021	0.5666		0.0000	0.0000	0.3782	0.0000	0.0000	0.0000	0.0000	0.8227	0.0000	0.0000	0.0000	0.0000	0.9412	0.0000
	Delta	0.9338	0.9357	0.9313	-0.0070	0.5205	0.9906		0.0000	0.3980	0.0000	0.0000	0.0000	0.0000	0.7760	0.0000	0.0000	0.0000	0.0000	0.8104	0.0000
	I_n_	0.9329	0.9324	0.9342	-0.0107	0.5581	0.9923	0.9960		0.5187	0.0000	0.0000	0.0000	0.0000	0.8981	0.0000	0.0000	0.0000	0.0000	0.7135	0.0000
	PCA	0.0543	0.0586	0.0517	0.8545	-0.1074	0.0256	0.0246	0.0188		0.0000	0.0017	0.0004	0.0008	0.0000	0.1510	0.0986	0.0426	0.1329	0.0000	0.0000
	F_ST_ outlier test	0.4404	0.4235	0.4637	-0.2018	0.8128	0.4998	0.4732	0.5124	-0.2228		0.0000	0.0000	0.0000	0.0000	0.0000	0.0000	0.0000	0.0000	0.0000	0.0000
Dataset III	F_ST_	0.9328	0.9317	0.9353	0.0600	0.5751	0.7965	0.7705	0.7719	0.0909	0.3301		0.0000	0.0000	0.2647	0.0000	0.0000	0.0000	0.0000	0.0390	0.0000
	Delta	0.9347	0.9353	0.9327	0.0656	0.5337	0.7825	0.7690	0.7663	0.1025	0.2864	0.9925		0.0000	0.4242	0.0000	0.0000	0.0000	0.0000	0.0241	0.0000
	I_n_	0.9331	0.9312	0.9349	0.0611	0.5742	0.7829	0.7648	0.7656	0.0969	0.3150	0.9943	0.9960		0.3212	0.0000	0.0000	0.0000	0.0000	0.0357	0.0000
	PCA	-0.0147	-0.0121	-0.0197	0.6450	-0.1333	-0.0065	0.0083	0.0037	0.5534	-0.1435	-0.0325	-0.0233	-0.0289		0.0000	0.3690	0.3671	0.4471	0.0000	0.0000
	F_ST_ outlier test	0.5524	0.5361	0.5793	-0.0525	0.8862	0.4451	0.3910	0.4189	-0.0418	0.6063	0.6337	0.6006	0.6444	-0.1440		0.0000	0.0000	0.0000	0.0711	0.0000
Dataset IV	F_ST_	0.9883	0.9862	0.9875	0.0201	0.6028	0.9301	0.9216	0.9222	0.0480	0.4567	0.9411	0.9387	0.9384	-0.0261	0.5771		0.0000	0.0000	0.4901	0.0000
	Delta	0.9459	0.9485	0.9474	0.0470	0.5487	0.9177	0.9022	0.9014	0.0590	0.4237	0.9241	0.9169	0.9152	-0.0262	0.5475	0.9683		0.0000	0.1065	0.0000
	I_n_	0.9825	0.9798	0.9832	0.0184	0.6081	0.9313	0.9277	0.9308	0.0437	0.4733	0.9273	0.9279	0.9300	-0.0221	0.5798	0.9948	0.9710		0.5267	0.0000
	PCA	0.0107	0.0147	0.0101	1.0000	-0.1195	-0.0021	-0.0070	-0.0107	0.8545	-0.2018	0.0600	0.0656	0.0611	0.6450	-0.0525	0.0201	0.0470	0.0184		0.0001
	F_ST_ outlier test	0.6043	0.5867	0.6267	-0.1154	0.9119	0.5549	0.5292	0.5634	-0.1250	0.8250	0.5819	0.5589	0.5943	-0.1376	0.8758	0.6344	0.6104	0.6592	-0.1154	

Spearman’s rank correlation coefficients (lower triangle), and corresponding *P*-values (upper triangle), between information content estimates produced by the five selection methods (Weir & Cockerham’s F_ST_, Delta, informativeness (I_n_), PCA, F_ST_-based outlier test) using the four training datasets (I, II, III and IV).

Using an information content cutoff value ≥0.25, which indicates very great genetic differentiation [64], a total of 627 AIMs were identified by the methods of Weir & Cockerham’s F_ST_, Delta, I_n_, and F_ST_-based outlier test. Of these, the top-ranked 384 AIMs were selected using the five methods and the four training datasets. The extent of overlap of the 384 AIMs across the five selection methods and the four training datasets is shown in Fig 2. Overlap between any two methods and across datasets ranged between 382 (Weir & Cockerham’s F_ST_ and Delta for dataset I; Fig 2A) and 134 (Delta and PCA for dataset III; Fig 2C). The number of AIMs that were simultaneously selected by the five methods was lower, ranging from 82 (dataset I; Fig 2A) to 97 (dataset IV; Fig 2D). A substantially higher amount of overlap (273 AIMs; Fig 2E), supported by high correlation values (*r*_s_ ≥0.7557, *P*<0.001; Fig 2F), was observed across the four training datasets, suggesting that the different population groupings have a small effect on the AIMs ranking. The global ranking of the 384 AIMs was used to design reduced panels of 192-, 144-, 96-, and 48 that included SNPs with the highest respective global scores. The performance of these reduced panels was subsequently assessed using the holdout and simulated sets.

### Validation of the AIMs Panels

**Fig 3:**
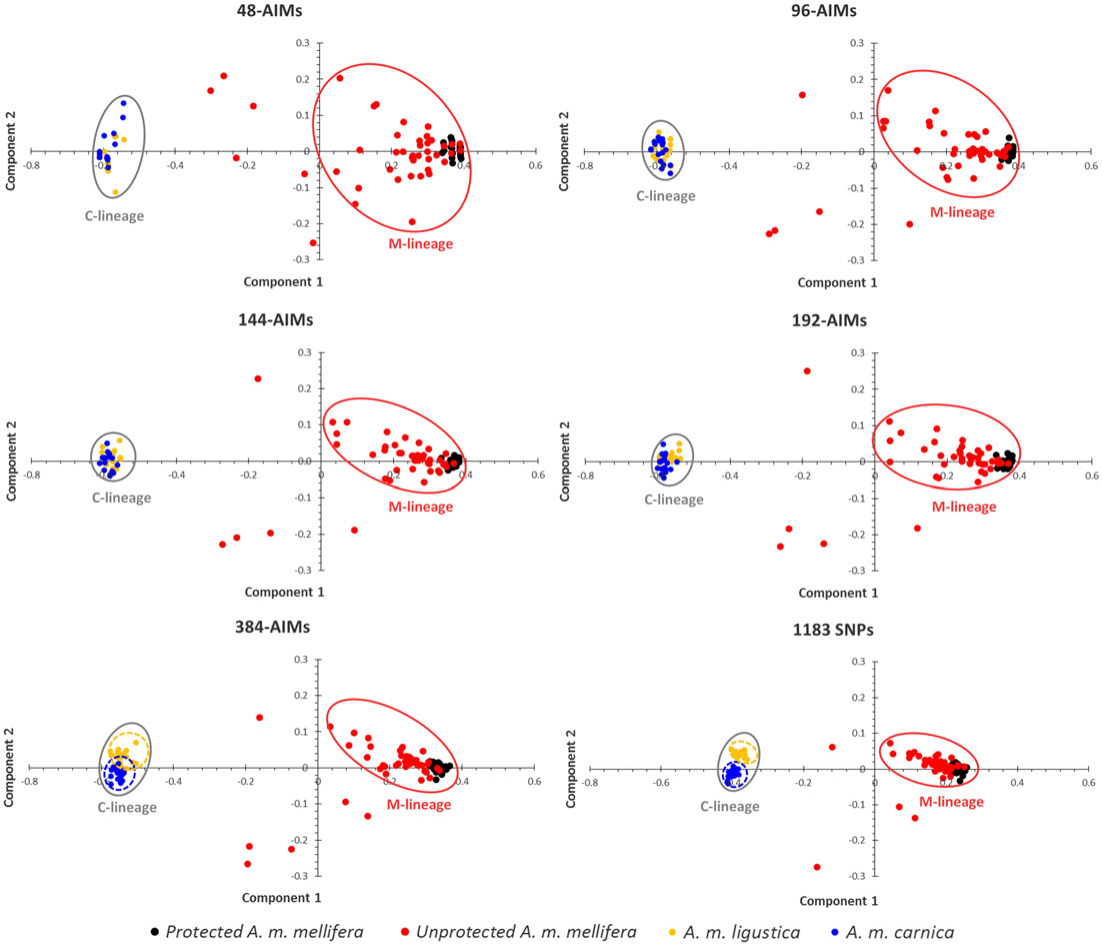
Principal components analysis. Plots obtained for the holdout set using the five AIMs panels (48-, 96-, 144-, 192-, 384-AIMs) and the initial 1183 SNP dataset.

The performance of the five AIMs panels (48-, 96-, 144-, 192-, 384-AIMs) was first validated by using PCA to produce a visual summary of the observed genetic variation carried by the holdout set (Fig 3). The overall diversity pattern is characterized by the presence of two distinct clusters, which are coincidental with the M and C evolutionary lineages. This pattern was captured by every single AIMs panel, although a greater dispersion was observed for the smaller panels. Additionally, the panels with less than 192 AIMs were unable to distinguish the two C-lineage subspecies, *A*.*m*. *ligustica* and *A*.*m*. *carnica*, which were clearly identified by the initial 1183 SNPs and, to a lesser degree, by the 384-AIMs panel.

Ancestry and admixture analyses based on admixture estimates confirm the overall pattern captured by the PCA (S3 Table and S1 Fig). At the optimal K = 2 (inferred by the initial 1183 SNP dataset and the five AIMs panel), the two clusters corresponded to the C and M-lineages. However, C-lineage individuals formed a more homogeneous cluster than those of the M-lineage individuals. While membership proportions in the C-lineage cluster were greater than 95% for the five AIMs panels, the M-lineage cluster comprised 13 (384-AIMs and 1183 SNPs), 14 (48- and 192-AIMs) and 15 (96- and 144-AIMs) individuals with membership proportions lower than 85%, a pattern that was already evident in the PCA plots.

The introgression levels exhibited by individuals of the M-lineage cluster were significantly higher (Student's t-test, *P*<0.001) in unprotected (13.76–15.18%, with 1183 SNPs and 48 AIMs, respectively) than in protected individuals (0.08–0.52%, with 96 AIMs and 1183 SNPs, respectively) for any AIMs panel. The overall estimates of C-lineage introgression into *A*. *m*. *mellifera* varied with the panel (8.4, 7.9, 7.8, 7.9, 7.5 and 7.7% with 48-, 96-, 144-, 192-, 384-AIMs and 1183 SNPs, respectively), although the differences were not statistically significant (Mann-Whitney test, 0.8225≤ *P* ≤0.9983; S4 Table).

In addition to the admixture analyses using the holdout set, the AIMs panels were further validated using a simulated set of 10 different levels of C-lineage introgression (0, 1, 5, 10, 20, 30, 40, 50, 75, and 90%). As for the analyses with the holdout set, the simulated set produced two clusters corresponding to M and C lineages with no significant differences in admixture proportions between the different AIMs panels and the initial 1183 SNP dataset (Mann-Whitney test, *P* ≥0.2313; S5 Table).

### Assignment’s precision and accuracy

**Fig 4:**
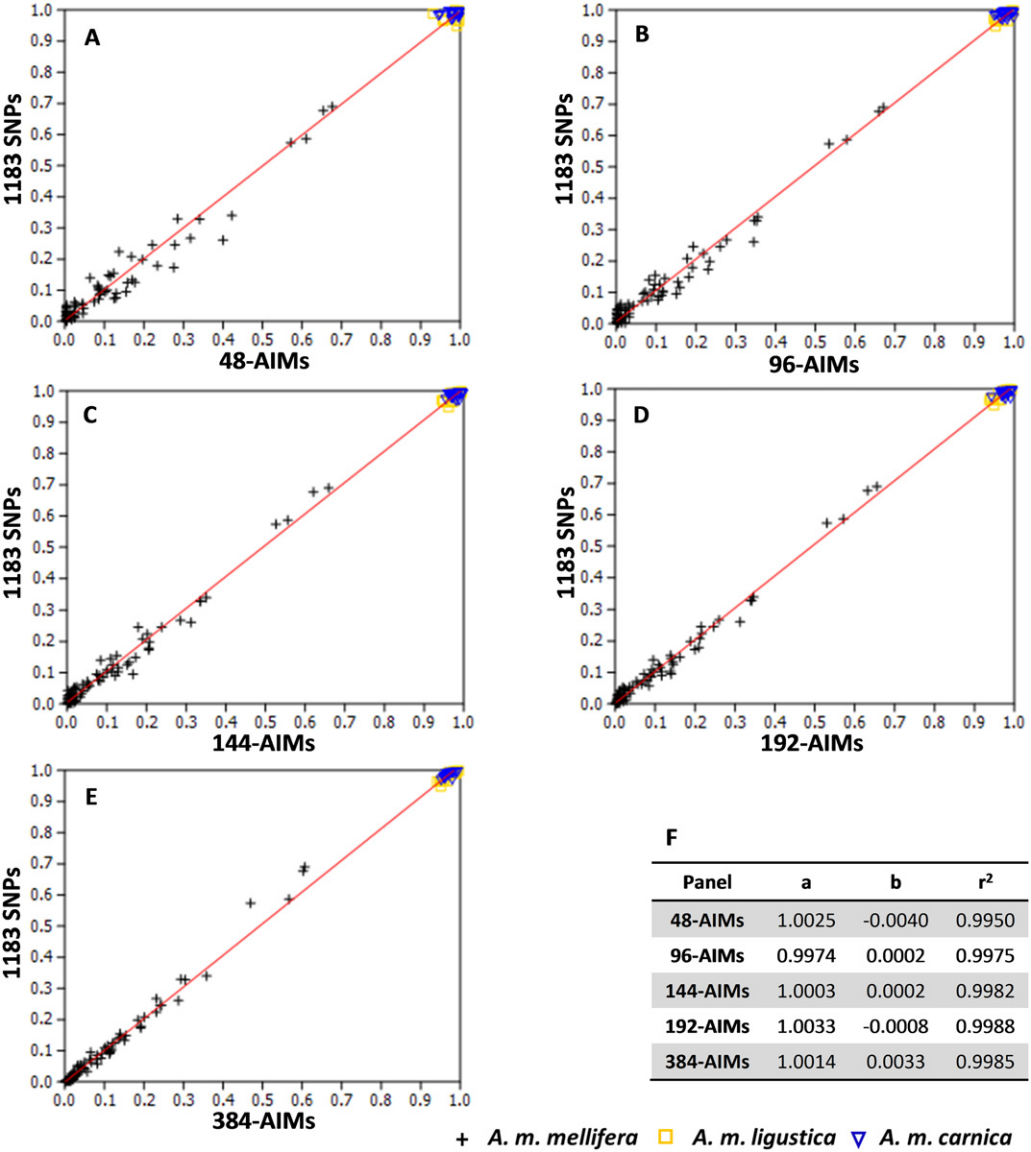
Linear regression. (A-E) Plots between admixture proportions inferred from the initial 1183 SNP dataset and those inferred from the five AIMs panels (48-, 96-, 144-, 192-, 384-AIMs) using individuals of the holdout set. (F) Parameters and coefficients for each AIMs panel.

The power of the reduced AIMs panels in identifying *A*. *m*. *mellifera* and estimating admixture proportions was evaluated on the holdout set. Estimates of C-lineage introgression into *A*. *m*. *mellifera* inferred from the five panels were greatly concordant with those inferred from the initial 1183 SNP dataset, as indicated by the high correlation values (r ≥0.997; Fig 4). Despite the high correlations obtained for each comparison, the error rate in admixture estimates, which is very low for all the panels (0.0012–0.0042 with the simulated set and 0.4–1.3 with the holdout set), does increase as the size of the panel decreases (S2 Fig). Nevertheless, the reduced AIMs panels provide good precision in estimating admixture proportions.

**Fig 5:**
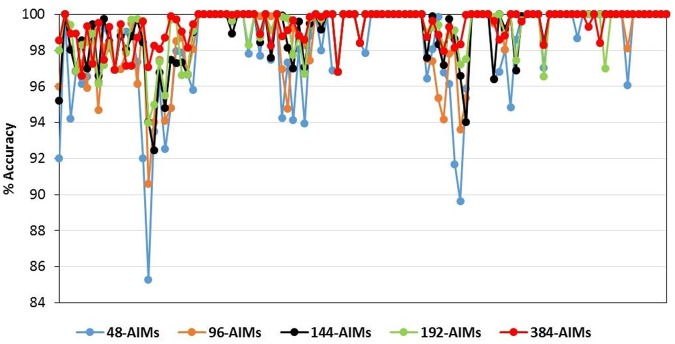
Assignment accuracy. Percentage obtained with the five AIMs panels (48-, 96-, 144-, 192-, 384-AIMs) for each of the 113 individuals of the holdout set.

As another assessment of the performance of the panels, the accuracy was calculated via absolute error. The success of assignment of the 113 individual genotypes of the holdout set to genetic origin and level of admixture inferred from the different AIMs panels is shown in Fig 5. The average percentage of correct assignment was high varying from 98.2, 98.8, 99.0, 99.2 to 99.4% for the 48-, 96-, 144-, 192- and 384-AIMs panels, respectively. The chosen AIMs panels accurately distinguish M/C admixture, therefore these results suggest that a small number of AIMs are sufficient to identify *A*. *m*. *mellifera* and estimate introgression from C-lineage colonies with great accuracy.

## Discussion

The recognition that native honey bee genetic diversity is fundamental for sustainable beekeeping and for facing the challenges of a rapidly changing world (e.g. climate change, novel diseases and parasites) is stimulating implementation of conservation programs across Europe in an attempt to recover and protect *A*. *m*. *mellifera*, which is the European honey bee subspecies with the widest natural range [12], and at the same time the most threatened by introgression [9, 31]. The need of a reliable, high-throughput, and cost-effective tool for identifying candidate *A*. *m*. *mellifera* colonies targeted for conservation, a crucial step when managing conservatoires, motivated the design of reduced AIMs panels containing the most informative SNPs to verify ancestry and introgression from C-lineage subspecies. In this study we developed, validated and tested the first reduced AIMs panels for honey bees. Our results provide strong confidence in a panel of 384 AIMs and show that even smaller subsets of 192-, 144-, 96- and 48-AIMs are able to identify ancestry and estimate introgression with great accuracy. These reduced panels promise to be a useful tool for routine identification of *A*. *m*. *mellifera* colonies maintained in the breeding populations of conservation programs.

The AIMs included in the five reduced panels were simultaneously selected by pairwise Weir & Cockerham’s F_ST_, F_ST_-based outlier test, Delta, I_n_ and PCA, in order to balance out the limitations of each individual method [41, 58, 65]. These selection methods have proved to be powerful, although with varying performances, in identifying population informative markers in a wide range of organisms [43, 58, 60–61, 65]. A great extent of overlap of top-ranked AIMs was obtained for the five selection methods, especially for pairwise Weir & Cockerham’s F_ST_, Delta, and I_n_ suggesting that they capture the same information. Nonetheless, the smaller panels (48-, 96-, 144-, 192-AIMs) did not necessarily include all AIMs simultaneously detected by the five methods as the global ranking depended on the average score. High pairwise correlation values were obtained for Weir & Cockerham’s F_ST_, Delta and I_n_ but not for PCA, as found by Wilkinson et al. [65]. PCA has been recommended for ranking markers because it has the advantage of generating an overall estimate for a single SNP locus whereas the other methods require estimate of an average from pairwise calculations when the number of populations is greater than two [58].

The five reduced panels tested with the holdout and simulated sets performed virtually as well as the initial 1183 SNP dataset, as revealed by the strong correlations obtained between admixture estimates and low associated error rates. The assignment power was high across the five panels with average values of correct assignment varying between 98.2 and 99.4%, although the accuracy decreased slightly with panel size. Nonetheless, even the 48-AIMs panel exhibited high accuracy levels, which is not surprising as it includes the AIMs with the greatest resolution power. Studies on other organisms have also found good performances with panels of similar sizes [43, 45, 60, 65], detecting sharp drops in accuracy for a number of SNPs below 25 [45, 60].

Evaluation of different combinations of the focal *A*. *m*. *mellifera* and the two most common sources of foreign genes, *A*. *m*. *ligustica* and *A*. *m*. *carnica*, revealed a negligible effect of population groupings on the AIMs ranking. These results suggest that the designed panels are suited for identifying and assessing introgression of *A*. *m*. *ligustica*, *A*. *m*. *carnica* or both into *A*. *m*. *mellifera*. While these panels will possibly perform well in the presence of other C-lineage subspecies, more complex combinations that include sources of different evolutionary lineages will require further testing and, most likely, new panels developed from broader baseline datasets. Additionally, it should be noted, that these reduced panels are not suitable for standard population genetic analyses, including determining allelic diversity or measuring isolation by distance, genetic drift or bottleneck effect. The bias introduced through selection for markers that segregate among target populations would seriously compromise these calculations [66–67].

Ancestry identification of honey bee subspecies is undergoing steady development (reviewed by Meixner et al. [68]) from classical morphometry, analysis of allozymes, mitochondrial DNA, nuclear microsatellites, and now SNP tools. Because researchers must balance the cost of genotyping many samples versus many loci, herein we developed five nested reduced panels that include AIMs with the highest resolution power for discriminating subspecies of the divergent M and C evolutionary lineages. While the 384-AIMs panel is also capable of discriminating the C-lineage *A*. *m*. *ligustica* and *A*. *m*. *carnica*, for estimating C-lineage introgression into *A*. *m*. *mellifera* we recommend using the 96-AIMs panel because it is accurate; and high-throughput 96-plex genotyping assays can be outsourced at an affordable cost ($8 900 for 480 samples), representing a saving of 92.4% when compared with the 1536-plex assay ($116 800 for 480 samples).

In conclusion, the proposed AIMs panels can be actively used as a tool in conservation management of *A*. *m*. *mellifera* populations that suffer from hybridization and introgression with the most commonly introduced and beekeepers’ preferred *A*. *m*. *ligustica* and *A*. *m*. *carnica* subspecies. This can be an important advance because the current European regulation on organic beekeeping states that “preference shall be given to the use of European breeds of *Apis mellifera* and their local ecotypes” and several conservation programs have been undertaken in Europe (reviewed by De la Rúa et al. [8]). The use of these panels will apply well to monitoring, management and conservation programs of *A*. *m*. *mellifera* in Western Europe, which usually require high-sample throughput, and will be a resource for the honey bee community to obtain accurate genetic information at reduced costs.

## Supporting Information

S1 Fig Ancestry estimates. Global estimates (y-axis), for the 113 individuals of the holdout set (x-axis), inferred from the five AIMs panels (48-, 96-, 144-, 192-, 384-AIMs) and the initial 1183 SNP dataset using the model-based approach implemented in the ADMIXTURE software. Results are shown for the optimal *K* = 2, which distinguishes the M (red) and C (cyan) evolutionary lineages of *A*. *mellifera*.
(TIFF)

S2 Fig Standard deviation (SD) of admixture proportions. Precision estimates obtained using the SD of the differences between admixture proportions inferred from the initial 1183 SNP dataset and the five AIMs panels (48-, 96-, 144-, 192-, 384-AIMs) using the holdout (blue line) and simulated (orange line) sets.
(TIFF)

S1 Table Input file containing the 1183 coded SNPs for the 113 honey bee samples. (XLSX)

S2 Table Information content values of the initial 1183 SNP dataset estimated by the five selection methods (Weir & Cockerham’s F_ST_, Delta, informativeness (I_n_), PCA and the F_ST_-based outlier test) and for the four training datasets (I to IV). The SNPs are ordered from high to low information content. The top 48, 96, 144, 192 and 384 SNPs were included in the five reduced panels. SNPs marked with an asterisk (*) were excluded from the reduced panels because they were within a genetic distance <1 cM of other informative SNPs.
(DOCX)

S3 Table Admixture proportion estimates inferred from the five AIMs panels (48-, 96-, 144-, 192-, 384-AIMs) and the initial 1183 SNP dataset for the holdout set. The holdout set consisted of 34 pure (training set) and 43 reserved individuals of *A*. *m*. *mellifera* and all reference individuals of *A*. *m*. *ligustica* (17) and *A*. *m*. *carnica* (19). * Samples marked with an asterisk (*) are of *A*. *m*. *mellifera* from protected populations (pure breeding for conservation purposes; see Pinto et al. 2014 [9] for details).
(DOCX)

S4 Table *P*-values of Mann-Whitney pairwise several-sample-test. Values obtained from comparing individual admixture proportions estimated with the five AIMs panels (48-, 96-, 144-, 192-, 384-AIMs) and the initial 1183 SNP dataset using the holdout set.
(DOCX)

S5 Table *P*-values of Mann-Whitney pairwise several-sample-test. Values obtained from comparing admixture proportions inferred from the five AIMs panels and the 1183 initial SNP dataset using the simulated set. The simulated set was generated with the program ONCOR (Kalinowski et al. 2007) using the function “simulate a single mixture”. Ten populations, each with 100 genotypes, were simulated using different levels of C-lineage introgression (0, 1, 5, 10, 20, 30, 40, 50, 75, and 90%).
(DOCX)
