# Supplementary figures and images for: Reduced SNP Panels for Genetic Identification and Introgression Analysis in the Dark Honey Bee (Apis mellifera mellifera)
Source: PLoS One. 2015 Apr 13;10(4):e0124365. doi: 10.1371/journal.pone.0124365 (PMC4395157; doi:10.1371/journal.pone.0124365)

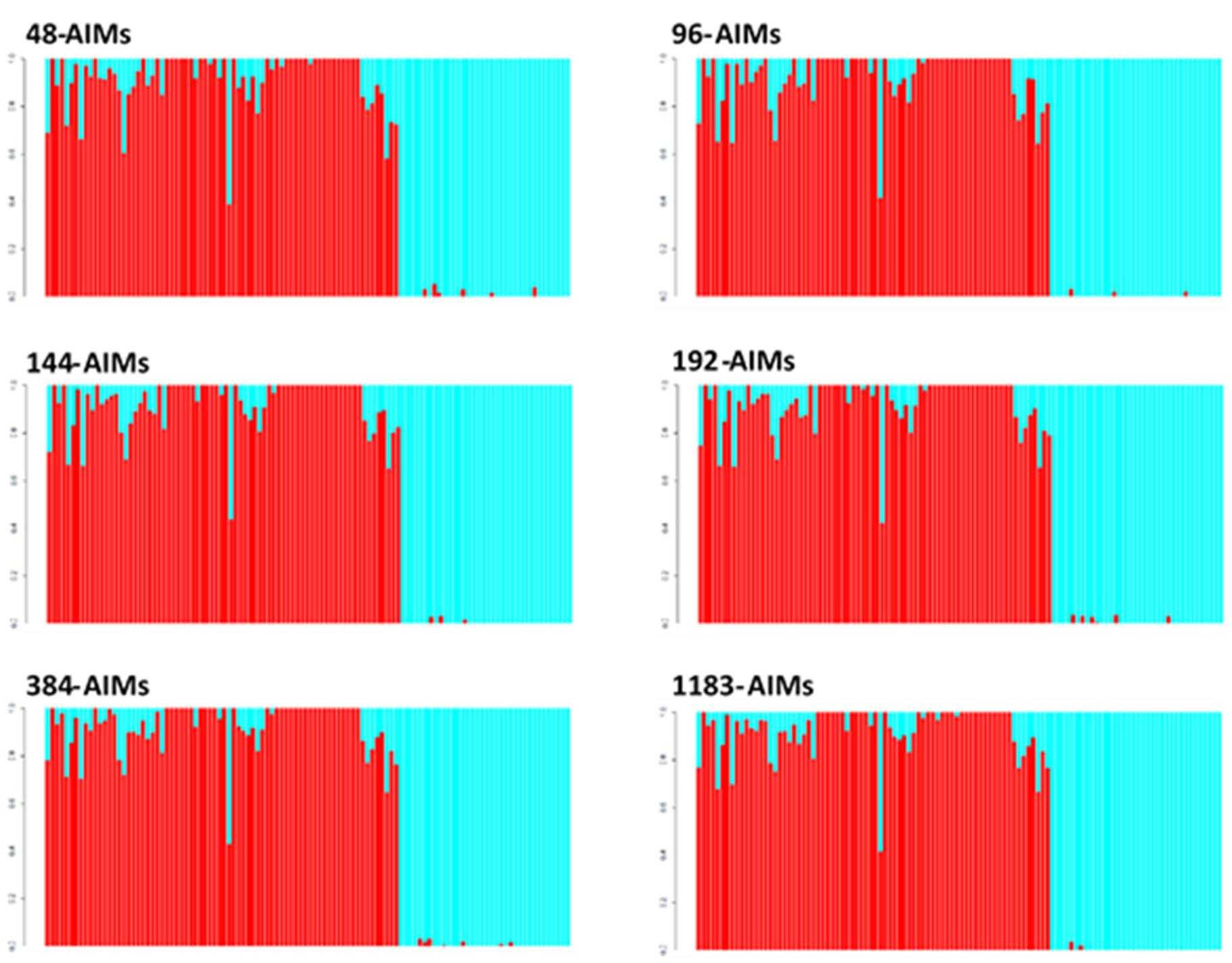

Supplement: S1 Fig — Global estimates (y-axis), for the 113 individuals of the holdout set (x-axis), inferred from the five AIMs panels (48-, 96-, 144-, 192-, 384-AIMs) and the initial 1183 SNP dataset using the model-based approach implemented in the ADMIXTURE software. Results are shown for the optimal K = 2, which distinguishes the M (red) and C (cyan) evolutionary lineages of A. mellifera. (TIFF) [file pone.0124365.s001.tiff]

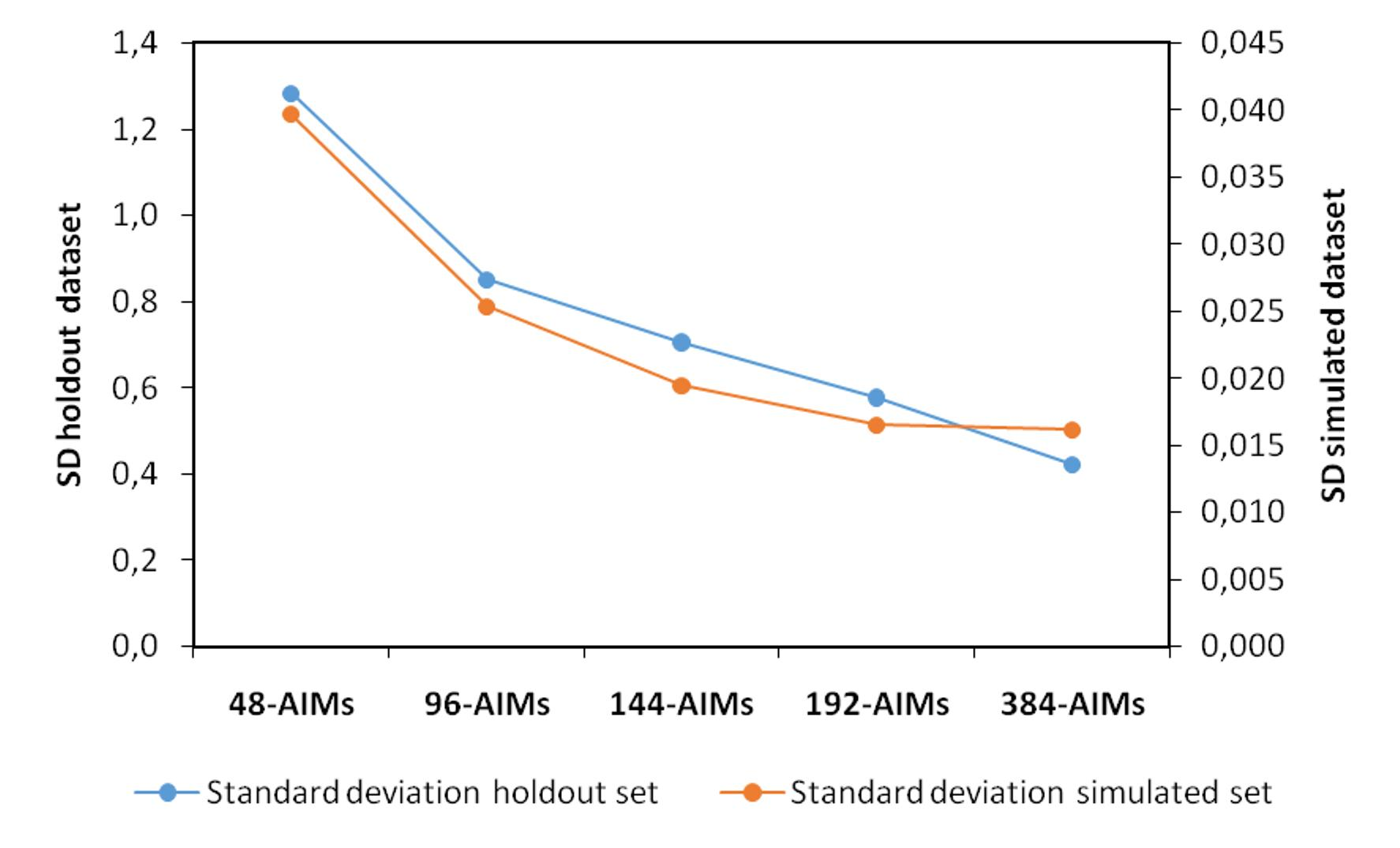

Supplement: S2 Fig — Precision estimates obtained using the SD of the differences between admixture proportions inferred from the initial 1183 SNP dataset and the five AIMs panels (48-, 96-, 144-, 192-, 384-AIMs) using the holdout (blue line) and simulated (orange line) sets. (TIFF) [file pone.0124365.s002.tiff]
