# Supplementary material for: Reduced SNP Panels for Genetic Identification and Introgression Analysis in the Dark Honey Bee (Apis mellifera mellifera)
Source: PLoS One. 2015 Apr 13;10(4):e0124365. doi: 10.1371/journal.pone.0124365 (PMC4395157; doi:10.1371/journal.pone.0124365)
Supplement: S2 Table — The SNPs are ordered from high to low information content. The top 48, 96, 144, 192 and 384 SNPs were included in the five reduced panels. SNPs marked with an asterisk (*) were excluded from the reduced panels because they were within a genetic distance <1 cM of other informative SNPs. (DOCX) [file pone.0124365.s004.docx]

**S2 Table.** **Information content values of the initial 1183 SNP dataset estimated by the five selection methods (Weir & Cockerham’s F_ST_, Delta, informativeness (I_n_), PCA and the F_ST_-based outlier test) and for the four training datasets (I to IV).**

| **SNP code** | **Dataset I:  *A. m. mellifera* & C lineage ( *A. m. ligustica* & *A. m. carnica together)*** | | |  |  | **Dataset II: *A. m. mellifera* & *A. m. ligustica*** | | | | | **Dataset III: *A. m. mellifera* & *A. m. carnica*** | | | | | | **Dataset IV: *A. m. mellifera, A. m. ligustica* & *A. m. carnica*** | | | | |
| --- | --- | --- | --- | --- | --- | --- | --- | --- | --- | --- | --- | --- | --- | --- | --- | --- | --- | --- | --- | --- | --- |
|  | **F_ST_** | **Delta** | **I_n_** | **PCA** | **F_ST_ outlier test** | **F_ST_** | **Delta** | **I_n_** | **PCA** | **F_ST_ outlier test** | **F_ST_** | **Delta** | **I_n_** | **PCA** | **F_ST_ outlier test** | **F_ST_** | | **Delta** | **I_n_** | **PCA** | **F_ST_ outlier test** |
| ahb12266 | 1.0000 | 1.0000 | 0.693147 | 0.135147 | 0.66181 | 1.0000 | 1.00000 | 0.693147 | 0.144920 | 0.66381 | 1.0000 | 1.00000 | 0.693147 | 0.14474 | 0.65200 | 1.0000 | | 0.66667 | 0.636514 | 0.135147 | 0.64861 |
| AMB-00453240 | 1.0000 | 1.0000 | 0.693147 | 0.013499 | 0.65718 | 1.0000 | 1.00000 | 0.693147 | 0.011540 | 0.66316 | 1.0000 | 1.00000 | 0.693147 | 0.00526 | 0.65047 | 1.0000 | | 0.66667 | 0.636514 | 0.013499 | 0.64859 |
| *AMB-00546972 | 1.0000 | 1.0000 | 0.693147 | 0.020781 | 0.64685 | 1.0000 | 1.00000 | 0.693147 | 0.024340 | 0.65963 | 1.0000 | 1.00000 | 0.693147 | 0.00382 | 0.64581 | 1.0000 | | 0.66667 | 0.636514 | 0.020781 | 0.63765 |
| ahb7299 | 1.0000 | 1.0000 | 0.693147 | 0.010630 | 0.65780 | 1.0000 | 1.00000 | 0.693147 | 0.010720 | 0.66313 | 1.0000 | 1.00000 | 0.693147 | 0.00503 | 0.65330 | 1.0000 | | 0.66667 | 0.636514 | 0.010630 | 0.64656 |
| AMB-00348118 | 1.0000 | 1.0000 | 0.693147 | 0.005749 | 0.64354 | 1.0000 | 1.00000 | 0.693147 | 0.008150 | 0.65842 | 1.0000 | 1.00000 | 0.693147 | 0.00797 | 0.64736 | 1.0000 | | 0.66667 | 0.636514 | 0.005749 | 0.62698 |
| ahb2883 | 1.0000 | 1.0000 | 0.693147 | 0.005332 | 0.65898 | 1.0000 | 1.00000 | 0.693147 | 0.006000 | 0.66454 | 1.0000 | 1.00000 | 0.693147 | 0.00715 | 0.65264 | 1.0000 | | 0.66667 | 0.636514 | 0.005332 | 0.65230 |
| ahb6949 | 0.9697 | 0.9697 | 0.624791 | 0.017922 | 0.59797 | 0.9587 | 0.96970 | 0.624791 | 0.028410 | 0.64162 | 0.9595 | 0.96970 | 0.624791 | 0.02043 | 0.62219 | 0.9613 | | 0.64647 | 0.584017 | 0.017922 | 0.55535 |
| ahb3753 | 0.9692 | 0.9688 | 0.623128 | 0.133899 | 0.59838 | 0.9570 | 0.96875 | 0.623128 | 0.140560 | 0.64138 | 0.9595 | 0.96875 | 0.623128 | 0.13923 | 0.62184 | 0.9605 | | 0.64583 | 0.582695 | 0.133899 | 0.55560 |
| ahb1644 | 0.9706 | 0.9706 | 0.626369 | 0.014162 | 0.59837 | 0.9596 | 0.97059 | 0.626369 | 0.016110 | 0.64127 | 0.9611 | 0.97059 | 0.626369 | 0.01703 | 0.62173 | 0.9625 | | 0.64706 | 0.585271 | 0.014162 | 0.55450 |
| *est6726 | 1.0000 | 1.0000 | 0.693147 | 0.006893 | 0.65885 | 1.0000 | 1.00000 | 0.693147 | 0.006230 | 0.66304 | 1.0000 | 1.00000 | 0.693147 | 0.00366 | 0.65104 | 1.0000 | | 0.66667 | 0.636514 | 0.006893 | 0.64904 |
| est8927 | 0.9413 | 0.9444 | 0.584324 | 0.070832 | 0.59980 | 0.9150 | 0.88235 | 0.505105 | 0.071560 | 0.64073 | 1.0000 | 1.00000 | 0.693147 | 0.04570 | 0.65147 | 0.9308 | | 0.66667 | 0.539562 | 0.070832 | 0.56166 |
| AMB-00981357 | 0.9706 | 0.9722 | 0.629296 | 0.008080 | 0.60076 | 0.9584 | 0.94118 | 0.579557 | 0.006390 | 0.64150 | 1.0000 | 1.00000 | 0.693147 | 0.01366 | 0.65056 | 0.9638 | | 0.66667 | 0.574676 | 0.008080 | 0.56487 |
| *ahb2567 | 0.9413 | 0.9444 | 0.584324 | 0.042469 | 0.59842 | 0.9150 | 0.88235 | 0.505105 | 0.027280 | 0.64064 | 1.0000 | 1.00000 | 0.693147 | 0.01347 | 0.65265 | 0.9308 | | 0.66667 | 0.539562 | 0.042469 | 0.56185 |
| AMB-00632040 | 1.0000 | 1.0000 | 0.693147 | 0.006453 | 0.65766 | 1.0000 | 1.00000 | 0.693147 | 0.006650 | 0.66176 | 1.0000 | 1.00000 | 0.693147 | 0.00245 | 0.64932 | 1.0000 | | 0.66667 | 0.636514 | 0.006453 | 0.64362 |
| *est6301 | 0.9706 | 0.9706 | 0.626369 | 0.007281 | 0.59774 | 0.9596 | 0.97059 | 0.626369 | 0.014190 | 0.64232 | 0.9611 | 0.97059 | 0.626369 | 0.01175 | 0.62220 | 0.9625 | | 0.64706 | 0.585271 | 0.007281 | 0.55612 |
| AMB-01090245 | 1.0000 | 1.0000 | 0.693147 | 0.005342 | 0.65666 | 1.0000 | 1.00000 | 0.693147 | 0.008190 | 0.66443 | 1.0000 | 1.00000 | 0.693147 | 0.00170 | 0.65307 | 1.0000 | | 0.66667 | 0.636514 | 0.005342 | 0.64980 |
| AMB-00713138 | 0.9271 | 0.9444 | 0.584324 | 0.255169 | 0.59906 | 0.8846 | 0.88235 | 0.505105 | 0.270270 | 0.63862 | 1.0000 | 1.00000 | 0.693147 | 0.26731 | 0.64676 | 0.9028 | | 0.66667 | 0.539562 | 0.255169 | 0.56179 |
| est8764 | 1.0000 | 1.0000 | 0.693147 | 0.004066 | 0.66057 | 1.0000 | 1.00000 | 0.693147 | 0.003970 | 0.66351 | 1.0000 | 1.00000 | 0.693147 | 0.00535 | 0.65290 | 1.0000 | | 0.66667 | 0.636514 | 0.004066 | 0.65023 |
| est6253 | 0.9706 | 0.9706 | 0.626369 | 0.008735 | 0.59839 | 0.9596 | 0.97059 | 0.626369 | 0.008880 | 0.64075 | 0.9611 | 0.97059 | 0.626369 | 0.01053 | 0.62155 | 0.9625 | | 0.64706 | 0.585271 | 0.008735 | 0.55669 |
| AMB-00605402 | 0.9413 | 0.9444 | 0.584324 | 0.016422 | 0.59839 | 0.9150 | 0.88235 | 0.505105 | 0.012960 | 0.63993 | 1.0000 | 1.00000 | 0.693147 | 0.00940 | 0.65059 | 0.9308 | | 0.66667 | 0.539562 | 0.016422 | 0.56347 |
| AMB-00861482 | 0.9688 | 0.9697 | 0.624791 | 0.007459 | 0.60055 | 0.9575 | 0.94118 | 0.579557 | 0.007370 | 0.64185 | 1.0000 | 1.00000 | 0.693147 | 0.00629 | 0.64680 | 0.9618 | | 0.66667 | 0.574676 | 0.007459 | 0.56353 |
| est1030 | 1.0000 | 1.0000 | 0.693147 | 0.003986 | 0.66352 | 1.0000 | 1.00000 | 0.693147 | 0.003970 | 0.66385 | 1.0000 | 1.00000 | 0.693147 | 0.00382 | 0.65076 | 1.0000 | | 0.66667 | 0.636514 | 0.003986 | 0.64898 |
| ahb10840 | 0.9413 | 0.9444 | 0.584324 | 0.016903 | 0.59924 | 0.9150 | 0.88235 | 0.505105 | 0.021160 | 0.64048 | 1.0000 | 1.00000 | 0.693147 | 0.00445 | 0.65277 | 0.9308 | | 0.66667 | 0.539562 | 0.016903 | 0.56161 |
| ahb7978 | 0.9702 | 0.9714 | 0.627869 | 0.004731 | 0.60103 | 1.0000 | 1.00000 | 0.693147 | 0.004110 | 0.66291 | 0.9602 | 0.94737 | 0.588665 | 0.03994 | 0.62162 | 0.9632 | | 0.66667 | 0.579257 | 0.004731 | 0.56306 |
| *ahb2974 | 1.0000 | 1.0000 | 0.693147 | 0.003765 | 0.65921 | 1.0000 | 1.00000 | 0.693147 | 0.005840 | 0.66294 | 1.0000 | 1.00000 | 0.693147 | 0.00222 | 0.65433 | 1.0000 | | 0.66667 | 0.636514 | 0.003765 | 0.64685 |
| AMB-00977845 | 1.0000 | 1.0000 | 0.693147 | 0.003765 | 0.65936 | 1.0000 | 1.00000 | 0.693147 | 0.005840 | 0.66171 | 1.0000 | 1.00000 | 0.693147 | 0.00222 | 0.65119 | 1.0000 | | 0.66667 | 0.636514 | 0.003765 | 0.64226 |
| AMB-00220846 | 1.0000 | 1.0000 | 0.693147 | 0.003944 | 0.66230 | 1.0000 | 1.00000 | 0.693147 | 0.003640 | 0.66256 | 1.0000 | 1.00000 | 0.693147 | 0.00317 | 0.65011 | 1.0000 | | 0.66667 | 0.636514 | 0.003944 | 0.64979 |
| ahb1933 | 1.0000 | 1.0000 | 0.693147 | 0.003906 | 0.66082 | 1.0000 | 1.00000 | 0.693147 | 0.003840 | 0.66371 | 1.0000 | 1.00000 | 0.693147 | 0.00267 | 0.64996 | 1.0000 | | 0.66667 | 0.636514 | 0.003906 | 0.65147 |
| est5929 | 0.9706 | 0.9722 | 0.629296 | 0.004761 | 0.60050 | 0.9584 | 0.94118 | 0.579557 | 0.006830 | 0.64183 | 1.0000 | 1.00000 | 0.693147 | 0.00472 | 0.65287 | 0.9638 | | 0.66667 | 0.574676 | 0.004761 | 0.56679 |
| AMB-01056372 | 0.9413 | 0.9444 | 0.584324 | 0.009649 | 0.59788 | 0.9150 | 0.88235 | 0.505105 | 0.005420 | 0.63972 | 1.0000 | 1.00000 | 0.693147 | 0.01764 | 0.65244 | 0.9308 | | 0.66667 | 0.539562 | 0.009649 | 0.56241 |
| *ahb1080 | 1.0000 | 1.0000 | 0.693147 | 0.003267 | 0.65954 | 1.0000 | 1.00000 | 0.693147 | 0.003700 | 0.66277 | 1.0000 | 1.00000 | 0.693147 | 0.00247 | 0.65233 | 1.0000 | | 0.66667 | 0.636514 | 0.003267 | 0.64500 |
| est2664 | 0.9706 | 0.9706 | 0.626369 | 0.004715 | 0.59886 | 0.9596 | 0.97059 | 0.626369 | 0.006390 | 0.64157 | 0.9611 | 0.97059 | 0.626369 | 0.00898 | 0.62131 | 0.9625 | | 0.64706 | 0.585271 | 0.004715 | 0.55573 |
| *est1887 | 0.9706 | 0.9706 | 0.626369 | 0.008289 | 0.59886 | 0.9596 | 0.97059 | 0.626369 | 0.005380 | 0.64145 | 0.9611 | 0.97059 | 0.626369 | 0.00338 | 0.62213 | 0.9625 | | 0.64706 | 0.585271 | 0.008289 | 0.55610 |
| *ahb2182 | 0.9706 | 0.9722 | 0.629296 | 0.004066 | 0.59965 | 1.0000 | 1.00000 | 0.693147 | 0.003970 | 0.66259 | 0.9602 | 0.94737 | 0.588665 | 0.00535 | 0.62348 | 0.9636 | | 0.66667 | 0.579257 | 0.004066 | 0.56449 |
| AMB-00509364 | 0.9642 | 0.9630 | 0.613256 | 0.004183 | 0.59921 | 1.0000 | 1.00000 | 0.693147 | 0.003630 | 0.66053 | 0.9522 | 0.92857 | 0.561935 | 0.01718 | 0.62233 | 0.9576 | | 0.66667 | 0.565984 | 0.004183 | 0.56180 |
| est1597 | 0.9696 | 0.9688 | 0.623128 | 0.007866 | 0.59952 | 0.9578 | 0.96875 | 0.623128 | 0.008940 | 0.64062 | 0.9595 | 0.96875 | 0.623128 | 0.00306 | 0.62253 | 0.9609 | | 0.64583 | 0.582695 | 0.007866 | 0.55629 |
| ahb9636 | 0.9706 | 0.9722 | 0.629296 | 0.002814 | 0.60262 | 1.0000 | 1.00000 | 0.693147 | 0.007290 | 0.66295 | 0.9602 | 0.94737 | 0.588665 | 0.00450 | 0.62231 | 0.9636 | | 0.66667 | 0.579257 | 0.002814 | 0.56724 |
| ahb439 | 0.9633 | 0.9722 | 0.629296 | 0.005283 | 0.59989 | 1.0000 | 1.00000 | 0.693147 | 0.003310 | 0.66133 | 0.9459 | 0.94737 | 0.588665 | 0.00386 | 0.62279 | 0.9480 | | 0.66667 | 0.579257 | 0.005283 | 0.56282 |
| *ahb9479 | 0.9413 | 0.9444 | 0.584324 | 0.017858 | 0.59855 | 1.0000 | 1.00000 | 0.693147 | 0.011330 | 0.66135 | 0.9191 | 0.89474 | 0.519349 | 0.00000 | 0.62109 | 0.9298 | | 0.66667 | 0.545945 | 0.017858 | 0.56142 |
| ahb12228 | 0.9706 | 0.9706 | 0.626369 | 0.003711 | 0.59770 | 0.9596 | 0.97059 | 0.626369 | 0.004060 | 0.64183 | 0.9611 | 0.97059 | 0.626369 | 0.01299 | 0.62292 | 0.9625 | | 0.64706 | 0.585271 | 0.003711 | 0.55463 |
| AMB-00555384 | 0.9395 | 0.9428 | 0.563335 | 0.045297 | 0.59126 | 0.9139 | 0.91177 | 0.514510 | 0.025380 | 0.63539 | 0.9611 | 0.97059 | 0.626369 | 0.04157 | 0.62186 | 0.9248 | | 0.64706 | 0.524291 | 0.045297 | 0.54932 |
| ahb8411 | 0.9706 | 0.9722 | 0.629296 | 0.003641 | 0.60110 | 1.0000 | 1.00000 | 0.693147 | 0.001250 | 0.66338 | 0.9602 | 0.94737 | 0.588665 | 0.01109 | 0.62204 | 0.9636 | | 0.66667 | 0.579257 | 0.003641 | 0.56593 |
| *est486 | 0.9413 | 0.9444 | 0.584324 | 0.007336 | 0.59766 | 0.9150 | 0.88235 | 0.505105 | 0.010840 | 0.64107 | 1.0000 | 1.00000 | 0.693147 | 0.00382 | 0.65061 | 0.9308 | | 0.66667 | 0.539562 | 0.007336 | 0.56198 |
| ahb11909 | 1.0000 | 1.0000 | 0.693147 | 0.002443 | 0.65833 | 1.0000 | 1.00000 | 0.693147 | 0.003490 | 0.66417 | 1.0000 | 1.00000 | 0.693147 | 0.00175 | 0.65110 | 1.0000 | | 0.66667 | 0.636514 | 0.002443 | 0.64586 |
| *ahb282 | 0.9413 | 0.9444 | 0.584324 | 0.010082 | 0.59838 | 1.0000 | 1.00000 | 0.693147 | 0.004790 | 0.66312 | 0.9191 | 0.89474 | 0.519349 | 0.00345 | 0.61941 | 0.9298 | | 0.66667 | 0.545945 | 0.010082 | 0.56048 |
| ahb10720 | 0.9683 | 0.9706 | 0.626369 | 0.003721 | 0.60170 | 1.0000 | 1.00000 | 0.693147 | 0.003950 | 0.66180 | 0.9568 | 0.94444 | 0.584324 | 0.00432 | 0.62177 | 0.9604 | | 0.66667 | 0.577066 | 0.003721 | 0.56525 |
| *ahb6234 | 1.0000 | 1.0000 | 0.693147 | 0.002181 | 0.66089 | 1.0000 | 1.00000 | 0.693147 | 0.003230 | 0.66488 | 1.0000 | 1.00000 | 0.693147 | 0.00170 | 0.65142 | 1.0000 | | 0.66667 | 0.636514 | 0.002181 | 0.65048 |
| ahb7107 | 0.9411 | 0.9412 | 0.579557 | 0.008838 | 0.59708 | 0.9199 | 0.94118 | 0.579557 | 0.010420 | 0.63975 | 0.9228 | 0.94118 | 0.579557 | 0.01256 | 0.62164 | 0.9255 | | 0.62745 | 0.547476 | 0.008838 | 0.55426 |
| *est7035 | 0.9395 | 0.9428 | 0.563335 | 0.024248 | 0.58964 | 0.9596 | 0.97059 | 0.626369 | 0.011010 | 0.64253 | 0.9178 | 0.91796 | 0.523436 | 0.02343 | 0.61499 | 0.9246 | | 0.64706 | 0.528784 | 0.024248 | 0.54743 |
| est5253 | 0.8827 | 0.8889 | 0.512546 | 0.017730 | 0.59520 | 1.0000 | 1.00000 | 0.693147 | 0.022620 | 0.66186 | 0.8331 | 0.78947 | 0.413493 | 0.01248 | 0.61938 | 0.8714 | | 0.66667 | 0.502858 | 0.017730 | 0.56029 |
| ahb12436 | 1.0000 | 1.0000 | 0.693147 | 0.001941 | 0.65924 | 1.0000 | 1.00000 | 0.693147 | 0.001890 | 0.66431 | 1.0000 | 1.00000 | 0.693147 | 0.00277 | 0.65198 | 1.0000 | | 0.66667 | 0.636514 | 0.001941 | 0.65113 |
| AMB-01141593 | 0.9411 | 0.9412 | 0.579557 | 0.010032 | 0.59762 | 0.9199 | 0.94118 | 0.579557 | 0.011210 | 0.64055 | 0.9228 | 0.94118 | 0.579557 | 0.00704 | 0.62014 | 0.9255 | | 0.62745 | 0.547476 | 0.010032 | 0.55427 |
| AMB-01112431 | 0.9706 | 0.9706 | 0.626369 | 0.003914 | 0.59814 | 0.9596 | 0.97059 | 0.626369 | 0.004380 | 0.64206 | 0.9611 | 0.97059 | 0.626369 | 0.00414 | 0.62246 | 0.9625 | | 0.64706 | 0.585271 | 0.003914 | 0.55721 |
| AMB-00831952 | 0.9706 | 0.9722 | 0.629296 | 0.003385 | 0.60081 | 1.0000 | 1.00000 | 0.693147 | 0.001760 | 0.66371 | 0.9602 | 0.94737 | 0.588665 | 0.00544 | 0.62197 | 0.9636 | | 0.66667 | 0.579257 | 0.003385 | 0.56586 |
| est2362 | 0.9413 | 0.9444 | 0.584324 | 0.006873 | 0.59931 | 1.0000 | 1.00000 | 0.693147 | 0.004550 | 0.66337 | 0.9191 | 0.89474 | 0.519349 | 0.00295 | 0.62170 | 0.9298 | | 0.66667 | 0.545945 | 0.006873 | 0.56087 |
| *ahb10154 | 0.9706 | 0.9706 | 0.626369 | 0.002951 | 0.59830 | 0.9596 | 0.97059 | 0.626369 | 0.003090 | 0.64144 | 0.9611 | 0.97059 | 0.626369 | 0.01095 | 0.62207 | 0.9625 | | 0.64706 | 0.585271 | 0.002951 | 0.55653 |
| AMB-00905793 | 1.0000 | 1.0000 | 0.693147 | 0.002205 | 0.64770 | 1.0000 | 1.00000 | 0.693147 | 0.002020 | 0.66177 | 1.0000 | 1.00000 | 0.693147 | 0.00217 | 0.64703 | 1.0000 | | 0.66667 | 0.636514 | 0.002205 | 0.63977 |
| est8368 | 1.0000 | 1.0000 | 0.693147 | 0.002031 | 0.65163 | 1.0000 | 1.00000 | 0.693147 | 0.001840 | 0.65906 | 1.0000 | 1.00000 | 0.693147 | 0.00267 | 0.64687 | 1.0000 | | 0.66667 | 0.636514 | 0.002031 | 0.63867 |
| *ahb9535 | 0.9706 | 0.9722 | 0.629296 | 0.003047 | 0.60037 | 1.0000 | 1.00000 | 0.693147 | 0.001760 | 0.66394 | 0.9602 | 0.94737 | 0.588665 | 0.00475 | 0.62255 | 0.9636 | | 0.66667 | 0.579257 | 0.003047 | 0.56528 |
| AMB-00896332 | 0.9706 | 0.9706 | 0.626369 | 0.003188 | 0.59887 | 0.9596 | 0.97059 | 0.626369 | 0.002380 | 0.64278 | 0.9611 | 0.97059 | 0.626369 | 0.00617 | 0.62219 | 0.9625 | | 0.64706 | 0.585271 | 0.003188 | 0.55591 |
| est7014 | 0.9706 | 0.9706 | 0.626369 | 0.002686 | 0.59943 | 0.9596 | 0.97059 | 0.626369 | 0.002470 | 0.64200 | 0.9611 | 0.97059 | 0.626369 | 0.00974 | 0.62224 | 0.9625 | | 0.64706 | 0.585271 | 0.002686 | 0.55599 |
| ahb6818 | 0.9120 | 0.9167 | 0.546253 | 0.009311 | 0.59622 | 0.9150 | 0.88235 | 0.505105 | 0.014520 | 0.64028 | 0.9602 | 0.94737 | 0.588665 | 0.01562 | 0.62288 | 0.8912 | | 0.63158 | 0.479322 | 0.009311 | 0.55044 |
| AMB-00228327 | 0.9413 | 0.9444 | 0.584324 | 0.005876 | 0.59741 | 1.0000 | 1.00000 | 0.693147 | 0.007490 | 0.66271 | 0.9191 | 0.89474 | 0.519349 | 0.00260 | 0.62088 | 0.9298 | | 0.66667 | 0.545945 | 0.005876 | 0.56058 |
| AMB-00995347 | 0.9387 | 0.9444 | 0.584324 | 0.005996 | 0.59830 | 1.0000 | 1.00000 | 0.693147 | 0.002410 | 0.66151 | 0.9145 | 0.89474 | 0.519349 | 0.00930 | 0.62013 | 0.9254 | | 0.66667 | 0.545945 | 0.005996 | 0.56107 |
| *est8209 | 0.9413 | 0.9444 | 0.584324 | 0.009127 | 0.59731 | 0.9584 | 0.94118 | 0.579557 | 0.002270 | 0.64089 | 0.9602 | 0.94737 | 0.588665 | 0.01050 | 0.62272 | 0.9258 | | 0.63158 | 0.515910 | 0.009127 | 0.55124 |
| ahb12014 | 1.0000 | 1.0000 | 0.693147 | 0.001784 | 0.66362 | 1.0000 | 1.00000 | 0.693147 | 0.001870 | 0.66164 | 1.0000 | 1.00000 | 0.693147 | 0.00177 | 0.65309 | 1.0000 | | 0.66667 | 0.636514 | 0.001784 | 0.65023 |
| AMB-01040171 | 0.9706 | 0.9706 | 0.626369 | 0.002739 | 0.59819 | 0.9596 | 0.97059 | 0.626369 | 0.002970 | 0.64219 | 0.9611 | 0.97059 | 0.626369 | 0.00784 | 0.62213 | 0.9625 | | 0.64706 | 0.585271 | 0.002739 | 0.55534 |
| AMB-00967423 | 0.9688 | 0.9706 | 0.626369 | 0.003411 | 0.60151 | 0.9567 | 0.94118 | 0.579557 | 0.002710 | 0.64166 | 1.0000 | 1.00000 | 0.693147 | 0.00412 | 0.65026 | 0.9614 | | 0.66667 | 0.574676 | 0.003411 | 0.56411 |
| *est6877 | 0.9692 | 0.9688 | 0.623128 | 0.003065 | 0.59745 | 0.9570 | 0.96875 | 0.623128 | 0.004310 | 0.64131 | 0.9595 | 0.96875 | 0.623128 | 0.01103 | 0.62168 | 0.9605 | | 0.64583 | 0.582695 | 0.003065 | 0.55547 |
| ahb5519 | 0.8827 | 0.8889 | 0.512546 | 0.010103 | 0.59632 | 0.8227 | 0.76471 | 0.392407 | 0.012120 | 0.63821 | 1.0000 | 1.00000 | 0.693147 | 0.01576 | 0.65067 | 0.8757 | | 0.66667 | 0.495630 | 0.010103 | 0.56038 |
| *est5252 | 0.9706 | 0.9706 | 0.626369 | 0.003076 | 0.60004 | 0.9596 | 0.97059 | 0.626369 | 0.001760 | 0.64294 | 0.9611 | 0.97059 | 0.626369 | 0.00820 | 0.62134 | 0.9625 | | 0.64706 | 0.585271 | 0.003076 | 0.55621 |
| AMB-00524451 | 0.9232 | 0.9282 | 0.538550 | 0.105968 | 0.59098 | 0.9540 | 0.96667 | 0.619519 | 0.170690 | 0.64126 | 0.8878 | 0.87576 | 0.466098 | 0.20232 | 0.61367 | 0.9093 | | 0.64445 | 0.498736 | 0.105968 | 0.54842 |
| est7637 | 0.9120 | 0.9167 | 0.546253 | 0.011665 | 0.59696 | 0.9150 | 0.88235 | 0.505105 | 0.010760 | 0.63943 | 0.9602 | 0.94737 | 0.588665 | 0.00667 | 0.62379 | 0.8912 | | 0.63158 | 0.479322 | 0.011665 | 0.55075 |
| ahb2169 | 0.9706 | 0.9722 | 0.629296 | 0.002701 | 0.60115 | 1.0000 | 1.00000 | 0.693147 | 0.002280 | 0.66277 | 0.9602 | 0.94737 | 0.588665 | 0.00288 | 0.62326 | 0.9636 | | 0.66667 | 0.579257 | 0.002701 | 0.56563 |
| *AMB-00823001 | 0.9706 | 0.9722 | 0.629296 | 0.003065 | 0.59935 | 0.9584 | 0.94118 | 0.579557 | 0.003580 | 0.64253 | 1.0000 | 1.00000 | 0.693147 | 0.00170 | 0.65140 | 0.9638 | | 0.66667 | 0.574676 | 0.003065 | 0.56569 |
| AMB-00528196 | 1.0000 | 1.0000 | 0.693147 | 0.001771 | 0.65728 | 1.0000 | 1.00000 | 0.693147 | 0.001760 | 0.66483 | 1.0000 | 1.00000 | 0.693147 | 0.00170 | 0.65451 | 1.0000 | | 0.66667 | 0.636514 | 0.001771 | 0.64789 |
| *ahb3116 | 1.0000 | 1.0000 | 0.693147 | 0.001771 | 0.65956 | 1.0000 | 1.00000 | 0.693147 | 0.001760 | 0.66358 | 1.0000 | 1.00000 | 0.693147 | 0.00170 | 0.65334 | 1.0000 | | 0.66667 | 0.636514 | 0.001771 | 0.65020 |
| *AMB-00616336 | 0.9395 | 0.9428 | 0.563335 | 0.010948 | 0.59163 | 0.9596 | 0.97059 | 0.626369 | 0.009970 | 0.64225 | 0.9178 | 0.91796 | 0.523436 | 0.00619 | 0.61466 | 0.9246 | | 0.64706 | 0.528784 | 0.010948 | 0.54790 |
| ahb10181 | 0.9706 | 0.9706 | 0.626369 | 0.003667 | 0.59845 | 0.9596 | 0.97059 | 0.626369 | 0.001760 | 0.64148 | 0.9611 | 0.97059 | 0.626369 | 0.00639 | 0.62118 | 0.9625 | | 0.64706 | 0.585271 | 0.003667 | 0.55684 |
| AMB-00032042 | 0.9120 | 0.9167 | 0.546253 | 0.005434 | 0.59710 | 1.0000 | 1.00000 | 0.693147 | 0.007210 | 0.66276 | 0.8768 | 0.84211 | 0.462548 | 0.00760 | 0.61985 | 0.8990 | | 0.66667 | 0.521521 | 0.005434 | 0.56011 |
| AMB-00713787 | 1.0000 | 1.0000 | 0.693147 | 0.001771 | 0.66252 | 1.0000 | 1.00000 | 0.693147 | 0.001760 | 0.66468 | 1.0000 | 1.00000 | 0.693147 | 0.00170 | 0.65104 | 1.0000 | | 0.66667 | 0.636514 | 0.001771 | 0.64714 |
| AMB-00925896 | 1.0000 | 1.0000 | 0.693147 | 0.001771 | 0.66326 | 1.0000 | 1.00000 | 0.693147 | 0.001760 | 0.66294 | 1.0000 | 1.00000 | 0.693147 | 0.00170 | 0.65137 | 1.0000 | | 0.66667 | 0.636514 | 0.001771 | 0.65028 |
| *est11364 | 1.0000 | 1.0000 | 0.693147 | 0.001771 | 0.66075 | 1.0000 | 1.00000 | 0.693147 | 0.001760 | 0.66256 | 1.0000 | 1.00000 | 0.693147 | 0.00170 | 0.65256 | 1.0000 | | 0.66667 | 0.636514 | 0.001771 | 0.65188 |
| ahb10805 | 1.0000 | 1.0000 | 0.693147 | 0.001771 | 0.65722 | 1.0000 | 1.00000 | 0.693147 | 0.001760 | 0.66408 | 1.0000 | 1.00000 | 0.693147 | 0.00170 | 0.65087 | 1.0000 | | 0.66667 | 0.636514 | 0.001771 | 0.65136 |
| AMB-00879167 | 0.9706 | 0.9706 | 0.626369 | 0.001880 | 0.59999 | 0.9596 | 0.97059 | 0.626369 | 0.002820 | 0.64170 | 0.9611 | 0.97059 | 0.626369 | 0.01086 | 0.62190 | 0.9625 | | 0.64706 | 0.585271 | 0.001880 | 0.55673 |
| *ahb11154 | 1.0000 | 1.0000 | 0.693147 | 0.001771 | 0.65934 | 1.0000 | 1.00000 | 0.693147 | 0.001760 | 0.66535 | 1.0000 | 1.00000 | 0.693147 | 0.00170 | 0.64988 | 1.0000 | | 0.66667 | 0.636514 | 0.001771 | 0.64690 |
| *est9997 | 1.0000 | 1.0000 | 0.693147 | 0.001771 | 0.66293 | 1.0000 | 1.00000 | 0.693147 | 0.001760 | 0.66395 | 1.0000 | 1.00000 | 0.693147 | 0.00170 | 0.64917 | 1.0000 | | 0.66667 | 0.636514 | 0.001771 | 0.64534 |
| *est9248 | 1.0000 | 1.0000 | 0.693147 | 0.001771 | 0.65873 | 1.0000 | 1.00000 | 0.693147 | 0.001760 | 0.66273 | 1.0000 | 1.00000 | 0.693147 | 0.00170 | 0.65199 | 1.0000 | | 0.66667 | 0.636514 | 0.001771 | 0.64544 |
| *ahb12013 | 1.0000 | 1.0000 | 0.693147 | 0.001771 | 0.66177 | 1.0000 | 1.00000 | 0.693147 | 0.001760 | 0.66209 | 1.0000 | 1.00000 | 0.693147 | 0.00170 | 0.65107 | 1.0000 | | 0.66667 | 0.636514 | 0.001771 | 0.64747 |
| est6550 | 1.0000 | 1.0000 | 0.693147 | 0.001771 | 0.66452 | 1.0000 | 1.00000 | 0.693147 | 0.001760 | 0.66188 | 1.0000 | 1.00000 | 0.693147 | 0.00170 | 0.65058 | 1.0000 | | 0.66667 | 0.636514 | 0.001771 | 0.64899 |
| AMB-00166085 | 0.9688 | 0.9706 | 0.626369 | 0.002670 | 0.60076 | 1.0000 | 1.00000 | 0.693147 | 0.002310 | 0.66128 | 0.9567 | 0.94118 | 0.579557 | 0.00412 | 0.62242 | 0.9614 | | 0.66667 | 0.574676 | 0.002670 | 0.56345 |
| est6107 | 0.9413 | 0.9444 | 0.584324 | 0.004770 | 0.59841 | 0.9584 | 0.94118 | 0.579557 | 0.004450 | 0.64136 | 0.9602 | 0.94737 | 0.588665 | 0.00768 | 0.62324 | 0.9258 | | 0.63158 | 0.515910 | 0.004770 | 0.55134 |
| ahb8015 | 0.9706 | 0.9722 | 0.629296 | 0.002702 | 0.60111 | 0.9584 | 0.94118 | 0.579557 | 0.003600 | 0.64131 | 1.0000 | 1.00000 | 0.693147 | 0.00189 | 0.65297 | 0.9638 | | 0.66667 | 0.574676 | 0.002702 | 0.56577 |
| *est9255 | 1.0000 | 1.0000 | 0.693147 | 0.001771 | 0.66070 | 1.0000 | 1.00000 | 0.693147 | 0.001760 | 0.66174 | 1.0000 | 1.00000 | 0.693147 | 0.00170 | 0.65030 | 1.0000 | | 0.66667 | 0.636514 | 0.001771 | 0.65114 |
| ahb10689 | 0.9706 | 0.9722 | 0.629296 | 0.002928 | 0.60090 | 0.9584 | 0.94118 | 0.579557 | 0.003480 | 0.64186 | 1.0000 | 1.00000 | 0.693147 | 0.00170 | 0.65216 | 0.9638 | | 0.66667 | 0.574676 | 0.002928 | 0.56511 |
| est9729 | 0.9706 | 0.9722 | 0.629296 | 0.002928 | 0.59938 | 0.9584 | 0.94118 | 0.579557 | 0.003480 | 0.64202 | 1.0000 | 1.00000 | 0.693147 | 0.00170 | 0.64864 | 0.9638 | | 0.66667 | 0.574676 | 0.002928 | 0.56660 |
| ahb6757 | 0.9706 | 0.9722 | 0.629296 | 0.002481 | 0.60184 | 0.9584 | 0.94118 | 0.579557 | 0.002350 | 0.64211 | 1.0000 | 1.00000 | 0.693147 | 0.00260 | 0.65128 | 0.9638 | | 0.66667 | 0.574676 | 0.002481 | 0.56518 |
| est11186 | 0.9706 | 0.9722 | 0.629296 | 0.002417 | 0.60076 | 1.0000 | 1.00000 | 0.693147 | 0.001760 | 0.66131 | 0.9602 | 0.94737 | 0.588665 | 0.00446 | 0.62166 | 0.9636 | | 0.66667 | 0.579257 | 0.002417 | 0.56500 |
| *est4550 | 0.9386 | 0.9419 | 0.561782 | 0.017469 | 0.59052 | 0.9587 | 0.96970 | 0.624791 | 0.014420 | 0.64110 | 0.9161 | 0.91707 | 0.521905 | 0.00344 | 0.61544 | 0.9230 | | 0.64647 | 0.527554 | 0.017469 | 0.54861 |
| *est2248 | 0.9706 | 0.9706 | 0.626369 | 0.003020 | 0.59844 | 0.9596 | 0.97059 | 0.626369 | 0.002040 | 0.64113 | 0.9611 | 0.97059 | 0.626369 | 0.00464 | 0.62234 | 0.9625 | | 0.64706 | 0.585271 | 0.003020 | 0.55576 |
| ahb2731 | 0.9413 | 0.9444 | 0.584324 | 0.002988 | 0.59988 | 1.0000 | 1.00000 | 0.693147 | 0.002760 | 0.66239 | 0.9191 | 0.89474 | 0.519349 | 0.00609 | 0.62171 | 0.9298 | | 0.66667 | 0.545945 | 0.002988 | 0.56121 |
| ahb1931 | 0.9413 | 0.9444 | 0.584324 | 0.003264 | 0.59940 | 0.9150 | 0.88235 | 0.505105 | 0.004180 | 0.64010 | 1.0000 | 1.00000 | 0.693147 | 0.00518 | 0.65058 | 0.9308 | | 0.66667 | 0.539562 | 0.003264 | 0.56294 |
| ahb7936 | 0.9120 | 0.9167 | 0.546253 | 0.016318 | 0.59687 | 0.9150 | 0.88235 | 0.505105 | 0.008870 | 0.63959 | 0.9602 | 0.94737 | 0.588665 | 0.00311 | 0.62414 | 0.8912 | | 0.63158 | 0.479322 | 0.016318 | 0.55012 |
| *est2365 | 0.9706 | 0.9722 | 0.629296 | 0.002226 | 0.60143 | 0.9584 | 0.94118 | 0.579557 | 0.001760 | 0.64116 | 1.0000 | 1.00000 | 0.693147 | 0.00454 | 0.64882 | 0.9638 | | 0.66667 | 0.574676 | 0.002226 | 0.56561 |
| ahb9130 | 0.9706 | 0.9722 | 0.629296 | 0.002878 | 0.60157 | 0.9584 | 0.94118 | 0.579557 | 0.002870 | 0.64086 | 1.0000 | 1.00000 | 0.693147 | 0.00170 | 0.64940 | 0.9638 | | 0.66667 | 0.574676 | 0.002878 | 0.56647 |
| AMB-00803815 | 0.9120 | 0.9167 | 0.546253 | 0.007972 | 0.59798 | 0.9584 | 0.94118 | 0.579557 | 0.013860 | 0.64229 | 0.9191 | 0.89474 | 0.519349 | 0.00476 | 0.62064 | 0.8904 | | 0.62745 | 0.481120 | 0.007972 | 0.54929 |
| est1736 | 0.9413 | 0.9444 | 0.584324 | 0.004241 | 0.59908 | 0.9584 | 0.94118 | 0.579557 | 0.004830 | 0.64210 | 0.9602 | 0.94737 | 0.588665 | 0.00430 | 0.62282 | 0.9258 | | 0.63158 | 0.515910 | 0.004241 | 0.55248 |
| *est813 | 0.9413 | 0.9444 | 0.584324 | 0.005334 | 0.59886 | 0.9584 | 0.94118 | 0.579557 | 0.005520 | 0.64188 | 0.9602 | 0.94737 | 0.588665 | 0.00238 | 0.62394 | 0.9258 | | 0.63158 | 0.515910 | 0.005334 | 0.55146 |
| *est7615 | 0.8827 | 0.8889 | 0.512546 | 0.016371 | 0.59726 | 0.8698 | 0.82353 | 0.444495 | 0.013140 | 0.63832 | 0.9602 | 0.94737 | 0.588665 | 0.00916 | 0.62281 | 0.8600 | | 0.63158 | 0.452686 | 0.016371 | 0.54971 |
| *est8667 | 0.9413 | 0.9444 | 0.584324 | 0.004377 | 0.59945 | 0.9584 | 0.94118 | 0.579557 | 0.004330 | 0.64124 | 0.9602 | 0.94737 | 0.588665 | 0.00561 | 0.62213 | 0.9258 | | 0.63158 | 0.515910 | 0.004377 | 0.55223 |
| est9223 | 0.9120 | 0.9167 | 0.546253 | 0.008443 | 0.59713 | 0.9150 | 0.88235 | 0.505105 | 0.004240 | 0.64061 | 0.9602 | 0.94737 | 0.588665 | 0.01108 | 0.62250 | 0.8912 | | 0.63158 | 0.479322 | 0.008443 | 0.55014 |
| *ahb636 | 0.9692 | 0.9688 | 0.623128 | 0.003193 | 0.59740 | 0.9570 | 0.96875 | 0.623128 | 0.001950 | 0.64199 | 0.9595 | 0.96875 | 0.623128 | 0.00513 | 0.62193 | 0.9605 | | 0.64583 | 0.582695 | 0.003193 | 0.55574 |
| *ahb2175 | 0.9706 | 0.9722 | 0.629296 | 0.001946 | 0.60093 | 1.0000 | 1.00000 | 0.693147 | 0.001760 | 0.66261 | 0.9602 | 0.94737 | 0.588665 | 0.00302 | 0.62350 | 0.9636 | | 0.66667 | 0.579257 | 0.001946 | 0.56541 |
| *est2039 | 0.9387 | 0.9444 | 0.584324 | 0.003824 | 0.59861 | 0.9558 | 0.94118 | 0.579557 | 0.003530 | 0.64126 | 0.9578 | 0.94737 | 0.588665 | 0.01457 | 0.62216 | 0.9211 | | 0.63158 | 0.515910 | 0.003824 | 0.55189 |
| est11097 | 0.9706 | 0.9722 | 0.629296 | 0.001812 | 0.60166 | 1.0000 | 1.00000 | 0.693147 | 0.001760 | 0.66338 | 0.9602 | 0.94737 | 0.588665 | 0.00338 | 0.62237 | 0.9636 | | 0.66667 | 0.579257 | 0.001812 | 0.56667 |
| ahb7276 | 1.0000 | 1.0000 | 0.693147 | 0.000729 | 0.65838 | 1.0000 | 1.00000 | 0.693147 | 0.000760 | 0.66325 | 1.0000 | 1.00000 | 0.693147 | 0.00157 | 0.65092 | 1.0000 | | 0.66667 | 0.636514 | 0.000729 | 0.64680 |
| *est4464 | 0.9115 | 0.9118 | 0.540031 | 0.014105 | 0.59655 | 0.8810 | 0.91176 | 0.540031 | 0.013820 | 0.63974 | 0.8852 | 0.91176 | 0.540031 | 0.00599 | 0.62047 | 0.8890 | | 0.60784 | 0.514672 | 0.014105 | 0.55183 |
| AMB-00466276 | 0.8827 | 0.8889 | 0.512546 | 0.016419 | 0.59688 | 0.8227 | 0.76471 | 0.392407 | 0.002870 | 0.63892 | 1.0000 | 1.00000 | 0.693147 | 0.00706 | 0.65152 | 0.8757 | | 0.66667 | 0.495630 | 0.016419 | 0.56151 |
| AMB-01113590 | 0.9706 | 0.9722 | 0.629296 | 0.002037 | 0.60151 | 1.0000 | 1.00000 | 0.693147 | 0.001760 | 0.66268 | 0.9602 | 0.94737 | 0.588665 | 0.00238 | 0.62290 | 0.9636 | | 0.66667 | 0.579257 | 0.002037 | 0.56509 |
| *est8869 | 0.9413 | 0.9444 | 0.584324 | 0.003395 | 0.59959 | 1.0000 | 1.00000 | 0.693147 | 0.006360 | 0.66223 | 0.9191 | 0.89474 | 0.519349 | 0.00170 | 0.62035 | 0.9298 | | 0.66667 | 0.545945 | 0.003395 | 0.56181 |
| est9901 | 0.9357 | 0.9412 | 0.579557 | 0.004563 | 0.59828 | 0.9537 | 0.93750 | 0.574297 | 0.004830 | 0.64142 | 0.9559 | 0.94444 | 0.584324 | 0.00559 | 0.62310 | 0.9177 | | 0.62963 | 0.510919 | 0.004563 | 0.55140 |
| est7044 | 0.9413 | 0.9444 | 0.584324 | 0.003321 | 0.59854 | 1.0000 | 1.00000 | 0.693147 | 0.002630 | 0.66229 | 0.9191 | 0.89474 | 0.519349 | 0.00492 | 0.61969 | 0.9298 | | 0.66667 | 0.545945 | 0.003321 | 0.56207 |
| AMB-00733344 | 0.9413 | 0.9444 | 0.584324 | 0.003394 | 0.59827 | 1.0000 | 1.00000 | 0.693147 | 0.001760 | 0.66460 | 0.9191 | 0.89474 | 0.519349 | 0.00528 | 0.62101 | 0.9298 | | 0.66667 | 0.545945 | 0.003394 | 0.56153 |
| AMB-01152391 | 0.9120 | 0.9167 | 0.546253 | 0.009108 | 0.59731 | 0.9150 | 0.88235 | 0.505105 | 0.008920 | 0.64052 | 0.9602 | 0.94737 | 0.588665 | 0.00273 | 0.62393 | 0.8912 | | 0.63158 | 0.479322 | 0.009108 | 0.55090 |
| AMB-00981727 | 0.9706 | 0.9722 | 0.629296 | 0.001936 | 0.60051 | 1.0000 | 1.00000 | 0.693147 | 0.001760 | 0.66264 | 0.9602 | 0.94737 | 0.588665 | 0.00249 | 0.62227 | 0.9636 | | 0.66667 | 0.579257 | 0.001936 | 0.56427 |
| ahb12331 | 0.9706 | 0.9706 | 0.626369 | 0.001981 | 0.59853 | 0.9596 | 0.97059 | 0.626369 | 0.001980 | 0.64122 | 0.9611 | 0.97059 | 0.626369 | 0.00446 | 0.62275 | 0.9625 | | 0.64706 | 0.585271 | 0.001981 | 0.55657 |
| *est11407 | 0.9404 | 0.9444 | 0.584324 | 0.004542 | 0.59854 | 0.9134 | 0.88235 | 0.505105 | 0.004120 | 0.63907 | 1.0000 | 1.00000 | 0.693147 | 0.00190 | 0.65101 | 0.9295 | | 0.66667 | 0.539562 | 0.004542 | 0.56256 |
| ahb8451 | 1.0000 | 1.0000 | 0.693147 | 0.000554 | 0.66109 | 1.0000 | 1.00000 | 0.693147 | 0.000440 | 0.66262 | 1.0000 | 1.00000 | 0.693147 | 0.00065 | 0.64941 | 1.0000 | | 0.66667 | 0.636514 | 0.000554 | 0.65219 |
| *AMB-01049929 | 0.9706 | 0.9722 | 0.629296 | 0.001872 | 0.60039 | 1.0000 | 1.00000 | 0.693147 | 0.001760 | 0.66279 | 0.9602 | 0.94737 | 0.588665 | 0.00260 | 0.62141 | 0.9636 | | 0.66667 | 0.579257 | 0.001872 | 0.56698 |
| est5975 | 1.0000 | 1.0000 | 0.693147 | 0.000443 | 0.66316 | 1.0000 | 1.00000 | 0.693147 | 0.000440 | 0.66399 | 1.0000 | 1.00000 | 0.693147 | 0.00043 | 0.65009 | 1.0000 | | 0.66667 | 0.636514 | 0.000443 | 0.64894 |
| AMB-01002419 | 1.0000 | 1.0000 | 0.693147 | 0.000471 | 0.65738 | 1.0000 | 1.00000 | 0.693147 | 0.000470 | 0.66470 | 1.0000 | 1.00000 | 0.693147 | 0.00048 | 0.64701 | 1.0000 | | 0.66667 | 0.636514 | 0.000471 | 0.64436 |
| *est3557 | 0.9706 | 0.9706 | 0.626369 | 0.002166 | 0.59855 | 0.9596 | 0.97059 | 0.626369 | 0.002260 | 0.64203 | 0.9611 | 0.97059 | 0.626369 | 0.00265 | 0.62220 | 0.9625 | | 0.64706 | 0.585271 | 0.002166 | 0.55602 |
| ahb1595 | 0.9413 | 0.9444 | 0.584324 | 0.004165 | 0.59842 | 0.9584 | 0.94118 | 0.579557 | 0.005520 | 0.64159 | 0.9602 | 0.94737 | 0.588665 | 0.00309 | 0.62213 | 0.9258 | | 0.63158 | 0.515910 | 0.004165 | 0.55248 |
| ahb11961 | 0.9120 | 0.9167 | 0.546253 | 0.006836 | 0.59719 | 0.9150 | 0.88235 | 0.505105 | 0.009560 | 0.63932 | 0.9602 | 0.94737 | 0.588665 | 0.00407 | 0.62333 | 0.8912 | | 0.63158 | 0.479322 | 0.006836 | 0.55111 |
| *ahb1616 | 0.9706 | 0.9706 | 0.626369 | 0.002039 | 0.59833 | 0.9596 | 0.97059 | 0.626369 | 0.001880 | 0.64207 | 0.9611 | 0.97059 | 0.626369 | 0.00315 | 0.62267 | 0.9625 | | 0.64706 | 0.585271 | 0.002039 | 0.55672 |
| AMB-00716174 | 0.9395 | 0.9429 | 0.581997 | 0.003596 | 0.59902 | 0.9565 | 0.93750 | 0.574297 | 0.004820 | 0.64195 | 0.9594 | 0.94737 | 0.588665 | 0.00424 | 0.62295 | 0.9236 | | 0.63158 | 0.513199 | 0.003596 | 0.55100 |
| est9803 | 0.9706 | 0.9706 | 0.626369 | 0.002349 | 0.59836 | 0.9596 | 0.97059 | 0.626369 | 0.002370 | 0.64112 | 0.9611 | 0.97059 | 0.626369 | 0.00309 | 0.62195 | 0.9625 | | 0.64706 | 0.585271 | 0.002349 | 0.55543 |
| ahb7415 | 0.9411 | 0.9412 | 0.579557 | 0.003737 | 0.59791 | 0.9199 | 0.94118 | 0.579557 | 0.003280 | 0.63997 | 0.9228 | 0.94118 | 0.579557 | 0.01338 | 0.62127 | 0.9255 | | 0.62745 | 0.547476 | 0.003737 | 0.55495 |
| AMB-00862048 | 0.9120 | 0.9167 | 0.546253 | 0.004291 | 0.59784 | 1.0000 | 1.00000 | 0.693147 | 0.005060 | 0.66299 | 0.8768 | 0.84211 | 0.462548 | 0.00372 | 0.62007 | 0.8990 | | 0.66667 | 0.521521 | 0.004291 | 0.56130 |
| ahb11416 | 1.0000 | 1.0000 | 0.693147 | 0.000175 | 0.66129 | 1.0000 | 1.00000 | 0.693147 | 0.000000 | 0.66350 | 1.0000 | 1.00000 | 0.693147 | 0.00103 | 0.64924 | 1.0000 | | 0.66667 | 0.636514 | 0.000175 | 0.65036 |
| ahb9901 | 0.9706 | 0.9722 | 0.629296 | 0.001746 | 0.60079 | 1.0000 | 1.00000 | 0.693147 | 0.001710 | 0.66201 | 0.9602 | 0.94737 | 0.588665 | 0.00175 | 0.62318 | 0.9636 | | 0.66667 | 0.579257 | 0.001746 | 0.56442 |
| AMB-01032172 | 0.9383 | 0.9375 | 0.574297 | 0.004864 | 0.59671 | 0.9148 | 0.93750 | 0.574297 | 0.006300 | 0.64013 | 0.9196 | 0.93750 | 0.574297 | 0.00557 | 0.62027 | 0.9216 | | 0.62500 | 0.543156 | 0.004864 | 0.55292 |
| *est8339 | 0.9029 | 0.9032 | 0.529489 | 0.011675 | 0.59669 | 0.9516 | 0.91667 | 0.546253 | 0.011920 | 0.64135 | 0.9176 | 0.89474 | 0.519349 | 0.00256 | 0.62164 | 0.8819 | | 0.61111 | 0.463663 | 0.011675 | 0.54937 |
| *est8308 | 0.8827 | 0.8889 | 0.512546 | 0.011955 | 0.59550 | 0.9150 | 0.88235 | 0.505105 | 0.010210 | 0.63912 | 0.9191 | 0.89474 | 0.519349 | 0.01641 | 0.62127 | 0.8540 | | 0.59649 | 0.443085 | 0.011955 | 0.55000 |
| AMB-00501634 | 0.9702 | 0.9714 | 0.627869 | 0.001874 | 0.60010 | 0.9574 | 0.93750 | 0.574297 | 0.001850 | 0.64058 | 1.0000 | 1.00000 | 0.693147 | 0.00213 | 0.64850 | 0.9636 | | 0.66667 | 0.572057 | 0.001874 | 0.56579 |
| *est5127 | 0.9660 | 0.9655 | 0.617555 | 0.002575 | 0.59823 | 0.9539 | 0.96552 | 0.617555 | 0.003080 | 0.64002 | 0.9539 | 0.96552 | 0.617555 | 0.00379 | 0.62089 | 0.9563 | | 0.64368 | 0.578250 | 0.002575 | 0.55596 |
| ahb10610 | 0.9413 | 0.9444 | 0.584324 | 0.002741 | 0.59853 | 0.9584 | 0.94118 | 0.579557 | 0.003530 | 0.64211 | 0.9602 | 0.94737 | 0.588665 | 0.00449 | 0.62365 | 0.9258 | | 0.63158 | 0.515910 | 0.002741 | 0.55109 |
| est6839 | 0.9120 | 0.9167 | 0.546253 | 0.004541 | 0.59693 | 0.8698 | 0.82353 | 0.444495 | 0.004640 | 0.63966 | 1.0000 | 1.00000 | 0.693147 | 0.00365 | 0.65074 | 0.9014 | | 0.66667 | 0.514370 | 0.004541 | 0.56201 |
| *ahb1523 | 0.8827 | 0.8889 | 0.512546 | 0.006473 | 0.59622 | 1.0000 | 1.00000 | 0.693147 | 0.002360 | 0.66246 | 0.8331 | 0.78947 | 0.413493 | 0.01056 | 0.61970 | 0.8714 | | 0.66667 | 0.502858 | 0.006473 | 0.56044 |
| ahb11330 | 0.8535 | 0.8611 | 0.482000 | 0.014800 | 0.59523 | 0.8227 | 0.76471 | 0.392407 | 0.016850 | 0.63844 | 0.9602 | 0.94737 | 0.588665 | 0.01658 | 0.62236 | 0.8326 | | 0.63158 | 0.432524 | 0.014800 | 0.54854 |
| AMB-01059470 | 0.9120 | 0.9167 | 0.546253 | 0.002494 | 0.59707 | 0.8698 | 0.82353 | 0.444495 | 0.007330 | 0.63937 | 1.0000 | 1.00000 | 0.693147 | 0.00731 | 0.65099 | 0.9014 | | 0.66667 | 0.514370 | 0.002494 | 0.56153 |
| ahb8642 | 0.9706 | 0.9706 | 0.626369 | 0.001500 | 0.59867 | 0.9596 | 0.97059 | 0.626369 | 0.000000 | 0.64156 | 0.9611 | 0.97059 | 0.626369 | 0.00653 | 0.62236 | 0.9625 | | 0.64706 | 0.585271 | 0.001500 | 0.55796 |
| est9038 | 0.9706 | 0.9706 | 0.626369 | 0.001885 | 0.59844 | 0.9596 | 0.97059 | 0.626369 | 0.001890 | 0.64186 | 0.9611 | 0.97059 | 0.626369 | 0.00191 | 0.62262 | 0.9625 | | 0.64706 | 0.585271 | 0.001885 | 0.55573 |
| ahb12155 | 0.9413 | 0.9444 | 0.584324 | 0.003122 | 0.59837 | 0.9150 | 0.88235 | 0.505105 | 0.002390 | 0.64018 | 1.0000 | 1.00000 | 0.693147 | 0.00247 | 0.65154 | 0.9308 | | 0.66667 | 0.539562 | 0.003122 | 0.56323 |
| *AMB-00834673 | 1.0000 | 1.0000 | 0.693147 | 0.000000 | 0.66254 | 1.0000 | 1.00000 | 0.693147 | 0.000000 | 0.66304 | 1.0000 | 1.00000 | 0.693147 | 0.00000 | 0.65028 | 1.0000 | | 0.66667 | 0.636514 | 0.000000 | 0.64827 |
| ahb10298 | 0.9706 | 0.9722 | 0.629296 | 0.001771 | 0.60120 | 0.9584 | 0.94118 | 0.579557 | 0.001760 | 0.64159 | 1.0000 | 1.00000 | 0.693147 | 0.00170 | 0.64947 | 0.9638 | | 0.66667 | 0.574676 | 0.001771 | 0.56582 |
| ahb8307 | 0.9085 | 0.9150 | 0.519180 | 0.018387 | 0.58986 | 0.9596 | 0.97059 | 0.626369 | 0.019120 | 0.64079 | 0.8730 | 0.86533 | 0.455674 | 0.00525 | 0.61235 | 0.8895 | | 0.64706 | 0.496223 | 0.018387 | 0.54743 |
| AMB-00398036 | 0.9413 | 0.9444 | 0.584324 | 0.003494 | 0.59787 | 0.9150 | 0.88235 | 0.505105 | 0.003090 | 0.64004 | 1.0000 | 1.00000 | 0.693147 | 0.00170 | 0.65121 | 0.9308 | | 0.66667 | 0.539562 | 0.003494 | 0.56404 |
| ahb10918 | 0.9706 | 0.9706 | 0.626369 | 0.001992 | 0.59879 | 0.9596 | 0.97059 | 0.626369 | 0.002040 | 0.64129 | 0.9611 | 0.97059 | 0.626369 | 0.00208 | 0.62087 | 0.9625 | | 0.64706 | 0.585271 | 0.001992 | 0.55650 |
| ahb2458 | 0.9706 | 0.9706 | 0.626369 | 0.002572 | 0.59879 | 0.9596 | 0.97059 | 0.626369 | 0.000460 | 0.64321 | 0.9611 | 0.97059 | 0.626369 | 0.00131 | 0.62138 | 0.9625 | | 0.64706 | 0.585271 | 0.002572 | 0.55526 |
| ahb4848 | 0.9706 | 0.9706 | 0.626369 | 0.001818 | 0.59820 | 0.9596 | 0.97059 | 0.626369 | 0.001880 | 0.64116 | 0.9611 | 0.97059 | 0.626369 | 0.00188 | 0.62299 | 0.9625 | | 0.64706 | 0.585271 | 0.001818 | 0.55686 |
| ahb1068 | 0.9701 | 0.9697 | 0.624791 | 0.001783 | 0.59935 | 0.9587 | 0.96970 | 0.624791 | 0.001840 | 0.64172 | 0.9603 | 0.96970 | 0.624791 | 0.00188 | 0.62263 | 0.9617 | | 0.64647 | 0.584017 | 0.001783 | 0.55519 |
| est7526 | 0.9085 | 0.9150 | 0.519180 | 0.007434 | 0.59070 | 0.9596 | 0.97059 | 0.626369 | 0.008060 | 0.64284 | 0.8730 | 0.86533 | 0.455674 | 0.01169 | 0.61245 | 0.8895 | | 0.64706 | 0.496223 | 0.007434 | 0.54723 |
| est8456 | 0.8827 | 0.8889 | 0.512546 | 0.004837 | 0.59584 | 1.0000 | 1.00000 | 0.693147 | 0.004950 | 0.66225 | 0.8331 | 0.78947 | 0.413493 | 0.00673 | 0.61839 | 0.8714 | | 0.66667 | 0.502858 | 0.004837 | 0.55876 |
| ahb11569 | 0.8796 | 0.8889 | 0.512546 | 0.009038 | 0.59575 | 0.9567 | 0.94118 | 0.579557 | 0.010630 | 0.64267 | 0.8724 | 0.84211 | 0.462548 | 0.00477 | 0.61902 | 0.8524 | | 0.62745 | 0.455246 | 0.009038 | 0.54926 |
| est662 | 0.9103 | 0.9118 | 0.540031 | 0.006172 | 0.59680 | 0.8788 | 0.91176 | 0.540031 | 0.007600 | 0.63884 | 0.8852 | 0.91176 | 0.540031 | 0.00768 | 0.61880 | 0.8882 | | 0.60784 | 0.514672 | 0.006172 | 0.55220 |
| ahb5635 | 0.8511 | 0.8571 | 0.477850 | 0.036377 | 0.59632 | 0.8698 | 0.82353 | 0.444495 | 0.039630 | 0.63940 | 0.9171 | 0.88889 | 0.512546 | 0.04491 | 0.62129 | 0.8184 | | 0.59259 | 0.411476 | 0.036377 | 0.54849 |
| AMB-00178689 | 0.9413 | 0.9444 | 0.584324 | 0.002350 | 0.59800 | 0.9150 | 0.88235 | 0.505105 | 0.004110 | 0.64036 | 1.0000 | 1.00000 | 0.693147 | 0.00170 | 0.65153 | 0.9308 | | 0.66667 | 0.539562 | 0.002350 | 0.56163 |
| *est10300 | 0.9706 | 0.9706 | 0.626369 | 0.001749 | 0.59816 | 0.9596 | 0.97059 | 0.626369 | 0.001880 | 0.64147 | 0.9611 | 0.97059 | 0.626369 | 0.00176 | 0.62195 | 0.9625 | | 0.64706 | 0.585271 | 0.001749 | 0.55561 |
| ahb9415 | 0.9083 | 0.9134 | 0.517341 | 0.009375 | 0.58962 | 0.9199 | 0.94118 | 0.579557 | 0.009000 | 0.64140 | 0.8761 | 0.88855 | 0.478173 | 0.01396 | 0.61426 | 0.8861 | | 0.62745 | 0.491771 | 0.009375 | 0.54746 |
| est3431 | 0.9706 | 0.9706 | 0.626369 | 0.001719 | 0.59844 | 0.9596 | 0.97059 | 0.626369 | 0.002010 | 0.64241 | 0.9611 | 0.97059 | 0.626369 | 0.00170 | 0.62078 | 0.9625 | | 0.64706 | 0.585271 | 0.001719 | 0.55603 |
| est3894 | 0.9120 | 0.9167 | 0.546253 | 0.005481 | 0.59672 | 0.9150 | 0.88235 | 0.505105 | 0.002390 | 0.64034 | 0.9602 | 0.94737 | 0.588665 | 0.00607 | 0.62257 | 0.8912 | | 0.63158 | 0.479322 | 0.005481 | 0.55077 |
| est1901 | 0.9395 | 0.9428 | 0.563335 | 0.005622 | 0.59115 | 0.9596 | 0.97059 | 0.626369 | 0.010060 | 0.64111 | 0.9178 | 0.91796 | 0.523436 | 0.00148 | 0.61539 | 0.9246 | | 0.64706 | 0.528784 | 0.005622 | 0.54850 |
| est10774 | 0.9702 | 0.9722 | 0.629296 | 0.000591 | 0.60126 | 1.0000 | 1.00000 | 0.693147 | 0.000440 | 0.66080 | 0.9594 | 0.94737 | 0.588665 | 0.00111 | 0.62347 | 0.9628 | | 0.66667 | 0.579257 | 0.000591 | 0.56469 |
| ahb2102 | 0.9115 | 0.9118 | 0.540031 | 0.006781 | 0.59631 | 0.8810 | 0.91176 | 0.540031 | 0.008790 | 0.63963 | 0.8852 | 0.91176 | 0.540031 | 0.00379 | 0.62049 | 0.8890 | | 0.60784 | 0.514672 | 0.006781 | 0.55215 |
| est814 | 0.9120 | 0.9167 | 0.546253 | 0.005175 | 0.59683 | 0.9584 | 0.94118 | 0.579557 | 0.005520 | 0.64211 | 0.9191 | 0.89474 | 0.519349 | 0.00321 | 0.62166 | 0.8904 | | 0.62745 | 0.481120 | 0.005175 | 0.54977 |
| est10857 | 0.9080 | 0.9143 | 0.543215 | 0.004679 | 0.59693 | 0.8608 | 0.81250 | 0.434176 | 0.003930 | 0.63870 | 1.0000 | 1.00000 | 0.693147 | 0.00247 | 0.65396 | 0.8977 | | 0.66667 | 0.510427 | 0.004679 | 0.56113 |
| *est5549 | 0.9706 | 0.9706 | 0.626369 | 0.001717 | 0.59810 | 0.9596 | 0.97059 | 0.626369 | 0.001820 | 0.64056 | 0.9611 | 0.97059 | 0.626369 | 0.00172 | 0.62279 | 0.9625 | | 0.64706 | 0.585271 | 0.001717 | 0.55562 |
| *ahb1291 | 0.9395 | 0.9428 | 0.563335 | 0.008074 | 0.59171 | 0.9596 | 0.97059 | 0.626369 | 0.002070 | 0.64148 | 0.9178 | 0.91796 | 0.523436 | 0.00237 | 0.61580 | 0.9246 | | 0.64706 | 0.528784 | 0.008074 | 0.54895 |
| AMB-00889927 | 0.9413 | 0.9444 | 0.584324 | 0.002539 | 0.59878 | 0.9584 | 0.94118 | 0.579557 | 0.003480 | 0.64153 | 0.9602 | 0.94737 | 0.588665 | 0.00260 | 0.62300 | 0.9258 | | 0.63158 | 0.515910 | 0.002539 | 0.55044 |
| AMB-00247891 | 0.8535 | 0.8611 | 0.482000 | 0.005594 | 0.59446 | 0.7735 | 0.70588 | 0.346350 | 0.008720 | 0.63859 | 1.0000 | 1.00000 | 0.693147 | 0.00551 | 0.64991 | 0.8542 | | 0.66667 | 0.481765 | 0.005594 | 0.56186 |
| AMB-00822311 | 0.9413 | 0.9444 | 0.584324 | 0.002796 | 0.59807 | 0.9150 | 0.88235 | 0.505105 | 0.002340 | 0.63955 | 1.0000 | 1.00000 | 0.693147 | 0.00170 | 0.65033 | 0.9308 | | 0.66667 | 0.539562 | 0.002796 | 0.56383 |
| *ahb8428 | 0.9413 | 0.9444 | 0.584324 | 0.001977 | 0.59928 | 1.0000 | 1.00000 | 0.693147 | 0.001760 | 0.66364 | 0.9191 | 0.89474 | 0.519349 | 0.00271 | 0.62073 | 0.9298 | | 0.66667 | 0.545945 | 0.001977 | 0.56094 |
| ahb10407 | 0.9120 | 0.9167 | 0.546253 | 0.002303 | 0.59748 | 1.0000 | 1.00000 | 0.693147 | 0.002880 | 0.66400 | 0.8768 | 0.84211 | 0.462548 | 0.00492 | 0.62002 | 0.8990 | | 0.66667 | 0.521521 | 0.002303 | 0.56043 |
| AMB-00120904 | 0.9706 | 0.9722 | 0.629296 | 0.000612 | 0.60100 | 0.9584 | 0.94118 | 0.579557 | 0.000570 | 0.64188 | 1.0000 | 1.00000 | 0.693147 | 0.00043 | 0.65156 | 0.9638 | | 0.66667 | 0.574676 | 0.000612 | 0.56540 |
| est8093 | 0.9085 | 0.9150 | 0.519180 | 0.005073 | 0.58970 | 0.9596 | 0.97059 | 0.626369 | 0.006200 | 0.64187 | 0.8730 | 0.86533 | 0.455674 | 0.01239 | 0.61443 | 0.8895 | | 0.64706 | 0.496223 | 0.005073 | 0.54832 |
| ahb1447 | 0.9085 | 0.9150 | 0.519180 | 0.006265 | 0.58954 | 0.8662 | 0.85294 | 0.441798 | 0.007270 | 0.63296 | 0.9611 | 0.97059 | 0.626369 | 0.00765 | 0.62320 | 0.8906 | | 0.64706 | 0.490014 | 0.006265 | 0.54858 |
| *ahb6468 | 0.9395 | 0.9428 | 0.563335 | 0.005339 | 0.59187 | 0.9596 | 0.97059 | 0.626369 | 0.005700 | 0.64114 | 0.9178 | 0.91796 | 0.523436 | 0.00175 | 0.61515 | 0.9246 | | 0.64706 | 0.528784 | 0.005339 | 0.54868 |
| est9257 | 0.9413 | 0.9444 | 0.584324 | 0.002102 | 0.59909 | 0.9584 | 0.94118 | 0.579557 | 0.003230 | 0.64216 | 0.9602 | 0.94737 | 0.588665 | 0.00238 | 0.62267 | 0.9258 | | 0.63158 | 0.515910 | 0.002102 | 0.55104 |
| AMB-00090327 | 0.8535 | 0.8611 | 0.482000 | 0.010313 | 0.59550 | 0.9584 | 0.94118 | 0.579557 | 0.009170 | 0.64123 | 0.8331 | 0.78947 | 0.413493 | 0.00676 | 0.61871 | 0.8284 | | 0.62745 | 0.435154 | 0.010313 | 0.54988 |
| AMB-00386695 | 0.9692 | 0.9688 | 0.623128 | 0.001721 | 0.59828 | 0.9578 | 0.96875 | 0.623128 | 0.000000 | 0.64113 | 0.9587 | 0.96875 | 0.623128 | 0.00403 | 0.62189 | 0.9605 | | 0.64583 | 0.582695 | 0.001721 | 0.55485 |
| ahb6833 | 0.9095 | 0.9167 | 0.546253 | 0.004058 | 0.59678 | 0.9117 | 0.88235 | 0.505105 | 0.006260 | 0.64023 | 0.9586 | 0.94737 | 0.588665 | 0.00338 | 0.62240 | 0.8869 | | 0.63158 | 0.479322 | 0.004058 | 0.54974 |
| AMB-00143758 | 0.9647 | 0.9600 | 0.608375 | 0.001860 | 0.59729 | 0.9504 | 0.96000 | 0.608375 | 0.001810 | 0.64090 | 0.9504 | 0.96000 | 0.608375 | 0.00179 | 0.62136 | 0.9524 | | 0.64000 | 0.570888 | 0.001860 | 0.55502 |
| AMB-00300500 | 0.9411 | 0.9412 | 0.579557 | 0.002416 | 0.59736 | 0.9199 | 0.94118 | 0.579557 | 0.003770 | 0.64026 | 0.9228 | 0.94118 | 0.579557 | 0.00416 | 0.62037 | 0.9255 | | 0.62745 | 0.547476 | 0.002416 | 0.55470 |
| AMB-00692843 | 0.9395 | 0.9428 | 0.563335 | 0.002689 | 0.59182 | 0.9139 | 0.91177 | 0.514510 | 0.004820 | 0.63479 | 0.9611 | 0.97059 | 0.626369 | 0.00722 | 0.62122 | 0.9248 | | 0.64706 | 0.524291 | 0.002689 | 0.54823 |
| ahb8763est5330 | 0.9085 | 0.9150 | 0.519180 | 0.003531 | 0.59015 | 0.9596 | 0.97059 | 0.626369 | 0.007500 | 0.64151 | 0.8730 | 0.86533 | 0.455674 | 0.01390 | 0.61393 | 0.8895 | | 0.64706 | 0.496223 | 0.003531 | 0.54851 |
| *est6661 | 0.8466 | 0.8595 | 0.449044 | 0.022469 | 0.58887 | 0.9596 | 0.97059 | 0.626369 | 0.015830 | 0.64193 | 0.7791 | 0.76006 | 0.352983 | 0.01995 | 0.61166 | 0.8289 | | 0.64706 | 0.454595 | 0.022469 | 0.54797 |
| AMB-00338679 | 0.9706 | 0.9722 | 0.629296 | 0.000000 | 0.60246 | 1.0000 | 1.00000 | 0.693147 | 0.000000 | 0.66155 | 0.9602 | 0.94737 | 0.588665 | 0.00000 | 0.62257 | 0.9636 | | 0.66667 | 0.579257 | 0.000000 | 0.56521 |
| AMB-00346628 | 0.8767 | 0.8788 | 0.501116 | 0.006153 | 0.59588 | 0.9563 | 0.93333 | 0.568458 | 0.009590 | 0.64167 | 0.8734 | 0.83333 | 0.453913 | 0.00458 | 0.61992 | 0.8527 | | 0.62222 | 0.445430 | 0.006153 | 0.54927 |
| AMB-01114739 | 0.9120 | 0.9167 | 0.546253 | 0.003778 | 0.59625 | 0.9584 | 0.94118 | 0.579557 | 0.003990 | 0.64208 | 0.9191 | 0.89474 | 0.519349 | 0.00395 | 0.62032 | 0.8904 | | 0.62745 | 0.481120 | 0.003778 | 0.54972 |
| ahb10278 | 0.9083 | 0.9134 | 0.517341 | 0.007822 | 0.58996 | 0.8703 | 0.88236 | 0.469429 | 0.005180 | 0.63310 | 0.9228 | 0.94118 | 0.579557 | 0.01323 | 0.62056 | 0.8864 | | 0.62745 | 0.487369 | 0.007822 | 0.54753 |
| AMB-00763721 | 0.8827 | 0.8889 | 0.512546 | 0.007000 | 0.59570 | 0.8698 | 0.82353 | 0.444495 | 0.006760 | 0.63910 | 0.9602 | 0.94737 | 0.588665 | 0.00198 | 0.62398 | 0.8600 | | 0.63158 | 0.452686 | 0.007000 | 0.55034 |
| AMB-00057479 | 0.9395 | 0.9428 | 0.563335 | 0.002702 | 0.59130 | 0.9596 | 0.97059 | 0.626369 | 0.005350 | 0.64142 | 0.9178 | 0.91796 | 0.523436 | 0.00379 | 0.61520 | 0.9246 | | 0.64706 | 0.528784 | 0.002702 | 0.54889 |
| *AMB-01112228 | 0.9085 | 0.9150 | 0.519180 | 0.008723 | 0.59022 | 0.9139 | 0.91177 | 0.514510 | 0.009810 | 0.63485 | 0.9178 | 0.91796 | 0.523436 | 0.01054 | 0.61543 | 0.8853 | | 0.61197 | 0.466275 | 0.008723 | 0.54479 |
| ahb1837 | 0.9411 | 0.9412 | 0.579557 | 0.002108 | 0.59714 | 0.9199 | 0.94118 | 0.579557 | 0.003530 | 0.64068 | 0.9228 | 0.94118 | 0.579557 | 0.00370 | 0.62060 | 0.9255 | | 0.62745 | 0.547476 | 0.002108 | 0.55466 |
| AMB-00837706 | 0.9625 | 0.9524 | 0.596291 | 0.001666 | 0.59806 | 0.9447 | 0.95238 | 0.596291 | 0.001690 | 0.64119 | 0.9461 | 0.95238 | 0.596291 | 0.00168 | 0.62246 | 0.9467 | | 0.63492 | 0.561126 | 0.001666 | 0.55506 |
| est8576 | 0.9706 | 0.9722 | 0.629296 | 0.000000 | 0.60097 | 0.9584 | 0.94118 | 0.579557 | 0.000000 | 0.64153 | 1.0000 | 1.00000 | 0.693147 | 0.00000 | 0.65073 | 0.9638 | | 0.66667 | 0.574676 | 0.000000 | 0.56610 |
| est10621 | 0.9413 | 0.9444 | 0.584324 | 0.001861 | 0.59786 | 0.9584 | 0.94118 | 0.579557 | 0.002030 | 0.64204 | 0.9602 | 0.94737 | 0.588665 | 0.00176 | 0.62399 | 0.9258 | | 0.63158 | 0.515910 | 0.001861 | 0.55212 |
| est11420 | 0.9413 | 0.9444 | 0.584324 | 0.001803 | 0.59794 | 0.9584 | 0.94118 | 0.579557 | 0.002160 | 0.64249 | 0.9602 | 0.94737 | 0.588665 | 0.00181 | 0.62259 | 0.9258 | | 0.63158 | 0.515910 | 0.001803 | 0.55128 |
| est10519 | 0.9120 | 0.9167 | 0.546253 | 0.003488 | 0.59695 | 0.9150 | 0.88235 | 0.505105 | 0.004610 | 0.63917 | 0.9602 | 0.94737 | 0.588665 | 0.00238 | 0.62333 | 0.8912 | | 0.63158 | 0.479322 | 0.003488 | 0.55096 |
| ahb657 | 0.8535 | 0.8611 | 0.482000 | 0.006439 | 0.59442 | 0.7735 | 0.70588 | 0.346350 | 0.007840 | 0.63777 | 1.0000 | 1.00000 | 0.693147 | 0.00170 | 0.65126 | 0.8542 | | 0.66667 | 0.481765 | 0.006439 | 0.55987 |
| AMB-00434246 | 0.8535 | 0.8611 | 0.482000 | 0.003977 | 0.59522 | 0.7735 | 0.70588 | 0.346350 | 0.004050 | 0.63711 | 1.0000 | 1.00000 | 0.693147 | 0.00726 | 0.65064 | 0.8542 | | 0.66667 | 0.481765 | 0.003977 | 0.56169 |
| AMB-01062287 | 0.9085 | 0.9150 | 0.519180 | 0.007472 | 0.59011 | 0.8662 | 0.85294 | 0.441798 | 0.001530 | 0.63182 | 0.9611 | 0.97059 | 0.626369 | 0.01259 | 0.62242 | 0.8906 | | 0.64706 | 0.490014 | 0.007472 | 0.54770 |
| ahb11104 | 0.9411 | 0.9412 | 0.579557 | 0.002452 | 0.59760 | 0.9199 | 0.94118 | 0.579557 | 0.002440 | 0.64047 | 0.9228 | 0.94118 | 0.579557 | 0.00306 | 0.62045 | 0.9255 | | 0.62745 | 0.547476 | 0.002452 | 0.55420 |
| ahb10996 | 0.9395 | 0.9428 | 0.563335 | 0.003339 | 0.59195 | 0.9139 | 0.91177 | 0.514510 | 0.004240 | 0.63554 | 0.9611 | 0.97059 | 0.626369 | 0.00234 | 0.62150 | 0.9248 | | 0.64706 | 0.524291 | 0.003339 | 0.54867 |
| *est8658 | 0.9120 | 0.9167 | 0.546253 | 0.002380 | 0.59676 | 1.0000 | 1.00000 | 0.693147 | 0.002450 | 0.66180 | 0.8768 | 0.84211 | 0.462548 | 0.00176 | 0.61982 | 0.8990 | | 0.66667 | 0.521521 | 0.002380 | 0.56087 |
| AMB-00931685 | 0.8945 | 0.9118 | 0.540031 | 0.006563 | 0.59426 | 0.8633 | 0.91176 | 0.540031 | 0.004850 | 0.63858 | 0.8741 | 0.91176 | 0.540031 | 0.00350 | 0.61952 | 0.8761 | | 0.60784 | 0.514672 | 0.006563 | 0.55345 |
| ahb6903 | 0.9411 | 0.9412 | 0.579557 | 0.002066 | 0.59759 | 0.9199 | 0.94118 | 0.579557 | 0.003080 | 0.64040 | 0.9228 | 0.94118 | 0.579557 | 0.00275 | 0.62097 | 0.9255 | | 0.62745 | 0.547476 | 0.002066 | 0.55343 |
| est9665 | 0.9120 | 0.9167 | 0.546253 | 0.002496 | 0.59710 | 0.8698 | 0.82353 | 0.444495 | 0.002670 | 0.63937 | 1.0000 | 1.00000 | 0.693147 | 0.00170 | 0.65173 | 0.9014 | | 0.66667 | 0.514370 | 0.002496 | 0.56225 |
| est2925 | 0.9085 | 0.9150 | 0.519180 | 0.007622 | 0.58959 | 0.9139 | 0.91177 | 0.514510 | 0.007950 | 0.63413 | 0.9178 | 0.91796 | 0.523436 | 0.01453 | 0.61504 | 0.8853 | | 0.61197 | 0.466275 | 0.007622 | 0.54463 |
| ahb8379 | 0.8535 | 0.8611 | 0.482000 | 0.008428 | 0.59574 | 0.8227 | 0.76471 | 0.392407 | 0.009670 | 0.63812 | 0.9602 | 0.94737 | 0.588665 | 0.00218 | 0.62253 | 0.8326 | | 0.63158 | 0.432524 | 0.008428 | 0.54952 |
| AMB-00967909 | 0.9386 | 0.9419 | 0.561782 | 0.003634 | 0.59149 | 0.9121 | 0.91088 | 0.512984 | 0.003600 | 0.63350 | 0.9603 | 0.96970 | 0.624791 | 0.00277 | 0.62237 | 0.9232 | | 0.64647 | 0.523064 | 0.003634 | 0.54796 |
| ahb12427 | 0.9120 | 0.9167 | 0.546253 | 0.002265 | 0.59818 | 1.0000 | 1.00000 | 0.693147 | 0.001760 | 0.66234 | 0.8768 | 0.84211 | 0.462548 | 0.00188 | 0.61930 | 0.8990 | | 0.66667 | 0.521521 | 0.002265 | 0.56144 |
| est5821 | 0.9083 | 0.9134 | 0.517341 | 0.005867 | 0.59100 | 0.8703 | 0.88236 | 0.469429 | 0.005960 | 0.63284 | 0.9228 | 0.94118 | 0.579557 | 0.00579 | 0.62055 | 0.8864 | | 0.62745 | 0.487369 | 0.005867 | 0.54843 |
| AMB-00158028 | 0.8536 | 0.8726 | 0.459320 | 0.012533 | 0.58855 | 0.7741 | 0.78186 | 0.364431 | 0.011410 | 0.63039 | 0.9503 | 0.95833 | 0.605676 | 0.00683 | 0.62134 | 0.8221 | | 0.63889 | 0.450142 | 0.012533 | 0.54803 |
| *ahb7856 | 0.9411 | 0.9412 | 0.579557 | 0.002169 | 0.59605 | 0.9199 | 0.94118 | 0.579557 | 0.002520 | 0.64014 | 0.9228 | 0.94118 | 0.579557 | 0.00277 | 0.62074 | 0.9255 | | 0.62745 | 0.547476 | 0.002169 | 0.55306 |
| ahb4562 | 0.9055 | 0.9118 | 0.514510 | 0.005343 | 0.59025 | 0.8580 | 0.83726 | 0.425055 | 0.012950 | 0.63194 | 0.9611 | 0.97059 | 0.626369 | 0.00234 | 0.62192 | 0.8895 | | 0.64706 | 0.482747 | 0.005343 | 0.54842 |
| *ahb4375 | 0.8535 | 0.8611 | 0.482000 | 0.008695 | 0.59464 | 0.8227 | 0.76471 | 0.392407 | 0.008370 | 0.63804 | 0.9602 | 0.94737 | 0.588665 | 0.00220 | 0.62229 | 0.8326 | | 0.63158 | 0.432524 | 0.008695 | 0.54996 |
| *AMB-00695295 | 0.8827 | 0.8889 | 0.512546 | 0.005428 | 0.59612 | 0.9584 | 0.94118 | 0.579557 | 0.005520 | 0.64200 | 0.8768 | 0.84211 | 0.462548 | 0.00218 | 0.61971 | 0.8578 | | 0.62745 | 0.455246 | 0.005428 | 0.54930 |
| est2458 | 0.8244 | 0.8333 | 0.453913 | 0.007600 | 0.59553 | 0.9584 | 0.94118 | 0.579557 | 0.016490 | 0.64204 | 0.7880 | 0.73684 | 0.369943 | 0.00689 | 0.61827 | 0.8023 | | 0.62745 | 0.419404 | 0.007600 | 0.54841 |
| ahb8927 | 0.9411 | 0.9412 | 0.579557 | 0.002180 | 0.59763 | 0.9199 | 0.94118 | 0.579557 | 0.002150 | 0.64072 | 0.9228 | 0.94118 | 0.579557 | 0.00245 | 0.62045 | 0.9255 | | 0.62745 | 0.547476 | 0.002180 | 0.55277 |
| AMB-00965483 | 0.9376 | 0.9410 | 0.560145 | 0.003142 | 0.59130 | 0.9578 | 0.96875 | 0.623128 | 0.002420 | 0.64219 | 0.9145 | 0.91612 | 0.520292 | 0.00376 | 0.61422 | 0.9213 | | 0.64583 | 0.526256 | 0.003142 | 0.54787 |
| ahb9515 | 0.9413 | 0.9444 | 0.584324 | 0.000459 | 0.59973 | 1.0000 | 1.00000 | 0.693147 | 0.000510 | 0.66230 | 0.9191 | 0.89474 | 0.519349 | 0.00057 | 0.62110 | 0.9298 | | 0.66667 | 0.545945 | 0.000459 | 0.56166 |
| est5859 | 0.8244 | 0.8333 | 0.453913 | 0.017266 | 0.59468 | 0.8698 | 0.82353 | 0.444495 | 0.014810 | 0.63991 | 0.8768 | 0.84211 | 0.462548 | 0.01548 | 0.61955 | 0.7844 | | 0.56141 | 0.386318 | 0.017266 | 0.54726 |
| *AMB-00702427 | 0.9374 | 0.9375 | 0.574297 | 0.002366 | 0.59571 | 0.9148 | 0.93750 | 0.574297 | 0.002690 | 0.63953 | 0.9181 | 0.93750 | 0.574297 | 0.00329 | 0.62073 | 0.9208 | | 0.62500 | 0.543156 | 0.002366 | 0.55409 |
| est2622 | 0.8535 | 0.8611 | 0.482000 | 0.005002 | 0.59515 | 0.8227 | 0.76471 | 0.392407 | 0.005500 | 0.63822 | 0.9602 | 0.94737 | 0.588665 | 0.00664 | 0.62266 | 0.8326 | | 0.63158 | 0.432524 | 0.005002 | 0.54974 |
| ahb2504 | 0.9095 | 0.9167 | 0.546253 | 0.001885 | 0.59692 | 1.0000 | 1.00000 | 0.693147 | 0.001940 | 0.66472 | 0.8724 | 0.84211 | 0.462548 | 0.00170 | 0.62037 | 0.8951 | | 0.66667 | 0.521521 | 0.001885 | 0.55974 |
| ahb7242 | 0.8827 | 0.8889 | 0.512546 | 0.005474 | 0.59603 | 0.9150 | 0.88235 | 0.505105 | 0.005710 | 0.64055 | 0.9191 | 0.89474 | 0.519349 | 0.00318 | 0.62104 | 0.8540 | | 0.59649 | 0.443085 | 0.005474 | 0.54968 |
| est10348 | 0.9411 | 0.9412 | 0.579557 | 0.002035 | 0.59745 | 0.9199 | 0.94118 | 0.579557 | 0.002260 | 0.64079 | 0.9228 | 0.94118 | 0.579557 | 0.00210 | 0.61993 | 0.9255 | | 0.62745 | 0.547476 | 0.002035 | 0.55421 |
| ahb12241 | 0.8516 | 0.8611 | 0.482000 | 0.003565 | 0.59557 | 1.0000 | 1.00000 | 0.693147 | 0.001800 | 0.66292 | 0.7846 | 0.73684 | 0.369943 | 0.00714 | 0.61813 | 0.8445 | | 0.66667 | 0.488518 | 0.003565 | 0.55873 |
| *ahb12288 | 0.8535 | 0.8611 | 0.482000 | 0.006845 | 0.59516 | 0.8698 | 0.82353 | 0.444495 | 0.007840 | 0.63878 | 0.9191 | 0.89474 | 0.519349 | 0.00760 | 0.61998 | 0.8209 | | 0.59649 | 0.415024 | 0.006845 | 0.54885 |
| ahb1303 | 0.8775 | 0.8873 | 0.481929 | 0.021837 | 0.58853 | 0.8662 | 0.85294 | 0.441798 | 0.014040 | 0.63328 | 0.9178 | 0.91796 | 0.523436 | 0.02872 | 0.61523 | 0.8493 | | 0.61197 | 0.430507 | 0.021837 | 0.54310 |
| AMB-00617969 | 0.8535 | 0.8611 | 0.482000 | 0.007239 | 0.59558 | 0.8227 | 0.76471 | 0.392407 | 0.007630 | 0.63803 | 0.9602 | 0.94737 | 0.588665 | 0.00176 | 0.62388 | 0.8326 | | 0.63158 | 0.432524 | 0.007239 | 0.54952 |
| est3855 | 0.8771 | 0.8840 | 0.478635 | 0.020692 | 0.58909 | 0.8810 | 0.91176 | 0.540031 | 0.014420 | 0.63927 | 0.8353 | 0.85913 | 0.440199 | 0.00702 | 0.61224 | 0.8482 | | 0.60784 | 0.459760 | 0.020692 | 0.54654 |
| est10243 | 0.8535 | 0.8611 | 0.482000 | 0.008293 | 0.59560 | 0.9150 | 0.88235 | 0.505105 | 0.006030 | 0.64055 | 0.8768 | 0.84211 | 0.462548 | 0.00543 | 0.61971 | 0.8195 | | 0.58823 | 0.415785 | 0.008293 | 0.54863 |
| est6375 | 0.8244 | 0.8333 | 0.453913 | 0.011319 | 0.59532 | 0.8227 | 0.76471 | 0.392407 | 0.010330 | 0.63727 | 0.9191 | 0.89474 | 0.519349 | 0.01041 | 0.62092 | 0.7916 | | 0.59649 | 0.393457 | 0.011319 | 0.54767 |
| AMB-00364848 | 0.8771 | 0.8840 | 0.478635 | 0.095204 | 0.58945 | 0.8810 | 0.91176 | 0.540031 | 0.102980 | 0.63941 | 0.8353 | 0.85913 | 0.440199 | 0.00366 | 0.61423 | 0.8482 | | 0.60784 | 0.459760 | 0.095204 | 0.54632 |
| est10000 | 0.9395 | 0.9428 | 0.563335 | 0.002263 | 0.59055 | 0.9139 | 0.91177 | 0.514510 | 0.003330 | 0.63520 | 0.9611 | 0.97059 | 0.626369 | 0.00208 | 0.62292 | 0.9248 | | 0.64706 | 0.524291 | 0.002263 | 0.54940 |
| *ahb11272 | 0.8466 | 0.8595 | 0.449044 | 0.016770 | 0.58792 | 0.7643 | 0.73530 | 0.332658 | 0.016250 | 0.62919 | 0.9611 | 0.97059 | 0.626369 | 0.00385 | 0.62217 | 0.8334 | | 0.64706 | 0.447703 | 0.016770 | 0.54825 |
| *ahb11136 | 0.8827 | 0.8889 | 0.512546 | 0.003268 | 0.59602 | 0.9584 | 0.94118 | 0.579557 | 0.002030 | 0.64252 | 0.8768 | 0.84211 | 0.462548 | 0.00673 | 0.62047 | 0.8578 | | 0.62745 | 0.455246 | 0.003268 | 0.54955 |
| *est9592 | 0.7953 | 0.8056 | 0.427820 | 0.011732 | 0.59475 | 0.7221 | 0.64706 | 0.304877 | 0.011680 | 0.63618 | 0.9602 | 0.94737 | 0.588665 | 0.00544 | 0.62291 | 0.7904 | | 0.63158 | 0.406018 | 0.011732 | 0.54882 |
| AMB-00768477 | 0.9115 | 0.9118 | 0.540031 | 0.002999 | 0.59631 | 0.8810 | 0.91176 | 0.540031 | 0.003290 | 0.63951 | 0.8852 | 0.91176 | 0.540031 | 0.00397 | 0.61946 | 0.8890 | | 0.60784 | 0.514672 | 0.002999 | 0.55408 |
| *est9813 | 0.9395 | 0.9428 | 0.563335 | 0.002367 | 0.59175 | 0.9596 | 0.97059 | 0.626369 | 0.001690 | 0.64094 | 0.9178 | 0.91796 | 0.523436 | 0.00433 | 0.61489 | 0.9246 | | 0.64706 | 0.528784 | 0.002367 | 0.54774 |
| ahb482 | 0.8827 | 0.8889 | 0.512546 | 0.002654 | 0.59522 | 1.0000 | 1.00000 | 0.693147 | 0.001760 | 0.66298 | 0.8331 | 0.78947 | 0.413493 | 0.00315 | 0.61964 | 0.8714 | | 0.66667 | 0.502858 | 0.002654 | 0.55752 |
| est4942 | 0.8535 | 0.8611 | 0.482000 | 0.007532 | 0.59606 | 0.8698 | 0.82353 | 0.444495 | 0.005460 | 0.63921 | 0.9191 | 0.89474 | 0.519349 | 0.00449 | 0.62114 | 0.8209 | | 0.59649 | 0.415024 | 0.007532 | 0.54835 |
| ahb2816 | 0.9120 | 0.9167 | 0.546253 | 0.001880 | 0.59690 | 0.9584 | 0.94118 | 0.579557 | 0.002170 | 0.64209 | 0.9191 | 0.89474 | 0.519349 | 0.00411 | 0.62035 | 0.8904 | | 0.62745 | 0.481120 | 0.001880 | 0.54941 |
| *AMB-01006277 | 0.9413 | 0.9444 | 0.584324 | 0.000000 | 0.59873 | 1.0000 | 1.00000 | 0.693147 | 0.000000 | 0.66301 | 0.9191 | 0.89474 | 0.519349 | 0.00000 | 0.62064 | 0.9298 | | 0.66667 | 0.545945 | 0.000000 | 0.56249 |
| AMB-00187437 | 0.9053 | 0.9097 | 0.512183 | 0.003825 | 0.58956 | 0.9165 | 0.93750 | 0.574297 | 0.003310 | 0.64159 | 0.8710 | 0.88487 | 0.473107 | 0.02069 | 0.61325 | 0.8811 | | 0.62500 | 0.487548 | 0.003825 | 0.54783 |
| AMB-00194823 | 0.7662 | 0.7778 | 0.403395 | 0.006610 | 0.59374 | 0.6120 | 0.52941 | 0.232214 | 0.005500 | 0.63561 | 1.0000 | 1.00000 | 0.693147 | 0.00643 | 0.65158 | 0.8166 | | 0.66667 | 0.462483 | 0.006610 | 0.56002 |
| *est11292 | 0.7662 | 0.7778 | 0.403395 | 0.011556 | 0.59380 | 0.6120 | 0.52941 | 0.232214 | 0.008940 | 0.63540 | 1.0000 | 1.00000 | 0.693147 | 0.00170 | 0.65309 | 0.8166 | | 0.66667 | 0.462483 | 0.011556 | 0.55946 |
| AMB-01059827 | 0.9085 | 0.9150 | 0.519180 | 0.004281 | 0.58999 | 0.9596 | 0.97059 | 0.626369 | 0.005200 | 0.64202 | 0.8730 | 0.86533 | 0.455674 | 0.00170 | 0.61346 | 0.8895 | | 0.64706 | 0.496223 | 0.004281 | 0.54784 |
| AMB-00731725 | 0.8827 | 0.8889 | 0.512546 | 0.001485 | 0.59618 | 0.9584 | 0.94118 | 0.579557 | 0.005280 | 0.64191 | 0.8768 | 0.84211 | 0.462548 | 0.01031 | 0.62006 | 0.8578 | | 0.62745 | 0.455246 | 0.001485 | 0.54879 |
| ahb12100 | 0.9411 | 0.9412 | 0.579557 | 0.001771 | 0.59757 | 0.9199 | 0.94118 | 0.579557 | 0.001760 | 0.64081 | 0.9228 | 0.94118 | 0.579557 | 0.00170 | 0.62083 | 0.9255 | | 0.62745 | 0.547476 | 0.001771 | 0.55266 |
| *est2088 | 0.9085 | 0.9150 | 0.519180 | 0.004986 | 0.59073 | 0.9139 | 0.91177 | 0.514510 | 0.005520 | 0.63457 | 0.9178 | 0.91796 | 0.523436 | 0.00855 | 0.61478 | 0.8853 | | 0.61197 | 0.466275 | 0.004986 | 0.54409 |
| ahb9342 | 0.8523 | 0.8529 | 0.473508 | 0.014712 | 0.59588 | 0.8053 | 0.85294 | 0.473508 | 0.019610 | 0.63738 | 0.8116 | 0.85294 | 0.473508 | 0.00177 | 0.61830 | 0.8174 | | 0.56863 | 0.457790 | 0.014712 | 0.55131 |
| est10619 | 0.8771 | 0.8840 | 0.478635 | 0.010359 | 0.58828 | 0.8810 | 0.91176 | 0.540031 | 0.004990 | 0.63946 | 0.8353 | 0.85913 | 0.440199 | 0.01559 | 0.61367 | 0.8482 | | 0.60784 | 0.459760 | 0.010359 | 0.54539 |
| ahb7150 | 0.8827 | 0.8889 | 0.512546 | 0.003334 | 0.59695 | 0.9150 | 0.88235 | 0.505105 | 0.000860 | 0.64096 | 0.9191 | 0.89474 | 0.519349 | 0.01375 | 0.62059 | 0.8540 | | 0.59649 | 0.443085 | 0.003334 | 0.54826 |
| ahb4045 | 0.8775 | 0.8873 | 0.481929 | 0.006869 | 0.58825 | 0.8164 | 0.79412 | 0.382949 | 0.007820 | 0.63124 | 0.9611 | 0.97059 | 0.626369 | 0.00208 | 0.62203 | 0.8600 | | 0.64706 | 0.465641 | 0.006869 | 0.54750 |
| *est9811 | 0.9057 | 0.9132 | 0.516042 | 0.004365 | 0.58930 | 0.9578 | 0.96875 | 0.623128 | 0.002230 | 0.64275 | 0.8681 | 0.86349 | 0.452627 | 0.00378 | 0.61323 | 0.8848 | | 0.64583 | 0.493742 | 0.004365 | 0.54772 |
| ahb11359 | 0.8404 | 0.8546 | 0.433930 | 0.020707 | 0.58839 | 0.7722 | 0.79411 | 0.362391 | 0.022460 | 0.62924 | 0.8831 | 0.91176 | 0.540031 | 0.01285 | 0.61997 | 0.8097 | | 0.60784 | 0.422879 | 0.020707 | 0.54620 |
| *est2516 | 0.9120 | 0.9167 | 0.546253 | 0.001150 | 0.59747 | 0.9150 | 0.88235 | 0.505105 | 0.000000 | 0.64077 | 0.9602 | 0.94737 | 0.588665 | 0.00550 | 0.62219 | 0.8912 | | 0.63158 | 0.479322 | 0.001150 | 0.54987 |
| *est5057 | 0.8535 | 0.8611 | 0.482000 | 0.003871 | 0.59512 | 0.8698 | 0.82353 | 0.444495 | 0.009060 | 0.63871 | 0.9191 | 0.89474 | 0.519349 | 0.00669 | 0.61984 | 0.8209 | | 0.59649 | 0.415024 | 0.003871 | 0.54820 |
| *est7384 | 0.9411 | 0.9412 | 0.579557 | 0.000733 | 0.59658 | 0.9199 | 0.94118 | 0.579557 | 0.000000 | 0.64009 | 0.9228 | 0.94118 | 0.579557 | 0.00318 | 0.62033 | 0.9255 | | 0.62745 | 0.547476 | 0.000733 | 0.55522 |
| AMB-01060814 | 0.9115 | 0.9118 | 0.540031 | 0.002316 | 0.59618 | 0.8810 | 0.91176 | 0.540031 | 0.002680 | 0.63952 | 0.8852 | 0.91176 | 0.540031 | 0.00318 | 0.61955 | 0.8890 | | 0.60784 | 0.514672 | 0.002316 | 0.55266 |
| ahb10921 | 0.7953 | 0.8056 | 0.427820 | 0.010161 | 0.59496 | 0.9584 | 0.94118 | 0.579557 | 0.007290 | 0.64183 | 0.7414 | 0.68421 | 0.330595 | 0.00424 | 0.61680 | 0.7798 | | 0.62745 | 0.407192 | 0.010161 | 0.54892 |
| ahb7843 | 0.9395 | 0.9428 | 0.563335 | 0.001432 | 0.59127 | 0.9596 | 0.97059 | 0.626369 | 0.001890 | 0.64195 | 0.9178 | 0.91796 | 0.523436 | 0.00197 | 0.61486 | 0.9246 | | 0.64706 | 0.528784 | 0.001432 | 0.54852 |
| ahb7506 | 0.8771 | 0.8840 | 0.478635 | 0.008322 | 0.59022 | 0.8277 | 0.85294 | 0.431638 | 0.009230 | 0.63269 | 0.8852 | 0.91176 | 0.540031 | 0.00362 | 0.61960 | 0.8485 | | 0.60784 | 0.455450 | 0.008322 | 0.54658 |
| *est6674 | 0.9085 | 0.9150 | 0.519180 | 0.002703 | 0.59005 | 0.9596 | 0.97059 | 0.626369 | 0.002520 | 0.64160 | 0.8730 | 0.86533 | 0.455674 | 0.00496 | 0.61308 | 0.8895 | | 0.64706 | 0.496223 | 0.002703 | 0.54754 |
| *ahb2793 | 0.8827 | 0.8889 | 0.512546 | 0.002278 | 0.59672 | 0.9584 | 0.94118 | 0.579557 | 0.003480 | 0.64193 | 0.8768 | 0.84211 | 0.462548 | 0.00379 | 0.61934 | 0.8578 | | 0.62745 | 0.455246 | 0.002278 | 0.54883 |
| AMB-00392194 | 0.8459 | 0.8546 | 0.444533 | 0.015076 | 0.58932 | 0.7860 | 0.82353 | 0.398452 | 0.009940 | 0.63100 | 0.8481 | 0.88235 | 0.505105 | 0.02611 | 0.61997 | 0.8111 | | 0.58823 | 0.426740 | 0.015076 | 0.54632 |
| est10016 | 0.9395 | 0.9428 | 0.563335 | 0.001767 | 0.59006 | 0.9139 | 0.91177 | 0.514510 | 0.002080 | 0.63446 | 0.9611 | 0.97059 | 0.626369 | 0.00180 | 0.62197 | 0.9248 | | 0.64706 | 0.524291 | 0.001767 | 0.54942 |
| ahb5308 | 0.9395 | 0.9428 | 0.563335 | 0.000693 | 0.59151 | 0.9139 | 0.91177 | 0.514510 | 0.000000 | 0.63470 | 0.9611 | 0.97059 | 0.626369 | 0.00528 | 0.62209 | 0.9248 | | 0.64706 | 0.524291 | 0.000693 | 0.54914 |
| AMB-00948140 | 0.9085 | 0.9150 | 0.519180 | 0.003044 | 0.58925 | 0.8662 | 0.85294 | 0.441798 | 0.003320 | 0.63346 | 0.9611 | 0.97059 | 0.626369 | 0.00339 | 0.62118 | 0.8906 | | 0.64706 | 0.490014 | 0.003044 | 0.54826 |
| *AMB-00108359 | 0.8994 | 0.9087 | 0.508633 | 0.004638 | 0.58952 | 0.8485 | 0.84664 | 0.431644 | 0.005160 | 0.63179 | 0.9559 | 0.96429 | 0.615471 | 0.00234 | 0.62081 | 0.8751 | | 0.64286 | 0.481692 | 0.004638 | 0.54771 |
| AMB-00717504 | 0.8972 | 0.9063 | 0.533182 | 0.001812 | 0.59578 | 0.9528 | 0.94118 | 0.579557 | 0.001760 | 0.64212 | 0.8983 | 0.86667 | 0.487895 | 0.00338 | 0.62026 | 0.8716 | | 0.62745 | 0.466473 | 0.001812 | 0.55052 |
| ahb6062 | 0.8466 | 0.8595 | 0.449044 | 0.006149 | 0.58786 | 0.9596 | 0.97059 | 0.626369 | 0.004860 | 0.64202 | 0.7791 | 0.76006 | 0.352983 | 0.01127 | 0.61081 | 0.8289 | | 0.64706 | 0.454595 | 0.006149 | 0.54700 |
| *est8319 | 0.9085 | 0.9150 | 0.519180 | 0.003455 | 0.59058 | 0.9596 | 0.97059 | 0.626369 | 0.004210 | 0.64116 | 0.8730 | 0.86533 | 0.455674 | 0.00000 | 0.61411 | 0.8895 | | 0.64706 | 0.496223 | 0.003455 | 0.54803 |
| *est4637 | 0.8771 | 0.8840 | 0.478635 | 0.008019 | 0.58910 | 0.8810 | 0.91176 | 0.540031 | 0.011650 | 0.63899 | 0.8353 | 0.85913 | 0.440199 | 0.00344 | 0.61345 | 0.8482 | | 0.60784 | 0.459760 | 0.008019 | 0.54617 |
| est4672 | 0.8775 | 0.8873 | 0.481929 | 0.004542 | 0.58943 | 0.9596 | 0.97059 | 0.626369 | 0.004930 | 0.64116 | 0.8268 | 0.81270 | 0.400444 | 0.00411 | 0.61204 | 0.8575 | | 0.64706 | 0.472536 | 0.004542 | 0.54670 |
| est3715 | 0.9120 | 0.9167 | 0.546253 | 0.001771 | 0.59639 | 0.9150 | 0.88235 | 0.505105 | 0.001760 | 0.64020 | 0.9602 | 0.94737 | 0.588665 | 0.00170 | 0.62306 | 0.8912 | | 0.63158 | 0.479322 | 0.001771 | 0.55006 |
| ahb2063 | 0.8418 | 0.8554 | 0.444028 | 0.005779 | 0.58883 | 0.9579 | 0.96970 | 0.624791 | 0.006150 | 0.64257 | 0.7752 | 0.75917 | 0.351594 | 0.00969 | 0.61030 | 0.8232 | | 0.64647 | 0.453432 | 0.005779 | 0.54678 |
| AMB-00370876 | 0.8827 | 0.8889 | 0.512546 | 0.001771 | 0.59671 | 1.0000 | 1.00000 | 0.693147 | 0.001760 | 0.66302 | 0.8331 | 0.78947 | 0.413493 | 0.00170 | 0.61840 | 0.8714 | | 0.66667 | 0.502858 | 0.001771 | 0.56063 |
| AMB-00166666 | 0.9120 | 0.9167 | 0.546253 | 0.000060 | 0.59609 | 1.0000 | 1.00000 | 0.693147 | 0.000040 | 0.66220 | 0.8768 | 0.84211 | 0.462548 | 0.00014 | 0.62008 | 0.8990 | | 0.66667 | 0.521521 | 0.000060 | 0.56135 |
| AMB-00551238 | 0.8775 | 0.8873 | 0.481929 | 0.010444 | 0.58865 | 0.8662 | 0.85294 | 0.441798 | 0.013910 | 0.63257 | 0.9178 | 0.91796 | 0.523436 | 0.00397 | 0.61521 | 0.8493 | | 0.61197 | 0.430507 | 0.010444 | 0.54260 |
| AMB-00453032 | 0.9395 | 0.9428 | 0.563335 | 0.001533 | 0.59129 | 0.9139 | 0.91177 | 0.514510 | 0.001500 | 0.63528 | 0.9611 | 0.97059 | 0.626369 | 0.00181 | 0.62103 | 0.9248 | | 0.64706 | 0.524291 | 0.001533 | 0.54905 |
| AMB-00568495 | 0.9395 | 0.9428 | 0.563335 | 0.001453 | 0.59141 | 0.9596 | 0.97059 | 0.626369 | 0.001290 | 0.64196 | 0.9178 | 0.91796 | 0.523436 | 0.00157 | 0.61510 | 0.9246 | | 0.64706 | 0.528784 | 0.001453 | 0.54839 |
| AMB-00302826 | 0.8244 | 0.8333 | 0.453913 | 0.005577 | 0.59587 | 0.7735 | 0.70588 | 0.346350 | 0.006090 | 0.63707 | 0.9602 | 0.94737 | 0.588665 | 0.00200 | 0.62301 | 0.8093 | | 0.63158 | 0.417256 | 0.005577 | 0.54886 |
| AMB-00857424 | 0.8771 | 0.8840 | 0.478635 | 0.006120 | 0.58839 | 0.8810 | 0.91176 | 0.540031 | 0.005980 | 0.64014 | 0.8353 | 0.85913 | 0.440199 | 0.00834 | 0.61245 | 0.8482 | | 0.60784 | 0.459760 | 0.006120 | 0.54683 |
| est7368 | 0.9411 | 0.9412 | 0.579557 | 0.000190 | 0.59692 | 0.9199 | 0.94118 | 0.579557 | 0.000760 | 0.64086 | 0.9228 | 0.94118 | 0.579557 | 0.00062 | 0.62055 | 0.9255 | | 0.62745 | 0.547476 | 0.000190 | 0.55439 |
| AMB-00755979 | 0.8535 | 0.8611 | 0.482000 | 0.002724 | 0.59509 | 0.9584 | 0.94118 | 0.579557 | 0.005120 | 0.64329 | 0.8331 | 0.78947 | 0.413493 | 0.00345 | 0.61805 | 0.8284 | | 0.62745 | 0.435154 | 0.002724 | 0.54881 |
| AMB-00729255 | 0.8535 | 0.8611 | 0.482000 | 0.001905 | 0.59486 | 1.0000 | 1.00000 | 0.693147 | 0.002500 | 0.66234 | 0.7880 | 0.73684 | 0.369943 | 0.00235 | 0.61832 | 0.8472 | | 0.66667 | 0.488518 | 0.001905 | 0.55850 |
| ahb9838 | 0.8771 | 0.8840 | 0.478635 | 0.003998 | 0.58946 | 0.8810 | 0.91176 | 0.540031 | 0.006350 | 0.64005 | 0.8353 | 0.85913 | 0.440199 | 0.00992 | 0.61384 | 0.8482 | | 0.60784 | 0.459760 | 0.003998 | 0.54695 |
| AMB-00930007 | 0.8827 | 0.8889 | 0.512546 | 0.002686 | 0.59683 | 0.9150 | 0.88235 | 0.505105 | 0.003790 | 0.63960 | 0.9191 | 0.89474 | 0.519349 | 0.00245 | 0.62107 | 0.8540 | | 0.59649 | 0.443085 | 0.002686 | 0.54937 |
| *ahb11801 | 0.8244 | 0.8333 | 0.453913 | 0.005331 | 0.59535 | 0.7735 | 0.70588 | 0.346350 | 0.005960 | 0.63760 | 0.9602 | 0.94737 | 0.588665 | 0.00179 | 0.62283 | 0.8093 | | 0.63158 | 0.417256 | 0.005331 | 0.54961 |
| ahb12413 | 0.9054 | 0.9108 | 0.513448 | 0.003197 | 0.59029 | 0.9166 | 0.93939 | 0.576994 | 0.004960 | 0.64158 | 0.8736 | 0.88676 | 0.475703 | 0.00313 | 0.61243 | 0.8826 | | 0.62626 | 0.489714 | 0.003197 | 0.54702 |
| est4237 | 0.7372 | 0.7500 | 0.380396 | 0.021970 | 0.59173 | 0.6683 | 0.58824 | 0.267050 | 0.024770 | 0.63701 | 0.9191 | 0.89474 | 0.519349 | 0.02633 | 0.62087 | 0.7294 | | 0.59649 | 0.355086 | 0.021970 | 0.54816 |
| est9593 | 0.8244 | 0.8333 | 0.453913 | 0.003780 | 0.59478 | 0.9584 | 0.94118 | 0.579557 | 0.003250 | 0.64140 | 0.7880 | 0.73684 | 0.369943 | 0.01466 | 0.61726 | 0.8023 | | 0.62745 | 0.419404 | 0.003780 | 0.54862 |
| ahb2046 | 0.8745 | 0.8611 | 0.451790 | 0.006158 | 0.58877 | 0.8792 | 0.88889 | 0.512546 | 0.011400 | 0.63977 | 0.8161 | 0.83626 | 0.413924 | 0.00895 | 0.61230 | 0.8210 | | 0.59259 | 0.437131 | 0.006158 | 0.54617 |
| ahb6662 | 0.8757 | 0.8856 | 0.474003 | 0.004379 | 0.58953 | 0.8186 | 0.82353 | 0.398452 | 0.006330 | 0.63062 | 0.9228 | 0.94118 | 0.579557 | 0.00684 | 0.62085 | 0.8509 | | 0.62745 | 0.453939 | 0.004379 | 0.54660 |
| AMB-01067455 | 0.9095 | 0.9167 | 0.546253 | 0.000051 | 0.59676 | 1.0000 | 1.00000 | 0.693147 | 0.000000 | 0.66304 | 0.8724 | 0.84211 | 0.462548 | 0.00041 | 0.61899 | 0.8951 | | 0.66667 | 0.521521 | 0.000051 | 0.55984 |
| AMB-01103568 | 0.9070 | 0.9135 | 0.516901 | 0.004290 | 0.58855 | 0.9117 | 0.90809 | 0.509358 | 0.004790 | 0.63458 | 0.9178 | 0.91796 | 0.523436 | 0.00512 | 0.61494 | 0.8842 | | 0.61197 | 0.463616 | 0.004290 | 0.54476 |
| *ahb7938 | 0.8827 | 0.8889 | 0.512546 | 0.002393 | 0.59654 | 0.8698 | 0.82353 | 0.444495 | 0.002010 | 0.63891 | 0.9602 | 0.94737 | 0.588665 | 0.00209 | 0.62203 | 0.8600 | | 0.63158 | 0.452686 | 0.002393 | 0.55055 |
| AMB-00504979 | 0.7309 | 0.6154 | 0.284103 | 0.007578 | 0.59140 | 0.3487 | 0.16667 | 0.061555 | 0.005280 | 0.63258 | 1.0000 | 1.00000 | 0.693147 | 0.01094 | 0.63832 | 0.9366 | | 0.66667 | 0.518061 | 0.007578 | 0.56072 |
| est9211 | 0.9083 | 0.9134 | 0.517341 | 0.002717 | 0.58954 | 0.9199 | 0.94118 | 0.579557 | 0.003120 | 0.64070 | 0.8761 | 0.88855 | 0.478173 | 0.00377 | 0.61464 | 0.8861 | | 0.62745 | 0.491771 | 0.002717 | 0.54768 |
| est7418 | 0.9085 | 0.9150 | 0.519180 | 0.002416 | 0.59034 | 0.8662 | 0.85294 | 0.441798 | 0.002550 | 0.63247 | 0.9611 | 0.97059 | 0.626369 | 0.00257 | 0.62220 | 0.8906 | | 0.64706 | 0.490014 | 0.002416 | 0.54813 |
| AMB-00609176 | 0.8446 | 0.8261 | 0.446929 | 0.007407 | 0.59404 | 0.8968 | 0.83333 | 0.453913 | 0.002750 | 0.63918 | 0.8928 | 0.81818 | 0.439456 | 0.01417 | 0.61837 | 0.8179 | | 0.55555 | 0.379803 | 0.007407 | 0.54651 |
| AMB-00149822 | 0.8716 | 0.8815 | 0.474353 | 0.006606 | 0.58910 | 0.9121 | 0.91088 | 0.512984 | 0.007370 | 0.63480 | 0.8635 | 0.85205 | 0.440326 | 0.01596 | 0.61298 | 0.8424 | | 0.60725 | 0.424487 | 0.006606 | 0.54227 |
| est8385 | 0.9083 | 0.9134 | 0.517341 | 0.003023 | 0.58941 | 0.9199 | 0.94118 | 0.579557 | 0.001760 | 0.63983 | 0.8761 | 0.88855 | 0.478173 | 0.00648 | 0.61326 | 0.8861 | | 0.62745 | 0.491771 | 0.003023 | 0.54818 |
| *ahb2222 | 0.8428 | 0.8562 | 0.436116 | 0.008904 | 0.58771 | 0.8810 | 0.91176 | 0.540031 | 0.009880 | 0.63992 | 0.7838 | 0.80650 | 0.375536 | 0.01193 | 0.61172 | 0.8107 | | 0.60784 | 0.428732 | 0.008904 | 0.54617 |
| AMB-00679056 | 0.7953 | 0.8056 | 0.427820 | 0.006236 | 0.59469 | 0.7221 | 0.64706 | 0.304877 | 0.007550 | 0.63750 | 0.9602 | 0.94737 | 0.588665 | 0.00274 | 0.62139 | 0.7904 | | 0.63158 | 0.406018 | 0.006236 | 0.54945 |
| *AMB-00501924 | 0.9395 | 0.9428 | 0.563335 | 0.000630 | 0.59081 | 0.9596 | 0.97059 | 0.626369 | 0.000910 | 0.64273 | 0.9178 | 0.91796 | 0.523436 | 0.00043 | 0.61507 | 0.9246 | | 0.64706 | 0.528784 | 0.000630 | 0.54818 |
| AMB-01011676 | 0.8535 | 0.8611 | 0.482000 | 0.002350 | 0.59460 | 0.9584 | 0.94118 | 0.579557 | 0.002030 | 0.64191 | 0.8331 | 0.78947 | 0.413493 | 0.00635 | 0.61919 | 0.8284 | | 0.62745 | 0.435154 | 0.002350 | 0.54893 |
| *est9209 | 0.9083 | 0.9134 | 0.517341 | 0.002717 | 0.58914 | 0.9199 | 0.94118 | 0.579557 | 0.003120 | 0.64035 | 0.8761 | 0.88855 | 0.478173 | 0.00377 | 0.61385 | 0.8861 | | 0.62745 | 0.491771 | 0.002717 | 0.54761 |
| *est10746 | 0.9085 | 0.9150 | 0.519180 | 0.001955 | 0.58982 | 0.8662 | 0.85294 | 0.441798 | 0.003470 | 0.63340 | 0.9611 | 0.97059 | 0.626369 | 0.00177 | 0.62218 | 0.8906 | | 0.64706 | 0.490014 | 0.001955 | 0.54821 |
| ahb5244 | 0.8226 | 0.8235 | 0.444495 | 0.006409 | 0.59515 | 0.7684 | 0.82353 | 0.444495 | 0.007040 | 0.63800 | 0.7757 | 0.82353 | 0.444495 | 0.00900 | 0.61859 | 0.7823 | | 0.54902 | 0.432360 | 0.006409 | 0.55208 |
| AMB-00292319 | 0.9085 | 0.9150 | 0.519180 | 0.003035 | 0.58977 | 0.9139 | 0.91177 | 0.514510 | 0.004810 | 0.63489 | 0.9178 | 0.91796 | 0.523436 | 0.00423 | 0.61532 | 0.8853 | | 0.61197 | 0.466275 | 0.003035 | 0.54444 |
| ahb4448 | 0.8827 | 0.8889 | 0.512546 | 0.001393 | 0.59510 | 0.8227 | 0.76471 | 0.392407 | 0.001490 | 0.63794 | 1.0000 | 1.00000 | 0.693147 | 0.00043 | 0.65065 | 0.8757 | | 0.66667 | 0.495630 | 0.001393 | 0.56248 |
| ahb12360 | 0.9083 | 0.9134 | 0.517341 | 0.002795 | 0.59034 | 0.9199 | 0.94118 | 0.579557 | 0.002710 | 0.63981 | 0.8761 | 0.88855 | 0.478173 | 0.00382 | 0.61346 | 0.8861 | | 0.62745 | 0.491771 | 0.002795 | 0.54691 |
| ahb7537 | 0.8158 | 0.8317 | 0.419326 | 0.006184 | 0.58659 | 0.7099 | 0.67647 | 0.288447 | 0.007910 | 0.62797 | 0.9611 | 0.97059 | 0.626369 | 0.00607 | 0.62207 | 0.8110 | | 0.64706 | 0.434630 | 0.006184 | 0.54802 |
| est5492 | 0.9106 | 0.9143 | 0.543215 | 0.000815 | 0.59754 | 0.9574 | 0.93750 | 0.574297 | 0.001160 | 0.64158 | 0.9191 | 0.89474 | 0.519349 | 0.00085 | 0.62076 | 0.8891 | | 0.62500 | 0.478318 | 0.000815 | 0.55055 |
| *AMB-00853505 | 0.8755 | 0.8849 | 0.478961 | 0.005982 | 0.58973 | 0.8662 | 0.85294 | 0.441798 | 0.010190 | 0.63308 | 0.9159 | 0.91503 | 0.519180 | 0.00429 | 0.61482 | 0.8474 | | 0.61002 | 0.428192 | 0.005982 | 0.54386 |
| AMB-00854008 | 0.8226 | 0.8235 | 0.444495 | 0.009120 | 0.59363 | 0.7684 | 0.82353 | 0.444495 | 0.006060 | 0.63786 | 0.7757 | 0.82353 | 0.444495 | 0.00527 | 0.61878 | 0.7823 | | 0.54902 | 0.432360 | 0.009120 | 0.54983 |
| *est1223 | 0.8820 | 0.8824 | 0.505105 | 0.002136 | 0.59488 | 0.8428 | 0.88235 | 0.505105 | 0.004340 | 0.63857 | 0.8481 | 0.88235 | 0.505105 | 0.00466 | 0.61890 | 0.8530 | | 0.58823 | 0.485061 | 0.002136 | 0.55064 |
| est3992 | 0.8244 | 0.8333 | 0.453913 | 0.004826 | 0.59502 | 0.8227 | 0.76471 | 0.392407 | 0.003230 | 0.63745 | 0.9191 | 0.89474 | 0.519349 | 0.00781 | 0.62123 | 0.7916 | | 0.59649 | 0.393457 | 0.004826 | 0.54693 |
| ahb1532 | 0.7644 | 0.8056 | 0.427820 | 0.010142 | 0.59385 | 0.9469 | 0.94118 | 0.579557 | 0.009810 | 0.64055 | 0.6907 | 0.68421 | 0.330595 | 0.00170 | 0.61723 | 0.7303 | | 0.62745 | 0.407192 | 0.010142 | 0.54821 |
| AMB-00445292 | 0.8587 | 0.8767 | 0.464818 | 0.003667 | 0.58859 | 0.9504 | 0.96000 | 0.608375 | 0.002530 | 0.64136 | 0.7929 | 0.80211 | 0.384128 | 0.00796 | 0.61100 | 0.8260 | | 0.64000 | 0.458973 | 0.003667 | 0.54722 |
| AMB-01061243 | 0.8775 | 0.8873 | 0.481929 | 0.005717 | 0.58979 | 0.8662 | 0.85294 | 0.441798 | 0.007140 | 0.63284 | 0.9178 | 0.91796 | 0.523436 | 0.00436 | 0.61451 | 0.8493 | | 0.61197 | 0.430507 | 0.005717 | 0.54305 |
| AMB-00772725 | 0.8466 | 0.8595 | 0.449044 | 0.008893 | 0.58877 | 0.8164 | 0.79412 | 0.382949 | 0.004390 | 0.63176 | 0.9178 | 0.91796 | 0.523436 | 0.01347 | 0.61572 | 0.8170 | | 0.61197 | 0.404676 | 0.008893 | 0.54230 |
| AMB-00608704 | 0.8466 | 0.8595 | 0.449044 | 0.004458 | 0.58779 | 0.9596 | 0.97059 | 0.626369 | 0.004730 | 0.64192 | 0.7791 | 0.76006 | 0.352983 | 0.00452 | 0.61131 | 0.8289 | | 0.64706 | 0.454595 | 0.004458 | 0.54718 |
| ahb1172 | 0.6793 | 0.6944 | 0.337961 | 0.009255 | 0.59215 | 1.0000 | 1.00000 | 0.693147 | 0.001760 | 0.66382 | 0.4832 | 0.42105 | 0.174338 | 0.00473 | 0.61538 | 0.7872 | | 0.66667 | 0.464885 | 0.009255 | 0.55875 |
| *est5351 | 0.8535 | 0.8611 | 0.482000 | 0.002089 | 0.59639 | 0.8227 | 0.76471 | 0.392407 | 0.001950 | 0.63889 | 0.9602 | 0.94737 | 0.588665 | 0.00275 | 0.62342 | 0.8326 | | 0.63158 | 0.432524 | 0.002089 | 0.54957 |
| AMB-01042893 | 0.9083 | 0.9134 | 0.517341 | 0.001963 | 0.59074 | 0.9199 | 0.94118 | 0.579557 | 0.001820 | 0.64081 | 0.8761 | 0.88855 | 0.478173 | 0.00264 | 0.61486 | 0.8861 | | 0.62745 | 0.491771 | 0.001963 | 0.54856 |
| AMB-00580429 | 0.7777 | 0.8333 | 0.453913 | 0.002794 | 0.59397 | 1.0000 | 1.00000 | 0.693147 | 0.001790 | 0.65858 | 0.6531 | 0.68421 | 0.330595 | 0.00282 | 0.61738 | 0.7513 | | 0.66667 | 0.477702 | 0.002794 | 0.55875 |
| est8053 | 0.7662 | 0.7778 | 0.403395 | 0.007608 | 0.59405 | 0.7221 | 0.64706 | 0.304877 | 0.008210 | 0.63660 | 0.9191 | 0.89474 | 0.519349 | 0.01028 | 0.62092 | 0.7455 | | 0.59649 | 0.364178 | 0.007608 | 0.54661 |
| ahb11808 | 0.8511 | 0.8571 | 0.477850 | 0.001771 | 0.59490 | 0.7735 | 0.70588 | 0.346350 | 0.001760 | 0.63771 | 1.0000 | 1.00000 | 0.693147 | 0.00170 | 0.64848 | 0.8517 | | 0.66667 | 0.481765 | 0.001771 | 0.56028 |
| est540 | 0.7082 | 0.7222 | 0.358634 | 0.010043 | 0.59334 | 0.5530 | 0.47059 | 0.199887 | 0.010260 | 0.63548 | 0.9602 | 0.94737 | 0.588665 | 0.00338 | 0.62254 | 0.7630 | | 0.63158 | 0.392448 | 0.010043 | 0.54859 |
| AMB-00695511 | 0.8523 | 0.8529 | 0.473508 | 0.003380 | 0.59483 | 0.8053 | 0.85294 | 0.473508 | 0.004010 | 0.63979 | 0.8116 | 0.85294 | 0.473508 | 0.00378 | 0.61976 | 0.8174 | | 0.56863 | 0.457790 | 0.003380 | 0.55181 |
| AMB-00389856 | 0.9115 | 0.9118 | 0.540031 | 0.000815 | 0.59650 | 0.8810 | 0.91176 | 0.540031 | 0.000960 | 0.63946 | 0.8852 | 0.91176 | 0.540031 | 0.00163 | 0.62037 | 0.8890 | | 0.60784 | 0.514672 | 0.000815 | 0.55216 |
| ahb4891 | 0.8535 | 0.8611 | 0.482000 | 0.002255 | 0.59497 | 0.8698 | 0.82353 | 0.444495 | 0.006260 | 0.63925 | 0.9191 | 0.89474 | 0.519349 | 0.00240 | 0.62104 | 0.8209 | | 0.59649 | 0.415024 | 0.002255 | 0.54822 |
| est5099 | 0.8745 | 0.8889 | 0.512546 | 0.000347 | 0.59508 | 0.8062 | 0.76471 | 0.392407 | 0.000370 | 0.63868 | 1.0000 | 1.00000 | 0.693147 | 0.00072 | 0.64799 | 0.8632 | | 0.66667 | 0.495630 | 0.000347 | 0.55954 |
| AMB-01092352 | 0.8757 | 0.8864 | 0.480425 | 0.002662 | 0.58941 | 0.8129 | 0.79323 | 0.381530 | 0.003330 | 0.63102 | 0.9603 | 0.96970 | 0.624791 | 0.00308 | 0.62163 | 0.8572 | | 0.64647 | 0.464464 | 0.002662 | 0.54782 |
| *AMB-00824536 | 0.9115 | 0.9118 | 0.540031 | 0.001120 | 0.59566 | 0.8810 | 0.91176 | 0.540031 | 0.000630 | 0.63967 | 0.8852 | 0.91176 | 0.540031 | 0.00103 | 0.61986 | 0.8890 | | 0.60784 | 0.514672 | 0.001120 | 0.55364 |
| AMB-00464977 | 0.7662 | 0.7778 | 0.403395 | 0.007621 | 0.59389 | 0.9584 | 0.94118 | 0.579557 | 0.001090 | 0.64174 | 0.6932 | 0.63158 | 0.294600 | 0.00755 | 0.61670 | 0.7612 | | 0.62745 | 0.398028 | 0.007621 | 0.54857 |
| est3280 | 0.6504 | 0.6667 | 0.318257 | 0.013675 | 0.59264 | 0.9584 | 0.94118 | 0.579557 | 0.019630 | 0.64048 | 0.4832 | 0.42105 | 0.174338 | 0.01014 | 0.61393 | 0.7318 | | 0.62745 | 0.387474 | 0.013675 | 0.54813 |
| AMB-00761887 | 0.9108 | 0.9167 | 0.546253 | 0.000000 | 0.59660 | 0.9134 | 0.88235 | 0.505105 | 0.000000 | 0.63992 | 0.9594 | 0.94737 | 0.588665 | 0.00000 | 0.62415 | 0.8891 | | 0.63158 | 0.479322 | 0.000000 | 0.55082 |
| *est3868 | 0.8431 | 0.8579 | 0.437570 | 0.009354 | 0.58754 | 0.8703 | 0.88236 | 0.469429 | 0.010410 | 0.63292 | 0.8279 | 0.83592 | 0.411962 | 0.01277 | 0.61190 | 0.8073 | | 0.58824 | 0.396799 | 0.009354 | 0.54174 |
| ahb10590 | 0.8244 | 0.8333 | 0.453913 | 0.002901 | 0.59460 | 0.8227 | 0.76471 | 0.392407 | 0.005170 | 0.63893 | 0.9191 | 0.89474 | 0.519349 | 0.00479 | 0.62027 | 0.7916 | | 0.59649 | 0.393457 | 0.002901 | 0.54817 |
| *est8202 | 0.7620 | 0.7714 | 0.398020 | 0.006005 | 0.59482 | 0.9574 | 0.93750 | 0.574297 | 0.012010 | 0.64176 | 0.6932 | 0.63158 | 0.294600 | 0.00106 | 0.61708 | 0.7563 | | 0.62500 | 0.394786 | 0.006005 | 0.54942 |
| *ahb4376 | 0.8244 | 0.8333 | 0.453913 | 0.003581 | 0.59492 | 0.9584 | 0.94118 | 0.579557 | 0.002290 | 0.64180 | 0.7880 | 0.73684 | 0.369943 | 0.00256 | 0.61738 | 0.8023 | | 0.62745 | 0.419404 | 0.003581 | 0.54894 |
| AMB-01111335 | 0.8405 | 0.8555 | 0.434672 | 0.004606 | 0.58765 | 0.9184 | 0.94118 | 0.579557 | 0.004580 | 0.64050 | 0.7782 | 0.78329 | 0.358291 | 0.00732 | 0.61113 | 0.8145 | | 0.62745 | 0.437028 | 0.004606 | 0.54718 |
| *ahb10914 | 0.7543 | 0.7762 | 0.366819 | 0.011166 | 0.58669 | 0.5934 | 0.55883 | 0.213063 | 0.010750 | 0.62573 | 0.9611 | 0.97059 | 0.626369 | 0.00208 | 0.62213 | 0.7800 | | 0.64706 | 0.420006 | 0.011166 | 0.54826 |
| *est10416 | 0.8431 | 0.8579 | 0.437570 | 0.005195 | 0.58788 | 0.9199 | 0.94118 | 0.579557 | 0.003770 | 0.64082 | 0.7782 | 0.78329 | 0.358291 | 0.00867 | 0.60911 | 0.8167 | | 0.62745 | 0.437028 | 0.005195 | 0.54574 |
| AMB-00734421 | 0.8471 | 0.8438 | 0.464191 | 0.004069 | 0.59472 | 0.7968 | 0.84375 | 0.464191 | 0.001760 | 0.63919 | 0.8036 | 0.84375 | 0.464191 | 0.00468 | 0.61813 | 0.8091 | | 0.56250 | 0.449664 | 0.004069 | 0.55163 |
| est3350 | 0.8771 | 0.8840 | 0.478635 | 0.003083 | 0.58929 | 0.8810 | 0.91176 | 0.540031 | 0.004480 | 0.64093 | 0.8353 | 0.85913 | 0.440199 | 0.00376 | 0.61193 | 0.8482 | | 0.60784 | 0.459760 | 0.003083 | 0.54645 |
| *est1833 | 0.7082 | 0.7222 | 0.358634 | 0.014747 | 0.59238 | 0.6120 | 0.52941 | 0.232214 | 0.013730 | 0.63591 | 0.9191 | 0.89474 | 0.519349 | 0.00458 | 0.62092 | 0.7184 | | 0.59649 | 0.349231 | 0.014747 | 0.54760 |
| est6796 | 0.8757 | 0.8856 | 0.474003 | 0.003576 | 0.58811 | 0.9199 | 0.94118 | 0.579557 | 0.004040 | 0.64022 | 0.8279 | 0.83592 | 0.411962 | 0.00280 | 0.61165 | 0.8498 | | 0.62745 | 0.459971 | 0.003576 | 0.54672 |
| AMB-00471388 | 0.8827 | 0.8889 | 0.512546 | 0.000856 | 0.59651 | 0.8698 | 0.82353 | 0.444495 | 0.000000 | 0.63890 | 0.9602 | 0.94737 | 0.588665 | 0.00120 | 0.62248 | 0.8600 | | 0.63158 | 0.452686 | 0.000856 | 0.54972 |
| ahb10049 | 0.8244 | 0.8333 | 0.453913 | 0.003724 | 0.59544 | 0.8698 | 0.82353 | 0.444495 | 0.004650 | 0.63974 | 0.8768 | 0.84211 | 0.462548 | 0.00281 | 0.62059 | 0.7844 | | 0.56141 | 0.386318 | 0.003724 | 0.54751 |
| *est7488 | 0.8431 | 0.8579 | 0.437570 | 0.010886 | 0.58775 | 0.8703 | 0.88236 | 0.469429 | 0.005460 | 0.63302 | 0.8279 | 0.83592 | 0.411962 | 0.01371 | 0.61086 | 0.8073 | | 0.58824 | 0.396799 | 0.010886 | 0.54068 |
| AMB-01054893 | 0.8466 | 0.8595 | 0.449044 | 0.004931 | 0.58840 | 0.9596 | 0.97059 | 0.626369 | 0.002760 | 0.64249 | 0.7791 | 0.76006 | 0.352983 | 0.00114 | 0.61062 | 0.8289 | | 0.64706 | 0.454595 | 0.004931 | 0.54725 |
| ahb621 | 0.8145 | 0.8252 | 0.413764 | 0.011530 | 0.58813 | 0.8053 | 0.85294 | 0.473508 | 0.006990 | 0.63842 | 0.7557 | 0.80031 | 0.376800 | 0.00950 | 0.61195 | 0.7740 | | 0.56863 | 0.404508 | 0.011530 | 0.54574 |
| AMB-00892550 | 0.8775 | 0.8873 | 0.481929 | 0.003617 | 0.58873 | 0.9139 | 0.91177 | 0.514510 | 0.003080 | 0.63475 | 0.8730 | 0.86533 | 0.455674 | 0.00580 | 0.61414 | 0.8485 | | 0.60785 | 0.432220 | 0.003617 | 0.54324 |
| AMB-00284342 | 0.7333 | 0.7353 | 0.368731 | 0.027418 | 0.59301 | 0.6619 | 0.73529 | 0.368731 | 0.025290 | 0.63763 | 0.6713 | 0.73529 | 0.368731 | 0.01633 | 0.61629 | 0.6795 | | 0.49019 | 0.364245 | 0.027418 | 0.54870 |
| ahb11850 | 0.8459 | 0.8546 | 0.444533 | 0.006356 | 0.58930 | 0.7860 | 0.82353 | 0.398452 | 0.006610 | 0.63074 | 0.8481 | 0.88235 | 0.505105 | 0.00448 | 0.61998 | 0.8111 | | 0.58823 | 0.426740 | 0.006356 | 0.54511 |
| AMB-01139336 | 0.7916 | 0.8000 | 0.422810 | 0.004807 | 0.59271 | 0.7735 | 0.70588 | 0.346350 | 0.003970 | 0.63744 | 0.9171 | 0.88889 | 0.512546 | 0.00728 | 0.62074 | 0.7621 | | 0.59259 | 0.372940 | 0.004807 | 0.54721 |
| est3864 | 0.8771 | 0.8840 | 0.478635 | 0.003099 | 0.59023 | 0.8810 | 0.91176 | 0.540031 | 0.002780 | 0.63981 | 0.8353 | 0.85913 | 0.440199 | 0.00418 | 0.61291 | 0.8482 | | 0.60784 | 0.459760 | 0.003099 | 0.54631 |
| est11285 | 0.7953 | 0.8056 | 0.427820 | 0.009614 | 0.59389 | 0.8698 | 0.82353 | 0.444495 | 0.008990 | 0.63945 | 0.8331 | 0.78947 | 0.413493 | 0.00136 | 0.61839 | 0.7508 | | 0.54902 | 0.363423 | 0.009614 | 0.54627 |
| AMB-01134795 | 0.8431 | 0.8579 | 0.437570 | 0.007139 | 0.58872 | 0.9199 | 0.94118 | 0.579557 | 0.006980 | 0.64170 | 0.7782 | 0.78329 | 0.358291 | 0.00000 | 0.60998 | 0.8167 | | 0.62745 | 0.437028 | 0.007139 | 0.54584 |
| est10931 | 0.7662 | 0.7778 | 0.403395 | 0.006407 | 0.59386 | 0.7221 | 0.64706 | 0.304877 | 0.006680 | 0.63724 | 0.9191 | 0.89474 | 0.519349 | 0.00429 | 0.62124 | 0.7455 | | 0.59649 | 0.364178 | 0.006407 | 0.54751 |
| ahb8068 | 0.7916 | 0.8000 | 0.422810 | 0.001978 | 0.59533 | 0.9574 | 0.93750 | 0.574297 | 0.008110 | 0.64097 | 0.7414 | 0.68421 | 0.330595 | 0.00486 | 0.61719 | 0.7757 | | 0.62500 | 0.404036 | 0.001978 | 0.54846 |
| ahb4723 | 0.7372 | 0.7500 | 0.380396 | 0.018287 | 0.59430 | 0.7221 | 0.64706 | 0.304877 | 0.016780 | 0.63772 | 0.8768 | 0.84211 | 0.462548 | 0.00324 | 0.61970 | 0.7026 | | 0.56141 | 0.331318 | 0.018287 | 0.54667 |
| AMB-01074696 | 0.8158 | 0.8317 | 0.419326 | 0.010095 | 0.58759 | 0.7643 | 0.73530 | 0.332658 | 0.008900 | 0.63039 | 0.9178 | 0.91796 | 0.523436 | 0.00492 | 0.61563 | 0.7886 | | 0.61197 | 0.385306 | 0.010095 | 0.54271 |
| ahb11479 | 0.7953 | 0.8056 | 0.427820 | 0.007401 | 0.59342 | 0.8227 | 0.76471 | 0.392407 | 0.009290 | 0.63815 | 0.8768 | 0.84211 | 0.462548 | 0.00161 | 0.61934 | 0.7530 | | 0.56141 | 0.363357 | 0.007401 | 0.54717 |
| ahb9298 | 0.8827 | 0.8889 | 0.512546 | 0.001168 | 0.59727 | 0.9150 | 0.88235 | 0.505105 | 0.001280 | 0.63998 | 0.9191 | 0.89474 | 0.519349 | 0.00088 | 0.62076 | 0.8540 | | 0.59649 | 0.443085 | 0.001168 | 0.54919 |
| ahb2788 | 0.8775 | 0.8873 | 0.481929 | 0.002634 | 0.59011 | 0.9139 | 0.91177 | 0.514510 | 0.003480 | 0.63584 | 0.8730 | 0.86533 | 0.455674 | 0.00558 | 0.61365 | 0.8485 | | 0.60785 | 0.432220 | 0.002634 | 0.54366 |
| est325 | 0.9085 | 0.9150 | 0.519180 | 0.001866 | 0.59004 | 0.9139 | 0.91177 | 0.514510 | 0.001900 | 0.63475 | 0.9178 | 0.91796 | 0.523436 | 0.00341 | 0.61478 | 0.8853 | | 0.61197 | 0.466275 | 0.001866 | 0.54424 |
| ahb1368 | 0.8523 | 0.8529 | 0.473508 | 0.001891 | 0.59512 | 0.8053 | 0.85294 | 0.473508 | 0.003570 | 0.63846 | 0.8116 | 0.85294 | 0.473508 | 0.00422 | 0.61892 | 0.8174 | | 0.56863 | 0.457790 | 0.001891 | 0.55186 |
| est4995 | 0.4780 | 0.5000 | 0.215762 | 0.056536 | 0.59025 | 0.0439 | 0.05882 | 0.020832 | 0.058230 | 0.63498 | 0.9191 | 0.89474 | 0.519349 | 0.06597 | 0.62164 | 0.8543 | | 0.59649 | 0.438503 | 0.056536 | 0.55058 |
| AMB-00015631 | 0.7284 | 0.7643 | 0.352352 | 0.011121 | 0.58567 | 0.5327 | 0.52679 | 0.190411 | 0.010730 | 0.62315 | 0.9559 | 0.96429 | 0.615471 | 0.00301 | 0.62179 | 0.7607 | | 0.64286 | 0.411205 | 0.011121 | 0.54871 |
| AMB-00287688 | 0.7384 | 0.7708 | 0.345536 | 0.016563 | 0.58550 | 0.5744 | 0.58456 | 0.208835 | 0.015480 | 0.62472 | 0.9196 | 0.93750 | 0.574297 | 0.00572 | 0.61994 | 0.7412 | | 0.62500 | 0.389010 | 0.016563 | 0.54663 |
| est10090 | 0.8459 | 0.8546 | 0.444533 | 0.004786 | 0.58961 | 0.8428 | 0.88235 | 0.505105 | 0.004760 | 0.63961 | 0.7951 | 0.82972 | 0.406831 | 0.00661 | 0.61120 | 0.8109 | | 0.58823 | 0.430956 | 0.004786 | 0.54606 |
| est9190 | 0.8244 | 0.8333 | 0.453913 | 0.003065 | 0.59499 | 0.9150 | 0.88235 | 0.505105 | 0.002330 | 0.64066 | 0.8331 | 0.78947 | 0.413493 | 0.00422 | 0.61886 | 0.7880 | | 0.58823 | 0.394284 | 0.003065 | 0.54776 |
| AMB-00853051 | 0.7082 | 0.7222 | 0.358634 | 0.009973 | 0.59283 | 0.9584 | 0.94118 | 0.579557 | 0.002010 | 0.64093 | 0.5919 | 0.52632 | 0.230453 | 0.00690 | 0.61485 | 0.7370 | | 0.62745 | 0.387752 | 0.009973 | 0.54845 |
| *est6446 | 0.8244 | 0.8333 | 0.453913 | 0.000810 | 0.59525 | 0.7221 | 0.64706 | 0.304877 | 0.000960 | 0.63914 | 1.0000 | 1.00000 | 0.693147 | 0.00060 | 0.65034 | 0.8369 | | 0.66667 | 0.471917 | 0.000810 | 0.56000 |
| *AMB-00731898 | 0.7662 | 0.7778 | 0.403395 | 0.005220 | 0.59489 | 0.9150 | 0.88235 | 0.505105 | 0.004570 | 0.63989 | 0.7414 | 0.68421 | 0.330595 | 0.00925 | 0.61748 | 0.7353 | | 0.58823 | 0.363540 | 0.005220 | 0.54652 |
| ahb2960 | 0.7662 | 0.7778 | 0.403395 | 0.002839 | 0.59356 | 0.9584 | 0.94118 | 0.579557 | 0.003840 | 0.64145 | 0.6932 | 0.63158 | 0.294600 | 0.00698 | 0.61803 | 0.7612 | | 0.62745 | 0.398028 | 0.002839 | 0.54778 |
| AMB-00996152 | 0.7953 | 0.8056 | 0.427820 | 0.003947 | 0.59430 | 0.7735 | 0.70588 | 0.346350 | 0.003160 | 0.63660 | 0.9191 | 0.89474 | 0.519349 | 0.00450 | 0.62158 | 0.7663 | | 0.59649 | 0.376798 | 0.003947 | 0.54808 |
| est4967 | 0.7662 | 0.7778 | 0.403395 | 0.006254 | 0.59344 | 0.7221 | 0.64706 | 0.304877 | 0.007420 | 0.63748 | 0.9191 | 0.89474 | 0.519349 | 0.00220 | 0.62168 | 0.7455 | | 0.59649 | 0.364178 | 0.006254 | 0.54768 |
| ahb12214 | 0.7662 | 0.7778 | 0.403395 | 0.006340 | 0.59352 | 0.7221 | 0.64706 | 0.304877 | 0.005230 | 0.63783 | 0.9191 | 0.89474 | 0.519349 | 0.00312 | 0.62065 | 0.7455 | | 0.59649 | 0.364178 | 0.006340 | 0.54809 |
| *ahb11367 | 0.7293 | 0.7429 | 0.374688 | 0.008790 | 0.59438 | 0.9110 | 0.87500 | 0.496929 | 0.003120 | 0.63930 | 0.6889 | 0.63158 | 0.294600 | 0.01336 | 0.61710 | 0.7042 | | 0.58333 | 0.348178 | 0.008790 | 0.54748 |
| est8113 | 0.8775 | 0.8873 | 0.481929 | 0.002512 | 0.58889 | 0.9139 | 0.91177 | 0.514510 | 0.003400 | 0.63475 | 0.8730 | 0.86533 | 0.455674 | 0.00665 | 0.61323 | 0.8485 | | 0.60785 | 0.432220 | 0.002512 | 0.54348 |
| ahb6614 | 0.8244 | 0.8333 | 0.453913 | 0.001150 | 0.59522 | 0.9584 | 0.94118 | 0.579557 | 0.002270 | 0.64031 | 0.7880 | 0.73684 | 0.369943 | 0.00701 | 0.61739 | 0.8023 | | 0.62745 | 0.419404 | 0.001150 | 0.54796 |
| ahb6126 | 0.7953 | 0.8056 | 0.427820 | 0.003581 | 0.59494 | 0.7735 | 0.70588 | 0.346350 | 0.001760 | 0.63660 | 0.9191 | 0.89474 | 0.519349 | 0.01067 | 0.62086 | 0.7663 | | 0.59649 | 0.376798 | 0.003581 | 0.54732 |
| *ahb12453 | 0.7662 | 0.7778 | 0.403395 | 0.008275 | 0.59411 | 0.7735 | 0.70588 | 0.346350 | 0.011370 | 0.63758 | 0.8768 | 0.84211 | 0.462548 | 0.00260 | 0.61980 | 0.7256 | | 0.56141 | 0.345315 | 0.008275 | 0.54589 |
| *est10009 | 0.8158 | 0.8317 | 0.419326 | 0.010973 | 0.58702 | 0.7643 | 0.73530 | 0.332658 | 0.011680 | 0.63036 | 0.9178 | 0.91796 | 0.523436 | 0.00291 | 0.61442 | 0.7886 | | 0.61197 | 0.385306 | 0.010973 | 0.54259 |
| *ahb722 | 0.8158 | 0.8317 | 0.419326 | 0.007236 | 0.58790 | 0.9139 | 0.91177 | 0.514510 | 0.005400 | 0.63499 | 0.7791 | 0.76006 | 0.352983 | 0.01067 | 0.61143 | 0.7842 | | 0.60785 | 0.387687 | 0.007236 | 0.54275 |
| *est10040 | 0.8754 | 0.8824 | 0.505105 | 0.001350 | 0.59548 | 0.9066 | 0.86667 | 0.487895 | 0.001370 | 0.64006 | 0.9161 | 0.89474 | 0.519349 | 0.00114 | 0.62109 | 0.8455 | | 0.59649 | 0.434828 | 0.001350 | 0.54824 |
| est2430 | 0.6625 | 0.6928 | 0.300212 | 0.009802 | 0.58516 | 0.3975 | 0.38235 | 0.122559 | 0.010080 | 0.62162 | 0.9611 | 0.97059 | 0.626369 | 0.00490 | 0.62215 | 0.7717 | | 0.64706 | 0.422316 | 0.009802 | 0.54856 |
| AMB-01006588 | 0.8428 | 0.8562 | 0.436116 | 0.005215 | 0.58798 | 0.8810 | 0.91176 | 0.540031 | 0.002970 | 0.64041 | 0.7838 | 0.80650 | 0.375536 | 0.00476 | 0.61129 | 0.8107 | | 0.60784 | 0.428732 | 0.005215 | 0.54619 |
| AMB-00906078 | 0.7372 | 0.7500 | 0.380396 | 0.008160 | 0.59307 | 0.7735 | 0.70588 | 0.346350 | 0.009060 | 0.63789 | 0.8331 | 0.78947 | 0.413493 | 0.00921 | 0.61925 | 0.6873 | | 0.52631 | 0.319659 | 0.008160 | 0.54566 |
| est59 | 0.7953 | 0.8056 | 0.427820 | 0.002602 | 0.59432 | 0.7221 | 0.64706 | 0.304877 | 0.001760 | 0.63673 | 0.9602 | 0.94737 | 0.588665 | 0.00438 | 0.62261 | 0.7904 | | 0.63158 | 0.406018 | 0.002602 | 0.54864 |
| ahb4126 | 0.6793 | 0.6944 | 0.337961 | 0.025900 | 0.59167 | 0.6683 | 0.58824 | 0.267050 | 0.028620 | 0.63708 | 0.8331 | 0.78947 | 0.413493 | 0.01503 | 0.61967 | 0.6412 | | 0.52631 | 0.292438 | 0.025900 | 0.54620 |
| est6434 | 0.8535 | 0.8611 | 0.482000 | 0.001765 | 0.59511 | 0.8698 | 0.82353 | 0.444495 | 0.001880 | 0.63885 | 0.9191 | 0.89474 | 0.519349 | 0.00196 | 0.62039 | 0.8209 | | 0.59649 | 0.415024 | 0.001765 | 0.54828 |
| *est8354 | 0.8827 | 0.8889 | 0.512546 | 0.000530 | 0.59464 | 0.9150 | 0.88235 | 0.505105 | 0.000470 | 0.64011 | 0.9191 | 0.89474 | 0.519349 | 0.00067 | 0.62048 | 0.8540 | | 0.59649 | 0.443085 | 0.000530 | 0.54913 |
| est6265 | 0.8145 | 0.8252 | 0.413764 | 0.009070 | 0.58868 | 0.8053 | 0.85294 | 0.473508 | 0.008070 | 0.63763 | 0.7557 | 0.80031 | 0.376800 | 0.00448 | 0.61148 | 0.7740 | | 0.56863 | 0.404508 | 0.009070 | 0.54514 |
| est1128 | 0.8466 | 0.8595 | 0.449044 | 0.004492 | 0.58784 | 0.9139 | 0.91177 | 0.514510 | 0.003970 | 0.63448 | 0.8268 | 0.81270 | 0.400444 | 0.00771 | 0.61217 | 0.8147 | | 0.60785 | 0.407068 | 0.004492 | 0.54204 |
| est5553 | 0.8771 | 0.8840 | 0.478635 | 0.002417 | 0.58932 | 0.8277 | 0.85294 | 0.431638 | 0.002480 | 0.63203 | 0.8852 | 0.91176 | 0.540031 | 0.00268 | 0.61837 | 0.8485 | | 0.60784 | 0.455450 | 0.002417 | 0.54761 |
| *AMB-01065251 | 0.8321 | 0.8381 | 0.426442 | 0.005593 | 0.58913 | 0.7609 | 0.80417 | 0.377403 | 0.007670 | 0.63045 | 0.8347 | 0.86667 | 0.487895 | 0.00365 | 0.61822 | 0.7916 | | 0.57778 | 0.410056 | 0.005593 | 0.54562 |
| ahb5608 | 0.6793 | 0.6944 | 0.337961 | 0.042062 | 0.59290 | 0.8227 | 0.76471 | 0.392407 | 0.020030 | 0.63887 | 0.6932 | 0.63158 | 0.294600 | 0.02524 | 0.61810 | 0.6291 | | 0.50981 | 0.289520 | 0.042062 | 0.54496 |
| *est9328 | 0.8009 | 0.8207 | 0.406503 | 0.010376 | 0.58758 | 0.7990 | 0.78024 | 0.368507 | 0.009890 | 0.63145 | 0.8618 | 0.85663 | 0.444366 | 0.00864 | 0.61337 | 0.7579 | | 0.57109 | 0.356328 | 0.010376 | 0.54082 |
| AMB-00446837 | 0.8466 | 0.8595 | 0.449044 | 0.005548 | 0.58726 | 0.9139 | 0.91177 | 0.514510 | 0.002860 | 0.63520 | 0.8268 | 0.81270 | 0.400444 | 0.00615 | 0.61181 | 0.8147 | | 0.60785 | 0.407068 | 0.005548 | 0.54222 |
| *AMB-00045754 | 0.9083 | 0.9134 | 0.517341 | 0.000119 | 0.59024 | 0.9199 | 0.94118 | 0.579557 | 0.000000 | 0.64064 | 0.8761 | 0.88855 | 0.478173 | 0.00189 | 0.61401 | 0.8861 | | 0.62745 | 0.491771 | 0.000119 | 0.54776 |
| est2423 | 0.6504 | 0.6667 | 0.318257 | 0.015854 | 0.59301 | 0.4910 | 0.41176 | 0.169703 | 0.015490 | 0.63474 | 0.9191 | 0.89474 | 0.519349 | 0.00309 | 0.62148 | 0.7131 | | 0.59649 | 0.346807 | 0.015854 | 0.54782 |
| *est10281 | 0.8466 | 0.8595 | 0.449044 | 0.006570 | 0.58983 | 0.8662 | 0.85294 | 0.441798 | 0.007170 | 0.63239 | 0.8730 | 0.86533 | 0.455674 | 0.00217 | 0.61346 | 0.8107 | | 0.57689 | 0.394991 | 0.006570 | 0.54232 |
| AMB-00301576 | 0.8771 | 0.8840 | 0.478635 | 0.002101 | 0.58947 | 0.8810 | 0.91176 | 0.540031 | 0.002480 | 0.63930 | 0.8353 | 0.85913 | 0.440199 | 0.00306 | 0.61295 | 0.8482 | | 0.60784 | 0.459760 | 0.002101 | 0.54707 |
| ahb12048 | 0.7034 | 0.7059 | 0.346350 | 0.020404 | 0.59248 | 0.6276 | 0.70588 | 0.346350 | 0.014090 | 0.63694 | 0.6375 | 0.70588 | 0.346350 | 0.00983 | 0.61829 | 0.6461 | | 0.47059 | 0.343662 | 0.020404 | 0.54983 |
| est5107 | 0.8775 | 0.8873 | 0.481929 | 0.000678 | 0.58919 | 0.9596 | 0.97059 | 0.626369 | 0.000000 | 0.64206 | 0.8268 | 0.81270 | 0.400444 | 0.00328 | 0.61204 | 0.8575 | | 0.64706 | 0.472536 | 0.000678 | 0.54698 |
| *est10426 | 0.7782 | 0.8023 | 0.376609 | 0.009014 | 0.58564 | 0.6493 | 0.64706 | 0.250446 | 0.008300 | 0.62539 | 0.9228 | 0.94118 | 0.579557 | 0.00188 | 0.62094 | 0.7688 | | 0.62745 | 0.400989 | 0.009014 | 0.54689 |
| ahb2647 | 0.8244 | 0.8333 | 0.453913 | 0.002878 | 0.59500 | 0.9150 | 0.88235 | 0.505105 | 0.002870 | 0.63992 | 0.8331 | 0.78947 | 0.413493 | 0.00170 | 0.61879 | 0.7880 | | 0.58823 | 0.394284 | 0.002878 | 0.54779 |
| ahb3343 | 0.8459 | 0.8546 | 0.444533 | 0.004220 | 0.58820 | 0.8428 | 0.88235 | 0.505105 | 0.003850 | 0.63888 | 0.7951 | 0.82972 | 0.406831 | 0.00478 | 0.61193 | 0.8109 | | 0.58823 | 0.430956 | 0.004220 | 0.54631 |
| ahb1990 | 0.8377 | 0.8520 | 0.430743 | 0.003664 | 0.58820 | 0.8762 | 0.90909 | 0.536691 | 0.005230 | 0.63917 | 0.7795 | 0.80383 | 0.372478 | 0.00530 | 0.60953 | 0.8047 | | 0.60606 | 0.426069 | 0.003664 | 0.54520 |
| AMB-00053302 | 0.8775 | 0.8873 | 0.481929 | 0.002108 | 0.59027 | 0.8662 | 0.85294 | 0.441798 | 0.003530 | 0.63317 | 0.9178 | 0.91796 | 0.523436 | 0.00370 | 0.61398 | 0.8493 | | 0.61197 | 0.430507 | 0.002108 | 0.54315 |
| ahb505 | 0.8244 | 0.8333 | 0.453913 | 0.001139 | 0.59484 | 0.7735 | 0.70588 | 0.346350 | 0.000780 | 0.63834 | 0.9602 | 0.94737 | 0.588665 | 0.00232 | 0.62208 | 0.8093 | | 0.63158 | 0.417256 | 0.001139 | 0.55018 |
| est73 | 0.8441 | 0.8563 | 0.445504 | 0.005060 | 0.58797 | 0.9117 | 0.90809 | 0.509358 | 0.005010 | 0.63535 | 0.8268 | 0.81270 | 0.400444 | 0.00387 | 0.61194 | 0.8123 | | 0.60539 | 0.404226 | 0.005060 | 0.54199 |
| est7916 | 0.8775 | 0.8873 | 0.481929 | 0.000830 | 0.58905 | 0.9596 | 0.97059 | 0.626369 | 0.000670 | 0.64224 | 0.8268 | 0.81270 | 0.400444 | 0.00096 | 0.61299 | 0.8575 | | 0.64706 | 0.472536 | 0.000830 | 0.54736 |
| AMB-00488728 | 0.8428 | 0.8562 | 0.436116 | 0.003712 | 0.58715 | 0.8810 | 0.91176 | 0.540031 | 0.002480 | 0.63940 | 0.7838 | 0.80650 | 0.375536 | 0.01046 | 0.61024 | 0.8107 | | 0.60784 | 0.428732 | 0.003712 | 0.54503 |
| est9333 | 0.8431 | 0.8579 | 0.437570 | 0.002266 | 0.58788 | 0.9199 | 0.94118 | 0.579557 | 0.002510 | 0.64208 | 0.7782 | 0.78329 | 0.358291 | 0.00524 | 0.61037 | 0.8167 | | 0.62745 | 0.437028 | 0.002266 | 0.54659 |
| AMB-00957883 | 0.8775 | 0.8873 | 0.481929 | 0.002583 | 0.58893 | 0.9139 | 0.91177 | 0.514510 | 0.002100 | 0.63540 | 0.8730 | 0.86533 | 0.455674 | 0.00428 | 0.61279 | 0.8485 | | 0.60785 | 0.432220 | 0.002583 | 0.54257 |
| AMB-01026557 | 0.8761 | 0.8706 | 0.461767 | 0.001848 | 0.58916 | 0.8113 | 0.83750 | 0.413007 | 0.001920 | 0.63167 | 0.8895 | 0.90000 | 0.525597 | 0.00490 | 0.62098 | 0.8311 | | 0.60000 | 0.441204 | 0.001848 | 0.54607 |
| AMB-00771202 | 0.8820 | 0.8824 | 0.505105 | 0.000628 | 0.59623 | 0.8428 | 0.88235 | 0.505105 | 0.000550 | 0.63871 | 0.8481 | 0.88235 | 0.505105 | 0.00063 | 0.61996 | 0.8530 | | 0.58823 | 0.485061 | 0.000628 | 0.55303 |
| AMB-00445003 | 0.8621 | 0.8519 | 0.472391 | 0.000675 | 0.59523 | 0.8615 | 0.80000 | 0.422810 | 0.001060 | 0.63905 | 0.9526 | 0.91667 | 0.546253 | 0.00059 | 0.62103 | 0.8411 | | 0.61111 | 0.420266 | 0.000675 | 0.54856 |
| est11466 | 0.7543 | 0.7762 | 0.366819 | 0.005429 | 0.58597 | 0.9596 | 0.97059 | 0.626369 | 0.001740 | 0.64140 | 0.6260 | 0.60217 | 0.239128 | 0.00797 | 0.60833 | 0.7652 | | 0.64706 | 0.423776 | 0.005429 | 0.54755 |
| ahb3698 | 0.8106 | 0.8301 | 0.405504 | 0.007768 | 0.58728 | 0.8703 | 0.88236 | 0.469429 | 0.006330 | 0.63312 | 0.7782 | 0.78329 | 0.358291 | 0.01379 | 0.61073 | 0.7724 | | 0.58824 | 0.372376 | 0.007768 | 0.54032 |
| *ahb4134 | 0.7850 | 0.8039 | 0.392072 | 0.010538 | 0.58722 | 0.7099 | 0.67647 | 0.288447 | 0.010030 | 0.62770 | 0.9178 | 0.91796 | 0.523436 | 0.00291 | 0.61506 | 0.7644 | | 0.61197 | 0.370821 | 0.010538 | 0.54237 |
| *ahb6424 | 0.7904 | 0.7941 | 0.417576 | 0.007613 | 0.59463 | 0.7280 | 0.79412 | 0.417576 | 0.003480 | 0.63670 | 0.7404 | 0.79412 | 0.417576 | 0.00086 | 0.61812 | 0.7459 | | 0.52941 | 0.408439 | 0.007613 | 0.55056 |
| AMB-01135894 | 0.8106 | 0.8301 | 0.405504 | 0.003714 | 0.58718 | 0.9199 | 0.94118 | 0.579557 | 0.002630 | 0.64032 | 0.7268 | 0.73065 | 0.312410 | 0.00755 | 0.60934 | 0.7871 | | 0.62745 | 0.419818 | 0.003714 | 0.54649 |
| ahb3288 | 0.7662 | 0.7778 | 0.403395 | 0.004004 | 0.59451 | 0.8698 | 0.82353 | 0.444495 | 0.005870 | 0.63893 | 0.7880 | 0.73684 | 0.369943 | 0.00531 | 0.61785 | 0.7204 | | 0.54902 | 0.344893 | 0.004004 | 0.54633 |
| est9672 | 0.7372 | 0.7500 | 0.380396 | 0.005749 | 0.59362 | 0.6683 | 0.58824 | 0.267050 | 0.006180 | 0.63539 | 0.9191 | 0.89474 | 0.519349 | 0.00178 | 0.62061 | 0.7294 | | 0.59649 | 0.355086 | 0.005749 | 0.54789 |
| ahb3056 | 0.8158 | 0.8317 | 0.419326 | 0.005055 | 0.58772 | 0.7643 | 0.73530 | 0.332658 | 0.006120 | 0.62994 | 0.9178 | 0.91796 | 0.523436 | 0.00451 | 0.61481 | 0.7886 | | 0.61197 | 0.385306 | 0.005055 | 0.54259 |
| *est2086 | 0.8466 | 0.8595 | 0.449044 | 0.003460 | 0.58856 | 0.8662 | 0.85294 | 0.441798 | 0.005580 | 0.63345 | 0.8730 | 0.86533 | 0.455674 | 0.00572 | 0.61297 | 0.8107 | | 0.57689 | 0.394991 | 0.003460 | 0.54140 |
| ahb2561 | 0.7372 | 0.7500 | 0.380396 | 0.001771 | 0.59407 | 1.0000 | 1.00000 | 0.693147 | 0.001760 | 0.66227 | 0.5919 | 0.52632 | 0.230453 | 0.00170 | 0.61512 | 0.7893 | | 0.66667 | 0.462406 | 0.001771 | 0.55935 |
| ahb7694 | 0.8431 | 0.8579 | 0.437570 | 0.002112 | 0.58896 | 0.9199 | 0.94118 | 0.579557 | 0.004480 | 0.64003 | 0.7782 | 0.78329 | 0.358291 | 0.00280 | 0.61068 | 0.8167 | | 0.62745 | 0.437028 | 0.002112 | 0.54600 |
| est10232 | 0.5928 | 0.6111 | 0.281374 | 0.014783 | 0.59155 | 0.3575 | 0.29412 | 0.114674 | 0.009710 | 0.63510 | 0.9191 | 0.89474 | 0.519349 | 0.00351 | 0.62139 | 0.7325 | | 0.59649 | 0.357379 | 0.014783 | 0.54753 |
| AMB-01095187 | 0.8099 | 0.8268 | 0.402833 | 0.009417 | 0.58594 | 0.7860 | 0.82353 | 0.398452 | 0.011350 | 0.63192 | 0.7951 | 0.82972 | 0.406831 | 0.00915 | 0.61054 | 0.7674 | | 0.55315 | 0.371034 | 0.009417 | 0.54051 |
| est3025 | 0.8804 | 0.8824 | 0.505105 | 0.000075 | 0.59524 | 0.8400 | 0.88235 | 0.505105 | 0.000000 | 0.63837 | 0.8481 | 0.88235 | 0.505105 | 0.00084 | 0.62062 | 0.8519 | | 0.58823 | 0.485061 | 0.000075 | 0.55172 |
| AMB-01129981 | 0.7662 | 0.7778 | 0.403395 | 0.004256 | 0.59338 | 0.7735 | 0.70588 | 0.346350 | 0.003660 | 0.63760 | 0.8768 | 0.84211 | 0.462548 | 0.00551 | 0.62024 | 0.7256 | | 0.56141 | 0.345315 | 0.004256 | 0.54613 |
| *AMB-00906075 | 0.7082 | 0.7222 | 0.358634 | 0.009381 | 0.59297 | 0.7735 | 0.70588 | 0.346350 | 0.009060 | 0.63860 | 0.7880 | 0.73684 | 0.369943 | 0.00738 | 0.61801 | 0.6519 | | 0.49123 | 0.298374 | 0.009381 | 0.54496 |
| ahb3639 | 0.8757 | 0.8856 | 0.474003 | 0.002504 | 0.58933 | 0.8703 | 0.88236 | 0.469429 | 0.004350 | 0.63283 | 0.8761 | 0.88855 | 0.478173 | 0.00262 | 0.61279 | 0.8454 | | 0.59237 | 0.430112 | 0.002504 | 0.54313 |
| AMB-01053857 | 0.7662 | 0.7778 | 0.403395 | 0.004097 | 0.59442 | 0.7221 | 0.64706 | 0.304877 | 0.003550 | 0.63628 | 0.9191 | 0.89474 | 0.519349 | 0.00264 | 0.61994 | 0.7455 | | 0.59649 | 0.364178 | 0.004097 | 0.54791 |
| ahb9245 | 0.8459 | 0.8546 | 0.444533 | 0.002718 | 0.58808 | 0.8428 | 0.88235 | 0.505105 | 0.003460 | 0.63961 | 0.7951 | 0.82972 | 0.406831 | 0.00452 | 0.61203 | 0.8109 | | 0.58823 | 0.430956 | 0.002718 | 0.54706 |
| *est9648 | 0.8086 | 0.8284 | 0.400499 | 0.008951 | 0.58657 | 0.7722 | 0.79411 | 0.362391 | 0.012050 | 0.62963 | 0.8353 | 0.85913 | 0.440199 | 0.00482 | 0.61288 | 0.7677 | | 0.57275 | 0.364863 | 0.008951 | 0.54056 |
| AMB-01069532 | 0.7212 | 0.7667 | 0.394036 | 0.006109 | 0.59291 | 0.8543 | 0.81818 | 0.439456 | 0.006930 | 0.63767 | 0.7279 | 0.73684 | 0.369943 | 0.00483 | 0.61781 | 0.6523 | | 0.54545 | 0.342316 | 0.006109 | 0.54533 |
| AMB-00519911 | 0.7166 | 0.7465 | 0.340416 | 0.004765 | 0.58457 | 0.9578 | 0.96875 | 0.623128 | 0.004630 | 0.64179 | 0.5595 | 0.54770 | 0.205278 | 0.00535 | 0.60575 | 0.7439 | | 0.64583 | 0.417216 | 0.004765 | 0.54791 |
| ahb8930 | 0.7703 | 0.7955 | 0.364345 | 0.088300 | 0.58581 | 0.7214 | 0.76114 | 0.327369 | 0.093350 | 0.62792 | 0.7909 | 0.82616 | 0.403031 | 0.09635 | 0.61222 | 0.7222 | | 0.55077 | 0.334675 | 0.088300 | 0.53827 |
| *ahb11528 | 0.6561 | 0.6849 | 0.294452 | 0.012445 | 0.58401 | 0.3595 | 0.34559 | 0.106318 | 0.005080 | 0.62082 | 0.9611 | 0.97059 | 0.626369 | 0.00177 | 0.62136 | 0.7855 | | 0.64706 | 0.426364 | 0.012445 | 0.54895 |
| AMB-00307538 | 0.8466 | 0.8595 | 0.449044 | 0.003992 | 0.58874 | 0.8662 | 0.85294 | 0.441798 | 0.004280 | 0.63295 | 0.8730 | 0.86533 | 0.455674 | 0.00370 | 0.61340 | 0.8107 | | 0.57689 | 0.394991 | 0.003992 | 0.54105 |
| ahb5317 | 0.7782 | 0.8023 | 0.376609 | 0.009856 | 0.58660 | 0.8703 | 0.88236 | 0.469429 | 0.011880 | 0.63260 | 0.7268 | 0.73065 | 0.312410 | 0.00703 | 0.60843 | 0.7409 | | 0.58824 | 0.353712 | 0.009856 | 0.54044 |
| *est6757 | 0.8775 | 0.8873 | 0.481929 | 0.001864 | 0.58923 | 0.8662 | 0.85294 | 0.441798 | 0.001920 | 0.63331 | 0.9178 | 0.91796 | 0.523436 | 0.00198 | 0.61552 | 0.8493 | | 0.61197 | 0.430507 | 0.001864 | 0.54387 |
| *est11180 | 0.7543 | 0.7762 | 0.366819 | 0.009099 | 0.58692 | 0.6530 | 0.61765 | 0.248888 | 0.013720 | 0.62809 | 0.9178 | 0.91796 | 0.523436 | 0.00315 | 0.61489 | 0.7448 | | 0.61197 | 0.360358 | 0.009099 | 0.54237 |
| ahb3844 | 0.6504 | 0.6667 | 0.318257 | 0.013901 | 0.59265 | 0.5530 | 0.47059 | 0.199887 | 0.010840 | 0.63600 | 0.8768 | 0.84211 | 0.462548 | 0.00344 | 0.61970 | 0.6638 | | 0.56141 | 0.309471 | 0.013901 | 0.54608 |
| *AMB-01076641 | 0.7441 | 0.7680 | 0.345540 | 0.010431 | 0.58533 | 0.7684 | 0.82353 | 0.444495 | 0.012110 | 0.63801 | 0.6568 | 0.71827 | 0.289362 | 0.04038 | 0.60755 | 0.6969 | | 0.54902 | 0.351280 | 0.010431 | 0.54479 |
| est232 | 0.8145 | 0.8252 | 0.413764 | 0.003442 | 0.58848 | 0.7451 | 0.79412 | 0.368604 | 0.004930 | 0.63049 | 0.8116 | 0.85294 | 0.473508 | 0.00621 | 0.61877 | 0.7743 | | 0.56863 | 0.400387 | 0.003442 | 0.54604 |
| est10035 | 0.8145 | 0.8252 | 0.413764 | 0.004082 | 0.58889 | 0.8053 | 0.85294 | 0.473508 | 0.004370 | 0.63828 | 0.7557 | 0.80031 | 0.376800 | 0.00610 | 0.61036 | 0.7740 | | 0.56863 | 0.404508 | 0.004082 | 0.54519 |
| *est830 | 0.7689 | 0.7831 | 0.372964 | 0.017707 | 0.58619 | 0.8662 | 0.85294 | 0.441798 | 0.006940 | 0.63278 | 0.7459 | 0.70392 | 0.308429 | 0.02209 | 0.60983 | 0.7340 | | 0.56863 | 0.330189 | 0.017707 | 0.53951 |
| *est11355 | 0.6504 | 0.6667 | 0.318257 | 0.009299 | 0.59205 | 0.8698 | 0.82353 | 0.444495 | 0.010420 | 0.63948 | 0.5919 | 0.52632 | 0.230453 | 0.00639 | 0.61662 | 0.6371 | | 0.54902 | 0.302208 | 0.009299 | 0.54590 |
| AMB-00381986 | 0.8406 | 0.8561 | 0.435155 | 0.002640 | 0.58723 | 0.9182 | 0.93939 | 0.576994 | 0.002050 | 0.64034 | 0.7740 | 0.78150 | 0.356010 | 0.00314 | 0.60896 | 0.8130 | | 0.62626 | 0.435064 | 0.002640 | 0.54649 |
| *AMB-00698311 | 0.8757 | 0.8856 | 0.474003 | 0.002247 | 0.58793 | 0.8703 | 0.88236 | 0.469429 | 0.002950 | 0.63349 | 0.8761 | 0.88855 | 0.478173 | 0.00307 | 0.61304 | 0.8454 | | 0.59237 | 0.430112 | 0.002247 | 0.54216 |
| *AMB-01129976 | 0.6504 | 0.6667 | 0.318257 | 0.012019 | 0.59223 | 0.6120 | 0.52941 | 0.232214 | 0.010920 | 0.63554 | 0.8331 | 0.78947 | 0.413493 | 0.00838 | 0.61950 | 0.6258 | | 0.52631 | 0.283817 | 0.012019 | 0.54557 |
| ahb1468 | 0.7662 | 0.7778 | 0.403395 | 0.004189 | 0.59444 | 0.8227 | 0.76471 | 0.392407 | 0.004090 | 0.63788 | 0.8331 | 0.78947 | 0.413493 | 0.00379 | 0.61859 | 0.7171 | | 0.52631 | 0.339079 | 0.004189 | 0.54630 |
| est8691 | 0.7832 | 0.7958 | 0.385586 | 0.007081 | 0.58786 | 0.7051 | 0.76471 | 0.341352 | 0.004520 | 0.62878 | 0.7757 | 0.82353 | 0.444495 | 0.00892 | 0.61865 | 0.7379 | | 0.54902 | 0.375892 | 0.007081 | 0.54542 |
| *ahb11366 | 0.5928 | 0.6111 | 0.281374 | 0.012585 | 0.59140 | 0.9150 | 0.88235 | 0.505105 | 0.004990 | 0.63980 | 0.4259 | 0.36842 | 0.148663 | 0.00736 | 0.61430 | 0.6811 | | 0.58823 | 0.339173 | 0.012585 | 0.54673 |
| *ahb8287 | 0.8739 | 0.8854 | 0.478842 | 0.002647 | 0.58926 | 0.9103 | 0.90993 | 0.511378 | 0.001280 | 0.63401 | 0.8681 | 0.86349 | 0.452627 | 0.00200 | 0.61277 | 0.8423 | | 0.60662 | 0.429792 | 0.002647 | 0.54291 |
| *ahb4357 | 0.7372 | 0.7500 | 0.380396 | 0.004951 | 0.59493 | 0.7221 | 0.64706 | 0.304877 | 0.005350 | 0.63673 | 0.8768 | 0.84211 | 0.462548 | 0.00256 | 0.61997 | 0.7026 | | 0.56141 | 0.331318 | 0.004951 | 0.54634 |
| *est6415 | 0.7662 | 0.7778 | 0.403395 | 0.003339 | 0.59442 | 0.7735 | 0.70588 | 0.346350 | 0.004050 | 0.63698 | 0.8768 | 0.84211 | 0.462548 | 0.00370 | 0.61923 | 0.7256 | | 0.56141 | 0.345315 | 0.003339 | 0.54604 |
| AMB-00168369 | 0.8428 | 0.8562 | 0.436116 | 0.004371 | 0.58750 | 0.8277 | 0.85294 | 0.431638 | 0.004100 | 0.63215 | 0.8353 | 0.85913 | 0.440199 | 0.00393 | 0.61308 | 0.8061 | | 0.57275 | 0.398963 | 0.004371 | 0.54187 |
| AMB-00815487 | 0.8099 | 0.8268 | 0.402833 | 0.006510 | 0.58792 | 0.7860 | 0.82353 | 0.398452 | 0.006370 | 0.63121 | 0.7951 | 0.82972 | 0.406831 | 0.00749 | 0.61126 | 0.7674 | | 0.55315 | 0.371034 | 0.006510 | 0.54108 |
| AMB-01091493 | 0.6215 | 0.6389 | 0.299423 | 0.014064 | 0.59224 | 0.5530 | 0.47059 | 0.199887 | 0.012880 | 0.63491 | 0.8331 | 0.78947 | 0.413493 | 0.00876 | 0.62075 | 0.6162 | | 0.52631 | 0.278276 | 0.014064 | 0.54506 |
| ahb4072 | 0.7639 | 0.7926 | 0.365409 | 0.020788 | 0.58498 | 0.7419 | 0.74798 | 0.324668 | 0.022960 | 0.62813 | 0.8174 | 0.83022 | 0.404460 | 0.01036 | 0.60971 | 0.7144 | | 0.55348 | 0.323882 | 0.020788 | 0.53828 |
| est1311 | 0.8158 | 0.8317 | 0.419326 | 0.004423 | 0.58754 | 0.8662 | 0.85294 | 0.441798 | 0.005510 | 0.63244 | 0.8268 | 0.81270 | 0.400444 | 0.01011 | 0.61149 | 0.7750 | | 0.56863 | 0.368401 | 0.004423 | 0.54079 |
| ahb5784 | 0.8158 | 0.8317 | 0.419326 | 0.008157 | 0.58795 | 0.8164 | 0.79412 | 0.382949 | 0.005130 | 0.63078 | 0.8730 | 0.86533 | 0.455674 | 0.00143 | 0.61382 | 0.7764 | | 0.57689 | 0.367724 | 0.008157 | 0.54106 |
| AMB-01099369 | 0.7034 | 0.7059 | 0.346350 | 0.009599 | 0.59309 | 0.6276 | 0.70588 | 0.346350 | 0.002710 | 0.63746 | 0.6375 | 0.70588 | 0.346350 | 0.01105 | 0.61623 | 0.6461 | | 0.47059 | 0.343662 | 0.009599 | 0.54885 |
| ahb176 | 0.8106 | 0.8301 | 0.405504 | 0.006230 | 0.58774 | 0.7646 | 0.76471 | 0.341352 | 0.006910 | 0.62861 | 0.8761 | 0.88855 | 0.478173 | 0.00220 | 0.61425 | 0.7748 | | 0.59237 | 0.370153 | 0.006230 | 0.54057 |
| AMB-01046082 | 0.7953 | 0.8056 | 0.427820 | 0.001939 | 0.59443 | 0.7735 | 0.70588 | 0.346350 | 0.001760 | 0.63740 | 0.9191 | 0.89474 | 0.519349 | 0.00228 | 0.62066 | 0.7663 | | 0.59649 | 0.376798 | 0.001939 | 0.54760 |
| est4928 | 0.7928 | 0.8056 | 0.427820 | 0.002193 | 0.59353 | 0.8196 | 0.76471 | 0.392407 | 0.002120 | 0.63761 | 0.8746 | 0.84211 | 0.462548 | 0.00238 | 0.62134 | 0.7490 | | 0.56141 | 0.363357 | 0.002193 | 0.54727 |
| AMB-01045067 | 0.7136 | 0.7467 | 0.325761 | 0.017920 | 0.58427 | 0.8703 | 0.88236 | 0.469429 | 0.013190 | 0.63239 | 0.6190 | 0.62539 | 0.236072 | 0.01640 | 0.60543 | 0.6892 | | 0.58824 | 0.328557 | 0.017920 | 0.53928 |
| AMB-00704104 | 0.8158 | 0.8317 | 0.419326 | 0.004208 | 0.58827 | 0.8662 | 0.85294 | 0.441798 | 0.005030 | 0.63379 | 0.8268 | 0.81270 | 0.400444 | 0.00630 | 0.61243 | 0.7750 | | 0.56863 | 0.368401 | 0.004208 | 0.53994 |
| ahb12370 | 0.7518 | 0.7663 | 0.359511 | 0.008011 | 0.58749 | 0.7323 | 0.79412 | 0.417576 | 0.007520 | 0.63782 | 0.6789 | 0.74149 | 0.324037 | 0.01005 | 0.60954 | 0.7018 | | 0.52941 | 0.356852 | 0.008011 | 0.54487 |
| ahb5010 | 0.7372 | 0.7500 | 0.380396 | 0.004499 | 0.59309 | 0.6683 | 0.58824 | 0.267050 | 0.001510 | 0.63646 | 0.9191 | 0.89474 | 0.519349 | 0.00253 | 0.62144 | 0.7294 | | 0.59649 | 0.355086 | 0.004499 | 0.54736 |
| est6236 | 0.8535 | 0.8611 | 0.482000 | 0.000000 | 0.59626 | 0.9150 | 0.88235 | 0.505105 | 0.000000 | 0.64009 | 0.8768 | 0.84211 | 0.462548 | 0.00000 | 0.61958 | 0.8195 | | 0.58823 | 0.415785 | 0.000000 | 0.54689 |
| AMB-00061630 | 0.6697 | 0.6857 | 0.331669 | 0.012423 | 0.59239 | 0.7610 | 0.68750 | 0.332948 | 0.009170 | 0.63776 | 0.7375 | 0.68421 | 0.330595 | 0.00637 | 0.61692 | 0.6090 | | 0.45833 | 0.274572 | 0.012423 | 0.54442 |
| AMB-00816775 | 0.7953 | 0.8056 | 0.427820 | 0.001400 | 0.59463 | 0.7735 | 0.70588 | 0.346350 | 0.001460 | 0.63785 | 0.9191 | 0.89474 | 0.519349 | 0.00235 | 0.62184 | 0.7663 | | 0.59649 | 0.376798 | 0.001400 | 0.54837 |
| AMB-00923649 | 0.6103 | 0.6286 | 0.292631 | 0.002913 | 0.59161 | 0.9565 | 0.93750 | 0.574297 | 0.008330 | 0.64236 | 0.4207 | 0.36842 | 0.148663 | 0.00376 | 0.61401 | 0.7257 | | 0.62500 | 0.387453 | 0.002913 | 0.54859 |
| AMB-00783213 | 0.7742 | 0.7990 | 0.368035 | 0.005343 | 0.58566 | 0.6656 | 0.70588 | 0.277311 | 0.007160 | 0.62664 | 0.8481 | 0.88235 | 0.505105 | 0.00429 | 0.61945 | 0.7400 | | 0.58823 | 0.373178 | 0.005343 | 0.54552 |
| ahb9340 | 0.7082 | 0.7222 | 0.358634 | 0.005513 | 0.59338 | 0.7735 | 0.70588 | 0.346350 | 0.006110 | 0.63717 | 0.7880 | 0.73684 | 0.369943 | 0.00767 | 0.61810 | 0.6519 | | 0.49123 | 0.298374 | 0.005513 | 0.54556 |
| *est9119 | 0.8158 | 0.8317 | 0.419326 | 0.004759 | 0.58848 | 0.8662 | 0.85294 | 0.441798 | 0.004280 | 0.63221 | 0.8268 | 0.81270 | 0.400444 | 0.00644 | 0.61192 | 0.7750 | | 0.56863 | 0.368401 | 0.004759 | 0.54035 |
| ahb3202 | 0.6215 | 0.6389 | 0.299423 | 0.010431 | 0.59203 | 0.4910 | 0.41176 | 0.169703 | 0.008530 | 0.63408 | 0.8768 | 0.84211 | 0.462548 | 0.00454 | 0.62022 | 0.6624 | | 0.56141 | 0.308405 | 0.010431 | 0.54630 |
| est9898 | 0.7069 | 0.7451 | 0.315570 | 0.006941 | 0.58379 | 0.8810 | 0.91176 | 0.540031 | 0.003410 | 0.64044 | 0.5620 | 0.59597 | 0.205983 | 0.01697 | 0.60322 | 0.6974 | | 0.60784 | 0.369197 | 0.006941 | 0.54637 |
| AMB-00936123 | 0.7082 | 0.7222 | 0.358634 | 0.002391 | 0.59248 | 0.9150 | 0.88235 | 0.505105 | 0.006210 | 0.64107 | 0.6434 | 0.57895 | 0.261365 | 0.00647 | 0.61743 | 0.6981 | | 0.58823 | 0.345201 | 0.002391 | 0.54550 |
| ahb1387 | 0.8431 | 0.8579 | 0.437570 | 0.003066 | 0.58715 | 0.8186 | 0.82353 | 0.398452 | 0.005980 | 0.63196 | 0.8761 | 0.88855 | 0.478173 | 0.00184 | 0.61369 | 0.8082 | | 0.59237 | 0.395173 | 0.003066 | 0.54243 |
| AMB-00433966 | 0.8367 | 0.8511 | 0.438775 | 0.000314 | 0.58930 | 0.9570 | 0.96875 | 0.623128 | 0.000000 | 0.64196 | 0.7639 | 0.74653 | 0.340416 | 0.00292 | 0.60906 | 0.8191 | | 0.64583 | 0.448850 | 0.000314 | 0.54727 |
| *est8750 | 0.6504 | 0.6667 | 0.318257 | 0.006659 | 0.59270 | 0.8698 | 0.82353 | 0.444495 | 0.005120 | 0.63901 | 0.5919 | 0.52632 | 0.230453 | 0.01348 | 0.61618 | 0.6371 | | 0.54902 | 0.302208 | 0.006659 | 0.54483 |
| AMB-00764110 | 0.8106 | 0.8301 | 0.405504 | 0.005462 | 0.58749 | 0.8186 | 0.82353 | 0.398452 | 0.007440 | 0.63196 | 0.8279 | 0.83592 | 0.411962 | 0.00472 | 0.61129 | 0.7682 | | 0.55728 | 0.360383 | 0.005462 | 0.53952 |
| est10259 | 0.8106 | 0.8301 | 0.405504 | 0.005081 | 0.58599 | 0.8186 | 0.82353 | 0.398452 | 0.006370 | 0.63223 | 0.8279 | 0.83592 | 0.411962 | 0.00602 | 0.61230 | 0.7682 | | 0.55728 | 0.360383 | 0.005081 | 0.53967 |
| *est11384 | 0.7082 | 0.7222 | 0.358634 | 0.003697 | 0.59297 | 0.6683 | 0.58824 | 0.267050 | 0.007100 | 0.63577 | 0.8768 | 0.84211 | 0.462548 | 0.00438 | 0.61958 | 0.6843 | | 0.56141 | 0.320850 | 0.003697 | 0.54616 |
| AMB-00789562 | 0.6504 | 0.6667 | 0.318257 | 0.008404 | 0.59180 | 0.8227 | 0.76471 | 0.392407 | 0.008860 | 0.63860 | 0.6434 | 0.57895 | 0.261365 | 0.00780 | 0.61559 | 0.6075 | | 0.50981 | 0.278964 | 0.008404 | 0.54386 |
| AMB-00695365 | 0.6014 | 0.6061 | 0.278171 | 0.138684 | 0.59213 | 0.5120 | 0.60606 | 0.278171 | 0.168940 | 0.63621 | 0.5292 | 0.60606 | 0.278171 | 0.01176 | 0.61600 | 0.5355 | | 0.40404 | 0.279698 | 0.138684 | 0.54828 |
| est8349 | 0.7082 | 0.7222 | 0.358634 | 0.002947 | 0.59264 | 0.6120 | 0.52941 | 0.232214 | 0.001950 | 0.63589 | 0.9191 | 0.89474 | 0.519349 | 0.00626 | 0.62060 | 0.7184 | | 0.59649 | 0.349231 | 0.002947 | 0.54738 |
| *ahb744 | 0.6215 | 0.6389 | 0.299423 | 0.014163 | 0.59233 | 0.6120 | 0.52941 | 0.232214 | 0.008740 | 0.63653 | 0.7880 | 0.73684 | 0.369943 | 0.00946 | 0.61778 | 0.5824 | | 0.49123 | 0.258373 | 0.014163 | 0.54435 |
| AMB-00687520 | 0.5640 | 0.5833 | 0.264041 | 0.013043 | 0.59149 | 0.8698 | 0.82353 | 0.444495 | 0.007780 | 0.63875 | 0.4259 | 0.36842 | 0.148663 | 0.01309 | 0.61392 | 0.6258 | | 0.54902 | 0.297206 | 0.013043 | 0.54473 |
| AMB-00856367 | 0.7670 | 0.7941 | 0.362391 | 0.004482 | 0.58509 | 0.6547 | 0.69485 | 0.268311 | 0.004590 | 0.62599 | 0.8455 | 0.88235 | 0.505105 | 0.00468 | 0.62011 | 0.7354 | | 0.58823 | 0.369851 | 0.004482 | 0.54688 |
| AMB-00620427 | 0.8086 | 0.8284 | 0.400499 | 0.005185 | 0.58665 | 0.7722 | 0.79411 | 0.362391 | 0.007420 | 0.62931 | 0.8353 | 0.85913 | 0.440199 | 0.00378 | 0.61308 | 0.7677 | | 0.57275 | 0.364863 | 0.005185 | 0.54024 |
| AMB-00315874 | 0.7082 | 0.7222 | 0.358634 | 0.002073 | 0.59364 | 0.9584 | 0.94118 | 0.579557 | 0.003250 | 0.64165 | 0.5919 | 0.52632 | 0.230453 | 0.00170 | 0.61537 | 0.7370 | | 0.62745 | 0.387752 | 0.002073 | 0.54873 |
| *est3873 | 0.8431 | 0.8579 | 0.437570 | 0.002623 | 0.58840 | 0.8703 | 0.88236 | 0.469429 | 0.003060 | 0.63394 | 0.8279 | 0.83592 | 0.411962 | 0.00317 | 0.61083 | 0.8073 | | 0.58824 | 0.396799 | 0.002623 | 0.54128 |
| AMB-00800015 | 0.7543 | 0.7762 | 0.366819 | 0.015529 | 0.58670 | 0.7643 | 0.73530 | 0.332658 | 0.015250 | 0.62921 | 0.8268 | 0.81270 | 0.400444 | 0.00344 | 0.61265 | 0.7061 | | 0.54180 | 0.317535 | 0.015529 | 0.53932 |
| ahb7875est4723 | 0.8775 | 0.8873 | 0.481929 | 0.000909 | 0.58929 | 0.8662 | 0.85294 | 0.441798 | 0.000440 | 0.63236 | 0.9178 | 0.91796 | 0.523436 | 0.00121 | 0.61509 | 0.8493 | | 0.61197 | 0.430507 | 0.000909 | 0.54317 |
| ahb7770 | 0.7518 | 0.7663 | 0.359511 | 0.005888 | 0.58681 | 0.6660 | 0.73530 | 0.316211 | 0.006930 | 0.62905 | 0.7404 | 0.79412 | 0.417576 | 0.00811 | 0.61844 | 0.7021 | | 0.52941 | 0.352926 | 0.005888 | 0.54475 |
| *AMB-00717792 | 0.8195 | 0.8182 | 0.439456 | 0.001771 | 0.59488 | 0.7635 | 0.81818 | 0.439456 | 0.001760 | 0.63756 | 0.7710 | 0.81818 | 0.439456 | 0.00170 | 0.61880 | 0.7774 | | 0.54545 | 0.427906 | 0.001771 | 0.55065 |
| ahb338 | 0.8466 | 0.8595 | 0.449044 | 0.002695 | 0.58879 | 0.8662 | 0.85294 | 0.441798 | 0.003260 | 0.63212 | 0.8730 | 0.86533 | 0.455674 | 0.00175 | 0.61316 | 0.8107 | | 0.57689 | 0.394991 | 0.002695 | 0.54197 |
| AMB-00425275 | 0.6838 | 0.7381 | 0.308067 | 0.009566 | 0.58417 | 0.4764 | 0.55182 | 0.177694 | 0.015600 | 0.62144 | 0.8952 | 0.90476 | 0.531359 | 0.00161 | 0.61987 | 0.6708 | | 0.60317 | 0.358797 | 0.009566 | 0.54539 |
| ahb3044 | 0.6561 | 0.6849 | 0.294452 | 0.007307 | 0.58328 | 0.4345 | 0.40809 | 0.134416 | 0.008300 | 0.62389 | 0.9178 | 0.91796 | 0.523436 | 0.01170 | 0.61459 | 0.7258 | | 0.61197 | 0.350114 | 0.007307 | 0.54365 |
| ahb1376 | 0.8106 | 0.8301 | 0.405504 | 0.005078 | 0.58557 | 0.8186 | 0.82353 | 0.398452 | 0.005620 | 0.63093 | 0.8279 | 0.83592 | 0.411962 | 0.00461 | 0.61190 | 0.7682 | | 0.55728 | 0.360383 | 0.005078 | 0.53901 |
| *AMB-00488882 | 0.8428 | 0.8562 | 0.436116 | 0.002814 | 0.58771 | 0.8277 | 0.85294 | 0.431638 | 0.002350 | 0.63226 | 0.8353 | 0.85913 | 0.440199 | 0.00365 | 0.61327 | 0.8061 | | 0.57275 | 0.398963 | 0.002814 | 0.54130 |
| AMB-00030513 | 0.5937 | 0.6277 | 0.255383 | 0.009447 | 0.58327 | 0.2808 | 0.28309 | 0.080510 | 0.010300 | 0.62156 | 0.9178 | 0.91796 | 0.523436 | 0.01185 | 0.61493 | 0.7425 | | 0.61197 | 0.363257 | 0.009447 | 0.54337 |
| est2739 | 0.8757 | 0.8856 | 0.474003 | 0.000000 | 0.58835 | 0.9199 | 0.94118 | 0.579557 | 0.000000 | 0.64059 | 0.8279 | 0.83592 | 0.411962 | 0.00000 | 0.61090 | 0.8498 | | 0.62745 | 0.459971 | 0.000000 | 0.54661 |
| *AMB-01009509 | 0.8132 | 0.8308 | 0.417872 | 0.003231 | 0.58743 | 0.9121 | 0.91088 | 0.512984 | 0.001760 | 0.63417 | 0.7752 | 0.75917 | 0.351594 | 0.00512 | 0.61193 | 0.7802 | | 0.60725 | 0.386549 | 0.003231 | 0.54143 |
| AMB-00634738 | 0.8158 | 0.8317 | 0.419326 | 0.004176 | 0.58671 | 0.8662 | 0.85294 | 0.441798 | 0.001150 | 0.63331 | 0.8268 | 0.81270 | 0.400444 | 0.00978 | 0.61163 | 0.7750 | | 0.56863 | 0.368401 | 0.004176 | 0.54080 |
| ahb10764 | 0.8422 | 0.8576 | 0.446008 | 0.001701 | 0.58826 | 0.8093 | 0.79228 | 0.380035 | 0.002120 | 0.63130 | 0.9145 | 0.91612 | 0.520292 | 0.00298 | 0.61452 | 0.8098 | | 0.61075 | 0.402303 | 0.001701 | 0.54222 |
| AMB-00085080 | 0.7034 | 0.7059 | 0.346350 | 0.005988 | 0.59350 | 0.6276 | 0.70588 | 0.346350 | 0.007880 | 0.63667 | 0.6375 | 0.70588 | 0.346350 | 0.00274 | 0.61651 | 0.6461 | | 0.47059 | 0.343662 | 0.005988 | 0.54935 |
| *AMB-00386010 | 0.6931 | 0.7206 | 0.321103 | 0.007468 | 0.58544 | 0.9139 | 0.91177 | 0.514510 | 0.004240 | 0.63389 | 0.5715 | 0.54954 | 0.207704 | 0.01381 | 0.60732 | 0.6998 | | 0.60785 | 0.346922 | 0.007468 | 0.54138 |
| *est8195 | 0.6793 | 0.6944 | 0.337961 | 0.007018 | 0.59291 | 0.7221 | 0.64706 | 0.304877 | 0.006610 | 0.63671 | 0.7880 | 0.73684 | 0.369943 | 0.00431 | 0.61847 | 0.6239 | | 0.49123 | 0.281621 | 0.007018 | 0.54449 |
| est2230 | 0.7662 | 0.7778 | 0.403395 | 0.000498 | 0.59313 | 0.9584 | 0.94118 | 0.579557 | 0.000510 | 0.64312 | 0.6932 | 0.63158 | 0.294600 | 0.00052 | 0.61606 | 0.7612 | | 0.62745 | 0.398028 | 0.000498 | 0.54792 |
| est4131 | 0.6734 | 0.6765 | 0.325107 | 0.005764 | 0.59371 | 0.5939 | 0.67647 | 0.325107 | 0.003970 | 0.63653 | 0.6043 | 0.67647 | 0.325107 | 0.01555 | 0.61601 | 0.6131 | | 0.45098 | 0.323936 | 0.005764 | 0.54994 |
| est10013 | 0.7237 | 0.7484 | 0.343244 | 0.014154 | 0.58561 | 0.7643 | 0.73530 | 0.332658 | 0.011680 | 0.62946 | 0.7791 | 0.76006 | 0.352983 | 0.01283 | 0.61071 | 0.6690 | | 0.50671 | 0.293947 | 0.014154 | 0.53842 |
| ahb11976 | 0.6734 | 0.6765 | 0.325107 | 0.005573 | 0.59295 | 0.5939 | 0.67647 | 0.325107 | 0.005990 | 0.63691 | 0.6043 | 0.67647 | 0.325107 | 0.01099 | 0.61637 | 0.6131 | | 0.45098 | 0.323936 | 0.005573 | 0.54799 |
| ahb10146 | 0.8099 | 0.8268 | 0.402833 | 0.005511 | 0.58604 | 0.7860 | 0.82353 | 0.398452 | 0.005160 | 0.63060 | 0.7951 | 0.82972 | 0.406831 | 0.00382 | 0.61188 | 0.7674 | | 0.55315 | 0.371034 | 0.005511 | 0.54013 |
| est1897 | 0.6793 | 0.6944 | 0.337961 | 0.002029 | 0.59294 | 0.4910 | 0.41176 | 0.169703 | 0.001880 | 0.63396 | 0.9602 | 0.94737 | 0.588665 | 0.00203 | 0.62256 | 0.7646 | | 0.63158 | 0.394169 | 0.002029 | 0.54874 |
| ahb2991 | 0.6692 | 0.7222 | 0.358634 | 0.005589 | 0.59251 | 0.7246 | 0.70588 | 0.346350 | 0.005760 | 0.63660 | 0.7435 | 0.73684 | 0.369943 | 0.00460 | 0.61745 | 0.5845 | | 0.49123 | 0.298374 | 0.005589 | 0.54527 |
| AMB-00452078 | 0.7333 | 0.7353 | 0.368731 | 0.003723 | 0.59384 | 0.6619 | 0.73529 | 0.368731 | 0.003490 | 0.63729 | 0.6713 | 0.73529 | 0.368731 | 0.00378 | 0.61740 | 0.6795 | | 0.49019 | 0.364245 | 0.003723 | 0.54928 |
| ahb8348 | 0.6793 | 0.6944 | 0.337961 | 0.007176 | 0.59221 | 0.7221 | 0.64706 | 0.304877 | 0.008350 | 0.63682 | 0.7880 | 0.73684 | 0.369943 | 0.00272 | 0.61815 | 0.6239 | | 0.49123 | 0.281621 | 0.007176 | 0.54456 |
| est7073 | 0.8106 | 0.8299 | 0.416342 | 0.003038 | 0.58807 | 0.9103 | 0.90993 | 0.511378 | 0.003790 | 0.63561 | 0.7712 | 0.75822 | 0.350133 | 0.00170 | 0.61147 | 0.7760 | | 0.60662 | 0.385349 | 0.003038 | 0.54195 |
| AMB-01109975 | 0.7953 | 0.8056 | 0.427820 | 0.001771 | 0.59393 | 0.8698 | 0.82353 | 0.444495 | 0.001760 | 0.63796 | 0.8331 | 0.78947 | 0.413493 | 0.00170 | 0.61952 | 0.7508 | | 0.54902 | 0.363423 | 0.001771 | 0.54645 |
| ahb11407 | 0.8106 | 0.8301 | 0.405504 | 0.003919 | 0.58751 | 0.8186 | 0.82353 | 0.398452 | 0.004670 | 0.63128 | 0.8279 | 0.83592 | 0.411962 | 0.00395 | 0.61216 | 0.7682 | | 0.55728 | 0.360383 | 0.003919 | 0.54043 |
| ahb5683 | 0.7662 | 0.7778 | 0.403395 | 0.001365 | 0.59382 | 0.9150 | 0.88235 | 0.505105 | 0.000000 | 0.64012 | 0.7414 | 0.68421 | 0.330595 | 0.00438 | 0.61801 | 0.7353 | | 0.58823 | 0.363540 | 0.001365 | 0.54746 |
| est957 | 0.7082 | 0.7222 | 0.358634 | 0.003273 | 0.59198 | 0.7735 | 0.70588 | 0.346350 | 0.005140 | 0.63717 | 0.7880 | 0.73684 | 0.369943 | 0.00724 | 0.61825 | 0.6519 | | 0.49123 | 0.298374 | 0.003273 | 0.54509 |
| ahb10347 | 0.5928 | 0.6111 | 0.281374 | 0.014832 | 0.59208 | 0.7735 | 0.70588 | 0.346350 | 0.015380 | 0.63753 | 0.5919 | 0.52632 | 0.230453 | 0.00440 | 0.61619 | 0.5465 | | 0.47059 | 0.244604 | 0.014832 | 0.54475 |
| AMB-00110490 | 0.7889 | 0.8235 | 0.444495 | 0.000524 | 0.59418 | 0.8894 | 0.87500 | 0.496929 | 0.000570 | 0.64027 | 0.7891 | 0.77778 | 0.403395 | 0.00088 | 0.61885 | 0.7314 | | 0.58333 | 0.385792 | 0.000524 | 0.54657 |
| est5112 | 0.7631 | 0.7647 | 0.392407 | 0.002416 | 0.59448 | 0.6968 | 0.76471 | 0.392407 | 0.002550 | 0.63777 | 0.7056 | 0.76471 | 0.392407 | 0.00257 | 0.61849 | 0.7133 | | 0.50981 | 0.385792 | 0.002416 | 0.55079 |
| ahb5454 | 0.7033 | 0.7435 | 0.310346 | 0.006184 | 0.58392 | 0.5364 | 0.58823 | 0.193490 | 0.007470 | 0.62047 | 0.8481 | 0.88235 | 0.505105 | 0.00448 | 0.62043 | 0.6872 | | 0.58823 | 0.347034 | 0.006184 | 0.54591 |
| *est11617 | 0.5353 | 0.5556 | 0.247361 | 0.008717 | 0.59133 | 0.8698 | 0.82353 | 0.444495 | 0.006250 | 0.63886 | 0.3665 | 0.31579 | 0.124335 | 0.00900 | 0.61430 | 0.6340 | | 0.54902 | 0.300735 | 0.008717 | 0.54529 |
| AMB-00658620 | 0.7085 | 0.7412 | 0.321088 | 0.082505 | 0.58392 | 0.7082 | 0.70589 | 0.292838 | 0.013890 | 0.62812 | 0.7717 | 0.77451 | 0.350181 | 0.16648 | 0.60986 | 0.6552 | | 0.51634 | 0.281045 | 0.082505 | 0.53597 |
| *ahb12438 | 0.5928 | 0.6111 | 0.281374 | 0.007811 | 0.59115 | 0.4910 | 0.41176 | 0.169703 | 0.011370 | 0.63495 | 0.8331 | 0.78947 | 0.413493 | 0.00497 | 0.61869 | 0.6127 | | 0.52631 | 0.275796 | 0.007811 | 0.54629 |
| ahb213 | 0.7742 | 0.7990 | 0.368035 | 0.007174 | 0.58594 | 0.7269 | 0.76470 | 0.330937 | 0.007640 | 0.62824 | 0.7951 | 0.82972 | 0.406831 | 0.00398 | 0.61246 | 0.7279 | | 0.55315 | 0.337784 | 0.007174 | 0.54040 |
| AMB-01115569 | 0.6435 | 0.6471 | 0.304877 | 0.006576 | 0.59328 | 0.5608 | 0.64706 | 0.304877 | 0.007970 | 0.63511 | 0.5715 | 0.64706 | 0.304877 | 0.00886 | 0.61580 | 0.5805 | | 0.43137 | 0.304978 | 0.006576 | 0.54898 |
| ahb5267 | 0.7441 | 0.7680 | 0.345540 | 0.007123 | 0.58496 | 0.6398 | 0.70588 | 0.277311 | 0.009030 | 0.62676 | 0.7757 | 0.82353 | 0.444495 | 0.00305 | 0.61721 | 0.6982 | | 0.54902 | 0.345979 | 0.007123 | 0.54460 |
| *est10387 | 0.7850 | 0.8039 | 0.392072 | 0.004430 | 0.58712 | 0.8164 | 0.79412 | 0.382949 | 0.008580 | 0.63109 | 0.8268 | 0.81270 | 0.400444 | 0.00444 | 0.61133 | 0.7386 | | 0.54180 | 0.339718 | 0.004430 | 0.53932 |
| AMB-00065920 | 0.7104 | 0.7097 | 0.349171 | 0.001239 | 0.59327 | 0.9540 | 0.92308 | 0.554591 | 0.000000 | 0.64057 | 0.6288 | 0.55556 | 0.247361 | 0.00481 | 0.61543 | 0.7243 | | 0.61539 | 0.373662 | 0.001239 | 0.54760 |
| AMB-01034105 | 0.7675 | 0.7946 | 0.367961 | 0.004644 | 0.58591 | 0.8614 | 0.87500 | 0.459356 | 0.003310 | 0.63370 | 0.7168 | 0.72697 | 0.307929 | 0.01086 | 0.60815 | 0.7270 | | 0.58333 | 0.346997 | 0.004644 | 0.53961 |
| ahb4580 | 0.7770 | 0.7974 | 0.372889 | 0.002258 | 0.58606 | 0.8053 | 0.85294 | 0.473508 | 0.003230 | 0.63876 | 0.6982 | 0.74768 | 0.315239 | 0.01741 | 0.60744 | 0.7343 | | 0.56863 | 0.375061 | 0.002258 | 0.54491 |
| *est4053 | 0.6625 | 0.6928 | 0.300212 | 0.007462 | 0.58549 | 0.9139 | 0.91177 | 0.514510 | 0.009090 | 0.63554 | 0.5151 | 0.49691 | 0.178698 | 0.00395 | 0.60567 | 0.6895 | | 0.60785 | 0.343755 | 0.007462 | 0.54130 |
| ahb10502 | 0.7397 | 0.7696 | 0.338911 | 0.005109 | 0.58631 | 0.8053 | 0.85294 | 0.473508 | 0.002800 | 0.63826 | 0.6394 | 0.69505 | 0.266221 | 0.00796 | 0.60513 | 0.6985 | | 0.56863 | 0.354410 | 0.005109 | 0.54402 |
| ahb3462 | 0.8459 | 0.8546 | 0.444533 | 0.000880 | 0.58895 | 0.7860 | 0.82353 | 0.398452 | 0.001060 | 0.63049 | 0.8481 | 0.88235 | 0.505105 | 0.00112 | 0.61968 | 0.8111 | | 0.58823 | 0.426740 | 0.000880 | 0.54620 |
| AMB-00272641 | 0.6903 | 0.6786 | 0.326590 | 0.004283 | 0.59397 | 0.8566 | 0.78571 | 0.410219 | 0.003330 | 0.63807 | 0.6815 | 0.57143 | 0.256816 | 0.00521 | 0.61604 | 0.6646 | | 0.52381 | 0.287776 | 0.004283 | 0.54489 |
| est11225 | 0.7770 | 0.7974 | 0.372889 | 0.005398 | 0.58592 | 0.7451 | 0.79412 | 0.368604 | 0.005780 | 0.63114 | 0.7557 | 0.80031 | 0.376800 | 0.00614 | 0.61151 | 0.7293 | | 0.53354 | 0.345475 | 0.005398 | 0.53997 |
| *est1224 | 0.7832 | 0.7958 | 0.385586 | 0.002251 | 0.58807 | 0.7684 | 0.82353 | 0.444495 | 0.004580 | 0.63805 | 0.7169 | 0.77090 | 0.349364 | 0.00532 | 0.61188 | 0.7376 | | 0.54902 | 0.379917 | 0.002251 | 0.54554 |
| *est2115 | 0.7850 | 0.8039 | 0.392072 | 0.004498 | 0.58691 | 0.8662 | 0.85294 | 0.441798 | 0.004140 | 0.63200 | 0.7791 | 0.76006 | 0.352983 | 0.00479 | 0.61055 | 0.7424 | | 0.56863 | 0.347602 | 0.004498 | 0.53951 |
| ahb10509 | 0.5353 | 0.5556 | 0.247361 | 0.005870 | 0.59123 | 0.8698 | 0.82353 | 0.444495 | 0.007130 | 0.63997 | 0.3665 | 0.31579 | 0.124335 | 0.00851 | 0.61494 | 0.6340 | | 0.54902 | 0.300735 | 0.005870 | 0.54466 |
| *est5927 | 0.7850 | 0.8039 | 0.392072 | 0.000453 | 0.58776 | 0.6530 | 0.61765 | 0.248888 | 0.000510 | 0.62694 | 0.9611 | 0.97059 | 0.626369 | 0.00043 | 0.62146 | 0.7931 | | 0.64706 | 0.425564 | 0.000453 | 0.54851 |
| *AMB-01061233 | 0.7407 | 0.7729 | 0.341175 | 0.008293 | 0.58534 | 0.7143 | 0.73529 | 0.307032 | 0.013440 | 0.62777 | 0.7838 | 0.80650 | 0.375536 | 0.00558 | 0.61145 | 0.6902 | | 0.53767 | 0.305146 | 0.008293 | 0.53595 |
| *AMB-00608448 | 0.6931 | 0.7206 | 0.321103 | 0.007989 | 0.58421 | 0.5934 | 0.55883 | 0.213063 | 0.008580 | 0.62619 | 0.8730 | 0.86533 | 0.455674 | 0.00469 | 0.61399 | 0.6817 | | 0.57689 | 0.310886 | 0.007989 | 0.54046 |
| ahb226 | 0.6442 | 0.6833 | 0.266800 | 0.011948 | 0.58411 | 0.7074 | 0.76667 | 0.394036 | 0.017790 | 0.63743 | 0.5147 | 0.60878 | 0.200581 | 0.01482 | 0.60372 | 0.5884 | | 0.51111 | 0.291164 | 0.011948 | 0.54216 |
| est7856 | 0.8158 | 0.8317 | 0.419326 | 0.001858 | 0.58738 | 0.9139 | 0.91177 | 0.514510 | 0.000780 | 0.63514 | 0.7791 | 0.76006 | 0.352983 | 0.00344 | 0.61177 | 0.7842 | | 0.60785 | 0.387687 | 0.001858 | 0.54163 |
| est8756 | 0.6793 | 0.6944 | 0.337961 | 0.001771 | 0.59296 | 0.9584 | 0.94118 | 0.579557 | 0.001760 | 0.64138 | 0.5385 | 0.47368 | 0.201532 | 0.00170 | 0.61536 | 0.7319 | | 0.62745 | 0.386376 | 0.001771 | 0.54753 |
| *ahb4257 | 0.7543 | 0.7762 | 0.366819 | 0.003371 | 0.58650 | 0.9139 | 0.91177 | 0.514510 | 0.003270 | 0.63535 | 0.6788 | 0.65480 | 0.273388 | 0.00340 | 0.60934 | 0.7339 | | 0.60785 | 0.361125 | 0.003371 | 0.54183 |
| ahb9104 | 0.6931 | 0.7206 | 0.321103 | 0.015465 | 0.58471 | 0.8662 | 0.85294 | 0.441798 | 0.007130 | 0.63267 | 0.6260 | 0.60217 | 0.239128 | 0.00425 | 0.60733 | 0.6664 | | 0.56863 | 0.308392 | 0.015465 | 0.53843 |
| est3830 | 0.6815 | 0.7190 | 0.303025 | 0.010907 | 0.58370 | 0.5239 | 0.52942 | 0.178895 | 0.005320 | 0.62298 | 0.8761 | 0.88855 | 0.478173 | 0.00413 | 0.61348 | 0.6855 | | 0.59237 | 0.322030 | 0.010907 | 0.54135 |
| ahb6174 | 0.5640 | 0.5833 | 0.264041 | 0.011045 | 0.59210 | 0.7221 | 0.64706 | 0.304877 | 0.009050 | 0.63685 | 0.5919 | 0.52632 | 0.230453 | 0.00842 | 0.61636 | 0.5060 | | 0.43137 | 0.222245 | 0.011045 | 0.54314 |
| AMB-00013530 | 0.7136 | 0.7467 | 0.325761 | 0.010999 | 0.58483 | 0.7646 | 0.76471 | 0.341352 | 0.014680 | 0.62893 | 0.7268 | 0.73065 | 0.312410 | 0.00849 | 0.60848 | 0.6581 | | 0.50981 | 0.285115 | 0.010999 | 0.53542 |
| AMB-00744399 | 0.4495 | 0.4722 | 0.200754 | 0.013346 | 0.59114 | 0.0439 | 0.05882 | 0.020832 | 0.000390 | 0.63483 | 0.8768 | 0.84211 | 0.462548 | 0.00369 | 0.62067 | 0.8026 | | 0.56141 | 0.391166 | 0.013346 | 0.54889 |
| AMB-00605425 | 0.7662 | 0.7778 | 0.403395 | 0.000973 | 0.59455 | 0.7221 | 0.64706 | 0.304877 | 0.000000 | 0.63682 | 0.9191 | 0.89474 | 0.519349 | 0.00119 | 0.62033 | 0.7455 | | 0.59649 | 0.364178 | 0.000973 | 0.54762 |
| *AMB-00856434 | 0.8432 | 0.8510 | 0.440644 | 0.000553 | 0.58903 | 0.8394 | 0.87879 | 0.501116 | 0.000680 | 0.63938 | 0.7909 | 0.82616 | 0.403031 | 0.00102 | 0.61149 | 0.8067 | | 0.58586 | 0.427637 | 0.000553 | 0.54605 |
| *est9814 | 0.7027 | 0.7418 | 0.309298 | 0.010303 | 0.58286 | 0.6184 | 0.67647 | 0.250928 | 0.013250 | 0.62471 | 0.7557 | 0.80031 | 0.376800 | 0.01444 | 0.61111 | 0.6528 | | 0.53354 | 0.290554 | 0.010303 | 0.53732 |
| AMB-00151688 | 0.7875 | 0.8121 | 0.384527 | 0.004342 | 0.58427 | 0.8030 | 0.81568 | 0.388277 | 0.002970 | 0.63027 | 0.7970 | 0.80833 | 0.380595 | 0.00667 | 0.61164 | 0.7392 | | 0.54379 | 0.341661 | 0.004342 | 0.53917 |
| *est763 | 0.8404 | 0.8472 | 0.436563 | 0.000880 | 0.58851 | 0.8359 | 0.87500 | 0.496929 | 0.000790 | 0.63869 | 0.7865 | 0.82237 | 0.399045 | 0.00092 | 0.61202 | 0.8023 | | 0.58333 | 0.424147 | 0.000880 | 0.54665 |
| *ahb3272 | 0.7372 | 0.7500 | 0.380396 | 0.001809 | 0.59306 | 0.9150 | 0.88235 | 0.505105 | 0.001470 | 0.64050 | 0.6932 | 0.63158 | 0.294600 | 0.00153 | 0.61636 | 0.7146 | | 0.58823 | 0.352997 | 0.001809 | 0.54698 |
| *ahb3426 | 0.7414 | 0.7698 | 0.345863 | 0.006936 | 0.58516 | 0.6972 | 0.69118 | 0.281733 | 0.004840 | 0.62629 | 0.8279 | 0.83592 | 0.411962 | 0.00641 | 0.61297 | 0.6997 | | 0.55728 | 0.309728 | 0.006936 | 0.53769 |
| AMB-00081835 | 0.7928 | 0.8056 | 0.427820 | 0.000286 | 0.59489 | 0.8674 | 0.82353 | 0.444495 | 0.000000 | 0.63881 | 0.8303 | 0.78947 | 0.413493 | 0.00075 | 0.61904 | 0.7467 | | 0.54902 | 0.363423 | 0.000286 | 0.54701 |
| ahb8266 | 0.6625 | 0.6928 | 0.300212 | 0.026049 | 0.58269 | 0.7643 | 0.73530 | 0.332658 | 0.025020 | 0.63143 | 0.6788 | 0.65480 | 0.273388 | 0.04734 | 0.60839 | 0.6047 | | 0.49020 | 0.259063 | 0.026049 | 0.53589 |
| AMB-00910336 | 0.7850 | 0.8039 | 0.392072 | 0.003802 | 0.58619 | 0.7643 | 0.73530 | 0.332658 | 0.004530 | 0.62883 | 0.8730 | 0.86533 | 0.455674 | 0.00198 | 0.61316 | 0.7461 | | 0.57689 | 0.346940 | 0.003802 | 0.54063 |
| ahb3431 | 0.7033 | 0.7435 | 0.310346 | 0.010358 | 0.58392 | 0.6020 | 0.64706 | 0.232308 | 0.010240 | 0.62355 | 0.7951 | 0.82972 | 0.406831 | 0.00726 | 0.61082 | 0.6621 | | 0.55315 | 0.297457 | 0.010358 | 0.53804 |
| AMB-00963630 | 0.7631 | 0.7647 | 0.392407 | 0.001696 | 0.59298 | 0.6968 | 0.76471 | 0.392407 | 0.002590 | 0.63742 | 0.7056 | 0.76471 | 0.392407 | 0.00238 | 0.61684 | 0.7133 | | 0.50981 | 0.385792 | 0.001696 | 0.54983 |
| ahb9990 | 0.7407 | 0.7729 | 0.341175 | 0.005820 | 0.58490 | 0.7722 | 0.79411 | 0.362391 | 0.010640 | 0.62918 | 0.7307 | 0.75387 | 0.323420 | 0.00875 | 0.60864 | 0.6886 | | 0.52941 | 0.305656 | 0.005820 | 0.53693 |
| est2028 | 0.7782 | 0.8023 | 0.376609 | 0.003580 | 0.58666 | 0.8703 | 0.88236 | 0.469429 | 0.002940 | 0.63316 | 0.7268 | 0.73065 | 0.312410 | 0.00431 | 0.60857 | 0.7409 | | 0.58824 | 0.353712 | 0.003580 | 0.54012 |
| *est7824 | 0.8459 | 0.8546 | 0.444533 | 0.000437 | 0.58853 | 0.8428 | 0.88235 | 0.505105 | 0.000470 | 0.63860 | 0.7951 | 0.82972 | 0.406831 | 0.00044 | 0.61249 | 0.8109 | | 0.58823 | 0.430956 | 0.000437 | 0.54559 |
| ahb1081 | 0.5944 | 0.6177 | 0.285554 | 0.007075 | 0.59189 | 0.7529 | 0.68750 | 0.332948 | 0.006450 | 0.63721 | 0.6136 | 0.55556 | 0.247361 | 0.00644 | 0.61615 | 0.5366 | | 0.45833 | 0.242387 | 0.007075 | 0.54392 |
| ahb2943 | 0.6931 | 0.7206 | 0.321103 | 0.010984 | 0.58437 | 0.8662 | 0.85294 | 0.441798 | 0.002870 | 0.63328 | 0.6260 | 0.60217 | 0.239128 | 0.00820 | 0.60692 | 0.6664 | | 0.56863 | 0.308392 | 0.010984 | 0.53932 |
| *ahb3142 | 0.6257 | 0.6487 | 0.270536 | 0.014657 | 0.58584 | 0.5939 | 0.67647 | 0.325107 | 0.015830 | 0.63619 | 0.5332 | 0.62384 | 0.238145 | 0.01899 | 0.60646 | 0.5631 | | 0.45098 | 0.275988 | 0.014657 | 0.54336 |
| ahb3988 | 0.6215 | 0.6389 | 0.299423 | 0.004804 | 0.59293 | 0.5530 | 0.47059 | 0.199887 | 0.003540 | 0.63572 | 0.8331 | 0.78947 | 0.413493 | 0.00591 | 0.61830 | 0.6162 | | 0.52631 | 0.278276 | 0.004804 | 0.54547 |
| AMB-00282111 | 0.7782 | 0.8023 | 0.376609 | 0.003439 | 0.58606 | 0.8186 | 0.82353 | 0.398452 | 0.011350 | 0.63148 | 0.7782 | 0.78329 | 0.358291 | 0.00377 | 0.61030 | 0.7313 | | 0.54902 | 0.334510 | 0.003439 | 0.53850 |
| ahb7032 | 0.7607 | 0.7778 | 0.403395 | 0.000837 | 0.59390 | 0.7660 | 0.70588 | 0.346350 | 0.000770 | 0.63773 | 0.8724 | 0.84211 | 0.462548 | 0.00099 | 0.62106 | 0.7168 | | 0.56141 | 0.345315 | 0.000837 | 0.54707 |
| AMB-01103262 | 0.7053 | 0.7402 | 0.312386 | 0.014913 | 0.58485 | 0.6398 | 0.70588 | 0.277311 | 0.017370 | 0.62548 | 0.7169 | 0.77090 | 0.349364 | 0.00628 | 0.61045 | 0.6503 | | 0.51393 | 0.290273 | 0.014913 | 0.53612 |
| AMB-00441225 | 0.6493 | 0.6765 | 0.288447 | 0.009819 | 0.58282 | 0.5311 | 0.50000 | 0.180343 | 0.005010 | 0.62692 | 0.8662 | 0.85294 | 0.441798 | 0.00940 | 0.61388 | 0.6542 | | 0.56863 | 0.296939 | 0.009819 | 0.53955 |
| ahb11064 | 0.7459 | 0.7745 | 0.350181 | 0.006196 | 0.58537 | 0.7082 | 0.70589 | 0.292838 | 0.006180 | 0.62752 | 0.8279 | 0.83592 | 0.411962 | 0.00382 | 0.61098 | 0.7016 | | 0.55728 | 0.313920 | 0.006196 | 0.53847 |
| AMB-00469027 | 0.6017 | 0.6373 | 0.261622 | 0.011414 | 0.58260 | 0.3263 | 0.32353 | 0.096955 | 0.003000 | 0.62053 | 0.9178 | 0.91796 | 0.523436 | 0.00342 | 0.61527 | 0.7252 | | 0.61197 | 0.357313 | 0.011414 | 0.54357 |
| ahb1791 | 0.7459 | 0.7745 | 0.350181 | 0.005704 | 0.58543 | 0.6493 | 0.64706 | 0.250446 | 0.005180 | 0.62638 | 0.8761 | 0.88855 | 0.478173 | 0.00170 | 0.61351 | 0.7207 | | 0.59237 | 0.337883 | 0.005704 | 0.54025 |
| ahb11533 | 0.6321 | 0.6650 | 0.280425 | 0.003375 | 0.58364 | 0.3263 | 0.32353 | 0.096955 | 0.001320 | 0.62235 | 0.9611 | 0.97059 | 0.626369 | 0.00044 | 0.62177 | 0.7799 | | 0.64706 | 0.429422 | 0.003375 | 0.54846 |
| AMB-00108337 | 0.7237 | 0.7484 | 0.343244 | 0.009151 | 0.58671 | 0.7099 | 0.67647 | 0.288447 | 0.009620 | 0.62671 | 0.8268 | 0.81270 | 0.400444 | 0.00184 | 0.61209 | 0.6778 | | 0.54180 | 0.300265 | 0.009151 | 0.53896 |
| AMB-00769113 | 0.6734 | 0.6765 | 0.325107 | 0.003742 | 0.59266 | 0.5939 | 0.67647 | 0.325107 | 0.003800 | 0.63749 | 0.6043 | 0.67647 | 0.325107 | 0.00445 | 0.61603 | 0.6131 | | 0.45098 | 0.323936 | 0.003742 | 0.54926 |
| ahb11692 | 0.6017 | 0.6373 | 0.261622 | 0.011710 | 0.58309 | 0.3263 | 0.32353 | 0.096955 | 0.003250 | 0.62225 | 0.9178 | 0.91796 | 0.523436 | 0.00240 | 0.61506 | 0.7252 | | 0.61197 | 0.357313 | 0.011710 | 0.54410 |
| AMB-00631953 | 0.5972 | 0.5862 | 0.265802 | 0.006035 | 0.59338 | 0.5004 | 0.58621 | 0.265802 | 0.007140 | 0.63512 | 0.5192 | 0.58621 | 0.265802 | 0.00841 | 0.61685 | 0.5234 | | 0.39081 | 0.267892 | 0.006035 | 0.54841 |
| ahb8533 | 0.6733 | 0.7173 | 0.291977 | 0.010931 | 0.58248 | 0.8277 | 0.85294 | 0.431638 | 0.006790 | 0.63156 | 0.5620 | 0.59597 | 0.205983 | 0.00677 | 0.60349 | 0.6457 | | 0.56863 | 0.301035 | 0.010931 | 0.53833 |
| ahb5288 | 0.7850 | 0.8039 | 0.392072 | 0.003524 | 0.58774 | 0.8164 | 0.79412 | 0.382949 | 0.003980 | 0.63148 | 0.8268 | 0.81270 | 0.400444 | 0.00325 | 0.61070 | 0.7386 | | 0.54180 | 0.339718 | 0.003524 | 0.53890 |
| est1929 | 0.7386 | 0.7712 | 0.337604 | 0.005939 | 0.58647 | 0.6656 | 0.70588 | 0.277311 | 0.006000 | 0.62569 | 0.7951 | 0.82972 | 0.406831 | 0.00486 | 0.61094 | 0.6927 | | 0.55315 | 0.314413 | 0.005939 | 0.53907 |
| ahb12387 | 0.6734 | 0.6765 | 0.325107 | 0.003069 | 0.59300 | 0.5939 | 0.67647 | 0.325107 | 0.003760 | 0.63600 | 0.6043 | 0.67647 | 0.325107 | 0.00551 | 0.61657 | 0.6131 | | 0.45098 | 0.323936 | 0.003069 | 0.54925 |
| *ahb6599 | 0.7574 | 0.7799 | 0.355128 | 0.002100 | 0.58593 | 0.6529 | 0.71371 | 0.283202 | 0.003250 | 0.62598 | 0.7958 | 0.83871 | 0.459181 | 0.00645 | 0.61839 | 0.7124 | | 0.55914 | 0.354940 | 0.002100 | 0.54397 |
| *ahb5286 | 0.7237 | 0.7484 | 0.343244 | 0.006494 | 0.58655 | 0.8164 | 0.79412 | 0.382949 | 0.008600 | 0.63203 | 0.7298 | 0.70743 | 0.311061 | 0.00315 | 0.60961 | 0.6724 | | 0.52941 | 0.299680 | 0.006494 | 0.53817 |
| est9104 | 0.6625 | 0.6928 | 0.300212 | 0.010293 | 0.58512 | 0.5934 | 0.55883 | 0.213063 | 0.005720 | 0.62624 | 0.8268 | 0.81270 | 0.400444 | 0.01250 | 0.61169 | 0.6351 | | 0.54180 | 0.277338 | 0.010293 | 0.53746 |
| AMB-00100154 | 0.8066 | 0.8284 | 0.400499 | 0.002426 | 0.58704 | 0.7891 | 0.81176 | 0.381319 | 0.002540 | 0.62958 | 0.8142 | 0.84033 | 0.415129 | 0.00328 | 0.61232 | 0.7763 | | 0.56022 | 0.360416 | 0.002426 | 0.54024 |
| *ahb9698 | 0.6504 | 0.6667 | 0.318257 | 0.001722 | 0.59262 | 0.4910 | 0.41176 | 0.169703 | 0.002160 | 0.63491 | 0.9191 | 0.89474 | 0.519349 | 0.00251 | 0.62181 | 0.7131 | | 0.59649 | 0.346807 | 0.001722 | 0.54706 |
| ahb7322 | 0.8395 | 0.8545 | 0.442474 | 0.000610 | 0.58735 | 0.8566 | 0.84375 | 0.430831 | 0.000870 | 0.63288 | 0.8681 | 0.86349 | 0.452627 | 0.00112 | 0.61396 | 0.8012 | | 0.57566 | 0.388769 | 0.000610 | 0.54177 |
| AMB-01132855 | 0.7202 | 0.7475 | 0.341866 | 0.006728 | 0.58493 | 0.7050 | 0.67558 | 0.287137 | 0.006480 | 0.62907 | 0.8237 | 0.81181 | 0.399007 | 0.00385 | 0.61147 | 0.6725 | | 0.54121 | 0.299200 | 0.006728 | 0.53822 |
| ahb6576 | 0.8158 | 0.8317 | 0.419326 | 0.001739 | 0.58770 | 0.8662 | 0.85294 | 0.441798 | 0.001950 | 0.63354 | 0.8268 | 0.81270 | 0.400444 | 0.00184 | 0.61279 | 0.7750 | | 0.56863 | 0.368401 | 0.001739 | 0.54124 |
| AMB-00712118 | 0.6733 | 0.7173 | 0.291977 | 0.008123 | 0.58260 | 0.5915 | 0.61764 | 0.219684 | 0.010060 | 0.62442 | 0.7838 | 0.80650 | 0.375536 | 0.01783 | 0.61057 | 0.6308 | | 0.53767 | 0.271624 | 0.008123 | 0.53600 |
| est1491 | 0.7000 | 0.7263 | 0.324222 | 0.008114 | 0.58543 | 0.7466 | 0.71875 | 0.318329 | 0.005950 | 0.62989 | 0.7557 | 0.73346 | 0.329856 | 0.00879 | 0.60969 | 0.6431 | | 0.48897 | 0.277014 | 0.008114 | 0.53646 |
| AMB-00594700 | 0.6931 | 0.7206 | 0.321103 | 0.009377 | 0.58454 | 0.7643 | 0.73530 | 0.332658 | 0.012520 | 0.62925 | 0.7298 | 0.70743 | 0.311061 | 0.00477 | 0.60941 | 0.6351 | | 0.49020 | 0.274727 | 0.009377 | 0.53678 |
| AMB-00607485 | 0.8431 | 0.8579 | 0.437570 | 0.000822 | 0.58667 | 0.8186 | 0.82353 | 0.398452 | 0.000830 | 0.63133 | 0.8761 | 0.88855 | 0.478173 | 0.00085 | 0.61349 | 0.8082 | | 0.59237 | 0.395173 | 0.000822 | 0.54099 |
| AMB-00373008 | 0.6668 | 0.6667 | 0.318257 | 0.002771 | 0.59240 | 0.5851 | 0.66667 | 0.318257 | 0.003180 | 0.63700 | 0.5957 | 0.66667 | 0.318257 | 0.00584 | 0.61665 | 0.6042 | | 0.44445 | 0.317535 | 0.002771 | 0.55016 |
| AMB-00881866 | 0.7782 | 0.8023 | 0.376609 | 0.002214 | 0.58575 | 0.7082 | 0.70589 | 0.292838 | 0.003210 | 0.62864 | 0.8761 | 0.88855 | 0.478173 | 0.00249 | 0.61377 | 0.7455 | | 0.59237 | 0.351581 | 0.002214 | 0.54130 |
| AMB-00870928 | 0.6134 | 0.6177 | 0.285554 | 0.003927 | 0.59111 | 0.5283 | 0.61765 | 0.285554 | 0.004450 | 0.63686 | 0.5393 | 0.61765 | 0.285554 | 0.01071 | 0.61636 | 0.5483 | | 0.41177 | 0.286715 | 0.003927 | 0.54858 |
| ahb891 | 0.7386 | 0.7712 | 0.337604 | 0.002112 | 0.58392 | 0.6020 | 0.64706 | 0.232308 | 0.002440 | 0.62146 | 0.8481 | 0.88235 | 0.505105 | 0.00632 | 0.61964 | 0.7112 | | 0.58823 | 0.357697 | 0.002112 | 0.54485 |
| ahb497 | 0.7253 | 0.7607 | 0.327013 | 0.005996 | 0.58486 | 0.6408 | 0.68750 | 0.261519 | 0.007290 | 0.62492 | 0.7865 | 0.82237 | 0.399045 | 0.00468 | 0.61098 | 0.6777 | | 0.54825 | 0.304680 | 0.005996 | 0.53894 |
| AMB-00179078 | 0.6733 | 0.7173 | 0.291977 | 0.009057 | 0.58297 | 0.5265 | 0.55882 | 0.183849 | 0.008770 | 0.62125 | 0.8353 | 0.85913 | 0.440199 | 0.00344 | 0.61219 | 0.6575 | | 0.57275 | 0.301089 | 0.009057 | 0.53973 |
| *est9604 | 0.7386 | 0.7712 | 0.337604 | 0.003268 | 0.58425 | 0.8428 | 0.88235 | 0.505105 | 0.002030 | 0.63926 | 0.6269 | 0.67182 | 0.250396 | 0.00247 | 0.60617 | 0.7061 | | 0.58823 | 0.363560 | 0.003268 | 0.54570 |
| est11342 | 0.8158 | 0.8317 | 0.419326 | 0.000877 | 0.58819 | 0.9139 | 0.91177 | 0.514510 | 0.001000 | 0.63447 | 0.7791 | 0.76006 | 0.352983 | 0.00099 | 0.61075 | 0.7842 | | 0.60785 | 0.387687 | 0.000877 | 0.54164 |
| est9645 | 0.6931 | 0.7206 | 0.321103 | 0.006043 | 0.58468 | 0.6530 | 0.61765 | 0.248888 | 0.010110 | 0.62750 | 0.8268 | 0.81270 | 0.400444 | 0.00527 | 0.61188 | 0.6540 | | 0.54180 | 0.287036 | 0.006043 | 0.53778 |
| ahb7699 | 0.8086 | 0.8284 | 0.400499 | 0.002236 | 0.58737 | 0.8277 | 0.85294 | 0.431638 | 0.002100 | 0.63162 | 0.7838 | 0.80650 | 0.375536 | 0.00265 | 0.61064 | 0.7668 | | 0.56863 | 0.366402 | 0.002236 | 0.53977 |
| AMB-00253137 | 0.7441 | 0.7680 | 0.345540 | 0.007000 | 0.58784 | 0.7051 | 0.76471 | 0.341352 | 0.001760 | 0.62897 | 0.7169 | 0.77090 | 0.349364 | 0.00894 | 0.60930 | 0.6917 | | 0.51393 | 0.321787 | 0.007000 | 0.53907 |
| ahb6411 | 0.6504 | 0.6667 | 0.318257 | 0.000262 | 0.59265 | 0.9584 | 0.94118 | 0.579557 | 0.000000 | 0.64246 | 0.4832 | 0.42105 | 0.174338 | 0.00147 | 0.61461 | 0.7318 | | 0.62745 | 0.387474 | 0.000262 | 0.54828 |
| AMB-01048080 | 0.5799 | 0.6485 | 0.229106 | 0.009432 | 0.57967 | 0.7976 | 0.84848 | 0.468960 | 0.002410 | 0.63904 | 0.3841 | 0.48006 | 0.127716 | 0.01627 | 0.59771 | 0.5848 | | 0.56565 | 0.314084 | 0.009432 | 0.54450 |
| est7592 | 0.5858 | 0.6356 | 0.242784 | 0.007713 | 0.58131 | 0.3172 | 0.35294 | 0.094988 | 0.004980 | 0.61601 | 0.8761 | 0.88855 | 0.478173 | 0.00863 | 0.61288 | 0.6730 | | 0.59237 | 0.322516 | 0.007713 | 0.54233 |
| ahb7458 | 0.7027 | 0.7418 | 0.309298 | 0.006119 | 0.58471 | 0.6184 | 0.67647 | 0.250928 | 0.007080 | 0.62545 | 0.7557 | 0.80031 | 0.376800 | 0.00719 | 0.61170 | 0.6528 | | 0.53354 | 0.290554 | 0.006119 | 0.53831 |
| AMB-00710546 | 0.7850 | 0.8039 | 0.392072 | 0.001676 | 0.58722 | 0.7099 | 0.67647 | 0.288447 | 0.001690 | 0.62706 | 0.9178 | 0.91796 | 0.523436 | 0.00163 | 0.61511 | 0.7644 | | 0.61197 | 0.370821 | 0.001676 | 0.54283 |
| ahb5117 | 0.7770 | 0.7974 | 0.372889 | 0.002659 | 0.58821 | 0.7451 | 0.79412 | 0.368604 | 0.003400 | 0.63012 | 0.7557 | 0.80031 | 0.376800 | 0.00443 | 0.61175 | 0.7293 | | 0.53354 | 0.345475 | 0.002659 | 0.53936 |
| *AMB-01113468 | 0.6504 | 0.6667 | 0.318257 | 0.003220 | 0.59182 | 0.5530 | 0.47059 | 0.199887 | 0.002720 | 0.63458 | 0.8768 | 0.84211 | 0.462548 | 0.00080 | 0.62002 | 0.6638 | | 0.56141 | 0.309471 | 0.003220 | 0.54571 |
| AMB-00609770 | 0.6888 | 0.7075 | 0.312385 | 0.004600 | 0.58554 | 0.5900 | 0.67647 | 0.270981 | 0.005200 | 0.62747 | 0.6713 | 0.73529 | 0.368731 | 0.00661 | 0.61825 | 0.6319 | | 0.49019 | 0.310709 | 0.004600 | 0.54322 |
| *ahb5929 | 0.7995 | 0.8135 | 0.400598 | 0.000899 | 0.58657 | 0.7894 | 0.84375 | 0.464191 | 0.000760 | 0.63813 | 0.7399 | 0.78819 | 0.364090 | 0.00292 | 0.61058 | 0.7574 | | 0.56250 | 0.394682 | 0.000899 | 0.54474 |
| ahb4959 | 0.7459 | 0.7745 | 0.350181 | 0.002191 | 0.58535 | 0.7646 | 0.76471 | 0.341352 | 0.009000 | 0.62993 | 0.7782 | 0.78329 | 0.358291 | 0.01248 | 0.61042 | 0.6939 | | 0.52219 | 0.306616 | 0.002191 | 0.53639 |
| AMB-00132090 | 0.6692 | 0.7222 | 0.358634 | 0.002242 | 0.59251 | 0.7246 | 0.70588 | 0.346350 | 0.001760 | 0.63677 | 0.7435 | 0.73684 | 0.369943 | 0.00455 | 0.61698 | 0.5845 | | 0.49123 | 0.298374 | 0.002242 | 0.54490 |
| *est9585 | 0.5353 | 0.5556 | 0.247361 | 0.006830 | 0.59127 | 0.5530 | 0.47059 | 0.199887 | 0.007860 | 0.63492 | 0.6932 | 0.63158 | 0.294600 | 0.00604 | 0.61733 | 0.4847 | | 0.42105 | 0.207709 | 0.006830 | 0.54341 |
| ahb12190 | 0.5944 | 0.6439 | 0.238709 | 0.008602 | 0.58189 | 0.6546 | 0.72727 | 0.362505 | 0.008600 | 0.63667 | 0.4600 | 0.56938 | 0.175482 | 0.01469 | 0.60124 | 0.5407 | | 0.48485 | 0.265912 | 0.008602 | 0.54279 |
| est4355 | 0.5928 | 0.6111 | 0.281374 | 0.005182 | 0.58992 | 0.7735 | 0.70588 | 0.346350 | 0.001990 | 0.63757 | 0.5919 | 0.52632 | 0.230453 | 0.00813 | 0.61636 | 0.5465 | | 0.47059 | 0.244604 | 0.005182 | 0.54426 |
| *ahb2783 | 0.6625 | 0.6928 | 0.300212 | 0.005222 | 0.58454 | 0.8662 | 0.85294 | 0.441798 | 0.003230 | 0.63298 | 0.5715 | 0.54954 | 0.207704 | 0.01028 | 0.60739 | 0.6493 | | 0.56863 | 0.301284 | 0.005222 | 0.53975 |
| ahb3118 | 0.5833 | 0.5882 | 0.267050 | 0.004728 | 0.59092 | 0.4963 | 0.58824 | 0.267050 | 0.004970 | 0.63493 | 0.5075 | 0.58824 | 0.267050 | 0.00760 | 0.61615 | 0.5165 | | 0.39216 | 0.269086 | 0.004728 | 0.54818 |
| *est828 | 0.7850 | 0.8039 | 0.392072 | 0.001625 | 0.58655 | 0.8662 | 0.85294 | 0.441798 | 0.002610 | 0.63395 | 0.7791 | 0.76006 | 0.352983 | 0.00368 | 0.60970 | 0.7424 | | 0.56863 | 0.347602 | 0.001625 | 0.53976 |
| AMB-00826573 | 0.6781 | 0.7092 | 0.296829 | 0.003836 | 0.58462 | 0.6968 | 0.76471 | 0.392407 | 0.005860 | 0.63781 | 0.5765 | 0.65945 | 0.243624 | 0.00726 | 0.60634 | 0.6240 | | 0.50981 | 0.308112 | 0.003836 | 0.54295 |
| AMB-00790811 | 0.7069 | 0.7451 | 0.315570 | 0.015819 | 0.58410 | 0.7722 | 0.79411 | 0.362391 | 0.007040 | 0.63002 | 0.6760 | 0.70123 | 0.279106 | 0.00023 | 0.60759 | 0.6542 | | 0.52941 | 0.286275 | 0.015819 | 0.53560 |
| ahb6367 | 0.4780 | 0.5000 | 0.215762 | 0.009431 | 0.59055 | 0.5530 | 0.47059 | 0.199887 | 0.019840 | 0.63476 | 0.5919 | 0.52632 | 0.230453 | 0.00913 | 0.61483 | 0.4111 | | 0.35088 | 0.174737 | 0.009431 | 0.54155 |
| *est11214 | 0.7053 | 0.7402 | 0.312386 | 0.005736 | 0.58334 | 0.6398 | 0.70588 | 0.277311 | 0.008610 | 0.62524 | 0.7169 | 0.77090 | 0.349364 | 0.00674 | 0.61021 | 0.6503 | | 0.51393 | 0.290273 | 0.005736 | 0.53800 |
| est9786 | 0.4808 | 0.5262 | 0.194534 | 0.012794 | 0.58220 | 0.9139 | 0.91177 | 0.514510 | 0.002800 | 0.63412 | 0.1391 | 0.18112 | 0.043191 | 0.00256 | 0.59935 | 0.7392 | | 0.60785 | 0.380022 | 0.012794 | 0.54387 |
| ahb781 | 0.7304 | 0.7604 | 0.330369 | 0.003236 | 0.58416 | 0.6695 | 0.72610 | 0.294597 | 0.007430 | 0.62728 | 0.7453 | 0.79112 | 0.367974 | 0.00532 | 0.61205 | 0.6753 | | 0.52741 | 0.305773 | 0.003236 | 0.53832 |
| AMB-00672348 | 0.8005 | 0.8144 | 0.388371 | 0.000761 | 0.58616 | 0.8385 | 0.87500 | 0.496929 | 0.000620 | 0.63902 | 0.7300 | 0.76974 | 0.336319 | 0.00071 | 0.60815 | 0.7508 | | 0.58333 | 0.394101 | 0.000761 | 0.54581 |
| est4307 | 0.3356 | 0.3611 | 0.145207 | 0.008932 | 0.58859 | 0.7735 | 0.70588 | 0.346350 | 0.007950 | 0.63781 | 0.0325 | 0.05263 | 0.018596 | 0.01071 | 0.61362 | 0.6692 | | 0.47059 | 0.294768 | 0.008932 | 0.54649 |
| ahb7365 | 0.7799 | 0.8089 | 0.378862 | 0.000158 | 0.58722 | 0.9015 | 0.92000 | 0.550559 | 0.000000 | 0.63903 | 0.6741 | 0.70947 | 0.287893 | 0.00022 | 0.60668 | 0.7409 | | 0.61333 | 0.398023 | 0.000158 | 0.54598 |
| *est7612 | 0.7166 | 0.7465 | 0.340416 | 0.005990 | 0.58480 | 0.8093 | 0.79228 | 0.380035 | 0.005790 | 0.63121 | 0.7206 | 0.70559 | 0.308313 | 0.00217 | 0.60859 | 0.6615 | | 0.52819 | 0.297483 | 0.005990 | 0.53696 |
| est6865 | 0.7782 | 0.8023 | 0.376609 | 0.002546 | 0.58607 | 0.8186 | 0.82353 | 0.398452 | 0.002870 | 0.63071 | 0.7782 | 0.78329 | 0.358291 | 0.00316 | 0.61050 | 0.7313 | | 0.54902 | 0.334510 | 0.002546 | 0.53794 |
| *ahb12365 | 0.7053 | 0.7402 | 0.312386 | 0.005307 | 0.58463 | 0.6398 | 0.70588 | 0.277311 | 0.007890 | 0.62526 | 0.7169 | 0.77090 | 0.349364 | 0.00617 | 0.61146 | 0.6503 | | 0.51393 | 0.290273 | 0.005307 | 0.53562 |
| est9285 | 0.7136 | 0.7467 | 0.325761 | 0.004874 | 0.58366 | 0.7646 | 0.76471 | 0.341352 | 0.005190 | 0.62968 | 0.7268 | 0.73065 | 0.312410 | 0.00561 | 0.60982 | 0.6581 | | 0.50981 | 0.285115 | 0.004874 | 0.53751 |
| est3744 | 0.5640 | 0.5833 | 0.264041 | 0.005341 | 0.59114 | 0.5530 | 0.47059 | 0.199887 | 0.005220 | 0.63450 | 0.7414 | 0.68421 | 0.330595 | 0.00492 | 0.61712 | 0.5262 | | 0.45614 | 0.228070 | 0.005341 | 0.54301 |
| *est4132 | 0.3939 | 0.4118 | 0.169703 | 0.014324 | 0.58970 | 0.3123 | 0.25000 | 0.095603 | 0.015240 | 0.63433 | 0.6288 | 0.55556 | 0.247361 | 0.01426 | 0.61560 | 0.4145 | | 0.37037 | 0.165348 | 0.014324 | 0.54124 |
| AMB-00356061 | 0.7136 | 0.7467 | 0.325761 | 0.005419 | 0.58398 | 0.7082 | 0.70589 | 0.292838 | 0.004630 | 0.62703 | 0.7782 | 0.78329 | 0.358291 | 0.00477 | 0.61105 | 0.6604 | | 0.52219 | 0.285213 | 0.005419 | 0.53649 |
| AMB-00160027 | 0.6122 | 0.6503 | 0.254237 | 0.007171 | 0.58376 | 0.6276 | 0.70588 | 0.346350 | 0.006390 | 0.63772 | 0.4997 | 0.60062 | 0.204060 | 0.00792 | 0.60362 | 0.5533 | | 0.47059 | 0.269541 | 0.007171 | 0.54230 |
| AMB-01088947 | 0.4495 | 0.4722 | 0.200754 | 0.014776 | 0.58969 | 0.6120 | 0.52941 | 0.232214 | 0.005720 | 0.63554 | 0.4832 | 0.42105 | 0.174338 | 0.00862 | 0.61595 | 0.3900 | | 0.35294 | 0.167102 | 0.014776 | 0.54178 |
| AMB-00512257 | 0.4780 | 0.5000 | 0.215762 | 0.003125 | 0.58959 | 0.8227 | 0.76471 | 0.392407 | 0.006250 | 0.63808 | 0.3047 | 0.26316 | 0.101210 | 0.01447 | 0.61352 | 0.5910 | | 0.50981 | 0.268783 | 0.003125 | 0.54472 |
| est788 | 0.6435 | 0.6471 | 0.304877 | 0.001749 | 0.59338 | 0.5608 | 0.64706 | 0.304877 | 0.004940 | 0.63636 | 0.5715 | 0.64706 | 0.304877 | 0.00493 | 0.61551 | 0.5805 | | 0.43137 | 0.304978 | 0.001749 | 0.54932 |
| *est3880 | 0.7407 | 0.7729 | 0.341175 | 0.002623 | 0.58542 | 0.6541 | 0.67647 | 0.260280 | 0.003060 | 0.62529 | 0.8353 | 0.85913 | 0.440199 | 0.00317 | 0.61347 | 0.7033 | | 0.57275 | 0.322896 | 0.002623 | 0.53950 |
| AMB-00615374 | 0.6793 | 0.6944 | 0.337961 | 0.001729 | 0.59282 | 0.7735 | 0.70588 | 0.346350 | 0.001770 | 0.63711 | 0.7414 | 0.68421 | 0.330595 | 0.00423 | 0.61762 | 0.6197 | | 0.47059 | 0.280643 | 0.001729 | 0.54515 |
| est4582 | 0.6321 | 0.6650 | 0.280425 | 0.001149 | 0.58399 | 0.9596 | 0.97059 | 0.626369 | 0.000000 | 0.64226 | 0.3966 | 0.39164 | 0.126790 | 0.00119 | 0.60137 | 0.7410 | | 0.64706 | 0.421495 | 0.001149 | 0.54728 |
| ahb1232 | 0.5230 | 0.5294 | 0.232214 | 0.005839 | 0.59138 | 0.4339 | 0.52941 | 0.232214 | 0.005460 | 0.63441 | 0.4453 | 0.52941 | 0.232214 | 0.00593 | 0.61550 | 0.4541 | | 0.35294 | 0.235527 | 0.005839 | 0.54909 |
| AMB-00755023 | 0.4242 | 0.4412 | 0.184546 | 0.012678 | 0.58908 | 0.5695 | 0.46667 | 0.197811 | 0.008960 | 0.63509 | 0.4832 | 0.42105 | 0.174338 | 0.01678 | 0.61660 | 0.3601 | | 0.31111 | 0.150172 | 0.012678 | 0.54048 |
| ahb5160 | 0.6888 | 0.7075 | 0.312385 | 0.002917 | 0.58633 | 0.6619 | 0.73529 | 0.368731 | 0.003940 | 0.63788 | 0.6047 | 0.68266 | 0.278431 | 0.00647 | 0.60911 | 0.6315 | | 0.49019 | 0.314431 | 0.002917 | 0.54298 |
| AMB-00909475 | 0.3925 | 0.4167 | 0.172143 | 0.015265 | 0.59077 | 0.4910 | 0.41176 | 0.169703 | 0.012560 | 0.63610 | 0.4832 | 0.42105 | 0.174338 | 0.01324 | 0.61511 | 0.3247 | | 0.28070 | 0.137970 | 0.015265 | 0.54030 |
| *est9651 | 0.7746 | 0.8007 | 0.369252 | 0.002350 | 0.58562 | 0.7143 | 0.73529 | 0.307032 | 0.002790 | 0.62696 | 0.8353 | 0.85913 | 0.440199 | 0.00161 | 0.61263 | 0.7333 | | 0.57275 | 0.340663 | 0.002350 | 0.53927 |
| est5036 | 0.3712 | 0.3824 | 0.155322 | 0.012166 | 0.59049 | 0.2867 | 0.38235 | 0.155322 | 0.016630 | 0.63370 | 0.2974 | 0.38235 | 0.155322 | 0.01863 | 0.61444 | 0.3043 | | 0.25490 | 0.159777 | 0.012166 | 0.54865 |
| est8741 | 0.4495 | 0.4722 | 0.200754 | 0.005218 | 0.59144 | 0.2854 | 0.23529 | 0.089413 | 0.007450 | 0.63244 | 0.7414 | 0.68421 | 0.330595 | 0.01482 | 0.61711 | 0.5375 | | 0.45614 | 0.226523 | 0.005218 | 0.54446 |
| est294 | 0.6398 | 0.6895 | 0.270073 | 0.007333 | 0.58188 | 0.5265 | 0.55882 | 0.183849 | 0.008230 | 0.61993 | 0.7838 | 0.80650 | 0.375536 | 0.00679 | 0.60986 | 0.6083 | | 0.53767 | 0.261325 | 0.007333 | 0.53705 |
| AMB-00930503 | 0.6725 | 0.6944 | 0.337961 | 0.002018 | 0.59244 | 0.7134 | 0.64706 | 0.304877 | 0.001760 | 0.63657 | 0.7811 | 0.73684 | 0.369943 | 0.00300 | 0.61752 | 0.6133 | | 0.49123 | 0.281621 | 0.002018 | 0.54542 |
| ahb5836 | 0.7782 | 0.8023 | 0.376609 | 0.001638 | 0.58560 | 0.7082 | 0.70589 | 0.292838 | 0.002020 | 0.62924 | 0.8761 | 0.88855 | 0.478173 | 0.00097 | 0.61326 | 0.7455 | | 0.59237 | 0.351581 | 0.001638 | 0.54123 |
| *est10431 | 0.6176 | 0.6634 | 0.261701 | 0.008976 | 0.58120 | 0.5879 | 0.58824 | 0.212765 | 0.015810 | 0.62258 | 0.7268 | 0.73065 | 0.312410 | 0.01112 | 0.60769 | 0.5639 | | 0.48710 | 0.230603 | 0.008976 | 0.53390 |
| AMB-00427542 | 0.7333 | 0.7353 | 0.368731 | 0.000320 | 0.59331 | 0.6619 | 0.73529 | 0.368731 | 0.000730 | 0.63687 | 0.6713 | 0.73529 | 0.368731 | 0.00045 | 0.61668 | 0.6795 | | 0.49019 | 0.364245 | 0.000320 | 0.55019 |
| AMB-00681715 | 0.6734 | 0.6765 | 0.325107 | 0.002083 | 0.59286 | 0.5939 | 0.67647 | 0.325107 | 0.003230 | 0.63689 | 0.6043 | 0.67647 | 0.325107 | 0.00170 | 0.61586 | 0.6131 | | 0.45098 | 0.323936 | 0.002083 | 0.54901 |
| ahb390 | 0.5833 | 0.5882 | 0.267050 | 0.003663 | 0.59183 | 0.4963 | 0.58824 | 0.267050 | 0.003760 | 0.63572 | 0.5075 | 0.58824 | 0.267050 | 0.00450 | 0.61714 | 0.5165 | | 0.39216 | 0.269086 | 0.003663 | 0.54824 |
| est1480 | 0.3925 | 0.4167 | 0.172143 | 0.014800 | 0.58904 | 0.7221 | 0.64706 | 0.304877 | 0.010440 | 0.63686 | 0.2406 | 0.21053 | 0.079169 | 0.00374 | 0.61426 | 0.4941 | | 0.43137 | 0.210437 | 0.014800 | 0.54345 |
| est4055 | 0.6176 | 0.6634 | 0.261701 | 0.009392 | 0.58036 | 0.5879 | 0.58824 | 0.212765 | 0.009590 | 0.62498 | 0.7268 | 0.73065 | 0.312410 | 0.01148 | 0.60778 | 0.5639 | | 0.48710 | 0.230603 | 0.009392 | 0.53246 |
| est4292 | 0.7543 | 0.7762 | 0.366819 | 0.002473 | 0.58728 | 0.8164 | 0.79412 | 0.382949 | 0.001690 | 0.63156 | 0.7791 | 0.76006 | 0.352983 | 0.00434 | 0.61092 | 0.7039 | | 0.52941 | 0.317519 | 0.002473 | 0.53837 |
| ahb4083 | 0.8106 | 0.8301 | 0.405504 | 0.000789 | 0.58616 | 0.8186 | 0.82353 | 0.398452 | 0.000880 | 0.63155 | 0.8279 | 0.83592 | 0.411962 | 0.00179 | 0.61170 | 0.7682 | | 0.55728 | 0.360383 | 0.000789 | 0.53914 |
| AMB-00044887 | 0.7136 | 0.7467 | 0.325761 | 0.002612 | 0.58315 | 0.8703 | 0.88236 | 0.469429 | 0.003930 | 0.63328 | 0.6190 | 0.62539 | 0.236072 | 0.00292 | 0.60438 | 0.6892 | | 0.58824 | 0.328557 | 0.002612 | 0.53808 |
| est3908 | 0.6625 | 0.6928 | 0.300212 | 0.006435 | 0.58445 | 0.7099 | 0.67647 | 0.288447 | 0.004880 | 0.62936 | 0.7298 | 0.70743 | 0.311061 | 0.00786 | 0.60880 | 0.6017 | | 0.47162 | 0.254700 | 0.006435 | 0.53543 |
| AMB-00684734 | 0.6231 | 0.6807 | 0.254524 | 0.007971 | 0.58182 | 0.5726 | 0.64706 | 0.227148 | 0.009090 | 0.62439 | 0.6486 | 0.71242 | 0.283594 | 0.01975 | 0.60697 | 0.5621 | | 0.47495 | 0.232209 | 0.007971 | 0.53192 |
| *est6625 | 0.7372 | 0.7500 | 0.380396 | 0.000000 | 0.59280 | 0.7735 | 0.70588 | 0.346350 | 0.000000 | 0.63749 | 0.8331 | 0.78947 | 0.413493 | 0.00000 | 0.61913 | 0.6873 | | 0.52631 | 0.319659 | 0.000000 | 0.54619 |
| est3686 | 0.5411 | 0.5817 | 0.226596 | 0.015538 | 0.58229 | 0.3975 | 0.38235 | 0.122559 | 0.007330 | 0.62245 | 0.7791 | 0.76006 | 0.352983 | 0.01211 | 0.60918 | 0.5610 | | 0.50671 | 0.235324 | 0.015538 | 0.53731 |
| *est2829 | 0.6017 | 0.6373 | 0.261622 | 0.016588 | 0.58374 | 0.5934 | 0.55883 | 0.213063 | 0.002640 | 0.62714 | 0.7298 | 0.70743 | 0.311061 | 0.02453 | 0.60957 | 0.5487 | | 0.47162 | 0.226261 | 0.016588 | 0.53529 |
| est1873 | 0.6793 | 0.6944 | 0.337961 | 0.001749 | 0.59212 | 0.8227 | 0.76471 | 0.392407 | 0.001880 | 0.63730 | 0.6932 | 0.63158 | 0.294600 | 0.00176 | 0.61682 | 0.6291 | | 0.50981 | 0.289520 | 0.001749 | 0.54404 |
| AMB-00557876 | 0.5990 | 0.6431 | 0.262605 | 0.012415 | 0.58554 | 0.5939 | 0.67647 | 0.325107 | 0.004890 | 0.63580 | 0.4775 | 0.59955 | 0.212079 | 0.00328 | 0.60457 | 0.5447 | | 0.45098 | 0.263344 | 0.012415 | 0.54246 |
| AMB-00612703 | 0.4495 | 0.4722 | 0.200754 | 0.013077 | 0.59113 | 0.5530 | 0.47059 | 0.199887 | 0.010750 | 0.63559 | 0.5385 | 0.47368 | 0.201532 | 0.00337 | 0.61483 | 0.3802 | | 0.31579 | 0.161795 | 0.013077 | 0.54096 |
| AMB-00711538 | 0.7770 | 0.7974 | 0.372889 | 0.000570 | 0.58611 | 0.8053 | 0.85294 | 0.473508 | 0.000570 | 0.63832 | 0.6982 | 0.74768 | 0.315239 | 0.00058 | 0.60834 | 0.7343 | | 0.56863 | 0.375061 | 0.000570 | 0.54538 |
| AMB-00350114 | 0.4209 | 0.4444 | 0.186225 | 0.010092 | 0.58974 | 0.1289 | 0.11765 | 0.042613 | 0.002200 | 0.63293 | 0.7880 | 0.73684 | 0.369943 | 0.00311 | 0.61804 | 0.6519 | | 0.49123 | 0.284609 | 0.010092 | 0.54561 |
| *AMB-00263182 | 0.6561 | 0.6849 | 0.294452 | 0.004346 | 0.58488 | 0.7643 | 0.73530 | 0.332658 | 0.005990 | 0.63065 | 0.6667 | 0.63726 | 0.261622 | 0.00821 | 0.60931 | 0.6005 | | 0.49020 | 0.254539 | 0.004346 | 0.53519 |
| AMB-00325337 | 0.5928 | 0.6354 | 0.259012 | 0.010093 | 0.58408 | 0.7557 | 0.73346 | 0.329856 | 0.005560 | 0.62887 | 0.5595 | 0.54770 | 0.205278 | 0.00727 | 0.60642 | 0.5436 | | 0.48897 | 0.234571 | 0.010093 | 0.53468 |
| *est9669 | 0.4625 | 0.4706 | 0.199887 | 0.005360 | 0.59059 | 0.3736 | 0.47059 | 0.199887 | 0.007060 | 0.63448 | 0.3849 | 0.47059 | 0.199887 | 0.00914 | 0.61560 | 0.3931 | | 0.31373 | 0.203959 | 0.005360 | 0.54794 |
| ahb7820 | 0.5941 | 0.6193 | 0.251219 | 0.009072 | 0.58468 | 0.4818 | 0.58824 | 0.212765 | 0.010010 | 0.62615 | 0.5715 | 0.64706 | 0.304877 | 0.00200 | 0.61747 | 0.5300 | | 0.43137 | 0.254606 | 0.009072 | 0.54237 |
| est2905 | 0.4625 | 0.4706 | 0.199887 | 0.005389 | 0.59059 | 0.3736 | 0.47059 | 0.199887 | 0.006670 | 0.63503 | 0.3849 | 0.47059 | 0.199887 | 0.01100 | 0.61495 | 0.3931 | | 0.31373 | 0.203959 | 0.005389 | 0.54703 |
| est507 | 0.2790 | 0.3056 | 0.119748 | 0.012625 | 0.58915 | 0.7221 | 0.64706 | 0.304877 | 0.006460 | 0.63746 | -0.0500 | 0.00000 | 0.000000 | 0.00000 | 0.62271 | 0.6731 | | 0.43137 | 0.304978 | 0.012625 | 0.55173 |
| ahb7245 | 0.7237 | 0.7484 | 0.343244 | 0.001961 | 0.58632 | 0.8662 | 0.85294 | 0.441798 | 0.001780 | 0.63279 | 0.6788 | 0.65480 | 0.273388 | 0.00438 | 0.60891 | 0.6878 | | 0.56863 | 0.318247 | 0.001961 | 0.53925 |
| ahb10289 | 0.7033 | 0.7435 | 0.310346 | 0.002931 | 0.58466 | 0.6020 | 0.64706 | 0.232308 | 0.004580 | 0.62448 | 0.7951 | 0.82972 | 0.406831 | 0.00417 | 0.61178 | 0.6621 | | 0.55315 | 0.297457 | 0.002931 | 0.53819 |
| *ahb9783 | 0.8158 | 0.8317 | 0.419326 | 0.000000 | 0.58828 | 0.8662 | 0.85294 | 0.441798 | 0.000000 | 0.63215 | 0.8268 | 0.81270 | 0.400444 | 0.00000 | 0.61175 | 0.7750 | | 0.56863 | 0.368401 | 0.000000 | 0.54035 |
| AMB-00901979 | 0.6888 | 0.7075 | 0.312385 | 0.003046 | 0.58638 | 0.5900 | 0.67647 | 0.270981 | 0.002910 | 0.62681 | 0.6713 | 0.73529 | 0.368731 | 0.00374 | 0.61637 | 0.6319 | | 0.49019 | 0.310709 | 0.003046 | 0.54394 |
| AMB-00767729 | 0.5956 | 0.6536 | 0.238203 | 0.005137 | 0.58175 | 0.6968 | 0.76471 | 0.392407 | 0.005950 | 0.63708 | 0.4455 | 0.55418 | 0.162717 | 0.00544 | 0.59950 | 0.5537 | | 0.50981 | 0.277222 | 0.005137 | 0.54265 |
| AMB-00459536 | 0.5928 | 0.6111 | 0.281374 | 0.003254 | 0.59135 | 0.8227 | 0.76471 | 0.392407 | 0.001620 | 0.63766 | 0.5385 | 0.47368 | 0.201532 | 0.00242 | 0.61514 | 0.5785 | | 0.50981 | 0.265408 | 0.003254 | 0.54376 |
| *ahb9405 | 0.3925 | 0.4167 | 0.172143 | 0.012844 | 0.58926 | 0.6120 | 0.52941 | 0.232214 | 0.012890 | 0.63612 | 0.3665 | 0.31579 | 0.124335 | 0.00498 | 0.61326 | 0.3674 | | 0.35294 | 0.156226 | 0.012844 | 0.54085 |
| ahb142 | 0.5625 | 0.5899 | 0.232830 | 0.007811 | 0.58447 | 0.5283 | 0.61765 | 0.285554 | 0.008150 | 0.63575 | 0.4640 | 0.56502 | 0.202060 | 0.00602 | 0.60612 | 0.4966 | | 0.41177 | 0.240745 | 0.007811 | 0.54128 |
| AMB-01112346 | 0.5714 | 0.6095 | 0.243706 | 0.009107 | 0.58362 | 0.6530 | 0.61765 | 0.248888 | 0.011350 | 0.62588 | 0.6260 | 0.60217 | 0.239128 | 0.00983 | 0.60682 | 0.5042 | | 0.41177 | 0.204920 | 0.009107 | 0.53294 |
| AMB-00921628 | 0.6625 | 0.6928 | 0.300212 | 0.004672 | 0.58472 | 0.5934 | 0.55883 | 0.213063 | 0.000230 | 0.62589 | 0.8268 | 0.81270 | 0.400444 | 0.00792 | 0.61196 | 0.6351 | | 0.54180 | 0.277338 | 0.004672 | 0.53912 |
| AMB-00834852 | 0.6257 | 0.6487 | 0.270536 | 0.002309 | 0.58523 | 0.5171 | 0.61765 | 0.231082 | 0.009360 | 0.62546 | 0.6043 | 0.67647 | 0.325107 | 0.00896 | 0.61711 | 0.5635 | | 0.45098 | 0.272479 | 0.002309 | 0.54359 |
| ahb10360 | 0.5175 | 0.6013 | 0.194247 | 0.026923 | 0.57767 | 0.3634 | 0.47059 | 0.119871 | 0.012970 | 0.61550 | 0.6568 | 0.71827 | 0.289362 | 0.01609 | 0.60861 | 0.4836 | | 0.47885 | 0.198608 | 0.026923 | 0.52962 |
| *est11799 | 0.5792 | 0.6209 | 0.234769 | 0.007979 | 0.58359 | 0.4404 | 0.55882 | 0.175945 | 0.007660 | 0.62032 | 0.6043 | 0.67647 | 0.325107 | 0.00350 | 0.61652 | 0.5204 | | 0.45098 | 0.247351 | 0.007979 | 0.54171 |
| *est2177 | 0.4701 | 0.5000 | 0.215762 | 0.004936 | 0.59103 | 0.6587 | 0.58824 | 0.267050 | 0.005520 | 0.63742 | 0.4725 | 0.42105 | 0.174338 | 0.00612 | 0.61431 | 0.4193 | | 0.39216 | 0.185931 | 0.004936 | 0.54111 |
| *est1600 | 0.4780 | 0.5000 | 0.215762 | 0.002993 | 0.59146 | 0.2093 | 0.17647 | 0.065436 | 0.002440 | 0.63406 | 0.8331 | 0.78947 | 0.413493 | 0.00245 | 0.61800 | 0.6711 | | 0.52631 | 0.301470 | 0.002993 | 0.54678 |
| est11 | 0.7543 | 0.7762 | 0.366819 | 0.000215 | 0.58689 | 0.9139 | 0.91177 | 0.514510 | 0.000000 | 0.63568 | 0.6788 | 0.65480 | 0.273388 | 0.00045 | 0.60784 | 0.7339 | | 0.60785 | 0.361125 | 0.000215 | 0.54096 |
| *est8331 | 0.4495 | 0.4722 | 0.200754 | 0.004488 | 0.58947 | 0.2854 | 0.23529 | 0.089413 | 0.008370 | 0.63432 | 0.7414 | 0.68421 | 0.330595 | 0.00401 | 0.61801 | 0.5375 | | 0.45614 | 0.226523 | 0.004488 | 0.54261 |
| est9606 | 0.6366 | 0.6814 | 0.265341 | 0.003120 | 0.58328 | 0.6968 | 0.76471 | 0.392407 | 0.003250 | 0.63676 | 0.5111 | 0.60682 | 0.199270 | 0.00703 | 0.60382 | 0.5864 | | 0.50981 | 0.289861 | 0.003120 | 0.54404 |
| ahb7542 | 0.6017 | 0.6373 | 0.261622 | 0.007249 | 0.58331 | 0.5934 | 0.55883 | 0.213063 | 0.008150 | 0.62605 | 0.7298 | 0.70743 | 0.311061 | 0.00532 | 0.60987 | 0.5487 | | 0.47162 | 0.226261 | 0.007249 | 0.53288 |
| AMB-00940641 | 0.6815 | 0.7190 | 0.303025 | 0.001715 | 0.58325 | 0.8703 | 0.88236 | 0.469429 | 0.002680 | 0.63350 | 0.5626 | 0.57276 | 0.203511 | 0.00507 | 0.60399 | 0.6695 | | 0.58824 | 0.320780 | 0.001715 | 0.53916 |
| *AMB-00692527 | 0.6105 | 0.6389 | 0.299423 | 0.001963 | 0.59299 | 0.5962 | 0.52941 | 0.232214 | 0.002080 | 0.63554 | 0.7775 | 0.73684 | 0.369943 | 0.00208 | 0.61785 | 0.5662 | | 0.49123 | 0.258373 | 0.001963 | 0.54507 |
| *ahb11439 | 0.5532 | 0.5588 | 0.249292 | 0.002486 | 0.59091 | 0.4648 | 0.55882 | 0.249292 | 0.003780 | 0.63507 | 0.4762 | 0.55882 | 0.249292 | 0.00606 | 0.61561 | 0.4851 | | 0.37255 | 0.252039 | 0.002486 | 0.54883 |
| AMB-00584678 | 0.4927 | 0.5000 | 0.215762 | 0.004575 | 0.59036 | 0.4035 | 0.50000 | 0.215762 | 0.005190 | 0.63518 | 0.4149 | 0.50000 | 0.215762 | 0.00546 | 0.61356 | 0.4234 | | 0.33333 | 0.219512 | 0.004575 | 0.54816 |
| AMB-00723724 | 0.4927 | 0.5000 | 0.215762 | 0.003587 | 0.59234 | 0.4035 | 0.50000 | 0.215762 | 0.003630 | 0.63447 | 0.4149 | 0.50000 | 0.215762 | 0.00978 | 0.61546 | 0.4234 | | 0.33333 | 0.219512 | 0.003587 | 0.54767 |
| ahb5948 | 0.5680 | 0.6226 | 0.224448 | 0.007874 | 0.58301 | 0.4783 | 0.58823 | 0.193490 | 0.013520 | 0.62077 | 0.5686 | 0.65325 | 0.257704 | 0.01803 | 0.60783 | 0.5030 | | 0.43550 | 0.212580 | 0.007874 | 0.53463 |
| AMB-00126062 | 0.6215 | 0.6389 | 0.299423 | 0.003345 | 0.59231 | 0.7221 | 0.64706 | 0.304877 | 0.000000 | 0.63700 | 0.6932 | 0.63158 | 0.294600 | 0.00210 | 0.61816 | 0.5572 | | 0.43137 | 0.246432 | 0.003345 | 0.54365 |
| est6069 | 0.2790 | 0.3056 | 0.119748 | 0.005434 | 0.58854 | 0.7221 | 0.64706 | 0.304877 | 0.005960 | 0.63774 | -0.0500 | 0.00000 | 0.000000 | 0.00220 | 0.62403 | 0.6731 | | 0.43137 | 0.304978 | 0.005434 | 0.55225 |
| *est8926 | 0.6625 | 0.6928 | 0.300212 | 0.004584 | 0.58481 | 0.8164 | 0.79412 | 0.382949 | 0.005730 | 0.63076 | 0.6260 | 0.60217 | 0.239128 | 0.00170 | 0.60804 | 0.6207 | | 0.52941 | 0.274160 | 0.004584 | 0.53673 |
| AMB-00625908 | 0.4209 | 0.4444 | 0.186225 | 0.007398 | 0.58979 | 0.4259 | 0.35294 | 0.141375 | 0.006380 | 0.63371 | 0.5919 | 0.52632 | 0.230453 | 0.00638 | 0.61559 | 0.3787 | | 0.35088 | 0.157888 | 0.007398 | 0.54137 |
| AMB-00352891 | 0.5838 | 0.6345 | 0.240333 | 0.003575 | 0.58275 | 0.5967 | 0.69697 | 0.339799 | 0.005490 | 0.63617 | 0.4892 | 0.59171 | 0.198509 | 0.01154 | 0.60412 | 0.5283 | | 0.46465 | 0.264031 | 0.003575 | 0.54187 |
| AMB-01082776 | 0.4991 | 0.5310 | 0.198510 | 0.015328 | 0.58400 | 0.3800 | 0.50000 | 0.163192 | 0.001950 | 0.62333 | 0.4762 | 0.55882 | 0.249292 | 0.01588 | 0.61509 | 0.4323 | | 0.37255 | 0.205140 | 0.015328 | 0.54332 |
| ahb2194 | 0.6065 | 0.6618 | 0.249620 | 0.007693 | 0.58261 | 0.7722 | 0.79411 | 0.362391 | 0.003030 | 0.62924 | 0.5027 | 0.54334 | 0.175092 | 0.00528 | 0.60182 | 0.5748 | | 0.52941 | 0.251240 | 0.007693 | 0.53366 |
| *est6461 | 0.5913 | 0.6569 | 0.234819 | 0.005574 | 0.57994 | 0.3634 | 0.47059 | 0.119871 | 0.002760 | 0.61551 | 0.7757 | 0.82353 | 0.444495 | 0.00170 | 0.61833 | 0.5906 | | 0.54902 | 0.297954 | 0.005574 | 0.54463 |
| *est6400 | 0.7441 | 0.7680 | 0.345540 | 0.002385 | 0.58549 | 0.7051 | 0.76471 | 0.341352 | 0.002080 | 0.63016 | 0.7169 | 0.77090 | 0.349364 | 0.00246 | 0.61109 | 0.6917 | | 0.51393 | 0.321787 | 0.002385 | 0.53833 |
| *est7498 | 0.4711 | 0.5243 | 0.192159 | 0.018480 | 0.58258 | 0.3133 | 0.32169 | 0.095093 | 0.015620 | 0.62258 | 0.7206 | 0.70559 | 0.308313 | 0.00494 | 0.61002 | 0.5009 | | 0.47039 | 0.206003 | 0.018480 | 0.53320 |
| *est3866 | 0.5532 | 0.5588 | 0.249292 | 0.003220 | 0.59210 | 0.4648 | 0.55882 | 0.249292 | 0.002780 | 0.63420 | 0.4762 | 0.55882 | 0.249292 | 0.00406 | 0.61587 | 0.4851 | | 0.37255 | 0.252039 | 0.003220 | 0.54769 |
| est2200 | 0.5230 | 0.5294 | 0.232214 | 0.002738 | 0.59046 | 0.4339 | 0.52941 | 0.232214 | 0.009610 | 0.63498 | 0.4453 | 0.52941 | 0.232214 | 0.00313 | 0.61527 | 0.4541 | | 0.35294 | 0.235527 | 0.002738 | 0.54769 |
| ahb4399 | 0.7543 | 0.7762 | 0.366819 | 0.001697 | 0.58621 | 0.8164 | 0.79412 | 0.382949 | 0.002050 | 0.63083 | 0.7791 | 0.76006 | 0.352983 | 0.00091 | 0.61024 | 0.7039 | | 0.52941 | 0.317519 | 0.001697 | 0.53939 |
| *est10 | 0.7069 | 0.7451 | 0.315570 | 0.000137 | 0.58431 | 0.8810 | 0.91176 | 0.540031 | 0.000000 | 0.63899 | 0.5620 | 0.59597 | 0.205983 | 0.00024 | 0.60462 | 0.6974 | | 0.60784 | 0.369197 | 0.000137 | 0.54541 |
| est7027 | 0.7237 | 0.7484 | 0.343244 | 0.001732 | 0.58687 | 0.6530 | 0.61765 | 0.248888 | 0.001670 | 0.62865 | 0.8730 | 0.86533 | 0.455674 | 0.00094 | 0.61304 | 0.6984 | | 0.57689 | 0.319208 | 0.001732 | 0.53972 |
| est5155 | 0.7850 | 0.8039 | 0.392072 | 0.000000 | 0.58797 | 0.8662 | 0.85294 | 0.441798 | 0.000000 | 0.63345 | 0.7791 | 0.76006 | 0.352983 | 0.00000 | 0.60995 | 0.7424 | | 0.56863 | 0.347602 | 0.000000 | 0.53995 |
| est9938 | 0.5641 | 0.6324 | 0.222090 | 0.012908 | 0.57947 | 0.4687 | 0.52941 | 0.159475 | 0.015350 | 0.61898 | 0.6844 | 0.72446 | 0.293151 | 0.00334 | 0.60777 | 0.5177 | | 0.48297 | 0.208069 | 0.012908 | 0.53034 |
| *ahb6844 | 0.5133 | 0.5621 | 0.198874 | 0.005296 | 0.58169 | 0.3676 | 0.50000 | 0.143984 | 0.010310 | 0.61852 | 0.5393 | 0.61765 | 0.285554 | 0.00866 | 0.61596 | 0.4533 | | 0.41177 | 0.214414 | 0.005296 | 0.54264 |
| est5796 | 0.5680 | 0.6226 | 0.224448 | 0.010260 | 0.58216 | 0.4783 | 0.58823 | 0.193490 | 0.009900 | 0.62077 | 0.5686 | 0.65325 | 0.257704 | 0.00497 | 0.60907 | 0.5030 | | 0.43550 | 0.212580 | 0.010260 | 0.53438 |
| AMB-00310216 | 0.4669 | 0.4881 | 0.147436 | 0.009935 | 0.57751 | 0.2414 | 0.39496 | 0.086571 | 0.008810 | 0.61230 | 0.5858 | 0.57143 | 0.256816 | 0.01578 | 0.61600 | 0.3692 | | 0.38095 | 0.178595 | 0.009935 | 0.54127 |
| AMB-00270786 | 0.4321 | 0.4412 | 0.184546 | 0.008320 | 0.59207 | 0.3442 | 0.44118 | 0.184546 | 0.000000 | 0.63332 | 0.3553 | 0.44118 | 0.184546 | 0.01274 | 0.61499 | 0.3631 | | 0.29412 | 0.188836 | 0.008320 | 0.54761 |
| ahb4473 | 0.6017 | 0.6373 | 0.261622 | 0.005791 | 0.58405 | 0.4658 | 0.44118 | 0.150281 | 0.003090 | 0.62445 | 0.8268 | 0.81270 | 0.400444 | 0.00274 | 0.61203 | 0.6138 | | 0.54180 | 0.267507 | 0.005791 | 0.53800 |
| ahb11091 | 0.6733 | 0.7173 | 0.291977 | 0.002493 | 0.58445 | 0.5265 | 0.55882 | 0.183849 | 0.002100 | 0.62188 | 0.8353 | 0.85913 | 0.440199 | 0.00254 | 0.61257 | 0.6575 | | 0.57275 | 0.301089 | 0.002493 | 0.53975 |
| AMB-00129763 | 0.5680 | 0.6226 | 0.224448 | 0.004195 | 0.58212 | 0.6276 | 0.70588 | 0.346350 | 0.006020 | 0.63639 | 0.4311 | 0.54799 | 0.162860 | 0.00560 | 0.59987 | 0.5150 | | 0.47059 | 0.252964 | 0.004195 | 0.54202 |
| est3328 | 0.7782 | 0.8023 | 0.376609 | 0.000616 | 0.58635 | 0.8186 | 0.82353 | 0.398452 | 0.000590 | 0.63138 | 0.7782 | 0.78329 | 0.358291 | 0.00085 | 0.61032 | 0.7313 | | 0.54902 | 0.334510 | 0.000616 | 0.53815 |
| ahb5182 | 0.6669 | 0.7124 | 0.283594 | 0.003389 | 0.58289 | 0.5726 | 0.64706 | 0.227148 | 0.005750 | 0.62318 | 0.7169 | 0.77090 | 0.349364 | 0.00441 | 0.61029 | 0.6138 | | 0.51393 | 0.268591 | 0.003389 | 0.53517 |
| ahb3219 | 0.6022 | 0.6520 | 0.244229 | 0.007142 | 0.58159 | 0.5171 | 0.61764 | 0.212229 | 0.007520 | 0.62286 | 0.6047 | 0.68266 | 0.278431 | 0.00409 | 0.60805 | 0.5388 | | 0.45511 | 0.230322 | 0.007142 | 0.53448 |
| *ahb3777 | 0.5920 | 0.6552 | 0.235206 | 0.005905 | 0.58086 | 0.5283 | 0.61765 | 0.205490 | 0.006960 | 0.62257 | 0.6162 | 0.68886 | 0.265604 | 0.00826 | 0.60596 | 0.5285 | | 0.45924 | 0.215637 | 0.005905 | 0.53139 |
| *ahb8772 | 0.3640 | 0.3889 | 0.158479 | 0.005170 | 0.58979 | 0.0439 | 0.05882 | 0.020832 | 0.003520 | 0.63358 | 0.7414 | 0.68421 | 0.330595 | 0.00272 | 0.61779 | 0.6433 | | 0.45614 | 0.277312 | 0.005170 | 0.54615 |
| ahb3403 | 0.7237 | 0.7484 | 0.343244 | 0.001349 | 0.58590 | 0.6530 | 0.61765 | 0.248888 | 0.001440 | 0.62605 | 0.8730 | 0.86533 | 0.455674 | 0.00053 | 0.61321 | 0.6984 | | 0.57689 | 0.319208 | 0.001349 | 0.53972 |
| *ahb3774 | 0.4321 | 0.4412 | 0.184546 | 0.004421 | 0.59100 | 0.3442 | 0.44118 | 0.184546 | 0.004660 | 0.63532 | 0.3553 | 0.44118 | 0.184546 | 0.00981 | 0.61290 | 0.3631 | | 0.29412 | 0.188836 | 0.004421 | 0.54632 |
| ahb546 | 0.4542 | 0.5227 | 0.174433 | 0.008466 | 0.57707 | 0.8149 | 0.82174 | 0.396098 | 0.007470 | 0.63055 | 0.1874 | 0.25518 | 0.057456 | 0.00339 | 0.59519 | 0.5884 | | 0.54783 | 0.275325 | 0.008466 | 0.53870 |
| AMB-00182422 | 0.2790 | 0.3056 | 0.119748 | 0.009601 | 0.58878 | 0.0439 | 0.05882 | 0.020832 | 0.004210 | 0.63346 | 0.5919 | 0.52632 | 0.230453 | 0.01212 | 0.61586 | 0.4776 | | 0.35088 | 0.188298 | 0.009601 | 0.54371 |
| est4575 | 0.2807 | 0.4210 | 0.091713 | 0.005887 | 0.56936 | -0.0266 | 0.08823 | 0.004571 | 0.019880 | 0.59426 | 0.6667 | 0.73529 | 0.368731 | 0.00742 | 0.61712 | 0.4812 | | 0.49019 | 0.281012 | 0.005887 | 0.54631 |
| *est2678 | 0.5858 | 0.6356 | 0.242784 | 0.005944 | 0.58098 | 0.7646 | 0.76471 | 0.341352 | 0.008040 | 0.62774 | 0.5045 | 0.52013 | 0.173853 | 0.00359 | 0.60124 | 0.5530 | | 0.50981 | 0.236032 | 0.005944 | 0.53311 |
| AMB-00642179 | 0.6122 | 0.6503 | 0.254237 | 0.002924 | 0.58291 | 0.6276 | 0.70588 | 0.346350 | 0.001840 | 0.63676 | 0.4997 | 0.60062 | 0.204060 | 0.01115 | 0.60446 | 0.5533 | | 0.47059 | 0.269541 | 0.002924 | 0.54164 |
| AMB-01019289 | 0.5376 | 0.5882 | 0.267050 | 0.002954 | 0.59188 | 0.5823 | 0.53333 | 0.234454 | 0.002890 | 0.63608 | 0.6489 | 0.63158 | 0.294600 | 0.00146 | 0.61637 | 0.4601 | | 0.42105 | 0.218306 | 0.002954 | 0.54303 |
| est11641 | 0.6321 | 0.6650 | 0.280425 | 0.004384 | 0.58419 | 0.6530 | 0.61765 | 0.248888 | 0.005450 | 0.62694 | 0.7298 | 0.70743 | 0.311061 | 0.00321 | 0.60992 | 0.5728 | | 0.47162 | 0.238718 | 0.004384 | 0.53407 |
| AMB-00417207 | 0.4209 | 0.4444 | 0.186225 | 0.006654 | 0.59006 | 0.2854 | 0.23529 | 0.089413 | 0.006960 | 0.63240 | 0.6932 | 0.63158 | 0.294600 | 0.00117 | 0.61650 | 0.4846 | | 0.42105 | 0.199981 | 0.006654 | 0.54295 |
| AMB-00140870 | 0.5505 | 0.5974 | 0.218383 | 0.010790 | 0.58171 | 0.4865 | 0.59375 | 0.214416 | 0.012410 | 0.62469 | 0.5043 | 0.60069 | 0.221999 | 0.00382 | 0.60670 | 0.4816 | | 0.40046 | 0.207115 | 0.010790 | 0.53157 |
| *est3910 | 0.6249 | 0.6563 | 0.274414 | 0.002773 | 0.58407 | 0.8103 | 0.78309 | 0.372964 | 0.005850 | 0.63038 | 0.5715 | 0.54954 | 0.207704 | 0.00361 | 0.60652 | 0.5922 | | 0.52206 | 0.259789 | 0.002773 | 0.53660 |
| *est4599 | 0.5617 | 0.6285 | 0.225131 | 0.000932 | 0.58068 | 0.2323 | 0.31801 | 0.071032 | 0.001040 | 0.61510 | 0.8804 | 0.90625 | 0.533182 | 0.00149 | 0.61994 | 0.6775 | | 0.60417 | 0.363599 | 0.000932 | 0.54764 |
| ahb7581 | 0.5133 | 0.5621 | 0.198874 | 0.010244 | 0.58246 | 0.4471 | 0.55883 | 0.195397 | 0.011370 | 0.62377 | 0.4640 | 0.56502 | 0.202060 | 0.00732 | 0.60586 | 0.4442 | | 0.37668 | 0.189514 | 0.010244 | 0.53178 |
| AMB-01154531 | 0.5500 | 0.6244 | 0.212263 | 0.055885 | 0.57814 | 0.4636 | 0.54044 | 0.160069 | 0.153290 | 0.61579 | 0.6394 | 0.69505 | 0.266221 | 0.00137 | 0.60658 | 0.4966 | | 0.46337 | 0.194588 | 0.055885 | 0.52803 |
| AMB-00404788 | 0.5109 | 0.5539 | 0.210225 | 0.009444 | 0.58177 | 0.3975 | 0.38235 | 0.122559 | 0.011090 | 0.62255 | 0.7298 | 0.70743 | 0.311061 | 0.00277 | 0.60892 | 0.5112 | | 0.47162 | 0.207755 | 0.009444 | 0.53424 |
| ahb906 | 0.7459 | 0.7745 | 0.350181 | 0.001479 | 0.58475 | 0.7646 | 0.76471 | 0.341352 | 0.001180 | 0.62951 | 0.7782 | 0.78329 | 0.358291 | 0.00209 | 0.61049 | 0.6939 | | 0.52219 | 0.306616 | 0.001479 | 0.53726 |
| *est7297 | 0.3316 | 0.4167 | 0.113646 | 0.013050 | 0.57583 | 0.1594 | 0.32353 | 0.060285 | 0.014820 | 0.60894 | 0.4149 | 0.50000 | 0.215762 | 0.01636 | 0.61569 | 0.2922 | | 0.33333 | 0.147387 | 0.013050 | 0.54061 |
| ahb694 | 0.3292 | 0.3900 | 0.108452 | 0.592140 | 0.57861 | 0.3514 | 0.45455 | 0.191455 | 0.585760 | 0.63441 | 0.2184 | 0.34929 | 0.080110 | 0.45252 | 0.59669 | 0.2601 | | 0.30303 | 0.139424 | 0.592140 | 0.53921 |
| AMB-00703267 | 0.3925 | 0.4167 | 0.172143 | 0.007112 | 0.58867 | 0.6683 | 0.58824 | 0.267050 | 0.006680 | 0.63674 | 0.3047 | 0.26316 | 0.101210 | 0.00177 | 0.61382 | 0.4216 | | 0.39216 | 0.178562 | 0.007112 | 0.54049 |
| AMB-00811503 | 0.5245 | 0.6029 | 0.197885 | 0.010563 | 0.57813 | 0.4492 | 0.56250 | 0.168736 | 0.007370 | 0.61760 | 0.5488 | 0.63889 | 0.227887 | 0.00857 | 0.60475 | 0.4582 | | 0.42593 | 0.182578 | 0.010563 | 0.52884 |
| AMB-00483604 | 0.5714 | 0.6095 | 0.243706 | 0.002670 | 0.58230 | 0.8662 | 0.85294 | 0.441798 | 0.002810 | 0.63316 | 0.3966 | 0.39164 | 0.126790 | 0.00463 | 0.60193 | 0.6274 | | 0.56863 | 0.295079 | 0.002670 | 0.53850 |
| AMB-00825543 | 0.6733 | 0.7173 | 0.291977 | 0.002191 | 0.58284 | 0.7722 | 0.79411 | 0.362391 | 0.001870 | 0.62948 | 0.6198 | 0.64860 | 0.240384 | 0.00712 | 0.60472 | 0.6235 | | 0.52941 | 0.271228 | 0.002191 | 0.53480 |
| AMB-00893621 | 0.5650 | 0.6111 | 0.281374 | 0.000729 | 0.59231 | 0.4496 | 0.41176 | 0.169703 | 0.000760 | 0.63555 | 0.8112 | 0.78947 | 0.413493 | 0.00157 | 0.61881 | 0.5770 | | 0.52631 | 0.275796 | 0.000729 | 0.54492 |
| *AMB-00644533 | 0.3712 | 0.3824 | 0.155322 | 0.004806 | 0.59028 | 0.2867 | 0.38235 | 0.155322 | 0.007700 | 0.63423 | 0.2974 | 0.38235 | 0.155322 | 0.00674 | 0.61427 | 0.3043 | | 0.25490 | 0.159777 | 0.004806 | 0.54581 |
| ahb1201 | 0.3075 | 0.4199 | 0.102160 | 0.012457 | 0.57601 | 0.0909 | 0.26470 | 0.036291 | 0.013890 | 0.60014 | 0.4762 | 0.55882 | 0.249292 | 0.00859 | 0.61615 | 0.3084 | | 0.37255 | 0.166312 | 0.012457 | 0.54195 |
| ahb7653 | 0.3925 | 0.4167 | 0.172143 | 0.003661 | 0.58999 | 0.2093 | 0.17647 | 0.065436 | 0.002970 | 0.63317 | 0.6932 | 0.63158 | 0.294600 | 0.00728 | 0.61712 | 0.5077 | | 0.42105 | 0.207908 | 0.003661 | 0.54274 |
| *est120 | 0.6495 | 0.6912 | 0.281733 | 0.003343 | 0.58329 | 0.7082 | 0.70589 | 0.292838 | 0.007470 | 0.62791 | 0.6737 | 0.67802 | 0.272097 | 0.00192 | 0.60740 | 0.5875 | | 0.47059 | 0.243788 | 0.003343 | 0.53110 |
| AMB-00781591 | 0.4481 | 0.5204 | 0.163371 | 0.006599 | 0.57944 | 0.5120 | 0.60606 | 0.278171 | 0.007710 | 0.63520 | 0.3061 | 0.44817 | 0.111704 | 0.00883 | 0.59601 | 0.3956 | | 0.40404 | 0.198508 | 0.006599 | 0.54029 |
| *AMB-00677514 | 0.4927 | 0.5000 | 0.215762 | 0.003159 | 0.59110 | 0.4035 | 0.50000 | 0.215762 | 0.003230 | 0.63431 | 0.4149 | 0.50000 | 0.215762 | 0.00359 | 0.61479 | 0.4234 | | 0.33333 | 0.219512 | 0.003159 | 0.54758 |
| *est1342 | 0.7033 | 0.7435 | 0.310346 | 0.001928 | 0.58423 | 0.6020 | 0.64706 | 0.232308 | 0.002490 | 0.62512 | 0.7951 | 0.82972 | 0.406831 | 0.00173 | 0.61059 | 0.6621 | | 0.55315 | 0.297457 | 0.001928 | 0.53762 |
| AMB-00868578 | 0.4495 | 0.4722 | 0.200754 | 0.003240 | 0.59001 | 0.6120 | 0.52941 | 0.232214 | 0.001720 | 0.63577 | 0.4832 | 0.42105 | 0.174338 | 0.01456 | 0.61498 | 0.3900 | | 0.35294 | 0.167102 | 0.003240 | 0.54144 |
| ahb1672 | 0.5833 | 0.5882 | 0.267050 | 0.001522 | 0.59167 | 0.4963 | 0.58824 | 0.267050 | 0.001730 | 0.63597 | 0.5075 | 0.58824 | 0.267050 | 0.00272 | 0.61508 | 0.5165 | | 0.39216 | 0.269086 | 0.001522 | 0.54854 |
| ahb4228 | 0.4475 | 0.5033 | 0.166449 | 0.011107 | 0.58124 | 0.3800 | 0.50000 | 0.163192 | 0.012050 | 0.62141 | 0.3973 | 0.50619 | 0.169438 | 0.01675 | 0.60547 | 0.3783 | | 0.33746 | 0.159170 | 0.011107 | 0.53056 |
| ahb2973 | 0.7186 | 0.7420 | 0.338064 | 0.001679 | 0.58545 | 0.7643 | 0.73530 | 0.332658 | 0.000760 | 0.63025 | 0.7721 | 0.74837 | 0.343244 | 0.00298 | 0.61040 | 0.6642 | | 0.49891 | 0.289334 | 0.001679 | 0.53744 |
| *est9847 | 0.5189 | 0.5964 | 0.194780 | 0.006808 | 0.57919 | 0.5900 | 0.67647 | 0.270981 | 0.006850 | 0.62852 | 0.4038 | 0.52476 | 0.145391 | 0.00959 | 0.59871 | 0.4634 | | 0.45098 | 0.198817 | 0.006808 | 0.53212 |
| *ahb5992 | 0.7441 | 0.7680 | 0.345540 | 0.001576 | 0.58497 | 0.7051 | 0.76471 | 0.341352 | 0.001180 | 0.62966 | 0.7169 | 0.77090 | 0.349364 | 0.00085 | 0.61035 | 0.6917 | | 0.51393 | 0.321787 | 0.001576 | 0.53978 |
| AMB-00860795 | 0.4780 | 0.5000 | 0.215762 | 0.002509 | 0.59086 | 0.4910 | 0.41176 | 0.169703 | 0.002230 | 0.63479 | 0.6434 | 0.57895 | 0.261365 | 0.00503 | 0.61668 | 0.4304 | | 0.38597 | 0.181639 | 0.002509 | 0.54291 |
| AMB-00707983 | 0.4356 | 0.4722 | 0.167047 | 0.007972 | 0.58305 | 0.3155 | 0.44118 | 0.133964 | 0.008870 | 0.62055 | 0.4149 | 0.50000 | 0.215762 | 0.00416 | 0.61542 | 0.3694 | | 0.33333 | 0.175154 | 0.007972 | 0.54183 |
| AMB-00467628 | 0.4674 | 0.5016 | 0.182448 | 0.006697 | 0.58349 | 0.4339 | 0.52941 | 0.232214 | 0.005490 | 0.63480 | 0.3649 | 0.47678 | 0.154260 | 0.00496 | 0.60470 | 0.4001 | | 0.35294 | 0.192789 | 0.006697 | 0.54219 |
| AMB-00712474 | 0.5404 | 0.6062 | 0.212377 | 0.006127 | 0.57895 | 0.6541 | 0.67647 | 0.260280 | 0.004570 | 0.62732 | 0.5027 | 0.54334 | 0.175092 | 0.00836 | 0.60294 | 0.4824 | | 0.45098 | 0.192380 | 0.006127 | 0.52701 |
| *AMB-00350107 | 0.3515 | 0.4132 | 0.135575 | 0.012339 | 0.57971 | 0.0901 | 0.14522 | 0.030953 | 0.008290 | 0.61783 | 0.6685 | 0.65296 | 0.270743 | 0.00924 | 0.60831 | 0.4969 | | 0.43531 | 0.198996 | 0.012339 | 0.53503 |
| est8873 | 0.2790 | 0.3056 | 0.119748 | 0.007409 | 0.58872 | 0.4910 | 0.41176 | 0.169703 | 0.007430 | 0.63502 | 0.2406 | 0.21053 | 0.079169 | 0.01186 | 0.61290 | 0.2704 | | 0.27451 | 0.113150 | 0.007409 | 0.53838 |
| est1920 | 0.2790 | 0.3056 | 0.119748 | 0.008638 | 0.58831 | 0.5530 | 0.47059 | 0.199887 | 0.011340 | 0.63490 | 0.1740 | 0.15789 | 0.058110 | 0.00368 | 0.61317 | 0.3410 | | 0.31373 | 0.137426 | 0.008638 | 0.54070 |
| AMB-01108789 | 0.3640 | 0.3889 | 0.158479 | 0.002746 | 0.59038 | 0.6683 | 0.58824 | 0.267050 | 0.004740 | 0.63628 | 0.2406 | 0.21053 | 0.079169 | 0.00843 | 0.61271 | 0.4358 | | 0.39216 | 0.182115 | 0.002746 | 0.54178 |
| AMB-00869828 | 0.5602 | 0.6242 | 0.217928 | 0.005061 | 0.58057 | 0.5171 | 0.61764 | 0.212229 | 0.008330 | 0.62315 | 0.5377 | 0.63003 | 0.223173 | 0.00527 | 0.60534 | 0.4924 | | 0.42002 | 0.200875 | 0.005061 | 0.53052 |
| *est6087 | 0.5462 | 0.5915 | 0.216348 | 0.003737 | 0.58189 | 0.5608 | 0.64706 | 0.304877 | 0.002520 | 0.63600 | 0.4261 | 0.54180 | 0.169281 | 0.00623 | 0.60277 | 0.4848 | | 0.43137 | 0.234602 | 0.003737 | 0.54247 |
| est7167 | 0.5248 | 0.5948 | 0.198992 | 0.003623 | 0.58106 | 0.3298 | 0.47059 | 0.115720 | 0.003810 | 0.61387 | 0.6375 | 0.70588 | 0.346350 | 0.00374 | 0.61724 | 0.4887 | | 0.47059 | 0.238252 | 0.003623 | 0.54280 |
| AMB-00049429 | 0.2029 | 0.2286 | 0.086611 | 0.007253 | 0.58746 | -0.0500 | 0.00000 | 0.000000 | 0.000000 | 0.64355 | 0.5166 | 0.44444 | 0.186225 | 0.00954 | 0.61582 | 0.4606 | | 0.29629 | 0.190496 | 0.007253 | 0.54874 |
| AMB-00735568 | 0.6495 | 0.6912 | 0.281733 | 0.002440 | 0.58212 | 0.7646 | 0.76471 | 0.341352 | 0.001470 | 0.62978 | 0.6190 | 0.62539 | 0.236072 | 0.00484 | 0.60602 | 0.5973 | | 0.50981 | 0.254372 | 0.002440 | 0.53310 |
| AMB-00970711 | 0.3640 | 0.3889 | 0.158479 | 0.004188 | 0.58970 | 0.2854 | 0.23529 | 0.089413 | 0.004690 | 0.63397 | 0.5919 | 0.52632 | 0.230453 | 0.00417 | 0.61607 | 0.3815 | | 0.35088 | 0.154095 | 0.004188 | 0.54112 |
| est2936 | 0.5913 | 0.6569 | 0.234819 | 0.004478 | 0.58046 | 0.5726 | 0.64706 | 0.227148 | 0.005260 | 0.62427 | 0.5955 | 0.66564 | 0.241894 | 0.00410 | 0.60487 | 0.5252 | | 0.44376 | 0.210855 | 0.004478 | 0.52899 |
| ahb4188 | 0.7386 | 0.7712 | 0.337604 | 0.000625 | 0.58577 | 0.7269 | 0.76470 | 0.330937 | 0.000960 | 0.62766 | 0.7405 | 0.77709 | 0.343717 | 0.00121 | 0.60886 | 0.6856 | | 0.51806 | 0.304471 | 0.000625 | 0.53700 |
| AMB-00317545 | 0.4780 | 0.5000 | 0.215762 | 0.002533 | 0.59091 | 0.4910 | 0.41176 | 0.169703 | 0.000000 | 0.63556 | 0.6434 | 0.57895 | 0.261365 | 0.00475 | 0.61744 | 0.4304 | | 0.38597 | 0.181639 | 0.002533 | 0.54256 |
| ahb9682 | 0.2182 | 0.2353 | 0.089413 | 0.011118 | 0.58836 | 0.1508 | 0.23529 | 0.089413 | 0.013520 | 0.63263 | 0.1595 | 0.23529 | 0.089413 | 0.00665 | 0.61179 | 0.1629 | | 0.15686 | 0.093057 | 0.011118 | 0.54560 |
| *est5733 | 0.3300 | 0.4592 | 0.110130 | 0.012488 | 0.57344 | 0.0800 | 0.23530 | 0.030743 | 0.008040 | 0.59889 | 0.5765 | 0.65945 | 0.243624 | 0.01261 | 0.60690 | 0.3814 | | 0.43963 | 0.166394 | 0.012488 | 0.53247 |
| ahb958 | 0.3861 | 0.4419 | 0.150019 | 0.009837 | 0.57837 | 0.3199 | 0.32264 | 0.096045 | 0.008820 | 0.61988 | 0.5656 | 0.54865 | 0.206521 | 0.01209 | 0.60622 | 0.3596 | | 0.36577 | 0.138862 | 0.009837 | 0.52752 |
| ahb10249 | 0.4221 | 0.5327 | 0.182263 | 0.011043 | 0.57871 | 0.4872 | 0.54444 | 0.188808 | 0.008540 | 0.62339 | 0.4462 | 0.52339 | 0.177186 | 0.00474 | 0.60365 | 0.3148 | | 0.36296 | 0.154667 | 0.011043 | 0.52355 |
| est4644 | 0.4911 | 0.5523 | 0.191657 | 0.005522 | 0.58123 | 0.3172 | 0.35294 | 0.094988 | 0.005090 | 0.61916 | 0.7268 | 0.73065 | 0.312410 | 0.00475 | 0.60822 | 0.5121 | | 0.48710 | 0.208343 | 0.005522 | 0.53310 |
| AMB-00609940 | 0.3640 | 0.3889 | 0.158479 | 0.004531 | 0.58988 | 0.3575 | 0.29412 | 0.114674 | 0.009200 | 0.63355 | 0.5385 | 0.47368 | 0.201532 | 0.00170 | 0.61589 | 0.3301 | | 0.31579 | 0.136241 | 0.004531 | 0.54099 |
| ahb6325 | 0.6815 | 0.7190 | 0.303025 | 0.002127 | 0.58426 | 0.7646 | 0.76471 | 0.341352 | 0.002110 | 0.62852 | 0.6737 | 0.67802 | 0.272097 | 0.00151 | 0.60626 | 0.6258 | | 0.50981 | 0.267969 | 0.002127 | 0.53412 |
| est941 | 0.4891 | 0.5530 | 0.182172 | 0.006598 | 0.57961 | 0.3911 | 0.51871 | 0.153733 | 0.006740 | 0.61873 | 0.4874 | 0.58373 | 0.213137 | 0.00814 | 0.60545 | 0.4209 | | 0.38915 | 0.174181 | 0.006598 | 0.52977 |
| AMB-00812417 | 0.4927 | 0.5000 | 0.215762 | 0.001372 | 0.59093 | 0.4035 | 0.50000 | 0.215762 | 0.001570 | 0.63595 | 0.4149 | 0.50000 | 0.215762 | 0.00444 | 0.61547 | 0.4234 | | 0.33333 | 0.219512 | 0.001372 | 0.54798 |
| ahb4809 | 0.1014 | 0.1389 | 0.050729 | 0.059553 | 0.58640 | 0.0353 | 0.05882 | 0.020832 | 0.056740 | 0.63369 | 0.2229 | 0.21053 | 0.079169 | 0.09622 | 0.61381 | 0.1241 | | 0.14035 | 0.055912 | 0.059553 | 0.53742 |
| AMB-00523662 | 0.5858 | 0.6356 | 0.242784 | 0.002847 | 0.58048 | 0.7082 | 0.70589 | 0.292838 | 0.002030 | 0.62730 | 0.5626 | 0.57276 | 0.203511 | 0.00844 | 0.60429 | 0.5293 | | 0.47059 | 0.216892 | 0.002847 | 0.53031 |
| AMB-00935006 | 0.3910 | 0.4428 | 0.151084 | 0.006428 | 0.57877 | 0.5311 | 0.50000 | 0.180343 | 0.012400 | 0.62423 | 0.3966 | 0.39164 | 0.126790 | 0.01447 | 0.60324 | 0.3330 | | 0.33333 | 0.130184 | 0.006428 | 0.52470 |
| ahb5060 | 0.5680 | 0.6226 | 0.224448 | 0.002769 | 0.58065 | 0.5532 | 0.64706 | 0.250446 | 0.004120 | 0.62652 | 0.4997 | 0.60062 | 0.204060 | 0.01028 | 0.60499 | 0.5018 | | 0.43137 | 0.213472 | 0.002769 | 0.53337 |
| ahb10301 | 0.6495 | 0.6912 | 0.281733 | 0.001820 | 0.58307 | 0.8186 | 0.82353 | 0.398452 | 0.001770 | 0.63128 | 0.5626 | 0.57276 | 0.203511 | 0.00171 | 0.60186 | 0.6198 | | 0.54902 | 0.277291 | 0.001820 | 0.53502 |
| ahb6997 | 0.5640 | 0.5833 | 0.264041 | 0.000920 | 0.59091 | 0.6683 | 0.58824 | 0.267050 | 0.001150 | 0.63625 | 0.6434 | 0.57895 | 0.261365 | 0.00043 | 0.61668 | 0.4964 | | 0.39216 | 0.215618 | 0.000920 | 0.54290 |
| AMB-00366921 | 0.1357 | 0.1667 | 0.061555 | 0.004421 | 0.58904 | -0.0500 | 0.00000 | 0.000000 | 0.007630 | 0.64396 | 0.3614 | 0.31579 | 0.124335 | 0.00468 | 0.61473 | 0.3143 | | 0.21053 | 0.128611 | 0.004421 | 0.54881 |
| ahb308 | 0.3315 | 0.3873 | 0.124785 | 0.010289 | 0.57662 | 0.5311 | 0.50000 | 0.180343 | 0.013340 | 0.62178 | 0.2706 | 0.28638 | 0.081813 | 0.01091 | 0.60068 | 0.3095 | | 0.33333 | 0.120935 | 0.010289 | 0.52374 |
| AMB-00997772 | 0.6065 | 0.6618 | 0.249620 | 0.002341 | 0.58089 | 0.6541 | 0.67647 | 0.260280 | 0.003140 | 0.62727 | 0.6198 | 0.64860 | 0.240384 | 0.00547 | 0.60334 | 0.5414 | | 0.45098 | 0.217900 | 0.002341 | 0.52980 |
| AMB-00402575 | 0.5203 | 0.5480 | 0.208077 | 0.003948 | 0.58352 | 0.4004 | 0.51694 | 0.172139 | 0.001160 | 0.62322 | 0.4966 | 0.57576 | 0.259429 | 0.00408 | 0.61665 | 0.4521 | | 0.38384 | 0.214192 | 0.003948 | 0.54281 |
| ahb5218 | 0.3612 | 0.4150 | 0.137687 | 0.012288 | 0.57979 | 0.0990 | 0.14706 | 0.032152 | 0.003610 | 0.62048 | 0.6788 | 0.65480 | 0.273388 | 0.00448 | 0.60928 | 0.5060 | | 0.43653 | 0.200592 | 0.012288 | 0.53524 |
| ahb11898 | 0.0542 | 0.0833 | 0.029787 | 0.011577 | 0.58733 | -0.0500 | 0.00000 | 0.000000 | 0.014660 | 0.64433 | 0.1740 | 0.15789 | 0.058110 | 0.00898 | 0.61241 | 0.1393 | | 0.10526 | 0.060805 | 0.011577 | 0.54576 |
| *AMB-00645946 | 0.1381 | 0.1667 | 0.061555 | 0.008452 | 0.58856 | -0.0500 | 0.00000 | 0.000000 | 0.009310 | 0.64256 | 0.3665 | 0.31579 | 0.124335 | 0.00105 | 0.61470 | 0.3172 | | 0.21053 | 0.128611 | 0.008452 | 0.54789 |
| AMB-00913659 | 0.6109 | 0.6787 | 0.280599 | 0.002313 | 0.58343 | 0.6438 | 0.66240 | 0.269079 | 0.003020 | 0.62684 | 0.6693 | 0.69336 | 0.291230 | 0.00239 | 0.60810 | 0.5206 | | 0.46224 | 0.239446 | 0.002313 | 0.53308 |
| *est7489 | 0.5411 | 0.5817 | 0.226596 | 0.003561 | 0.58345 | 0.7099 | 0.67647 | 0.288447 | 0.002300 | 0.62920 | 0.5151 | 0.49691 | 0.178698 | 0.00540 | 0.60530 | 0.4932 | | 0.45098 | 0.203714 | 0.003561 | 0.53222 |
| *est775 | 0.3317 | 0.4379 | 0.114169 | 0.011578 | 0.57240 | 0.1890 | 0.29411 | 0.058069 | 0.008500 | 0.61055 | 0.5077 | 0.56656 | 0.180454 | 0.01269 | 0.60106 | 0.3212 | | 0.37771 | 0.120357 | 0.011578 | 0.52118 |
| *ahb11720 | 0.4625 | 0.4706 | 0.199887 | 0.000827 | 0.59056 | 0.3736 | 0.47059 | 0.199887 | 0.001970 | 0.63459 | 0.3849 | 0.47059 | 0.199887 | 0.00453 | 0.61647 | 0.3931 | | 0.31373 | 0.203959 | 0.000827 | 0.54778 |
| AMB-00024857 | 0.5714 | 0.6095 | 0.243706 | 0.001525 | 0.58333 | 0.8662 | 0.85294 | 0.441798 | 0.001690 | 0.63307 | 0.3966 | 0.39164 | 0.126790 | 0.00203 | 0.60140 | 0.6274 | | 0.56863 | 0.295079 | 0.001525 | 0.53970 |
| ahb8402 | 0.3073 | 0.3333 | 0.132304 | 0.004080 | 0.58927 | 0.2093 | 0.17647 | 0.065436 | 0.003780 | 0.63347 | 0.5385 | 0.47368 | 0.201532 | 0.00404 | 0.61528 | 0.3464 | | 0.31579 | 0.136803 | 0.004080 | 0.54204 |
| ahb222 | -0.0500 | 0.0000 | 0.000000 | 0.006388 | 0.60017 | -0.0500 | 0.00000 | 0.000000 | 0.007510 | 0.64283 | -0.0500 | 0.00000 | 0.000000 | 0.01226 | 0.62323 | -0.0500 | | 0.00000 | 0.000000 | 0.006388 | 0.55543 |
| AMB-00103263 | -0.0500 | 0.0000 | 0.000000 | 0.010017 | 0.59936 | -0.0500 | 0.00000 | 0.000000 | 0.008570 | 0.64234 | -0.0500 | 0.00000 | 0.000000 | 0.00506 | 0.62270 | -0.0500 | | 0.00000 | 0.000000 | 0.010017 | 0.55491 |
| est1363 | -0.0500 | 0.0000 | 0.000000 | 0.015939 | 0.59874 | -0.0500 | 0.00000 | 0.000000 | 0.006770 | 0.64175 | -0.0500 | 0.00000 | 0.000000 | 0.00561 | 0.62163 | -0.0500 | | 0.00000 | 0.000000 | 0.015939 | 0.55577 |
| ahb1707 | 0.4827 | 0.5670 | 0.176681 | 0.003180 | 0.57846 | 0.6276 | 0.70588 | 0.346350 | 0.001790 | 0.63654 | 0.2979 | 0.44272 | 0.101602 | 0.00365 | 0.59517 | 0.4552 | | 0.47059 | 0.235076 | 0.003180 | 0.54317 |
| *AMB-01027388 | 0.2298 | 0.3056 | 0.119748 | 0.005969 | 0.58787 | 0.0808 | 0.11765 | 0.042613 | 0.003910 | 0.63340 | 0.4594 | 0.47368 | 0.201532 | 0.00410 | 0.61491 | 0.3190 | | 0.31579 | 0.145046 | 0.005969 | 0.54179 |
| ahb6404 | -0.0500 | 0.0000 | 0.000000 | 0.009204 | 0.59751 | -0.0500 | 0.00000 | 0.000000 | 0.007430 | 0.64215 | -0.0500 | 0.00000 | 0.000000 | 0.00870 | 0.62236 | -0.0500 | | 0.00000 | 0.000000 | 0.009204 | 0.55466 |
| AMB-00419626 | 0.2908 | 0.4248 | 0.095102 | 0.014341 | 0.57133 | 0.3270 | 0.47059 | 0.119871 | 0.019140 | 0.61615 | 0.2231 | 0.38390 | 0.076320 | 0.01644 | 0.59045 | 0.2342 | | 0.31373 | 0.090829 | 0.014341 | 0.49694 |
| est3802 | 0.4208 | 0.4706 | 0.165002 | 0.004518 | 0.58006 | 0.7643 | 0.73530 | 0.332658 | 0.005230 | 0.62977 | 0.2053 | 0.23375 | 0.061698 | 0.00267 | 0.59943 | 0.5369 | | 0.49020 | 0.230728 | 0.004518 | 0.53624 |
| est8460 | 0.4201 | 0.5065 | 0.148878 | 0.005252 | 0.57767 | 0.2908 | 0.44118 | 0.106199 | 0.008140 | 0.61629 | 0.4640 | 0.56502 | 0.202060 | 0.00877 | 0.60577 | 0.3619 | | 0.37668 | 0.149267 | 0.005252 | 0.52929 |
| est4702 | 0.4780 | 0.5000 | 0.215762 | 0.001395 | 0.59026 | 0.6120 | 0.52941 | 0.232214 | 0.002320 | 0.63627 | 0.5385 | 0.47368 | 0.201532 | 0.00209 | 0.61494 | 0.4111 | | 0.35294 | 0.176168 | 0.001395 | 0.54085 |
| AMB-01008399 | 0.3640 | 0.3889 | 0.158479 | 0.001936 | 0.58879 | 0.7221 | 0.64706 | 0.304877 | 0.002710 | 0.63673 | 0.1740 | 0.15789 | 0.058110 | 0.00328 | 0.61315 | 0.5211 | | 0.43137 | 0.219775 | 0.001936 | 0.54302 |
| AMB-01026012 | 0.2052 | 0.2571 | 0.098638 | 0.009381 | 0.58864 | 0.1135 | 0.12500 | 0.045407 | 0.004030 | 0.63459 | 0.3806 | 0.36842 | 0.148663 | 0.00372 | 0.61285 | 0.2332 | | 0.24561 | 0.102054 | 0.009381 | 0.53953 |
| AMB-01022084 | 0.0948 | 0.1177 | 0.042613 | 0.011977 | 0.58892 | 0.0494 | 0.11765 | 0.042613 | 0.008670 | 0.63330 | 0.0557 | 0.11765 | 0.042613 | 0.00596 | 0.61251 | 0.0554 | | 0.07843 | 0.044707 | 0.011977 | 0.54618 |
| *est9781 | 0.3315 | 0.3873 | 0.124785 | 0.010526 | 0.57862 | 0.3975 | 0.38235 | 0.122559 | 0.007620 | 0.62368 | 0.3966 | 0.39164 | 0.126790 | 0.00698 | 0.60238 | 0.2668 | | 0.26109 | 0.102878 | 0.010526 | 0.52094 |
| *AMB-00040122 | 0.3999 | 0.4286 | 0.178126 | 0.002088 | 0.58963 | 0.6430 | 0.56250 | 0.251473 | 0.000000 | 0.63577 | 0.3562 | 0.31579 | 0.124335 | 0.00591 | 0.61387 | 0.3853 | | 0.37500 | 0.168284 | 0.002088 | 0.54126 |
| ahb8153 | -0.0500 | 0.0000 | 0.000000 | 0.011554 | 0.59853 | -0.0500 | 0.00000 | 0.000000 | 0.002160 | 0.64305 | -0.0500 | 0.00000 | 0.000000 | 0.00881 | 0.62245 | -0.0500 | | 0.00000 | 0.000000 | 0.011554 | 0.55624 |
| *est7725 | 0.0017 | 0.0294 | 0.010303 | 0.016836 | 0.58982 | -0.0228 | 0.02941 | 0.010303 | 0.021010 | 0.63411 | -0.0186 | 0.02941 | 0.010303 | 0.03469 | 0.61339 | -0.0221 | | 0.01961 | 0.010868 | 0.016836 | 0.54543 |
| est8172 | 0.4961 | 0.5143 | 0.223678 | 0.000591 | 0.59106 | 0.4259 | 0.35294 | 0.141375 | 0.000440 | 0.63419 | 0.7323 | 0.66667 | 0.318257 | 0.00073 | 0.61750 | 0.5002 | | 0.44445 | 0.212361 | 0.000591 | 0.54285 |
| AMB-00207080 | 0.2145 | 0.3513 | 0.069951 | 0.014485 | 0.56847 | -0.0347 | 0.05882 | 0.002653 | 0.005090 | 0.60123 | 0.5332 | 0.61300 | 0.202241 | 0.01141 | 0.60432 | 0.3836 | | 0.40867 | 0.164430 | 0.014485 | 0.53257 |
| *AMB-00528349 | 0.3102 | 0.3235 | 0.127834 | 0.002034 | 0.59001 | 0.2311 | 0.32353 | 0.127834 | 0.005950 | 0.63346 | 0.2411 | 0.32353 | 0.127834 | 0.00598 | 0.61354 | 0.2468 | | 0.21569 | 0.132148 | 0.002034 | 0.54625 |
| ahb5409 | 0.4209 | 0.4444 | 0.186225 | 0.000893 | 0.59080 | 0.7735 | 0.70588 | 0.346350 | 0.000420 | 0.63687 | 0.2406 | 0.21053 | 0.079169 | 0.00124 | 0.61399 | 0.5529 | | 0.47059 | 0.241944 | 0.000893 | 0.54342 |
| *est6624 | -0.0500 | 0.0000 | 0.000000 | 0.008367 | 0.59851 | -0.0500 | 0.00000 | 0.000000 | 0.007400 | 0.64286 | -0.0500 | 0.00000 | 0.000000 | 0.00544 | 0.62090 | -0.0500 | | 0.00000 | 0.000000 | 0.008367 | 0.55423 |
| *ahb10579 | 0.2489 | 0.2647 | 0.101874 | 0.004988 | 0.58959 | 0.1772 | 0.26471 | 0.101874 | 0.005400 | 0.63366 | 0.1863 | 0.26471 | 0.101874 | 0.00263 | 0.61357 | 0.1905 | | 0.17647 | 0.105795 | 0.004988 | 0.54644 |
| AMB-00157650 | -0.0019 | 0.0303 | 0.010619 | 0.016850 | 0.58929 | -0.0500 | 0.00000 | 0.000000 | 0.006780 | 0.64077 | 0.0270 | 0.05263 | 0.018596 | 0.02852 | 0.61460 | 0.0094 | | 0.03509 | 0.019589 | 0.016850 | 0.54515 |
| *AMB-00609985 | 0.2137 | 0.2663 | 0.074874 | 0.007529 | 0.58051 | 0.1115 | 0.23530 | 0.051242 | 0.007900 | 0.61747 | 0.2135 | 0.29412 | 0.114674 | 0.01366 | 0.61383 | 0.1636 | | 0.19608 | 0.085705 | 0.007529 | 0.53899 |
| ahb9035 | 0.3168 | 0.3856 | 0.110148 | 0.008873 | 0.57971 | 0.2538 | 0.38236 | 0.107371 | 0.010710 | 0.62182 | 0.2709 | 0.38855 | 0.112707 | 0.00657 | 0.60437 | 0.2529 | | 0.25903 | 0.105678 | 0.008873 | 0.52729 |
| AMB-00257400 | 0.4549 | 0.4874 | 0.174901 | 0.002415 | 0.58191 | 0.4214 | 0.51515 | 0.224162 | 0.003350 | 0.63474 | 0.3509 | 0.46252 | 0.147151 | 0.00683 | 0.60438 | 0.3861 | | 0.34343 | 0.185523 | 0.002415 | 0.54132 |
| *est4044 | 0.4484 | 0.5126 | 0.169828 | 0.005891 | 0.57839 | 0.4280 | 0.44118 | 0.133964 | 0.003330 | 0.62180 | 0.5626 | 0.57276 | 0.203511 | 0.00452 | 0.60280 | 0.3926 | | 0.38184 | 0.146701 | 0.005891 | 0.52503 |
| est10068 | 0.2008 | 0.3333 | 0.064660 | 0.019095 | 0.57196 | 0.0945 | 0.26471 | 0.038321 | 0.017640 | 0.60222 | 0.2566 | 0.39474 | 0.098256 | 0.01952 | 0.59615 | 0.1615 | | 0.26316 | 0.068050 | 0.019095 | 0.50197 |
| ahb8858 | 0.0263 | 0.0556 | 0.019651 | 0.013697 | 0.58841 | -0.0500 | 0.00000 | 0.000000 | 0.004210 | 0.64151 | 0.1046 | 0.10526 | 0.037944 | 0.00777 | 0.61466 | 0.0781 | | 0.07017 | 0.039840 | 0.013697 | 0.54527 |
| AMB-00834688 | -0.0500 | 0.0000 | 0.000000 | 0.006701 | 0.59881 | -0.0500 | 0.00000 | 0.000000 | 0.002540 | 0.64243 | -0.0500 | 0.00000 | 0.000000 | 0.01514 | 0.62269 | -0.0500 | | 0.00000 | 0.000000 | 0.006701 | 0.55378 |
| ahb10355 | 0.4508 | 0.4984 | 0.179474 | 0.002398 | 0.58065 | 0.8164 | 0.79412 | 0.382949 | 0.001760 | 0.63141 | 0.2053 | 0.23375 | 0.061698 | 0.00321 | 0.59970 | 0.5965 | | 0.52941 | 0.268354 | 0.002398 | 0.53829 |
| *est1222 | 0.2452 | 0.2958 | 0.086523 | 0.007366 | 0.58061 | 0.1384 | 0.26471 | 0.061315 | 0.007310 | 0.61727 | 0.2411 | 0.32353 | 0.127834 | 0.00879 | 0.61273 | 0.1916 | | 0.21569 | 0.097106 | 0.007366 | 0.53896 |
| AMB-00492753 | 0.3160 | 0.3706 | 0.117274 | 0.003330 | 0.57793 | 0.7557 | 0.72059 | 0.321103 | 0.004000 | 0.62987 | 0.0120 | 0.07585 | 0.012103 | 0.00501 | 0.60036 | 0.6057 | | 0.48039 | 0.262633 | 0.003330 | 0.54051 |
| *AMB-00046394 | 0.4428 | 0.5229 | 0.163902 | 0.004272 | 0.57566 | 0.2482 | 0.32352 | 0.074370 | 0.005150 | 0.61324 | 0.6760 | 0.70123 | 0.279106 | 0.00325 | 0.60711 | 0.4659 | | 0.46749 | 0.186404 | 0.004272 | 0.53122 |
| *AMB-00457799 | -0.0500 | 0.0000 | 0.000000 | 0.007849 | 0.59718 | -0.0500 | 0.00000 | 0.000000 | 0.010830 | 0.64433 | -0.0500 | 0.00000 | 0.000000 | 0.00245 | 0.62203 | -0.0500 | | 0.00000 | 0.000000 | 0.007849 | 0.55538 |
| AMB-00331869 | 0.2986 | 0.3638 | 0.100808 | 0.009617 | 0.57963 | 0.2347 | 0.36053 | 0.098127 | 0.006150 | 0.62156 | 0.2523 | 0.36672 | 0.103280 | 0.01051 | 0.60151 | 0.2317 | | 0.24448 | 0.096719 | 0.009617 | 0.52351 |
| AMB-00968616 | -0.0500 | 0.0000 | 0.000000 | 0.004339 | 0.59909 | -0.0500 | 0.00000 | 0.000000 | 0.006990 | 0.64284 | -0.0500 | 0.00000 | 0.000000 | 0.00789 | 0.62285 | -0.0500 | | 0.00000 | 0.000000 | 0.004339 | 0.55536 |
| *ahb10780 | 0.5625 | 0.5899 | 0.232830 | 0.001388 | 0.58342 | 0.5283 | 0.61765 | 0.285554 | 0.002350 | 0.63534 | 0.4640 | 0.56502 | 0.202060 | 0.00136 | 0.60603 | 0.4966 | | 0.41177 | 0.240745 | 0.001388 | 0.54394 |
| *AMB-00354458 | 0.1381 | 0.1667 | 0.061555 | 0.003973 | 0.58787 | -0.0500 | 0.00000 | 0.000000 | 0.000000 | 0.64254 | 0.3665 | 0.31579 | 0.124335 | 0.01250 | 0.61443 | 0.3172 | | 0.21053 | 0.128611 | 0.003973 | 0.54804 |
| ahb9265 | 0.2489 | 0.2647 | 0.101874 | 0.003575 | 0.58899 | 0.1772 | 0.26471 | 0.101874 | 0.004370 | 0.63262 | 0.1863 | 0.26471 | 0.101874 | 0.00579 | 0.61368 | 0.1905 | | 0.17647 | 0.105795 | 0.003575 | 0.54458 |
| AMB-00111965 | 0.0729 | 0.2288 | 0.026399 | 0.031395 | 0.55765 | -0.0442 | 0.02941 | 0.000465 | 0.075900 | 0.58490 | 0.3195 | 0.45976 | 0.117057 | 0.05261 | 0.59616 | 0.2164 | | 0.32611 | 0.108277 | 0.031395 | 0.52823 |
| ahb5536 | 0.3102 | 0.3235 | 0.127834 | 0.002888 | 0.58993 | 0.2311 | 0.32353 | 0.127834 | 0.002560 | 0.63443 | 0.2411 | 0.32353 | 0.127834 | 0.00241 | 0.61373 | 0.2468 | | 0.21569 | 0.132148 | 0.002888 | 0.54695 |
| est8305 | -0.0016 | 0.0278 | 0.009725 | 0.005213 | 0.58944 | 0.0439 | 0.05882 | 0.020832 | 0.011540 | 0.63428 | -0.0500 | 0.00000 | 0.000000 | 0.01093 | 0.62376 | 0.0258 | | 0.03921 | 0.021936 | 0.005213 | 0.54527 |
| AMB-00230746 | 0.0164 | 0.1219 | 0.011581 | 0.025297 | 0.56454 | -0.0422 | 0.04779 | 0.001392 | 0.029030 | 0.59048 | 0.1863 | 0.26471 | 0.101874 | 0.01245 | 0.61251 | 0.1081 | | 0.20833 | 0.090018 | 0.025297 | 0.54016 |
| *ahb4874 | 0.1101 | 0.1389 | 0.050729 | 0.006517 | 0.58784 | 0.3575 | 0.29412 | 0.114674 | 0.006110 | 0.63434 | -0.0500 | 0.00000 | 0.000000 | 0.00000 | 0.62240 | 0.3021 | | 0.19608 | 0.118821 | 0.006517 | 0.54696 |
| ahb8744 | 0.2790 | 0.3056 | 0.119748 | 0.002950 | 0.58873 | 0.2093 | 0.17647 | 0.065436 | 0.002540 | 0.63398 | 0.4832 | 0.42105 | 0.174338 | 0.00599 | 0.61411 | 0.2947 | | 0.28070 | 0.117046 | 0.002950 | 0.53885 |
| est3202 | 0.6815 | 0.7190 | 0.303025 | 0.000000 | 0.58345 | 0.6493 | 0.64706 | 0.250446 | 0.000000 | 0.62538 | 0.7782 | 0.78329 | 0.358291 | 0.00000 | 0.61036 | 0.6313 | | 0.52219 | 0.268717 | 0.000000 | 0.53628 |
| AMB-00728581 | 0.3612 | 0.4150 | 0.137687 | 0.001964 | 0.57845 | -0.0342 | 0.02941 | 0.002612 | 0.003820 | 0.62309 | 0.7791 | 0.76006 | 0.352983 | 0.00219 | 0.61035 | 0.6958 | | 0.50671 | 0.314082 | 0.001964 | 0.54416 |
| AMB-00384184 | 0.0542 | 0.0833 | 0.029787 | 0.007861 | 0.58828 | -0.0500 | 0.00000 | 0.000000 | 0.003250 | 0.64167 | 0.1740 | 0.15789 | 0.058110 | 0.00587 | 0.61350 | 0.1393 | | 0.10526 | 0.060805 | 0.007861 | 0.54662 |
| AMB-01006752 | 0.1283 | 0.1928 | 0.047158 | 0.018383 | 0.57489 | 0.3975 | 0.38235 | 0.122559 | 0.035320 | 0.62163 | -0.0350 | 0.02322 | 0.001736 | 0.00834 | 0.60032 | 0.2901 | | 0.25490 | 0.108431 | 0.018383 | 0.53198 |
| *ahb2985 | 0.2489 | 0.2647 | 0.101874 | 0.003341 | 0.58947 | 0.1772 | 0.26471 | 0.101874 | 0.002370 | 0.63416 | 0.1863 | 0.26471 | 0.101874 | 0.00440 | 0.61404 | 0.1905 | | 0.17647 | 0.105795 | 0.003341 | 0.54622 |
| est1153 | 0.2507 | 0.2778 | 0.107520 | 0.005737 | 0.58818 | 0.4910 | 0.41176 | 0.169703 | 0.005180 | 0.63515 | 0.1740 | 0.15789 | 0.058110 | 0.00060 | 0.61254 | 0.2829 | | 0.27451 | 0.114839 | 0.005737 | 0.53988 |
| AMB-00223236 | 0.0638 | 0.0882 | 0.031599 | 0.006019 | 0.58985 | 0.0250 | 0.08824 | 0.031599 | 0.007300 | 0.63265 | 0.0306 | 0.08824 | 0.031599 | 0.01012 | 0.61343 | 0.0293 | | 0.05883 | 0.033213 | 0.006019 | 0.54510 |
| AMB-00575908 | -0.0500 | 0.0000 | 0.000000 | 0.010897 | 0.59807 | -0.0500 | 0.00000 | 0.000000 | 0.005760 | 0.64332 | -0.0500 | 0.00000 | 0.000000 | 0.00022 | 0.62354 | -0.0500 | | 0.00000 | 0.000000 | 0.010897 | 0.55443 |
| *est1170 | 0.6312 | 0.6830 | 0.260009 | 0.001000 | 0.58172 | 0.5979 | 0.67647 | 0.253919 | 0.002280 | 0.62367 | 0.6162 | 0.68886 | 0.265604 | 0.00148 | 0.60649 | 0.5674 | | 0.45924 | 0.237991 | 0.001000 | 0.53164 |
| AMB-01006260 | -0.0500 | 0.0000 | 0.000000 | 0.004709 | 0.59680 | -0.0500 | 0.00000 | 0.000000 | 0.005410 | 0.64215 | -0.0500 | 0.00000 | 0.000000 | 0.00777 | 0.62322 | -0.0500 | | 0.00000 | 0.000000 | 0.004709 | 0.55395 |
| AMB-00190928 | 0.4475 | 0.5033 | 0.166449 | 0.001807 | 0.58043 | 0.4648 | 0.55882 | 0.249292 | 0.002180 | 0.63659 | 0.3219 | 0.45356 | 0.124263 | 0.00664 | 0.59962 | 0.3863 | | 0.37255 | 0.187725 | 0.001807 | 0.54067 |
| ahb7601 | 0.0542 | 0.0833 | 0.029787 | 0.004196 | 0.58867 | -0.0500 | 0.00000 | 0.000000 | 0.007990 | 0.64258 | 0.1740 | 0.15789 | 0.058110 | 0.00525 | 0.61367 | 0.1393 | | 0.10526 | 0.060805 | 0.004196 | 0.54627 |
| AMB-00199828 | 0.0948 | 0.1177 | 0.042613 | 0.014505 | 0.58864 | 0.0494 | 0.11765 | 0.042613 | 0.006480 | 0.63198 | 0.0557 | 0.11765 | 0.042613 | 0.00351 | 0.61147 | 0.0554 | | 0.07843 | 0.044707 | 0.014505 | 0.54396 |
| *AMB-00776332 | 0.3357 | 0.3832 | 0.123503 | 0.004159 | 0.58157 | 0.3152 | 0.41176 | 0.169703 | 0.004540 | 0.63459 | 0.2326 | 0.35620 | 0.097635 | 0.00495 | 0.60150 | 0.2744 | | 0.27451 | 0.135243 | 0.004159 | 0.53961 |
| est6729 | 0.3737 | 0.4097 | 0.136323 | 0.002511 | 0.58309 | 0.3448 | 0.43750 | 0.182664 | 0.004330 | 0.63412 | 0.2698 | 0.38487 | 0.111096 | 0.00698 | 0.60333 | 0.3061 | | 0.29167 | 0.148059 | 0.002511 | 0.54014 |
| AMB-00646076 | 0.0638 | 0.0882 | 0.031599 | 0.006485 | 0.58866 | 0.0250 | 0.08824 | 0.031599 | 0.006180 | 0.63228 | 0.0306 | 0.08824 | 0.031599 | 0.00976 | 0.61343 | 0.0293 | | 0.05883 | 0.033213 | 0.006485 | 0.54576 |
| AMB-00923535 | 0.1566 | 0.1765 | 0.065436 | 0.003393 | 0.58916 | 0.0993 | 0.17647 | 0.065436 | 0.004210 | 0.63406 | 0.1069 | 0.17647 | 0.065436 | 0.00597 | 0.61358 | 0.1086 | | 0.11765 | 0.068385 | 0.003393 | 0.54665 |
| AMB-00046221 | 0.2328 | 0.3627 | 0.075236 | 0.014576 | 0.57266 | 0.2656 | 0.41176 | 0.102688 | 0.011450 | 0.61699 | 0.1540 | 0.31888 | 0.055905 | 0.00452 | 0.59143 | 0.1830 | | 0.27451 | 0.075645 | 0.014576 | 0.49837 |
| AMB-01023218 | 0.1838 | 0.2188 | 0.082546 | 0.000996 | 0.58746 | 0.5401 | 0.46667 | 0.197811 | 0.002220 | 0.63514 | -0.0500 | 0.00000 | 0.000000 | 0.00367 | 0.62313 | 0.4832 | | 0.31111 | 0.201918 | 0.000996 | 0.54989 |
| est102 | 0.3168 | 0.3856 | 0.110148 | 0.005513 | 0.57982 | 0.2538 | 0.38236 | 0.107371 | 0.007080 | 0.62112 | 0.2709 | 0.38855 | 0.112707 | 0.00715 | 0.60342 | 0.2529 | | 0.25903 | 0.105678 | 0.005513 | 0.52389 |
| AMB-00098317 | 0.3215 | 0.3983 | 0.114343 | 0.006687 | 0.57589 | 0.1712 | 0.23530 | 0.051242 | 0.008590 | 0.61411 | 0.5445 | 0.55229 | 0.191657 | 0.00227 | 0.60344 | 0.3428 | | 0.36819 | 0.128629 | 0.006687 | 0.52501 |
| *ahb10961 | 0.4018 | 0.5114 | 0.138971 | 0.005055 | 0.57426 | 0.4035 | 0.52941 | 0.150312 | 0.004140 | 0.62129 | 0.3636 | 0.49535 | 0.129424 | 0.00509 | 0.59766 | 0.3344 | | 0.35294 | 0.127032 | 0.005055 | 0.51411 |
| AMB-00087856 | 0.2844 | 0.3562 | 0.097635 | 0.003807 | 0.57742 | 0.1437 | 0.29411 | 0.058069 | 0.006220 | 0.61100 | 0.3261 | 0.41176 | 0.169703 | 0.00556 | 0.61442 | 0.2366 | | 0.27451 | 0.119431 | 0.003807 | 0.54018 |
| ahb7354 | -0.0016 | 0.0278 | 0.009725 | 0.006499 | 0.58999 | -0.0500 | 0.00000 | 0.000000 | 0.007060 | 0.64240 | 0.0325 | 0.05263 | 0.018596 | 0.00977 | 0.61334 | 0.0159 | | 0.03509 | 0.019589 | 0.006499 | 0.54516 |
| *AMB-00905664 | 0.3280 | 0.4369 | 0.109580 | 0.005224 | 0.57486 | 0.4004 | 0.51694 | 0.172139 | 0.004040 | 0.62392 | 0.2037 | 0.36523 | 0.071994 | 0.01091 | 0.59096 | 0.2791 | | 0.34463 | 0.121222 | 0.005224 | 0.52245 |
| est7473 | 0.2523 | 0.3268 | 0.085700 | 0.005391 | 0.57848 | 0.2867 | 0.38235 | 0.155322 | 0.005170 | 0.63348 | 0.1367 | 0.27709 | 0.054580 | 0.00841 | 0.59532 | 0.2053 | | 0.25490 | 0.109956 | 0.005391 | 0.53734 |
| AMB-00616537 | 0.1101 | 0.1389 | 0.050729 | 0.004824 | 0.58750 | 0.1289 | 0.11765 | 0.042613 | 0.006940 | 0.63429 | 0.1740 | 0.15789 | 0.058110 | 0.00977 | 0.61351 | 0.0733 | | 0.10526 | 0.040667 | 0.004824 | 0.53717 |
| AMB-00614253 | 0.2452 | 0.2958 | 0.086523 | 0.007135 | 0.57959 | 0.2311 | 0.32353 | 0.127834 | 0.007510 | 0.63332 | 0.1540 | 0.27090 | 0.065581 | 0.00328 | 0.60224 | 0.1907 | | 0.21569 | 0.098958 | 0.007135 | 0.53786 |
| *est4588 | 0.0017 | 0.0294 | 0.010303 | 0.014266 | 0.58959 | -0.0228 | 0.02941 | 0.010303 | 0.010130 | 0.63391 | -0.0186 | 0.02941 | 0.010303 | 0.00312 | 0.61420 | -0.0221 | | 0.01961 | 0.010868 | 0.014266 | 0.54594 |
| *AMB-00300910 | 0.0511 | 0.0833 | 0.029787 | 0.004073 | 0.58764 | -0.0500 | 0.00000 | 0.000000 | 0.008100 | 0.64141 | 0.1666 | 0.15789 | 0.058110 | 0.00759 | 0.61253 | 0.1352 | | 0.10526 | 0.060805 | 0.004073 | 0.54602 |
| *est5551 | 0.6312 | 0.6830 | 0.260009 | 0.000477 | 0.58043 | 0.6660 | 0.73530 | 0.316211 | 0.000310 | 0.63025 | 0.5527 | 0.63623 | 0.219689 | 0.00083 | 0.60424 | 0.5725 | | 0.49020 | 0.249545 | 0.000477 | 0.53427 |
| ahb4861 | 0.1853 | 0.2484 | 0.067127 | 0.009073 | 0.57765 | 0.1760 | 0.20588 | 0.051692 | 0.007570 | 0.62091 | 0.2706 | 0.28638 | 0.081813 | 0.01002 | 0.60201 | 0.1428 | | 0.19092 | 0.057276 | 0.009073 | 0.50659 |
| AMB-00315714 | -0.0016 | 0.0278 | 0.009725 | 0.006610 | 0.58933 | -0.0500 | 0.00000 | 0.000000 | 0.003900 | 0.64243 | 0.0325 | 0.05263 | 0.018596 | 0.00897 | 0.61415 | 0.0159 | | 0.03509 | 0.019589 | 0.006610 | 0.54690 |
| AMB-00205067 | 0.2100 | 0.2753 | 0.076942 | 0.010621 | 0.57484 | 0.5875 | 0.55794 | 0.211870 | 0.005190 | 0.62739 | -0.0362 | 0.02233 | 0.001587 | 0.00179 | 0.60239 | 0.4799 | | 0.37196 | 0.191753 | 0.010621 | 0.53712 |
| ahb2293 | 0.4674 | 0.5016 | 0.182448 | 0.001251 | 0.58349 | 0.3474 | 0.47059 | 0.148230 | 0.000170 | 0.62066 | 0.4453 | 0.52941 | 0.232214 | 0.00483 | 0.61609 | 0.4006 | | 0.35294 | 0.189873 | 0.001251 | 0.54294 |
| *AMB-00546280 | -0.0500 | 0.0000 | 0.000000 | 0.006395 | 0.59763 | -0.0500 | 0.00000 | 0.000000 | 0.009010 | 0.64177 | -0.0500 | 0.00000 | 0.000000 | 0.00139 | 0.62238 | -0.0500 | | 0.00000 | 0.000000 | 0.006395 | 0.55389 |
| AMB-00869070 | 0.3102 | 0.3235 | 0.127834 | 0.002929 | 0.58999 | 0.2311 | 0.32353 | 0.127834 | 0.001490 | 0.63288 | 0.2411 | 0.32353 | 0.127834 | 0.00193 | 0.61309 | 0.2468 | | 0.21569 | 0.132148 | 0.002929 | 0.54694 |
| AMB-00012593 | 0.1944 | 0.2222 | 0.083979 | 0.003314 | 0.58782 | 0.0439 | 0.05882 | 0.020832 | 0.003830 | 0.63254 | 0.4259 | 0.36842 | 0.148663 | 0.00379 | 0.61538 | 0.3065 | | 0.24561 | 0.115380 | 0.003314 | 0.54099 |
| AMB-00087121 | 0.0821 | 0.1111 | 0.040143 | 0.006039 | 0.58734 | 0.2854 | 0.23529 | 0.089413 | 0.005860 | 0.63323 | -0.0500 | 0.00000 | 0.000000 | 0.00000 | 0.62205 | 0.2354 | | 0.15686 | 0.093057 | 0.006039 | 0.54706 |
| ahb770 | 0.0948 | 0.1177 | 0.042613 | 0.004454 | 0.58893 | 0.0494 | 0.11765 | 0.042613 | 0.007650 | 0.63189 | 0.0557 | 0.11765 | 0.042613 | 0.00543 | 0.61286 | 0.0554 | | 0.07843 | 0.044707 | 0.004454 | 0.54545 |
| *est9912 | 0.4038 | 0.4428 | 0.152262 | 0.002108 | 0.58175 | 0.3736 | 0.47059 | 0.199887 | 0.003400 | 0.63466 | 0.3017 | 0.41796 | 0.125930 | 0.00425 | 0.60247 | 0.3380 | | 0.31373 | 0.163602 | 0.002108 | 0.53939 |
| ahb6199 | 0.4754 | 0.5035 | 0.183432 | 0.001721 | 0.58348 | 0.3522 | 0.47243 | 0.149145 | 0.000000 | 0.62185 | 0.4523 | 0.53125 | 0.233262 | 0.00403 | 0.61596 | 0.4052 | | 0.35417 | 0.190811 | 0.001721 | 0.54118 |
| AMB-00745371 | -0.0213 | 0.0621 | 0.001989 | 0.014470 | 0.54657 | 0.1509 | 0.32353 | 0.053749 | 0.040210 | 0.60643 | 0.0256 | 0.17182 | 0.017889 | 0.05833 | 0.58073 | 0.1586 | | 0.33023 | 0.088902 | 0.014470 | 0.52428 |
| ahb8254 | 0.2137 | 0.2663 | 0.074874 | 0.017529 | 0.58091 | 0.2039 | 0.29412 | 0.114674 | 0.001180 | 0.63182 | 0.1264 | 0.24149 | 0.055202 | 0.00641 | 0.59823 | 0.1627 | | 0.19608 | 0.087368 | 0.017529 | 0.53720 |
| AMB-00048598 | 0.1662 | 0.1944 | 0.072635 | 0.003314 | 0.58803 | 0.0439 | 0.05882 | 0.020832 | 0.003500 | 0.63328 | 0.3665 | 0.31579 | 0.124335 | 0.00532 | 0.61426 | 0.2489 | | 0.21053 | 0.094061 | 0.003314 | 0.54058 |
| ahb3091 | 0.5411 | 0.5817 | 0.226596 | 0.001812 | 0.58194 | 0.5934 | 0.55883 | 0.213063 | 0.002030 | 0.62736 | 0.6260 | 0.60217 | 0.239128 | 0.00170 | 0.60673 | 0.4738 | | 0.40145 | 0.189674 | 0.001812 | 0.52950 |
| AMB-00469637 | 0.2906 | 0.3143 | 0.123658 | 0.001623 | 0.58862 | 0.6531 | 0.56250 | 0.251473 | 0.001410 | 0.63560 | 0.1046 | 0.10526 | 0.037944 | 0.00233 | 0.61343 | 0.4710 | | 0.37500 | 0.189560 | 0.001623 | 0.54257 |
| est2326 | -0.0500 | 0.0000 | 0.000000 | 0.004376 | 0.59699 | -0.0500 | 0.00000 | 0.000000 | 0.001780 | 0.64308 | -0.0500 | 0.00000 | 0.000000 | 0.00739 | 0.62270 | -0.0500 | | 0.00000 | 0.000000 | 0.004376 | 0.55409 |
| AMB-00749439 | 0.0194 | 0.0625 | 0.022165 | 0.005030 | 0.58829 | -0.0500 | 0.00000 | 0.000000 | 0.005560 | 0.64115 | 0.0828 | 0.11111 | 0.040143 | 0.00724 | 0.61244 | 0.0641 | | 0.07407 | 0.042133 | 0.005030 | 0.54519 |
| est11018 | 0.5542 | 0.6291 | 0.213684 | 0.001697 | 0.58010 | 0.5039 | 0.58824 | 0.185619 | 0.002560 | 0.61825 | 0.5955 | 0.66564 | 0.241894 | 0.00184 | 0.60455 | 0.4895 | | 0.44376 | 0.192621 | 0.001697 | 0.52702 |
| AMB-00782039 | 0.1101 | 0.1389 | 0.050729 | 0.002880 | 0.58797 | -0.0500 | 0.00000 | 0.000000 | 0.000000 | 0.64257 | 0.3047 | 0.26316 | 0.101210 | 0.01109 | 0.61326 | 0.2588 | | 0.17544 | 0.105118 | 0.002880 | 0.54743 |
| *est2705 | 0.6017 | 0.6373 | 0.261622 | 0.000000 | 0.58332 | 0.5311 | 0.50000 | 0.180343 | 0.000000 | 0.62623 | 0.7791 | 0.76006 | 0.352983 | 0.00000 | 0.61083 | 0.5748 | | 0.50671 | 0.241771 | 0.000000 | 0.53608 |
| AMB-00546323 | 0.3792 | 0.3871 | 0.157612 | 0.001413 | 0.58847 | 0.3887 | 0.31250 | 0.122856 | 0.001810 | 0.63506 | 0.5695 | 0.46667 | 0.197811 | 0.00075 | 0.61567 | 0.3410 | | 0.31111 | 0.135430 | 0.001413 | 0.53996 |
| AMB-01102402 | 0.0542 | 0.0833 | 0.029787 | 0.002738 | 0.58800 | -0.0500 | 0.00000 | 0.000000 | 0.004660 | 0.64208 | 0.1740 | 0.15789 | 0.058110 | 0.00746 | 0.61379 | 0.1393 | | 0.10526 | 0.060805 | 0.002738 | 0.54663 |
| AMB-00732533 | 0.2790 | 0.3056 | 0.119748 | 0.002456 | 0.58777 | 0.5530 | 0.47059 | 0.199887 | 0.001710 | 0.63563 | 0.1740 | 0.15789 | 0.058110 | 0.00328 | 0.61185 | 0.3410 | | 0.31373 | 0.137426 | 0.002456 | 0.54082 |
| ahb6294 | 0.3674 | 0.4126 | 0.137123 | 0.002845 | 0.58116 | 0.2538 | 0.38236 | 0.107371 | 0.003880 | 0.62052 | 0.3498 | 0.44118 | 0.184546 | 0.00200 | 0.61476 | 0.3047 | | 0.29412 | 0.147252 | 0.002845 | 0.53980 |
| *est11786 | 0.2523 | 0.3268 | 0.085700 | 0.004285 | 0.57797 | 0.2867 | 0.38235 | 0.155322 | 0.004020 | 0.63414 | 0.1367 | 0.27709 | 0.054580 | 0.00702 | 0.59578 | 0.2053 | | 0.25490 | 0.109956 | 0.004285 | 0.53666 |
| est8514 | -0.0500 | 0.0000 | 0.000000 | 0.004015 | 0.59823 | -0.0500 | 0.00000 | 0.000000 | 0.004210 | 0.64423 | -0.0500 | 0.00000 | 0.000000 | 0.00398 | 0.62109 | -0.0500 | | 0.00000 | 0.000000 | 0.004015 | 0.55383 |
| ahb9803 | 0.3403 | 0.3840 | 0.124406 | 0.003449 | 0.58388 | 0.3152 | 0.41176 | 0.169703 | 0.003010 | 0.63487 | 0.2408 | 0.35913 | 0.100076 | 0.00259 | 0.60245 | 0.2777 | | 0.27451 | 0.136381 | 0.003449 | 0.53874 |
| AMB-00204536 | 0.1257 | 0.1471 | 0.053888 | 0.003194 | 0.58947 | 0.0742 | 0.14706 | 0.053888 | 0.003540 | 0.63297 | 0.0811 | 0.14706 | 0.053888 | 0.00555 | 0.61265 | 0.0819 | | 0.09804 | 0.056427 | 0.003194 | 0.54642 |
| AMB-00311256 | 0.1475 | 0.2067 | 0.052377 | 0.006483 | 0.57967 | 0.1508 | 0.23529 | 0.089413 | 0.005060 | 0.63393 | 0.0671 | 0.17973 | 0.034613 | 0.00746 | 0.59890 | 0.1060 | | 0.15686 | 0.064945 | 0.006483 | 0.53888 |
| AMB-01031112 | 0.1333 | 0.1667 | 0.061555 | 0.006485 | 0.58731 | 0.1223 | 0.11765 | 0.042613 | 0.006530 | 0.63341 | 0.2319 | 0.21053 | 0.079169 | 0.00158 | 0.61342 | 0.1077 | | 0.14035 | 0.052953 | 0.006485 | 0.53747 |
| ahb5549 | 0.5063 | 0.5962 | 0.192688 | 0.001767 | 0.57823 | 0.3558 | 0.46153 | 0.118478 | 0.001770 | 0.61370 | 0.6265 | 0.68826 | 0.260396 | 0.00402 | 0.60543 | 0.4595 | | 0.45884 | 0.179802 | 0.001767 | 0.52729 |
| ahb83 | 0.1662 | 0.1944 | 0.072635 | 0.003344 | 0.58763 | 0.2093 | 0.17647 | 0.065436 | 0.003680 | 0.63440 | 0.2406 | 0.21053 | 0.079169 | 0.00568 | 0.61360 | 0.1194 | | 0.14035 | 0.057595 | 0.003344 | 0.53604 |
| *ahb10470 | 0.4911 | 0.5523 | 0.191657 | 0.001789 | 0.57898 | 0.7082 | 0.70589 | 0.292838 | 0.001590 | 0.62774 | 0.3835 | 0.41486 | 0.121757 | 0.00224 | 0.60037 | 0.4762 | | 0.47059 | 0.196956 | 0.001789 | 0.52977 |
| AMB-00491794 | 0.3356 | 0.3611 | 0.145207 | 0.000855 | 0.58777 | 0.6120 | 0.52941 | 0.232214 | 0.002030 | 0.63685 | 0.2406 | 0.21053 | 0.079169 | 0.00167 | 0.61349 | 0.3786 | | 0.35294 | 0.156597 | 0.000855 | 0.54039 |
| ahb4928 | 0.1853 | 0.2484 | 0.067127 | 0.010001 | 0.57521 | 0.3975 | 0.38235 | 0.122559 | 0.004030 | 0.62230 | 0.0735 | 0.12848 | 0.026483 | 0.00526 | 0.59976 | 0.2215 | | 0.25490 | 0.084524 | 0.010001 | 0.52075 |
| AMB-00458656 | 0.1381 | 0.1667 | 0.061555 | 0.002298 | 0.58856 | -0.0500 | 0.00000 | 0.000000 | 0.000000 | 0.64260 | 0.3665 | 0.31579 | 0.124335 | 0.00507 | 0.61346 | 0.3172 | | 0.21053 | 0.128611 | 0.002298 | 0.54772 |
| ahb9730 | -0.0500 | 0.0000 | 0.000000 | 0.003659 | 0.59787 | -0.0500 | 0.00000 | 0.000000 | 0.004380 | 0.64123 | -0.0500 | 0.00000 | 0.000000 | 0.00478 | 0.62159 | -0.0500 | | 0.00000 | 0.000000 | 0.003659 | 0.55414 |
| est5732 | 0.1400 | 0.2745 | 0.045765 | 0.008837 | 0.56779 | 0.1024 | 0.26471 | 0.042051 | 0.009230 | 0.60625 | 0.1278 | 0.28329 | 0.049284 | 0.00961 | 0.59199 | 0.0945 | | 0.18886 | 0.042039 | 0.008837 | 0.47068 |
| AMB-01080880 | 0.4287 | 0.4967 | 0.161494 | 0.002778 | 0.57806 | 0.5239 | 0.52942 | 0.178895 | 0.002680 | 0.62251 | 0.4447 | 0.46750 | 0.146697 | 0.00323 | 0.60273 | 0.3625 | | 0.35295 | 0.138257 | 0.002778 | 0.52283 |
| AMB-00169185 | 0.1856 | 0.2222 | 0.083979 | 0.002746 | 0.58712 | 0.3413 | 0.29412 | 0.114674 | 0.004170 | 0.63520 | 0.1628 | 0.15789 | 0.058110 | 0.00412 | 0.61303 | 0.1644 | | 0.19608 | 0.076551 | 0.002746 | 0.53731 |
| *ahb3558 | 0.3640 | 0.3889 | 0.158479 | 0.000730 | 0.58934 | 0.2854 | 0.23529 | 0.089413 | 0.001380 | 0.63294 | 0.5919 | 0.52632 | 0.230453 | 0.00143 | 0.61564 | 0.3815 | | 0.35088 | 0.154095 | 0.000730 | 0.54052 |
| ahb5258 | 0.0941 | 0.1782 | 0.033780 | 0.008627 | 0.57420 | 0.0601 | 0.17647 | 0.032917 | 0.015320 | 0.61471 | 0.0671 | 0.17973 | 0.034613 | 0.01540 | 0.59948 | 0.0548 | | 0.11982 | 0.032053 | 0.008627 | 0.50582 |
| AMB-00599118 | 0.0619 | 0.1111 | 0.040143 | 0.004886 | 0.58782 | 0.0210 | 0.05882 | 0.020832 | 0.003970 | 0.63440 | 0.1343 | 0.15789 | 0.058110 | 0.00889 | 0.61249 | 0.0565 | | 0.10526 | 0.039433 | 0.004886 | 0.53694 |
| ahb5073 | 0.0960 | 0.2375 | 0.033065 | 0.009337 | 0.56654 | 0.0267 | 0.18750 | 0.019667 | 0.012870 | 0.59907 | 0.1245 | 0.27961 | 0.048162 | 0.01100 | 0.59171 | 0.0629 | | 0.18641 | 0.033373 | 0.009337 | 0.46958 |
| AMB-00659936 | 0.5972 | 0.6066 | 0.213429 | 0.000965 | 0.58115 | 0.4877 | 0.57466 | 0.185252 | 0.000870 | 0.62125 | 0.6027 | 0.63675 | 0.245115 | 0.00209 | 0.60743 | 0.4592 | | 0.42450 | 0.202802 | 0.000965 | 0.53278 |
| est6761 | 0.2652 | 0.2813 | 0.109031 | 0.001864 | 0.59041 | 0.1899 | 0.28125 | 0.109031 | 0.001920 | 0.63333 | 0.2000 | 0.28125 | 0.109031 | 0.00198 | 0.61354 | 0.2042 | | 0.18750 | 0.113086 | 0.001864 | 0.54614 |
| AMB-01156320 | 0.1934 | 0.2121 | 0.079822 | 0.002625 | 0.58847 | 0.1269 | 0.21212 | 0.079822 | 0.002640 | 0.63205 | 0.1398 | 0.21212 | 0.079822 | 0.00426 | 0.61373 | 0.1404 | | 0.14141 | 0.083213 | 0.002625 | 0.54494 |
| AMB-00927571 | -0.0144 | 0.0686 | 0.003681 | 0.037318 | 0.55826 | -0.0366 | 0.05883 | 0.002226 | 0.033420 | 0.59281 | 0.0736 | 0.18266 | 0.036199 | 0.01833 | 0.59691 | 0.0398 | | 0.16099 | 0.039464 | 0.037318 | 0.51248 |
| est4616 | 0.2796 | 0.2941 | 0.114674 | 0.001436 | 0.58970 | 0.2039 | 0.29412 | 0.114674 | 0.001180 | 0.63409 | 0.2135 | 0.29412 | 0.114674 | 0.00265 | 0.61417 | 0.2185 | | 0.19608 | 0.118821 | 0.001436 | 0.54672 |
| AMB-00435003 | 0.0263 | 0.0556 | 0.019651 | 0.006955 | 0.58856 | -0.0500 | 0.00000 | 0.000000 | 0.005160 | 0.64180 | 0.1046 | 0.10526 | 0.037944 | 0.00170 | 0.61302 | 0.0781 | | 0.07017 | 0.039840 | 0.006955 | 0.54536 |
| AMB-00627065 | 0.4038 | 0.4428 | 0.152262 | 0.001656 | 0.58346 | 0.2843 | 0.41177 | 0.120355 | 0.000000 | 0.62179 | 0.3849 | 0.47059 | 0.199887 | 0.00503 | 0.61495 | 0.3386 | | 0.31373 | 0.160955 | 0.001656 | 0.54155 |
| AMB-00411475 | 0.5761 | 0.6158 | 0.246318 | 0.000158 | 0.58198 | 0.5365 | 0.50208 | 0.180113 | 0.000000 | 0.62532 | 0.7206 | 0.70559 | 0.308313 | 0.00068 | 0.60992 | 0.5334 | | 0.47039 | 0.215586 | 0.000158 | 0.53381 |
| *est2661 | 0.0263 | 0.0556 | 0.019651 | 0.006411 | 0.58812 | 0.0439 | 0.05882 | 0.020832 | 0.006760 | 0.63469 | 0.0325 | 0.05263 | 0.018596 | 0.00485 | 0.61358 | -0.0017 | | 0.03921 | 0.015481 | 0.006411 | 0.53920 |
| est474 | 0.3073 | 0.3333 | 0.132304 | 0.000000 | 0.58808 | 0.7221 | 0.64706 | 0.304877 | 0.000000 | 0.63670 | 0.0325 | 0.05263 | 0.018596 | 0.00000 | 0.61457 | 0.6093 | | 0.43137 | 0.258003 | 0.000000 | 0.54567 |
| AMB-00644544 | 0.0423 | 0.1209 | 0.018626 | 0.018300 | 0.57342 | 0.0133 | 0.11765 | 0.017352 | 0.018560 | 0.61518 | 0.0248 | 0.12384 | 0.019830 | 0.01236 | 0.59802 | 0.0114 | | 0.08256 | 0.017512 | 0.018300 | 0.50557 |
| ahb10207 | 0.3516 | 0.4477 | 0.120119 | 0.001905 | 0.57687 | 0.4648 | 0.55882 | 0.249292 | 0.002160 | 0.63471 | 0.1850 | 0.34829 | 0.065875 | 0.00338 | 0.59053 | 0.3185 | | 0.37255 | 0.169023 | 0.001905 | 0.54057 |
| AMB-00283205 | 0.3349 | 0.3777 | 0.121009 | 0.002919 | 0.58138 | 0.3079 | 0.40625 | 0.166972 | 0.003290 | 0.63398 | 0.2376 | 0.35362 | 0.097771 | 0.00217 | 0.60178 | 0.2708 | | 0.27083 | 0.133925 | 0.002919 | 0.54046 |
| AMB-00387866 | 0.5920 | 0.6552 | 0.235206 | 0.000170 | 0.58109 | 0.5283 | 0.61765 | 0.205490 | 0.000130 | 0.62149 | 0.6162 | 0.68886 | 0.265604 | 0.00080 | 0.60693 | 0.5285 | | 0.45924 | 0.215637 | 0.000170 | 0.53042 |
| AMB-00258552 | 0.4038 | 0.4428 | 0.152262 | 0.001725 | 0.58211 | 0.3736 | 0.47059 | 0.199887 | 0.002310 | 0.63434 | 0.3017 | 0.41796 | 0.125930 | 0.00224 | 0.60413 | 0.3380 | | 0.31373 | 0.163602 | 0.001725 | 0.54075 |
| AMB-00673354 | -0.0016 | 0.0278 | 0.009725 | 0.004726 | 0.58919 | -0.0500 | 0.00000 | 0.000000 | 0.003250 | 0.64188 | 0.0325 | 0.05263 | 0.018596 | 0.01302 | 0.61231 | 0.0159 | | 0.03509 | 0.019589 | 0.004726 | 0.54501 |
| *ahb11929 | 0.5109 | 0.5539 | 0.210225 | 0.001297 | 0.58079 | 0.4658 | 0.44118 | 0.150281 | 0.001720 | 0.62294 | 0.6788 | 0.65480 | 0.273388 | 0.00116 | 0.60792 | 0.4723 | | 0.43653 | 0.188232 | 0.001297 | 0.53243 |
| AMB-00060184 | 0.0102 | 0.1373 | 0.009834 | 0.023645 | 0.55246 | 0.0191 | 0.17647 | 0.016598 | 0.027260 | 0.59160 | -0.0208 | 0.10217 | 0.005369 | 0.02533 | 0.57093 | -0.0104 | | 0.11765 | 0.011157 | 0.023645 | 0.40760 |
| ahb6768 | 0.3475 | 0.4395 | 0.122477 | 0.004279 | 0.57470 | 0.3902 | 0.44117 | 0.123230 | 0.004790 | 0.61609 | 0.3804 | 0.43808 | 0.121805 | 0.00198 | 0.59817 | 0.2817 | | 0.29411 | 0.104169 | 0.004279 | 0.51032 |
| AMB-00743044 | 0.3020 | 0.3595 | 0.112359 | 0.004295 | 0.57796 | 0.1760 | 0.20588 | 0.051692 | 0.001060 | 0.62062 | 0.5151 | 0.49691 | 0.178698 | 0.00469 | 0.60643 | 0.3229 | | 0.33127 | 0.120182 | 0.004295 | 0.52661 |
| AMB-00463059 | 0.1890 | 0.2680 | 0.063529 | 0.005849 | 0.57626 | 0.2311 | 0.32353 | 0.127834 | 0.005340 | 0.63329 | 0.0831 | 0.21827 | 0.036725 | 0.00225 | 0.59328 | 0.1506 | | 0.21569 | 0.088249 | 0.005849 | 0.53746 |
| AMB-00937767 | -0.0500 | 0.0000 | 0.000000 | 0.004489 | 0.59650 | -0.0500 | 0.00000 | 0.000000 | 0.002220 | 0.64170 | -0.0500 | 0.00000 | 0.000000 | 0.00322 | 0.62056 | -0.0500 | | 0.00000 | 0.000000 | 0.004489 | 0.55481 |
| AMB-00263086 | 0.3991 | 0.4420 | 0.151295 | 0.001735 | 0.58200 | 0.2843 | 0.41177 | 0.120355 | 0.001960 | 0.62165 | 0.3793 | 0.47059 | 0.199887 | 0.00195 | 0.61430 | 0.3350 | | 0.31373 | 0.160955 | 0.001735 | 0.53974 |
| AMB-00949801 | 0.0263 | 0.0556 | 0.019651 | 0.007835 | 0.58794 | 0.0439 | 0.05882 | 0.020832 | 0.006930 | 0.63370 | 0.0325 | 0.05263 | 0.018596 | 0.00238 | 0.61404 | -0.0017 | | 0.03921 | 0.015481 | 0.007835 | 0.53822 |
| est5271 | 0.4054 | 0.5098 | 0.140762 | 0.002346 | 0.57629 | 0.2908 | 0.44118 | 0.101701 | 0.003130 | 0.61065 | 0.4625 | 0.57121 | 0.186136 | 0.00295 | 0.60348 | 0.3481 | | 0.38081 | 0.136512 | 0.002346 | 0.52171 |
| est11295 | 0.0821 | 0.1111 | 0.040143 | 0.003433 | 0.58962 | 0.1289 | 0.11765 | 0.042613 | 0.005450 | 0.63488 | 0.1046 | 0.10526 | 0.037944 | 0.00343 | 0.61329 | 0.0453 | | 0.07843 | 0.031729 | 0.003433 | 0.53649 |
| ahb7265 | 0.0017 | 0.0294 | 0.010303 | 0.006655 | 0.58859 | -0.0228 | 0.02941 | 0.010303 | 0.002740 | 0.63341 | -0.0186 | 0.02941 | 0.010303 | 0.00578 | 0.61384 | -0.0221 | | 0.01961 | 0.010868 | 0.006655 | 0.54534 |
| AMB-00324781 | -0.0500 | 0.0000 | 0.000000 | 0.001643 | 0.59729 | -0.0500 | 0.00000 | 0.000000 | 0.003810 | 0.64231 | -0.0500 | 0.00000 | 0.000000 | 0.00858 | 0.62181 | -0.0500 | | 0.00000 | 0.000000 | 0.001643 | 0.55285 |
| AMB-00082672 | 0.1566 | 0.1765 | 0.065436 | 0.002580 | 0.58832 | 0.0993 | 0.17647 | 0.065436 | 0.002540 | 0.63327 | 0.1069 | 0.17647 | 0.065436 | 0.00349 | 0.61315 | 0.1086 | | 0.11765 | 0.068385 | 0.002580 | 0.54465 |
| *est2210 | 0.0263 | 0.0556 | 0.019651 | 0.003580 | 0.58708 | -0.0500 | 0.00000 | 0.000000 | 0.003880 | 0.64260 | 0.1046 | 0.10526 | 0.037944 | 0.00420 | 0.61332 | 0.0781 | | 0.07017 | 0.039840 | 0.003580 | 0.54510 |
| ahb6643 | -0.0500 | 0.0000 | 0.000000 | 0.003085 | 0.59849 | -0.0500 | 0.00000 | 0.000000 | 0.000000 | 0.64136 | -0.0500 | 0.00000 | 0.000000 | 0.00690 | 0.62275 | -0.0500 | | 0.00000 | 0.000000 | 0.003085 | 0.55392 |
| 1535 | 0.2451 | 0.037420 | 0.004759 | 0.56709 | -0.0155 | 0.11764 | 0.007602 | 0.006130 | 0.58956 | 0.2408 | 0.35913 | 0.100076 | 0.01375 | 0.60296 | 0.1246 | 0.23942 | | 0.068937 | 0.004759 | 0.52117 | 0.035 |
| est7565 | 0.0314 | 0.0899 | 0.016029 | 0.010908 | 0.57859 | -0.0258 | 0.05883 | 0.005472 | 0.003130 | 0.61463 | 0.0557 | 0.11765 | 0.042613 | 0.00948 | 0.61250 | 0.0130 | | 0.07843 | 0.028409 | 0.010908 | 0.53728 |
| AMB-00533838 | 0.1308 | 0.1667 | 0.061555 | 0.001807 | 0.58882 | 0.1188 | 0.11765 | 0.042613 | 0.002620 | 0.63313 | 0.2274 | 0.21053 | 0.079169 | 0.00900 | 0.61332 | 0.1047 | | 0.14035 | 0.052953 | 0.001807 | 0.53823 |
| AMB-00563464 | 0.1101 | 0.1389 | 0.050729 | 0.002338 | 0.58640 | -0.0500 | 0.00000 | 0.000000 | 0.000000 | 0.64180 | 0.3047 | 0.26316 | 0.101210 | 0.00488 | 0.61301 | 0.2588 | | 0.17544 | 0.105118 | 0.002338 | 0.54747 |
| AMB-00619361 | -0.0500 | 0.0000 | 0.000000 | 0.002969 | 0.59692 | -0.0500 | 0.00000 | 0.000000 | 0.000000 | 0.64116 | -0.0500 | 0.00000 | 0.000000 | 0.00841 | 0.62214 | -0.0500 | | 0.00000 | 0.000000 | 0.002969 | 0.55509 |
| ahb2669 | 0.1823 | 0.2369 | 0.063708 | 0.004896 | 0.58008 | 0.1772 | 0.26471 | 0.101874 | 0.004850 | 0.63493 | 0.0996 | 0.21208 | 0.045399 | 0.00170 | 0.59921 | 0.1353 | | 0.17647 | 0.076227 | 0.004896 | 0.53715 |
| AMB-00821262 | 0.1652 | 0.3065 | 0.064502 | 0.004087 | 0.56983 | 0.0724 | 0.20724 | 0.033680 | 0.004360 | 0.60765 | 0.2751 | 0.39474 | 0.098256 | 0.00920 | 0.59691 | 0.1332 | | 0.26316 | 0.065555 | 0.004087 | 0.49748 |
| ahb9822 | 0.2790 | 0.3056 | 0.119748 | 0.000256 | 0.58823 | 0.0439 | 0.05882 | 0.020832 | 0.000290 | 0.63223 | 0.5919 | 0.52632 | 0.230453 | 0.00064 | 0.61573 | 0.4776 | | 0.35088 | 0.188298 | 0.000256 | 0.54380 |
| *AMB-01023229 | 0.1276 | 0.2092 | 0.043646 | 0.007196 | 0.57489 | 0.1772 | 0.26471 | 0.101874 | 0.009810 | 0.63228 | 0.0348 | 0.15945 | 0.021659 | 0.00000 | 0.59264 | 0.0996 | | 0.17647 | 0.068688 | 0.007196 | 0.53743 |
| AMB-00480741 | 0.1510 | 0.2075 | 0.053034 | 0.004219 | 0.57927 | 0.1508 | 0.23529 | 0.089413 | 0.002450 | 0.63155 | 0.0736 | 0.18266 | 0.036199 | 0.00868 | 0.59759 | 0.1084 | | 0.15686 | 0.065553 | 0.004219 | 0.53900 |
| ahb9759 | -0.0500 | 0.0000 | 0.000000 | 0.002081 | 0.59798 | -0.0500 | 0.00000 | 0.000000 | 0.001760 | 0.64186 | -0.0500 | 0.00000 | 0.000000 | 0.00538 | 0.62264 | -0.0500 | | 0.00000 | 0.000000 | 0.002081 | 0.55470 |
| AMB-01115863 | 0.0328 | 0.0588 | 0.020832 | 0.003098 | 0.58973 | 0.0009 | 0.05882 | 0.020832 | 0.002810 | 0.63319 | 0.0058 | 0.05882 | 0.020832 | 0.00556 | 0.61283 | 0.0035 | | 0.03921 | 0.021936 | 0.003098 | 0.54594 |
| AMB-00598633 | -0.0317 | 0.0029 | 0.000034 | 0.007278 | 0.57909 | -0.0252 | 0.02941 | 0.010303 | 0.012230 | 0.63418 | -0.0319 | 0.03726 | 0.003890 | 0.01011 | 0.60064 | -0.0225 | | 0.04445 | 0.015842 | 0.007278 | 0.53726 |
| AMB-00587256 | 0.1101 | 0.1389 | 0.050729 | 0.002131 | 0.58763 | -0.0500 | 0.00000 | 0.000000 | 0.001760 | 0.64307 | 0.3047 | 0.26316 | 0.101210 | 0.00170 | 0.61308 | 0.2588 | | 0.17544 | 0.105118 | 0.002131 | 0.54726 |
| est7937 | 0.3493 | 0.4150 | 0.123258 | 0.002937 | 0.57804 | 0.2843 | 0.41177 | 0.120355 | 0.002290 | 0.62280 | 0.3017 | 0.41796 | 0.125930 | 0.00271 | 0.60403 | 0.2834 | | 0.27864 | 0.118216 | 0.002937 | 0.52689 |
| AMB-01141408 | 0.1257 | 0.1471 | 0.053888 | 0.002164 | 0.58912 | 0.0742 | 0.14706 | 0.053888 | 0.002620 | 0.63190 | 0.0811 | 0.14706 | 0.053888 | 0.00356 | 0.61373 | 0.0819 | | 0.09804 | 0.056427 | 0.002164 | 0.54473 |
| AMB-00197068 | 0.1048 | 0.2614 | 0.035426 | 0.004441 | 0.56173 | -0.0371 | 0.05883 | 0.002226 | 0.005230 | 0.59597 | 0.4311 | 0.54799 | 0.162860 | 0.00246 | 0.59984 | 0.3307 | | 0.40455 | 0.160289 | 0.004441 | 0.53404 |
| AMB-00176986 | -0.0017 | 0.0286 | 0.010006 | 0.002904 | 0.59078 | -0.0500 | 0.00000 | 0.000000 | 0.001760 | 0.64231 | 0.0307 | 0.05263 | 0.018596 | 0.00667 | 0.61454 | 0.0138 | | 0.03509 | 0.019589 | 0.002904 | 0.54552 |
| AMB-01037165 | 0.2182 | 0.2353 | 0.089413 | 0.001524 | 0.58994 | 0.1508 | 0.23529 | 0.089413 | 0.001370 | 0.63148 | 0.1595 | 0.23529 | 0.089413 | 0.00302 | 0.61409 | 0.1629 | | 0.15686 | 0.093057 | 0.001524 | 0.54363 |
| AMB-01077028 | 0.1662 | 0.1944 | 0.072635 | 0.001430 | 0.58704 | 0.1289 | 0.11765 | 0.042613 | 0.005930 | 0.63197 | 0.3047 | 0.26316 | 0.101210 | 0.00415 | 0.61293 | 0.1607 | | 0.17544 | 0.067670 | 0.001430 | 0.53755 |
| AMB-00137321 | 0.1698 | 0.3039 | 0.054842 | 0.003961 | 0.56747 | 0.1301 | 0.29412 | 0.050794 | 0.004930 | 0.60684 | 0.1566 | 0.31270 | 0.058663 | 0.01066 | 0.59290 | 0.1204 | | 0.20847 | 0.050386 | 0.003961 | 0.47743 |
| *est1790 | 0.1040 | 0.1429 | 0.052261 | 0.001684 | 0.58847 | -0.0500 | 0.00000 | 0.000000 | 0.002030 | 0.64140 | 0.2799 | 0.26316 | 0.101210 | 0.00338 | 0.61307 | 0.2442 | | 0.17544 | 0.105118 | 0.001684 | 0.54570 |
| est3134 | 0.4106 | 0.5180 | 0.144044 | 0.001572 | 0.57447 | 0.3634 | 0.47059 | 0.119871 | 0.002220 | 0.61408 | 0.4702 | 0.56037 | 0.168218 | 0.00385 | 0.60049 | 0.3473 | | 0.37358 | 0.128476 | 0.001572 | 0.51632 |
| est1848 | 0.3403 | 0.3840 | 0.124406 | 0.001739 | 0.58131 | 0.3152 | 0.41176 | 0.169703 | 0.001910 | 0.63294 | 0.2408 | 0.35913 | 0.100076 | 0.00271 | 0.60275 | 0.2777 | | 0.27451 | 0.136381 | 0.001739 | 0.54106 |
| AMB-00113737 | 0.1282 | 0.2418 | 0.042673 | 0.003012 | 0.57170 | -0.0130 | 0.11765 | 0.008376 | 0.002860 | 0.59280 | 0.2690 | 0.35294 | 0.141375 | 0.00507 | 0.61344 | 0.1435 | | 0.23529 | 0.096637 | 0.003012 | 0.54051 |
| ahb4778 | 0.2507 | 0.2778 | 0.107520 | 0.000927 | 0.58805 | 0.1289 | 0.11765 | 0.042613 | 0.001090 | 0.63257 | 0.4832 | 0.42105 | 0.174338 | 0.00143 | 0.61521 | 0.3198 | | 0.28070 | 0.123122 | 0.000927 | 0.54081 |
| est7877 | 0.4508 | 0.4984 | 0.179474 | 0.000074 | 0.58069 | 0.2523 | 0.26471 | 0.073340 | 0.000190 | 0.61996 | 0.7298 | 0.70743 | 0.311061 | 0.00020 | 0.60962 | 0.5203 | | 0.47162 | 0.211286 | 0.000074 | 0.53436 |
| est1933 | 0.5109 | 0.5539 | 0.210225 | 0.000000 | 0.58144 | 0.7099 | 0.67647 | 0.288447 | 0.000000 | 0.62699 | 0.4568 | 0.44427 | 0.151803 | 0.00000 | 0.60429 | 0.4777 | | 0.45098 | 0.197456 | 0.000000 | 0.53190 |
| ahb12208 | 0.1853 | 0.2484 | 0.067127 | 0.003657 | 0.57633 | 0.4658 | 0.44118 | 0.150281 | 0.004610 | 0.62462 | 0.0120 | 0.07585 | 0.012103 | 0.00339 | 0.59962 | 0.3094 | | 0.29412 | 0.115957 | 0.003657 | 0.52899 |
| est8071 | 0.2507 | 0.2778 | 0.107520 | 0.000972 | 0.58912 | 0.4910 | 0.41176 | 0.169703 | 0.000470 | 0.63576 | 0.1740 | 0.15789 | 0.058110 | 0.00157 | 0.61271 | 0.2829 | | 0.27451 | 0.114839 | 0.000972 | 0.53949 |
| AMB-01077554 | -0.0091 | 0.0948 | 0.005005 | 0.007996 | 0.55325 | 0.1270 | 0.29412 | 0.044565 | 0.008350 | 0.60397 | -0.0242 | 0.08359 | 0.004646 | 0.00581 | 0.57927 | 0.0964 | | 0.25181 | 0.056533 | 0.007996 | 0.50742 |
| AMB-00702326 | 0.0170 | 0.0909 | 0.032589 | 0.003557 | 0.58732 | 0.0649 | 0.13333 | 0.048593 | 0.002890 | 0.63251 | -0.0147 | 0.05556 | 0.019651 | 0.00758 | 0.61284 | 0.0047 | | 0.08889 | 0.032632 | 0.003557 | 0.53689 |
| ahb4529 | 0.0600 | 0.1193 | 0.024272 | 0.004505 | 0.57804 | 0.0742 | 0.14706 | 0.053888 | 0.003300 | 0.63209 | 0.0026 | 0.09443 | 0.012860 | 0.01446 | 0.60005 | 0.0332 | | 0.09804 | 0.036736 | 0.004505 | 0.53745 |
| ahb8709 | 0.0028 | 0.0313 | 0.010954 | 0.003893 | 0.58960 | -0.0243 | 0.03125 | 0.010954 | 0.005570 | 0.63372 | -0.0175 | 0.03125 | 0.010954 | 0.00170 | 0.61404 | -0.0222 | | 0.02083 | 0.011554 | 0.003893 | 0.54591 |
| est8831 | 0.2796 | 0.2941 | 0.114674 | 0.000262 | 0.58935 | 0.2039 | 0.29412 | 0.114674 | 0.000000 | 0.63417 | 0.2135 | 0.29412 | 0.114674 | 0.00147 | 0.61275 | 0.2185 | | 0.19608 | 0.118821 | 0.000262 | 0.54682 |
| AMB-00300732 | 0.1662 | 0.1944 | 0.072635 | 0.000000 | 0.58938 | -0.0500 | 0.00000 | 0.000000 | 0.000000 | 0.64214 | 0.4259 | 0.36842 | 0.148663 | 0.00000 | 0.61457 | 0.3746 | | 0.24561 | 0.153111 | 0.000000 | 0.54864 |
| AMB-00910592 | -0.0209 | 0.0262 | 0.002133 | 0.009929 | 0.57651 | -0.0228 | 0.02941 | 0.010303 | 0.012720 | 0.63285 | 0.0120 | 0.07585 | 0.012103 | 0.00338 | 0.59978 | 0.0157 | | 0.07017 | 0.026794 | 0.009929 | 0.53665 |
| AMB-00483832 | 0.0308 | 0.0833 | 0.029787 | 0.002284 | 0.58687 | -0.0500 | 0.00000 | 0.000000 | 0.002040 | 0.64185 | 0.1208 | 0.15789 | 0.058110 | 0.00325 | 0.61226 | 0.1115 | | 0.10526 | 0.060805 | 0.002284 | 0.54744 |
| AMB-00456540 | 0.0821 | 0.1111 | 0.040143 | 0.001771 | 0.58724 | 0.2854 | 0.23529 | 0.089413 | 0.001760 | 0.63306 | -0.0500 | 0.00000 | 0.000000 | 0.00170 | 0.62300 | 0.2354 | | 0.15686 | 0.093057 | 0.001771 | 0.54781 |
| AMB-00652385 | 0.4320 | 0.5049 | 0.156034 | 0.001464 | 0.57863 | 0.4132 | 0.52942 | 0.178895 | 0.001630 | 0.62429 | 0.3558 | 0.48298 | 0.138416 | 0.00184 | 0.60035 | 0.3645 | | 0.35295 | 0.150614 | 0.001464 | 0.52666 |
| ahb10133 | 0.2519 | 0.3595 | 0.082787 | 0.002676 | 0.57401 | 0.2843 | 0.41177 | 0.120355 | 0.004030 | 0.62162 | 0.1566 | 0.31270 | 0.058663 | 0.00439 | 0.59356 | 0.2018 | | 0.27451 | 0.087757 | 0.002676 | 0.51331 |
| ahb7607 | -0.0165 | 0.0507 | 0.003210 | 0.007311 | 0.56590 | 0.0250 | 0.08824 | 0.031599 | 0.001510 | 0.63310 | 0.0745 | 0.17492 | 0.027420 | 0.00932 | 0.59081 | 0.1008 | | 0.17544 | 0.069580 | 0.007311 | 0.53775 |
| AMB-00072036 | 0.1256 | 0.1936 | 0.046210 | 0.004689 | 0.57460 | 0.1813 | 0.21774 | 0.054587 | 0.003050 | 0.61960 | 0.1184 | 0.16774 | 0.037687 | 0.00939 | 0.59878 | 0.0841 | | 0.14516 | 0.039168 | 0.004689 | 0.49408 |
| est10383 | 0.0542 | 0.0833 | 0.029787 | 0.002066 | 0.58772 | -0.0500 | 0.00000 | 0.000000 | 0.000000 | 0.64170 | 0.1740 | 0.15789 | 0.058110 | 0.00593 | 0.61331 | 0.1393 | | 0.10526 | 0.060805 | 0.002066 | 0.54598 |
| AMB-00905128 | 0.3403 | 0.3840 | 0.124406 | 0.001033 | 0.58153 | 0.3152 | 0.41176 | 0.169703 | 0.001860 | 0.63411 | 0.2408 | 0.35913 | 0.100076 | 0.00148 | 0.60316 | 0.2777 | | 0.27451 | 0.136381 | 0.001033 | 0.54061 |
| ahb6619 | 0.0239 | 0.0849 | 0.014358 | 0.007521 | 0.57485 | -0.0342 | 0.02941 | 0.002612 | 0.007970 | 0.62096 | 0.0853 | 0.13726 | 0.029127 | 0.00598 | 0.59757 | 0.0308 | | 0.09151 | 0.021747 | 0.007521 | 0.50419 |
| est7832 | 0.1662 | 0.1944 | 0.072635 | 0.001749 | 0.58762 | 0.0439 | 0.05882 | 0.020832 | 0.001880 | 0.63353 | 0.3665 | 0.31579 | 0.124335 | 0.00176 | 0.61382 | 0.2489 | | 0.21053 | 0.094061 | 0.001749 | 0.54024 |
| AMB-00569121 | 0.3893 | 0.4268 | 0.144445 | 0.000535 | 0.58109 | 0.3596 | 0.45455 | 0.191455 | 0.000510 | 0.63404 | 0.2862 | 0.40192 | 0.118643 | 0.00082 | 0.60469 | 0.3225 | | 0.30303 | 0.155991 | 0.000535 | 0.54040 |
| *AMB-00675461 | -0.0016 | 0.0278 | 0.009725 | 0.002738 | 0.58942 | -0.0500 | 0.00000 | 0.000000 | 0.002400 | 0.64303 | 0.0325 | 0.05263 | 0.018596 | 0.00331 | 0.61361 | 0.0159 | | 0.03509 | 0.019589 | 0.002738 | 0.54529 |
| AMB-00900468 | 0.0729 | 0.1373 | 0.029127 | 0.005436 | 0.57434 | 0.0990 | 0.14706 | 0.032152 | 0.007900 | 0.61769 | 0.0735 | 0.12848 | 0.026483 | 0.00491 | 0.59721 | 0.0377 | | 0.09804 | 0.024475 | 0.005436 | 0.49182 |
| ahb8094 | 0.2022 | 0.2990 | 0.066669 | 0.002531 | 0.57595 | 0.2867 | 0.38235 | 0.155322 | 0.002730 | 0.63312 | 0.0747 | 0.22446 | 0.032699 | 0.00297 | 0.58812 | 0.1745 | | 0.25490 | 0.104396 | 0.002531 | 0.53785 |
| *ahb9669 | 0.4287 | 0.4967 | 0.161494 | 0.000782 | 0.57778 | 0.4573 | 0.47059 | 0.148230 | 0.000000 | 0.62202 | 0.5045 | 0.52013 | 0.173853 | 0.00300 | 0.60345 | 0.3615 | | 0.34675 | 0.136405 | 0.000782 | 0.52331 |
| *est2789 | -0.0020 | 0.0539 | 0.007104 | 0.004470 | 0.57489 | -0.0228 | 0.02941 | 0.010303 | 0.004170 | 0.63409 | 0.0735 | 0.12848 | 0.026483 | 0.00700 | 0.59852 | 0.0687 | | 0.10526 | 0.043999 | 0.004470 | 0.53955 |
| AMB-00768826 | 0.0263 | 0.0556 | 0.019651 | 0.002662 | 0.58854 | -0.0500 | 0.00000 | 0.000000 | 0.000000 | 0.64207 | 0.1046 | 0.10526 | 0.037944 | 0.00545 | 0.61191 | 0.0781 | | 0.07017 | 0.039840 | 0.002662 | 0.54373 |
| AMB-00908557 | -0.0016 | 0.0278 | 0.009725 | 0.002385 | 0.58846 | -0.0500 | 0.00000 | 0.000000 | 0.002820 | 0.64349 | 0.0325 | 0.05263 | 0.018596 | 0.00253 | 0.61468 | 0.0159 | | 0.03509 | 0.019589 | 0.002385 | 0.54629 |
| est4505 | 0.0863 | 0.1143 | 0.041341 | 0.000694 | 0.58790 | -0.0500 | 0.00000 | 0.000000 | 0.000130 | 0.64360 | 0.2406 | 0.21053 | 0.079169 | 0.00218 | 0.61279 | 0.1989 | | 0.14035 | 0.082542 | 0.000694 | 0.54787 |
| AMB-00549036 | 0.1110 | 0.1429 | 0.052261 | 0.002148 | 0.58823 | 0.0467 | 0.06250 | 0.022165 | 0.002140 | 0.63293 | 0.2319 | 0.21053 | 0.079169 | 0.00246 | 0.61170 | 0.1292 | | 0.14035 | 0.055384 | 0.002148 | 0.53829 |
| AMB-00508572 | 0.2987 | 0.3873 | 0.100934 | 0.000778 | 0.57738 | 0.1301 | 0.29412 | 0.050794 | 0.000440 | 0.60860 | 0.3849 | 0.47059 | 0.199887 | 0.00175 | 0.61498 | 0.2630 | | 0.31373 | 0.135589 | 0.000778 | 0.53980 |
| est9134 | 0.0542 | 0.0833 | 0.029787 | 0.001084 | 0.58928 | -0.0500 | 0.00000 | 0.000000 | 0.000000 | 0.64200 | 0.1740 | 0.15789 | 0.058110 | 0.00337 | 0.61303 | 0.1393 | | 0.10526 | 0.060805 | 0.001084 | 0.54636 |
| est5056 | 0.1197 | 0.2372 | 0.040410 | 0.003099 | 0.56963 | 0.0184 | 0.16734 | 0.017934 | 0.002910 | 0.59948 | 0.1789 | 0.29928 | 0.075048 | 0.00851 | 0.59955 | 0.0937 | | 0.19952 | 0.050252 | 0.003099 | 0.50827 |
| *est8860 | -0.0016 | 0.0278 | 0.009725 | 0.002487 | 0.58853 | -0.0500 | 0.00000 | 0.000000 | 0.001760 | 0.64345 | 0.0325 | 0.05263 | 0.018596 | 0.00264 | 0.61468 | 0.0159 | | 0.03509 | 0.019589 | 0.002487 | 0.54574 |
| ahb10678 | -0.0500 | 0.0000 | 0.000000 | 0.001951 | 0.59780 | -0.0500 | 0.00000 | 0.000000 | 0.001760 | 0.64037 | -0.0500 | 0.00000 | 0.000000 | 0.00181 | 0.62040 | -0.0500 | | 0.00000 | 0.000000 | 0.001951 | 0.55470 |
| AMB-00996696 | 0.0655 | 0.1846 | 0.024512 | 0.003525 | 0.56638 | -0.0266 | 0.08824 | 0.004849 | 0.005940 | 0.59096 | 0.1540 | 0.27090 | 0.065581 | 0.00586 | 0.59837 | 0.0683 | | 0.18060 | 0.045221 | 0.003525 | 0.51084 |
| AMB-00553854 | 0.2541 | 0.3971 | 0.082048 | 0.002177 | 0.57234 | 0.1211 | 0.29412 | 0.043899 | 0.002310 | 0.59969 | 0.3556 | 0.48917 | 0.131298 | 0.00307 | 0.59733 | 0.2224 | | 0.32611 | 0.088689 | 0.002177 | 0.50781 |
| est2655 | -0.0116 | 0.0523 | 0.004462 | 0.006829 | 0.56894 | 0.0327 | 0.11765 | 0.017352 | 0.007140 | 0.61228 | -0.0424 | 0.00619 | 0.000091 | 0.00689 | 0.60128 | 0.0077 | | 0.08256 | 0.017512 | 0.006829 | 0.50046 |
| AMB-00511584 | -0.0213 | 0.0474 | 0.001988 | 0.007773 | 0.56116 | 0.0233 | 0.14706 | 0.015966 | 0.008550 | 0.60147 | -0.0348 | 0.04180 | 0.001989 | 0.00483 | 0.59062 | 0.0114 | | 0.12591 | 0.021344 | 0.007773 | 0.48049 |
| AMB-01150329 | 0.1944 | 0.2222 | 0.083979 | 0.000394 | 0.58892 | 0.1289 | 0.11765 | 0.042613 | 0.000260 | 0.63353 | 0.3665 | 0.31579 | 0.124335 | 0.00038 | 0.61375 | 0.2118 | | 0.21053 | 0.084390 | 0.000394 | 0.53926 |
| AMB-00521666 | -0.0030 | 0.0997 | 0.006557 | 0.004214 | 0.55694 | 0.0594 | 0.20588 | 0.025184 | 0.010460 | 0.60092 | -0.0427 | 0.00465 | 0.000016 | 0.01017 | 0.58036 | 0.0179 | | 0.13725 | 0.022326 | 0.004214 | 0.45446 |
| est6635 | 0.0263 | 0.0556 | 0.019651 | 0.001771 | 0.58808 | -0.0500 | 0.00000 | 0.000000 | 0.001760 | 0.64400 | 0.1046 | 0.10526 | 0.037944 | 0.00170 | 0.61393 | 0.0781 | | 0.07017 | 0.039840 | 0.001771 | 0.54655 |
| est9708 | -0.0126 | 0.1026 | 0.006521 | 0.007419 | 0.55652 | -0.0216 | 0.11904 | 0.008964 | 0.007590 | 0.59671 | -0.0467 | 0.08333 | 0.004213 | 0.00606 | 0.58035 | -0.0414 | | 0.07936 | 0.006301 | 0.007419 | 0.42295 |
| AMB-00961910 | 0.0049 | 0.0605 | 0.008764 | 0.005055 | 0.57883 | 0.0250 | 0.08824 | 0.031599 | 0.002410 | 0.63199 | -0.0333 | 0.03561 | 0.002445 | 0.00849 | 0.59856 | -0.0088 | | 0.05883 | 0.021243 | 0.005055 | 0.53682 |
| AMB-00384581 | 0.0464 | 0.1095 | 0.020974 | 0.004560 | 0.57324 | -0.0342 | 0.02941 | 0.002612 | 0.008350 | 0.62113 | 0.1391 | 0.18112 | 0.043191 | 0.00274 | 0.60115 | 0.0733 | | 0.12075 | 0.033821 | 0.004560 | 0.51080 |
| *AMB-00424107 | 0.3459 | 0.4116 | 0.121004 | 0.000000 | 0.57872 | 0.1888 | 0.34375 | 0.074212 | 0.000000 | 0.61311 | 0.3879 | 0.46875 | 0.198913 | 0.00000 | 0.61569 | 0.2921 | | 0.31250 | 0.141513 | 0.000000 | 0.54103 |
| ahb6521 | -0.0016 | 0.0278 | 0.009725 | 0.001869 | 0.58960 | -0.0500 | 0.00000 | 0.000000 | 0.001690 | 0.64236 | 0.0325 | 0.05263 | 0.018596 | 0.00263 | 0.61490 | 0.0159 | | 0.03509 | 0.019589 | 0.001869 | 0.54549 |
| *ahb8292 | -0.0020 | 0.0997 | 0.006764 | 0.004296 | 0.55745 | -0.0377 | 0.05882 | 0.001978 | 0.003810 | 0.58567 | 0.1264 | 0.24149 | 0.055202 | 0.00758 | 0.60118 | 0.0741 | | 0.20021 | 0.056071 | 0.004296 | 0.52226 |
| *AMB-00039809 | 0.0542 | 0.0833 | 0.029787 | 0.000715 | 0.58869 | -0.0500 | 0.00000 | 0.000000 | 0.000440 | 0.64290 | 0.1740 | 0.15789 | 0.058110 | 0.00112 | 0.61365 | 0.1393 | | 0.10526 | 0.060805 | 0.000715 | 0.54650 |
| AMB-00034138 | -0.0500 | 0.0000 | 0.000000 | 0.000628 | 0.59876 | -0.0500 | 0.00000 | 0.000000 | 0.000550 | 0.64188 | -0.0500 | 0.00000 | 0.000000 | 0.00065 | 0.62383 | -0.0500 | | 0.00000 | 0.000000 | 0.000628 | 0.55567 |
| ahb9666 | 0.0251 | 0.0556 | 0.019651 | 0.001771 | 0.58889 | -0.0500 | 0.00000 | 0.000000 | 0.001760 | 0.64257 | 0.1018 | 0.10526 | 0.037944 | 0.00170 | 0.61258 | 0.0765 | | 0.07017 | 0.039840 | 0.001771 | 0.54603 |
| AMB-00915019 | -0.0044 | 0.0621 | 0.006218 | 0.004023 | 0.57452 | -0.0462 | 0.00000 | 0.000000 | 0.007350 | 0.60756 | 0.0557 | 0.11765 | 0.042613 | 0.00474 | 0.61221 | 0.0095 | | 0.07843 | 0.033448 | 0.004023 | 0.53841 |
| *ahb6690 | 0.2523 | 0.3268 | 0.085700 | 0.001076 | 0.57842 | 0.1160 | 0.26470 | 0.048628 | 0.001040 | 0.60997 | 0.2974 | 0.38235 | 0.155322 | 0.00095 | 0.61463 | 0.2082 | | 0.25490 | 0.108090 | 0.001076 | 0.53847 |
| *AMB-00924952 | 0.4221 | 0.5177 | 0.152395 | 0.000000 | 0.57577 | 0.5947 | 0.64350 | 0.229162 | 0.000000 | 0.62313 | 0.3131 | 0.40511 | 0.099127 | 0.00000 | 0.59621 | 0.3891 | | 0.42900 | 0.155871 | 0.000000 | 0.52365 |
| AMB-00637482 | 0.1744 | 0.2286 | 0.086611 | 0.000714 | 0.58709 | 0.0970 | 0.11765 | 0.042613 | 0.000000 | 0.63318 | 0.3428 | 0.33333 | 0.132304 | 0.00098 | 0.61313 | 0.1953 | | 0.22222 | 0.090367 | 0.000714 | 0.53913 |
| AMB-01050104 | 0.1853 | 0.2484 | 0.067127 | 0.002737 | 0.57704 | 0.3263 | 0.32353 | 0.096955 | 0.002720 | 0.62196 | 0.1391 | 0.18112 | 0.043191 | 0.00175 | 0.60005 | 0.1620 | | 0.21569 | 0.064897 | 0.002737 | 0.51192 |
| *est2046 | 0.1738 | 0.2000 | 0.074882 | 0.000597 | 0.58723 | 0.2854 | 0.23529 | 0.089413 | 0.000850 | 0.63360 | 0.1905 | 0.16667 | 0.061555 | 0.00080 | 0.61424 | 0.1335 | | 0.15686 | 0.061844 | 0.000597 | 0.53699 |
| est6628 | -0.0500 | 0.0000 | 0.000000 | 0.000699 | 0.59853 | -0.0500 | 0.00000 | 0.000000 | 0.000400 | 0.64319 | -0.0500 | 0.00000 | 0.000000 | 0.00095 | 0.62152 | -0.0500 | | 0.00000 | 0.000000 | 0.000699 | 0.55294 |
| AMB-00946259 | 0.0314 | 0.0899 | 0.016029 | 0.003890 | 0.57890 | 0.0494 | 0.11765 | 0.042613 | 0.001180 | 0.63332 | -0.0172 | 0.06502 | 0.006944 | 0.00659 | 0.60117 | 0.0109 | | 0.07843 | 0.028489 | 0.003890 | 0.53683 |
| est1831 | 0.2537 | 0.3987 | 0.081929 | 0.000183 | 0.56870 | 0.0429 | 0.20588 | 0.021836 | 0.001740 | 0.59022 | 0.4625 | 0.57121 | 0.186136 | 0.00411 | 0.60286 | 0.2869 | | 0.38081 | 0.126981 | 0.000183 | 0.52723 |
| AMB-00836604 | -0.0500 | 0.0000 | 0.000000 | 0.000262 | 0.59883 | -0.0500 | 0.00000 | 0.000000 | 0.000000 | 0.64265 | -0.0500 | 0.00000 | 0.000000 | 0.00147 | 0.62255 | -0.0500 | | 0.00000 | 0.000000 | 0.000262 | 0.55469 |
| *ahb60 | -0.0016 | 0.0278 | 0.009725 | 0.002110 | 0.59013 | -0.0500 | 0.00000 | 0.000000 | 0.001530 | 0.64299 | 0.0325 | 0.05263 | 0.018596 | 0.00113 | 0.61273 | 0.0159 | | 0.03509 | 0.019589 | 0.002110 | 0.54652 |
| AMB-00455540 | -0.0016 | 0.0278 | 0.009725 | 0.001860 | 0.58927 | -0.0500 | 0.00000 | 0.000000 | 0.001810 | 0.64215 | 0.0325 | 0.05263 | 0.018596 | 0.00179 | 0.61291 | 0.0159 | | 0.03509 | 0.019589 | 0.001860 | 0.54640 |
| AMB-00945426 | 0.0000 | 0.0294 | 0.010303 | 0.001946 | 0.58990 | -0.0278 | 0.02941 | 0.010303 | 0.001760 | 0.63355 | -0.0186 | 0.02941 | 0.010303 | 0.00302 | 0.61434 | -0.0245 | | 0.01961 | 0.010868 | 0.001946 | 0.54516 |
| *est6637 | 0.3315 | 0.3873 | 0.124785 | 0.000586 | 0.57591 | 0.3263 | 0.32353 | 0.096955 | 0.000000 | 0.62311 | 0.4568 | 0.44427 | 0.151803 | 0.00221 | 0.60320 | 0.2805 | | 0.29618 | 0.106698 | 0.000586 | 0.52184 |
| est2535 | 0.0263 | 0.0556 | 0.019651 | 0.000733 | 0.58969 | -0.0500 | 0.00000 | 0.000000 | 0.001170 | 0.64138 | 0.1046 | 0.10526 | 0.037944 | 0.00136 | 0.61354 | 0.0781 | | 0.07017 | 0.039840 | 0.000733 | 0.54601 |
| AMB-00346857 | 0.0638 | 0.0882 | 0.031599 | 0.000787 | 0.58884 | 0.0250 | 0.08824 | 0.031599 | 0.001020 | 0.63282 | 0.0306 | 0.08824 | 0.031599 | 0.00203 | 0.61408 | 0.0293 | | 0.05883 | 0.033213 | 0.000787 | 0.54531 |
| est10587 | 0.0542 | 0.0833 | 0.029787 | 0.000952 | 0.58708 | -0.0500 | 0.00000 | 0.000000 | 0.000000 | 0.64262 | 0.1740 | 0.15789 | 0.058110 | 0.00148 | 0.61252 | 0.1393 | | 0.10526 | 0.060805 | 0.000952 | 0.54619 |
| est2385 | -0.0016 | 0.0278 | 0.009725 | 0.001771 | 0.59006 | -0.0500 | 0.00000 | 0.000000 | 0.001760 | 0.64282 | 0.0325 | 0.05263 | 0.018596 | 0.00170 | 0.61206 | 0.0159 | | 0.03509 | 0.019589 | 0.001771 | 0.54647 |
| AMB-01150218 | 0.1117 | 0.2451 | 0.037420 | 0.003158 | 0.56668 | -0.0155 | 0.11764 | 0.007602 | 0.002870 | 0.59026 | 0.2408 | 0.35913 | 0.100076 | 0.00176 | 0.60295 | 0.1246 | | 0.23942 | 0.068937 | 0.003158 | 0.52084 |
| AMB-00708602 | -0.0500 | 0.0000 | 0.000000 | 0.000000 | 0.59852 | -0.0500 | 0.00000 | 0.000000 | 0.000000 | 0.64378 | -0.0500 | 0.00000 | 0.000000 | 0.00000 | 0.62338 | -0.0500 | | 0.00000 | 0.000000 | 0.000000 | 0.55552 |
| AMB-00334773 | 0.1823 | 0.2369 | 0.063708 | 0.001486 | 0.57937 | 0.1772 | 0.26471 | 0.101874 | 0.002970 | 0.63312 | 0.0996 | 0.21208 | 0.045399 | 0.00125 | 0.59742 | 0.1353 | | 0.17647 | 0.076227 | 0.001486 | 0.53785 |
| AMB-00328629 | -0.0500 | 0.0000 | 0.000000 | 0.000000 | 0.59882 | -0.0500 | 0.00000 | 0.000000 | 0.000000 | 0.64339 | -0.0500 | 0.00000 | 0.000000 | 0.00000 | 0.62297 | -0.0500 | | 0.00000 | 0.000000 | 0.000000 | 0.55408 |
| *AMB-00900481 | -0.0500 | 0.0000 | 0.000000 | 0.000000 | 0.59950 | -0.0500 | 0.00000 | 0.000000 | 0.000000 | 0.64242 | -0.0500 | 0.00000 | 0.000000 | 0.00000 | 0.62245 | -0.0500 | | 0.00000 | 0.000000 | 0.000000 | 0.55504 |
| *AMB-01062378 | -0.0016 | 0.0278 | 0.009725 | 0.000906 | 0.58845 | -0.0500 | 0.00000 | 0.000000 | 0.000860 | 0.64349 | 0.0325 | 0.05263 | 0.018596 | 0.00318 | 0.61386 | 0.0159 | | 0.03509 | 0.019589 | 0.000906 | 0.54443 |
| AMB-00855064 | -0.0500 | 0.0000 | 0.000000 | 0.000000 | 0.59973 | -0.0500 | 0.00000 | 0.000000 | 0.000000 | 0.64239 | -0.0500 | 0.00000 | 0.000000 | 0.00000 | 0.62226 | -0.0500 | | 0.00000 | 0.000000 | 0.000000 | 0.55476 |
| ahb9458 | 0.2519 | 0.3595 | 0.082787 | 0.001758 | 0.57547 | 0.2026 | 0.35294 | 0.078984 | 0.001780 | 0.61508 | 0.2252 | 0.36533 | 0.086328 | 0.00195 | 0.59828 | 0.1934 | | 0.24355 | 0.077307 | 0.001758 | 0.50589 |
| AMB-00750607 | -0.0016 | 0.0278 | 0.009725 | 0.001060 | 0.58927 | -0.0500 | 0.00000 | 0.000000 | 0.001520 | 0.64215 | 0.0325 | 0.05263 | 0.018596 | 0.00205 | 0.61321 | 0.0159 | | 0.03509 | 0.019589 | 0.001060 | 0.54594 |
| AMB-00221460 | 0.1566 | 0.2206 | 0.056916 | 0.002307 | 0.57543 | 0.0250 | 0.08824 | 0.015223 | 0.002330 | 0.62145 | 0.3345 | 0.33901 | 0.103496 | 0.00170 | 0.60207 | 0.2029 | | 0.22601 | 0.074433 | 0.002307 | 0.51901 |
| ahb4520 | 0.0211 | 0.0817 | 0.013553 | 0.002735 | 0.57463 | -0.0228 | 0.02941 | 0.010303 | 0.000200 | 0.63363 | 0.1391 | 0.18112 | 0.043191 | 0.00445 | 0.60045 | 0.1256 | | 0.14035 | 0.062937 | 0.002735 | 0.53756 |
| est1808 | 0.0314 | 0.0899 | 0.016029 | 0.002574 | 0.57800 | 0.0494 | 0.11765 | 0.042613 | 0.003010 | 0.63265 | -0.0172 | 0.06502 | 0.006944 | 0.00438 | 0.59724 | 0.0109 | | 0.07843 | 0.028489 | 0.002574 | 0.53812 |
| AMB-00415610 | -0.0500 | 0.0000 | 0.000000 | 0.000000 | 0.59694 | -0.0500 | 0.00000 | 0.000000 | 0.000000 | 0.64309 | -0.0500 | 0.00000 | 0.000000 | 0.00000 | 0.62356 | -0.0500 | | 0.00000 | 0.000000 | 0.000000 | 0.55373 |
| AMB-00925091 | -0.0500 | 0.0000 | 0.000000 | 0.000000 | 0.59764 | -0.0500 | 0.00000 | 0.000000 | 0.000000 | 0.64315 | -0.0500 | 0.00000 | 0.000000 | 0.00000 | 0.62186 | -0.0500 | | 0.00000 | 0.000000 | 0.000000 | 0.55677 |
| est8292 | -0.0016 | 0.0278 | 0.009725 | 0.000367 | 0.58986 | -0.0500 | 0.00000 | 0.000000 | 0.001090 | 0.64308 | 0.0325 | 0.05263 | 0.018596 | 0.00162 | 0.61417 | 0.0159 | | 0.03509 | 0.019589 | 0.000367 | 0.54650 |
| AMB-00718625 | -0.0500 | 0.0000 | 0.000000 | 0.000000 | 0.59863 | -0.0500 | 0.00000 | 0.000000 | 0.000000 | 0.64281 | -0.0500 | 0.00000 | 0.000000 | 0.00000 | 0.62180 | -0.0500 | | 0.00000 | 0.000000 | 0.000000 | 0.55407 |
| ahb1569 | 0.0263 | 0.0556 | 0.019651 | 0.001577 | 0.58891 | 0.0439 | 0.05882 | 0.020832 | 0.004230 | 0.63326 | 0.0325 | 0.05263 | 0.018596 | 0.00144 | 0.61357 | -0.0017 | | 0.03921 | 0.015481 | 0.001577 | 0.53740 |
| *est8515 | -0.0500 | 0.0000 | 0.000000 | 0.000000 | 0.59837 | -0.0500 | 0.00000 | 0.000000 | 0.000000 | 0.64168 | -0.0500 | 0.00000 | 0.000000 | 0.00000 | 0.62301 | -0.0500 | | 0.00000 | 0.000000 | 0.000000 | 0.55376 |
| *AMB-00715458 | -0.0500 | 0.0000 | 0.000000 | 0.000000 | 0.59758 | -0.0500 | 0.00000 | 0.000000 | 0.000000 | 0.64143 | -0.0500 | 0.00000 | 0.000000 | 0.00000 | 0.62292 | -0.0500 | | 0.00000 | 0.000000 | 0.000000 | 0.55591 |
| AMB-00748689 | 0.0542 | 0.0833 | 0.029787 | 0.000000 | 0.58770 | -0.0500 | 0.00000 | 0.000000 | 0.000000 | 0.64279 | 0.1740 | 0.15789 | 0.058110 | 0.00000 | 0.61362 | 0.1393 | | 0.10526 | 0.060805 | 0.000000 | 0.54641 |
| est6609 | 0.0250 | 0.1242 | 0.013715 | 0.003547 | 0.56568 | -0.0355 | 0.05882 | 0.002653 | 0.003780 | 0.59900 | 0.0736 | 0.18266 | 0.036199 | 0.00429 | 0.59981 | 0.0191 | | 0.12177 | 0.025015 | 0.003547 | 0.49621 |
| AMB-00919537 | -0.0500 | 0.0000 | 0.000000 | 0.000000 | 0.59891 | -0.0500 | 0.00000 | 0.000000 | 0.000000 | 0.64187 | -0.0500 | 0.00000 | 0.000000 | 0.00000 | 0.62175 | -0.0500 | | 0.00000 | 0.000000 | 0.000000 | 0.55447 |
| est10185 | 0.0263 | 0.0556 | 0.019651 | 0.000232 | 0.58893 | -0.0500 | 0.00000 | 0.000000 | 0.000000 | 0.64256 | 0.1046 | 0.10526 | 0.037944 | 0.00084 | 0.61330 | 0.0781 | | 0.07017 | 0.039840 | 0.000232 | 0.54587 |
| AMB-00489470 | -0.0500 | 0.0000 | 0.000000 | 0.000000 | 0.59729 | -0.0500 | 0.00000 | 0.000000 | 0.000000 | 0.64237 | -0.0500 | 0.00000 | 0.000000 | 0.00000 | 0.62125 | -0.0500 | | 0.00000 | 0.000000 | 0.000000 | 0.55673 |
| *est2758 | 0.0464 | 0.1095 | 0.020974 | 0.001025 | 0.57581 | -0.0228 | 0.02941 | 0.010303 | 0.000230 | 0.63298 | 0.2053 | 0.23375 | 0.061698 | 0.00529 | 0.60070 | 0.1839 | | 0.17544 | 0.083265 | 0.001025 | 0.53990 |
| ahb11198 | 0.0542 | 0.0833 | 0.029787 | 0.001047 | 0.58811 | 0.0439 | 0.05882 | 0.020832 | 0.002630 | 0.63358 | 0.1046 | 0.10526 | 0.037944 | 0.00079 | 0.61315 | 0.0319 | | 0.07017 | 0.025378 | 0.001047 | 0.53762 |
| est4660 | 0.1283 | 0.1928 | 0.047158 | 0.001989 | 0.57679 | -0.0342 | 0.02941 | 0.002612 | 0.001850 | 0.61866 | 0.3345 | 0.33901 | 0.103496 | 0.00253 | 0.60080 | 0.2402 | | 0.22601 | 0.088365 | 0.001989 | 0.53017 |
| AMB-00747219 | -0.0500 | 0.0000 | 0.000000 | 0.000000 | 0.59885 | -0.0500 | 0.00000 | 0.000000 | 0.000000 | 0.64110 | -0.0500 | 0.00000 | 0.000000 | 0.00000 | 0.62218 | -0.0500 | | 0.00000 | 0.000000 | 0.000000 | 0.55515 |
| ahb5249 | -0.0035 | 0.1127 | 0.006388 | 0.004606 | 0.54995 | -0.0388 | 0.05883 | 0.001755 | 0.001830 | 0.58303 | 0.0972 | 0.26625 | 0.037601 | 0.00416 | 0.58261 | 0.0600 | | 0.21672 | 0.041572 | 0.004606 | 0.48653 |
| ahb5207 | -0.0500 | 0.0000 | 0.000000 | 0.000000 | 0.59814 | -0.0500 | 0.00000 | 0.000000 | 0.000000 | 0.64192 | -0.0500 | 0.00000 | 0.000000 | 0.00000 | 0.62136 | -0.0500 | | 0.00000 | 0.000000 | 0.000000 | 0.55525 |
| est10536 | -0.0500 | 0.0000 | 0.000000 | 0.000000 | 0.59814 | -0.0500 | 0.00000 | 0.000000 | 0.000000 | 0.64044 | -0.0500 | 0.00000 | 0.000000 | 0.00000 | 0.62345 | -0.0500 | | 0.00000 | 0.000000 | 0.000000 | 0.55462 |
| AMB-00073185 | 0.0263 | 0.0556 | 0.019651 | 0.000368 | 0.58853 | -0.0500 | 0.00000 | 0.000000 | 0.000000 | 0.64070 | 0.1046 | 0.10526 | 0.037944 | 0.00113 | 0.61435 | 0.0781 | | 0.07017 | 0.039840 | 0.000368 | 0.54592 |
| est9274 | 0.0017 | 0.0294 | 0.010303 | 0.001254 | 0.59047 | -0.0228 | 0.02941 | 0.010303 | 0.001470 | 0.63302 | -0.0186 | 0.02941 | 0.010303 | 0.00214 | 0.61383 | -0.0221 | | 0.01961 | 0.010868 | 0.001254 | 0.54468 |
| *est9118 | -0.0500 | 0.0000 | 0.000000 | 0.000000 | 0.59833 | -0.0500 | 0.00000 | 0.000000 | 0.000000 | 0.64092 | -0.0500 | 0.00000 | 0.000000 | 0.00000 | 0.62188 | -0.0500 | | 0.00000 | 0.000000 | 0.000000 | 0.55498 |
| *ahb9204 | -0.0016 | 0.0278 | 0.009725 | 0.000503 | 0.59048 | -0.0500 | 0.00000 | 0.000000 | 0.000000 | 0.64204 | 0.0325 | 0.05263 | 0.018596 | 0.00193 | 0.61434 | 0.0159 | | 0.03509 | 0.019589 | 0.000503 | 0.54450 |
| AMB-00626770 | 0.1110 | 0.1429 | 0.052261 | 0.000000 | 0.58726 | 0.0467 | 0.06250 | 0.022165 | 0.000000 | 0.63415 | 0.2319 | 0.21053 | 0.079169 | 0.00000 | 0.61401 | 0.1292 | | 0.14035 | 0.055384 | 0.000000 | 0.53847 |
| AMB-00561565 | 0.0542 | 0.0833 | 0.029787 | 0.000897 | 0.58817 | 0.1289 | 0.11765 | 0.042613 | 0.001670 | 0.63305 | 0.0325 | 0.05263 | 0.018596 | 0.00064 | 0.61415 | 0.0420 | | 0.07843 | 0.028489 | 0.000897 | 0.53764 |
| AMB-00549407 | -0.0121 | 0.0294 | 0.010303 | 0.000712 | 0.58865 | -0.0500 | 0.00000 | 0.000000 | 0.000740 | 0.64181 | 0.0184 | 0.05882 | 0.020832 | 0.00103 | 0.61282 | 0.0097 | | 0.03921 | 0.021936 | 0.000712 | 0.54524 |
| est11459 | -0.0016 | 0.0278 | 0.009725 | 0.000119 | 0.58945 | -0.0500 | 0.00000 | 0.000000 | 0.000000 | 0.64143 | 0.0325 | 0.05263 | 0.018596 | 0.00189 | 0.61375 | 0.0159 | | 0.03509 | 0.019589 | 0.000119 | 0.54553 |
| est10432 | -0.0016 | 0.0278 | 0.009725 | 0.000050 | 0.58888 | -0.0500 | 0.00000 | 0.000000 | 0.000000 | 0.64346 | 0.0325 | 0.05263 | 0.018596 | 0.00040 | 0.61437 | 0.0159 | | 0.03509 | 0.019589 | 0.000050 | 0.54593 |
| est11596 | -0.0016 | 0.0278 | 0.009725 | 0.000215 | 0.58938 | -0.0500 | 0.00000 | 0.000000 | 0.000000 | 0.64336 | 0.0325 | 0.05263 | 0.018596 | 0.00045 | 0.61404 | 0.0159 | | 0.03509 | 0.019589 | 0.000215 | 0.54512 |
| est10329 | -0.0016 | 0.0278 | 0.009725 | 0.000175 | 0.58983 | -0.0500 | 0.00000 | 0.000000 | 0.000000 | 0.64194 | 0.0325 | 0.05263 | 0.018596 | 0.00103 | 0.61358 | 0.0159 | | 0.03509 | 0.019589 | 0.000175 | 0.54558 |
| est4488 | 0.0120 | 0.1275 | 0.010386 | 0.002889 | 0.55297 | -0.0435 | 0.02941 | 0.000629 | 0.003140 | 0.59340 | 0.0696 | 0.21517 | 0.027307 | 0.00406 | 0.58389 | 0.0222 | | 0.14345 | 0.021591 | 0.002889 | 0.44776 |
| AMB-00139758 | 0.0729 | 0.1373 | 0.029127 | 0.002451 | 0.57449 | 0.0990 | 0.14706 | 0.032152 | 0.002560 | 0.61762 | 0.0735 | 0.12848 | 0.026483 | 0.00225 | 0.60047 | 0.0377 | | 0.09804 | 0.024475 | 0.002451 | 0.49105 |
| ahb10230 | -0.0209 | 0.0262 | 0.002133 | 0.005015 | 0.57882 | -0.0342 | 0.02941 | 0.002612 | 0.005520 | 0.62048 | -0.0350 | 0.02322 | 0.001736 | 0.00175 | 0.60212 | -0.0403 | | 0.01961 | 0.001918 | 0.005015 | 0.51132 |
| *ahb11101 | -0.0016 | 0.0278 | 0.009725 | 0.000140 | 0.58988 | -0.0500 | 0.00000 | 0.000000 | 0.000000 | 0.64235 | 0.0325 | 0.05263 | 0.018596 | 0.00023 | 0.61278 | 0.0159 | | 0.03509 | 0.019589 | 0.000140 | 0.54681 |
| est2417 | -0.0016 | 0.0278 | 0.009725 | 0.000175 | 0.58993 | -0.0500 | 0.00000 | 0.000000 | 0.000000 | 0.64188 | 0.0325 | 0.05263 | 0.018596 | 0.00103 | 0.61329 | 0.0159 | | 0.03509 | 0.019589 | 0.000175 | 0.54546 |
| ahb6886 | -0.0212 | 0.0327 | 0.002017 | 0.003637 | 0.57610 | -0.0399 | 0.02942 | 0.001597 | 0.001780 | 0.61762 | -0.0333 | 0.03561 | 0.002445 | 0.00952 | 0.59874 | -0.0406 | | 0.02374 | 0.001876 | 0.003637 | 0.50106 |
| est10120 | -0.0016 | 0.0278 | 0.009725 | 0.000219 | 0.58837 | -0.0500 | 0.00000 | 0.000000 | 0.000000 | 0.64318 | 0.0325 | 0.05263 | 0.018596 | 0.00154 | 0.61332 | 0.0159 | | 0.03509 | 0.019589 | 0.000219 | 0.54452 |
| ahb2123 | 0.0897 | 0.1487 | 0.033261 | 0.001436 | 0.57833 | 0.0993 | 0.17647 | 0.065436 | 0.002250 | 0.63211 | 0.0248 | 0.12384 | 0.019830 | 0.00143 | 0.59709 | 0.0572 | | 0.11765 | 0.045743 | 0.001436 | 0.53610 |
| AMB-00179887 | -0.0020 | 0.0539 | 0.007104 | 0.001284 | 0.57751 | -0.0228 | 0.02941 | 0.010303 | 0.000200 | 0.63291 | 0.0735 | 0.12848 | 0.026483 | 0.00611 | 0.60171 | 0.0687 | | 0.10526 | 0.043999 | 0.001284 | 0.53824 |
| AMB-01102712 | -0.0016 | 0.0278 | 0.009725 | 0.000000 | 0.58940 | -0.0500 | 0.00000 | 0.000000 | 0.000000 | 0.64370 | 0.0325 | 0.05263 | 0.018596 | 0.00000 | 0.61357 | 0.0159 | | 0.03509 | 0.019589 | 0.000000 | 0.54513 |
| AMB-00917839 | -0.0026 | 0.0286 | 0.010006 | 0.000144 | 0.58983 | -0.0500 | 0.00000 | 0.000000 | 0.000000 | 0.64112 | 0.0288 | 0.05263 | 0.018596 | 0.00023 | 0.61320 | 0.0127 | | 0.03509 | 0.019589 | 0.000144 | 0.54535 |
| ahb1971 | 0.0017 | 0.0294 | 0.010303 | 0.000443 | 0.59009 | -0.0228 | 0.02941 | 0.010303 | 0.000440 | 0.63315 | -0.0186 | 0.02941 | 0.010303 | 0.00043 | 0.61320 | -0.0221 | | 0.01961 | 0.010868 | 0.000443 | 0.54553 |
| AMB-00341665 | -0.0294 | 0.0016 | 0.000012 | 0.002464 | 0.58142 | -0.0228 | 0.02941 | 0.010303 | 0.000670 | 0.63393 | -0.0350 | 0.02322 | 0.001736 | 0.00503 | 0.60320 | -0.0262 | | 0.03509 | 0.012437 | 0.002464 | 0.53614 |
| AMB-00851094 | -0.0292 | 0.0065 | 0.000053 | 0.004043 | 0.56737 | -0.0309 | 0.05882 | 0.003468 | 0.004200 | 0.60437 | -0.0172 | 0.06502 | 0.006944 | 0.00220 | 0.59717 | -0.0153 | | 0.08256 | 0.013231 | 0.004043 | 0.49084 |
| ahb6442 | 0.1478 | 0.2941 | 0.048450 | 0.000854 | 0.56316 | 0.0119 | 0.14706 | 0.013548 | 0.001040 | 0.59556 | 0.2968 | 0.42570 | 0.096599 | 0.00099 | 0.59396 | 0.1641 | | 0.28380 | 0.066424 | 0.000854 | 0.49789 |
| AMB-00619176 | 0.1066 | 0.1706 | 0.039696 | 0.001534 | 0.57566 | 0.2808 | 0.28309 | 0.080510 | 0.001000 | 0.62079 | 0.0120 | 0.07585 | 0.012103 | 0.00125 | 0.59892 | 0.1445 | | 0.18873 | 0.057648 | 0.001534 | 0.51435 |
| est424 | 0.0348 | 0.1569 | 0.016400 | 0.001782 | 0.55940 | -0.0041 | 0.11765 | 0.009696 | 0.000000 | 0.59816 | 0.0560 | 0.19195 | 0.023622 | 0.00771 | 0.58425 | 0.0110 | | 0.12797 | 0.016006 | 0.001782 | 0.42812 |
| ahb3528 | 0.0009 | 0.0588 | 0.020832 | 0.000168 | 0.58784 | 0.0086 | 0.06667 | 0.023680 | 0.000710 | 0.63348 | -0.0061 | 0.05263 | 0.018596 | 0.00063 | 0.61284 | -0.0291 | | 0.04445 | 0.016826 | 0.000168 | 0.53779 |
| est2830 | 0.1566 | 0.2206 | 0.056916 | 0.000000 | 0.57385 | -0.0342 | 0.02941 | 0.002612 | 0.000000 | 0.62404 | 0.3966 | 0.39164 | 0.126790 | 0.00000 | 0.60298 | 0.2980 | | 0.26109 | 0.109830 | 0.000000 | 0.53206 |
| AMB-00983304 | -0.0293 | 0.0033 | 0.000025 | 0.002315 | 0.57323 | 0.0009 | 0.05882 | 0.020832 | 0.001760 | 0.63323 | -0.0267 | 0.04644 | 0.003625 | 0.00247 | 0.59509 | -0.0041 | | 0.07017 | 0.025378 | 0.002315 | 0.53602 |
| AMB-00944389 | 0.0897 | 0.1487 | 0.033261 | 0.000085 | 0.57934 | 0.0993 | 0.17647 | 0.065436 | 0.000190 | 0.63294 | 0.0248 | 0.12384 | 0.019830 | 0.00020 | 0.59872 | 0.0572 | | 0.11765 | 0.045743 | 0.000085 | 0.53697 |
| *ahb3792 | 0.0292 | 0.0891 | 0.015633 | 0.000698 | 0.57751 | 0.0459 | 0.11765 | 0.042613 | 0.000430 | 0.63064 | -0.0172 | 0.06502 | 0.006944 | 0.00284 | 0.59851 | 0.0085 | | 0.07843 | 0.028489 | 0.000698 | 0.53640 |
| ahb763 | 0.1667 | 0.2974 | 0.054954 | 0.000000 | 0.56643 | 0.1384 | 0.26470 | 0.044863 | 0.000000 | 0.60856 | 0.2071 | 0.32662 | 0.064730 | 0.00000 | 0.59305 | 0.1208 | | 0.21775 | 0.048153 | 0.000000 | 0.46606 |
| est6322 | 0.0169 | 0.1275 | 0.011570 | 0.001708 | 0.56175 | -0.0371 | 0.05883 | 0.002226 | 0.001660 | 0.59305 | 0.0582 | 0.18886 | 0.028826 | 0.00178 | 0.58851 | 0.0101 | | 0.12591 | 0.020051 | 0.001708 | 0.47407 |
| est2267 | -0.0044 | 0.0621 | 0.006218 | 0.000853 | 0.57594 | 0.0494 | 0.11765 | 0.042613 | 0.000800 | 0.63437 | -0.0420 | 0.01239 | 0.000194 | 0.00096 | 0.59394 | 0.0023 | | 0.07843 | 0.031729 | 0.000853 | 0.53512 |
| *AMB-00961951 | -0.0294 | 0.0016 | 0.000012 | 0.001081 | 0.57957 | -0.0228 | 0.02941 | 0.010303 | 0.001150 | 0.63370 | -0.0350 | 0.02322 | 0.001736 | 0.00130 | 0.60226 | -0.0262 | | 0.03509 | 0.012437 | 0.001081 | 0.53867 |
| AMB-00894580 | -0.0209 | 0.0262 | 0.002133 | 0.000509 | 0.57703 | -0.0228 | 0.02941 | 0.010303 | 0.000190 | 0.63389 | 0.0120 | 0.07585 | 0.012103 | 0.00148 | 0.59951 | 0.0157 | | 0.07017 | 0.026794 | 0.000509 | 0.53670 |
| ahb5121 | -0.0293 | 0.0033 | 0.000025 | 0.000504 | 0.57470 | 0.0009 | 0.05882 | 0.020832 | 0.000390 | 0.63278 | -0.0267 | 0.04644 | 0.003625 | 0.00215 | 0.59186 | -0.0041 | | 0.07017 | 0.025378 | 0.000504 | 0.53698 |
| *ahb5906 | -0.0294 | 0.0016 | 0.000012 | 0.000962 | 0.58019 | -0.0228 | 0.02941 | 0.010303 | 0.001180 | 0.63194 | -0.0350 | 0.02322 | 0.001736 | 0.00068 | 0.60207 | -0.0262 | | 0.03509 | 0.012437 | 0.000962 | 0.53815 |
| AMB-00643734 | 0.0464 | 0.1095 | 0.020974 | 0.000013 | 0.57382 | -0.0342 | 0.02941 | 0.002612 | 0.000000 | 0.62168 | 0.1391 | 0.18112 | 0.043191 | 0.00010 | 0.60152 | 0.0733 | | 0.12075 | 0.033821 | 0.000013 | 0.51285 |
| AMB-00372961 | -0.0293 | 0.0033 | 0.000025 | 0.000239 | 0.57395 | 0.0009 | 0.05882 | 0.020832 | 0.000060 | 0.63354 | -0.0267 | 0.04644 | 0.003625 | 0.00070 | 0.59486 | -0.0041 | | 0.07017 | 0.025378 | 0.000239 | 0.53683 |
| AMB-00342757 | -0.0294 | 0.0016 | 0.000012 | 0.000134 | 0.58210 | -0.0228 | 0.02941 | 0.010303 | 0.000200 | 0.63313 | -0.0350 | 0.02322 | 0.001736 | 0.00046 | 0.60199 | -0.0262 | | 0.03509 | 0.012437 | 0.000134 | 0.53665 |
| ahb6743 | -0.0010 | 0.1177 | 0.007035 | 0.000483 | 0.55007 | -0.0294 | 0.08824 | 0.003985 | 0.000710 | 0.58429 | 0.0004 | 0.14397 | 0.010488 | 0.00177 | 0.57207 | -0.0216 | | 0.09598 | 0.007110 | 0.000483 | 0.39190 |
| *AMB-00750536 | -0.0241 | 0.0490 | 0.001308 | 0.000159 | 0.54955 | 0.0519 | 0.20588 | 0.026795 | 0.000000 | 0.60370 | -0.0250 | 0.09133 | 0.004266 | 0.00066 | 0.57066 | 0.0364 | | 0.19814 | 0.036107 | 0.000159 | 0.48136 |
