# Supplementary material for: Reduced SNP Panels for Genetic Identification and Introgression Analysis in the Dark Honey Bee (Apis mellifera mellifera)
Source: PLoS One. 2015 Apr 13;10(4):e0124365. doi: 10.1371/journal.pone.0124365 (PMC4395157; doi:10.1371/journal.pone.0124365)
Supplement: S3 Table — The holdout set consisted of 34 pure (training set) and 43 reserved individuals of A. m. mellifera and all reference individuals of A. m. ligustica (17) and A. m. carnica (19). * Samples marked with an asterisk (*) are of A. m. mellifera from protected populations (pure breeding for conservation purposes; see Pinto et al. 2014 [9] for details). (DOCX) [file pone.0124365.s005.docx]

**S3 Table.** **Admixture proportion estimates inferred from the five AIMs panels (48-, 96-, 144-, 192-, 384-AIMs) and the initial 1183 SNP dataset for the holdout set.**

| Sample ID | Population | Longitude | Latitude | 48-AIMs | 96-AIMs | 144-AIMs | 192-AIMs | 384-AIMs | 1183 SNPs |
| --- | --- | --- | --- | --- | --- | --- | --- | --- | --- |
| 1 | France | -0.6794 | 43.4567 | 0.68330 | 0.68279 | 0.67407 | 0.66795 | 0.61564 | 0.69263 |
| 2 | France | -0.6862 | 43.4549 | 0.65983 | 0.67150 | 0.63386 | 0.64437 | 0.61159 | 0.67833 |
| 3 | France | -0.6797 | 43.4535 | 0.57505 | 0.54099 | 0.53302 | 0.53554 | 0.47021 | 0.57083 |
| 4 | France | -0.6819 | 43.4513 | 0.31247 | 0.27276 | 0.28068 | 0.25265 | 0.21850 | 0.25307 |
| 5* | France | -0.6850 | 43.4534 | 0.00001 | 0.00001 | 0.00001 | 0.00001 | 0.00001 | 0.00001 |
| 6 | France | 6.5308 | 45.5917 | 0.11210 | 0.07297 | 0.07445 | 0.06061 | 0.06591 | 0.05526 |
| 7* | France | 3.6742 | 44.3617 | 0.00001 | 0.00001 | 0.00001 | 0.00001 | 0.01995 | 0.03181 |
| 8 | France | 1.9723 | 48.6013 | 0.28271 | 0.34799 | 0.33570 | 0.33834 | 0.28744 | 0.32502 |
| 9 | France | 1.9660 | 48.5963 | 0.10251 | 0.17782 | 0.16712 | 0.15281 | 0.14394 | 0.13843 |
| 10 | France | 6.5308 | 45.5917 | 0.33974 | 0.35540 | 0.33639 | 0.34093 | 0.29775 | 0.32026 |
| 11* | France | 1.9723 | 48.6013 | 0.00001 | 0.00001 | 0.00001 | 0.00001 | 0.00001 | 0.03043 |
| 12 | France | -0.9185 | 46.5684 | 0.08118 | 0.09883 | 0.08011 | 0.07747 | 0.06305 | 0.06985 |
| 13 | France | 6.5308 | 45.5917 | 0.08725 | 0.05661 | 0.05796 | 0.05642 | 0.04965 | 0.07876 |
| 14* | France | 1.9723 | 48.6013 | 0.04057 | 0.02673 | 0.04445 | 0.03514 | 0.00082 | 0.03231 |
| 15 | France | 1.9723 | 48.6013 | 0.06465 | 0.00001 | 0.03264 | 0.03507 | 0.02421 | 0.03904 |
| 16 | Belgium | 4.3384 | 50.0674 | 0.02205 | 0.02025 | 0.01435 | 0.02256 | 0.03930 | 0.01165 |
| 17 | Belgium | 4.3384 | 50.0674 | 0.02996 | 0.01958 | 0.03487 | 0.06555 | 0.06262 | 0.03709 |
| 18 | Belgium | 4.3384 | 50.0674 | 0.07282 | 0.10738 | 0.10352 | 0.10466 | 0.09589 | 0.08794 |
| 19 | Denmark | 11.1797 | 57.3169 | 0.13368 | 0.21721 | 0.19851 | 0.21021 | 0.21814 | 0.21240 |
| 20 | Denmark | 11.1797 | 57.3169 | 0.39671 | 0.34379 | 0.30983 | 0.31028 | 0.27970 | 0.24999 |
| 21 | Denmark | 11.1797 | 57.3169 | 0.14873 | 0.14328 | 0.15877 | 0.13336 | 0.10064 | 0.08275 |
| 22 | Denmark | 11.1797 | 57.3169 | 0.11696 | 0.10343 | 0.11069 | 0.10503 | 0.09820 | 0.07790 |
| 23 | Denmark | 11.1797 | 57.3169 | 0.04975 | 0.06611 | 0.07327 | 0.08030 | 0.11277 | 0.12574 |
| 24 | Denmark | 11.1797 | 57.3169 | 0.00001 | 0.00001 | 0.02641 | 0.05464 | 0.05307 | 0.05135 |
| 25 | Denmark | 11.1797 | 57.3169 | 0.11277 | 0.11827 | 0.10575 | 0.13531 | 0.13011 | 0.13221 |
| 26 | Denmark | 11.1797 | 57.3169 | 0.07014 | 0.10486 | 0.11860 | 0.12606 | 0.10176 | 0.09162 |
| 27* | Denmark | 11.1797 | 57.3169 | 0.00001 | 0.00001 | 0.00001 | 0.00001 | 0.01731 | 0.03286 |
| 28 | Denmark | 11.1797 | 57.3169 | 0.15084 | 0.17391 | 0.18308 | 0.20247 | 0.18793 | 0.19385 |
| 29* | Holland | 4.7809 | 53.1044 | 0.00001 | 0.00001 | 0.00001 | 0.00001 | 0.00001 | 0.00001 |
| 30* | Holland | 4.8067 | 53.0530 | 0.00001 | 0.00001 | 0.00001 | 0.00001 | 0.00001 | 0.00001 |
| 31* | Holland | 4.7809 | 53.1044 | 0.00001 | 0.00001 | 0.00001 | 0.00001 | 0.00001 | 0.00001 |
| 32* | Holland | 4.8067 | 53.0530 | 0.00001 | 0.00001 | 0.00001 | 0.00001 | 0.00001 | 0.00001 |
| 33 | Holland | 4.7476 | 53.0669 | 0.00001 | 0.00001 | 0.00001 | 0.00001 | 0.00001 | 0.00001 |
| 34* | Holland | 4.7809 | 53.1044 | 0.00001 | 0.00001 | 0.00001 | 0.00001 | 0.00001 | 0.00001 |
| 35 | Holland | 4.7476 | 53.0669 | 0.08216 | 0.07677 | 0.06640 | 0.07484 | 0.07859 | 0.07783 |
| 36* | Holland | 4.7809 | 53.1044 | 0.00001 | 0.00001 | 0.00001 | 0.00001 | 0.00001 | 0.00001 |
| 37* | Holland | 4.7809 | 53.1044 | 0.00001 | 0.00001 | 0.00001 | 0.00001 | 0.00001 | 0.00001 |
| 38* | Holland | 4.8067 | 53.0530 | 0.02202 | 0.00001 | 0.00001 | 0.01714 | 0.00001 | 0.00001 |
| 39* | Holland | 4.8067 | 53.0530 | 0.00001 | 0.00001 | 0.00001 | 0.00001 | 0.00001 | 0.00001 |
| 40 | Holland | 4.7809 | 53.1044 | 0.08022 | 0.05766 | 0.04148 | 0.04391 | 0.04602 | 0.05637 |
| 41* | Holland | 4.7809 | 53.1044 | 0.00001 | 0.00001 | 0.00001 | 0.00001 | 0.00001 | 0.00001 |
| 42 | Holland | 4.7809 | 53.1044 | 0.61354 | 0.58683 | 0.56384 | 0.57916 | 0.57120 | 0.58676 |
| 43* | Holland | 4.8067 | 53.0530 | 0.00001 | 0.00001 | 0.00001 | 0.00001 | 0.00001 | 0.00001 |
| 44 | Switzerland | 8.2972 | 47.0498 | 0.12068 | 0.09401 | 0.06385 | 0.06467 | 0.07569 | 0.06308 |
| 45 | Switzerland | 8.2972 | 47.0498 | 0.07619 | 0.15526 | 0.12105 | 0.10486 | 0.09404 | 0.10190 |
| 46 | Switzerland | 8.2972 | 47.0498 | 0.17583 | 0.10920 | 0.14671 | 0.13951 | 0.11396 | 0.11593 |
| 47 | Switzerland | 8.2972 | 47.0498 | 0.07647 | 0.08318 | 0.09199 | 0.08401 | 0.08421 | 0.09494 |
| 48 | Switzerland | 8.2972 | 47.0498 | 0.22711 | 0.18371 | 0.19587 | 0.19992 | 0.18110 | 0.16570 |
| 49 | Switzerland | 8.2972 | 47.0498 | 0.09958 | 0.06222 | 0.09267 | 0.08608 | 0.08961 | 0.08734 |
| 50* | Scotland | -6.2315 | 56.0795 | 0.00001 | 0.00001 | 0.00001 | 0.00001 | 0.00001 | 0.00001 |
| 51 | Scotland | -6.2315 | 56.0795 | 0.04414 | 0.01933 | 0.03215 | 0.02171 | 0.02530 | 0.02350 |
| 52* | Scotland | -6.2315 | 56.0795 | 0.00001 | 0.00001 | 0.00001 | 0.00001 | 0.00001 | 0.00001 |
| 53* | Scotland | -6.2315 | 56.0795 | 0.03084 | 0.00001 | 0.00001 | 0.00001 | 0.00001 | 0.00001 |
| 54* | Scotland | -6.1986 | 56.0696 | 0.00001 | 0.00001 | 0.00001 | 0.00001 | 0.00042 | 0.03117 |
| 55* | Scotland | -6.1986 | 56.0696 | 0.00001 | 0.00001 | 0.00001 | 0.00001 | 0.00001 | 0.00001 |
| 56* | Scotland | -6.1986 | 56.0696 | 0.00001 | 0.00001 | 0.00001 | 0.00001 | 0.00001 | 0.00001 |
| 57* | Scotland | -6.1986 | 56.0696 | 0.00001 | 0.00001 | 0.00001 | 0.00001 | 0.00001 | 0.00001 |
| 58* | Scotland | -6.1986 | 56.0696 | 0.00001 | 0.00001 | 0.00001 | 0.00001 | 0.00001 | 0.01523 |
| 59* | Scotland | -6.1986 | 56.0696 | 0.02161 | 0.00001 | 0.00001 | 0.00001 | 0.00001 | 0.00001 |
| 60* | Norway | 6.6659 | 58.2740 | 0.00001 | 0.00001 | 0.00001 | 0.00001 | 0.00001 | 0.00001 |
| 61* | Norway | 5.9587 | 58.5875 | 0.00001 | 0.00001 | 0.00001 | 0.00001 | 0.00001 | 0.00001 |
| 62 | Norway | 6.1609 | 58.6330 | 0.00001 | 0.00001 | 0.00001 | 0.00001 | 0.00001 | 0.00001 |
| 63* | Norway | 6.1342 | 58.6398 | 0.00001 | 0.00001 | 0.00001 | 0.00001 | 0.00001 | 0.00001 |
| 64* | Norway | 6.6696 | 58.2939 | 0.00001 | 0.00001 | 0.00001 | 0.00001 | 0.00001 | 0.00001 |
| 65* | Norway | 6.2871 | 58.3440 | 0.00001 | 0.00001 | 0.00001 | 0.00001 | 0.00001 | 0.00001 |
| 66* | Norway | 6.5697 | 58.2855 | 0.00001 | 0.00001 | 0.00001 | 0.00001 | 0.00001 | 0.00001 |
| 67* | Norway | 6.2871 | 58.3440 | 0.00001 | 0.00001 | 0.00001 | 0.00001 | 0.00001 | 0.00001 |
| 68* | Norway | 6.2864 | 58.3246 | 0.00001 | 0.00001 | 0.00001 | 0.00001 | 0.00001 | 0.00001 |
| 69* | Norway | 6.2864 | 58.3246 | 0.00001 | 0.00001 | 0.00001 | 0.00001 | 0.00001 | 0.00001 |
| 70 | England | -0.0872 | 50.8635 | 0.15869 | 0.14829 | 0.14749 | 0.13327 | 0.13595 | 0.12251 |
| 71 | England | -0.0872 | 50.8635 | 0.21430 | 0.25976 | 0.23490 | 0.24134 | 0.23067 | 0.23290 |
| 72 | England | -0.0872 | 50.8635 | 0.18641 | 0.23172 | 0.20144 | 0.17916 | 0.17380 | 0.18593 |
| 73 | England | -0.0873 | 50.8635 | 0.10971 | 0.08342 | 0.11361 | 0.12490 | 0.12227 | 0.14150 |
| 74 | England | -0.0873 | 50.8635 | 0.14556 | 0.08532 | 0.10464 | 0.09595 | 0.10036 | 0.10688 |
| 75 | England | -0.0873 | 50.8635 | 0.41894 | 0.35556 | 0.34925 | 0.34466 | 0.35462 | 0.33472 |
| 76 | England | -0.0873 | 50.8635 | 0.26612 | 0.22654 | 0.19681 | 0.19038 | 0.17959 | 0.16204 |
| 77 | England | -0.0873 | 50.8635 | 0.27590 | 0.18815 | 0.17461 | 0.21017 | 0.23537 | 0.23604 |
| 78 | Italy | 11.4006 | 44.4806 | 0.99999 | 0.99999 | 0.99999 | 0.99999 | 0.99999 | 0.99999 |
| 79 | Italy | 11.4007 | 44.4806 | 0.99999 | 0.99999 | 0.99999 | 0.99999 | 0.99999 | 0.99999 |
| 80 | Italy | 11.4008 | 44.4806 | 0.99999 | 0.99999 | 0.99999 | 0.99999 | 0.99999 | 0.99999 |
| 81 | Italy | 11.4009 | 44.4806 | 0.99999 | 0.99999 | 0.99999 | 0.99999 | 0.99999 | 0.99999 |
| 82 | Italy | 11.4010 | 44.4806 | 0.99999 | 0.96865 | 0.99999 | 0.96542 | 0.96759 | 0.96307 |
| 83 | Italy | 11.5271 | 44.7303 | 0.96816 | 0.99999 | 0.99999 | 0.99999 | 0.98714 | 0.99999 |
| 84 | Italy | 11.5271 | 44.7303 | 0.99999 | 0.99999 | 0.97238 | 0.96858 | 0.96777 | 0.97538 |
| 85 | Italy | 11.5271 | 44.7303 | 0.94548 | 0.99999 | 0.99999 | 0.99999 | 0.99999 | 0.99999 |
| 86 | Italy | 11.5272 | 44.7303 | 0.98147 | 0.99999 | 0.96882 | 0.97516 | 0.99999 | 0.99861 |
| 87 | Italy | 11.5272 | 44.7303 | 0.99999 | 0.99999 | 0.99999 | 0.99780 | 0.99658 | 0.99999 |
| 88 | Italy | 10.5865 | 44.6731 | 0.99999 | 0.99999 | 0.99999 | 0.99999 | 0.99999 | 0.99999 |
| 89 | Italy | 10.5865 | 44.6731 | 0.99999 | 0.99999 | 0.99999 | 0.99999 | 0.99999 | 0.99999 |
| 90 | Italy | 10.5865 | 44.6731 | 0.99999 | 0.99999 | 0.99999 | 0.99999 | 0.99999 | 0.99999 |
| 91 | Italy | 10.5865 | 44.6731 | 0.97018 | 0.97697 | 0.97932 | 0.96526 | 0.98054 | 0.99853 |
| 92 | Italy | 10.5865 | 44.6731 | 0.99999 | 0.99999 | 0.99999 | 0.99999 | 0.99999 | 0.99999 |
| 93 | Italy | 10.5865 | 44.6731 | 0.99999 | 0.99999 | 0.99999 | 0.99999 | 0.99999 | 0.99999 |
| 94 | Italy | 10.5865 | 44.6731 | 0.99999 | 0.99999 | 0.99999 | 0.99999 | 0.99999 | 0.99999 |
| 95 | Serbia | 15.7167 | 45.8000 | 0.99999 | 0.99999 | 0.99999 | 0.99999 | 0.99999 | 0.99999 |
| 96 | Serbia | 15.9800 | 45.8300 | 0.99999 | 0.99999 | 0.99999 | 0.99999 | 0.99999 | 0.99999 |
| 97 | Serbia | 16.2333 | 45.8000 | 0.99999 | 0.99999 | 0.99999 | 0.99999 | 0.99999 | 0.99999 |
| 98 | Serbia | 15.9800 | 45.8300 | 0.99999 | 0.99999 | 0.99999 | 0.99999 | 0.99999 | 0.99999 |
| 99 | Serbia | 15.9800 | 45.8300 | 0.99999 | 0.99999 | 0.99999 | 0.99999 | 0.99705 | 0.99999 |
| 100 | Serbia | 15.5833 | 45.1000 | 0.99999 | 0.99999 | 0.99999 | 0.99999 | 0.99999 | 0.99999 |
| 101 | Serbia | 15.9800 | 45.8300 | 0.99999 | 0.99999 | 0.99999 | 0.99999 | 0.98817 | 0.99999 |
| 102 | Serbia | 16.1100 | 45.8300 | 0.99999 | 0.99999 | 0.99999 | 0.97050 | 0.99999 | 0.99999 |
| 103 | Croatia | 16.1200 | 45.8300 | 0.99999 | 0.99999 | 0.99999 | 0.99999 | 0.99999 | 0.99999 |
| 104 | Croatia | 15.9800 | 45.8300 | 0.99999 | 0.99999 | 0.99999 | 0.99999 | 0.99999 | 0.99999 |
| 105 | Croatia | 15.9800 | 45.8300 | 0.99999 | 0.99999 | 0.99999 | 0.99999 | 0.99999 | 0.99999 |
| 106 | Croatia | 19.8667 | 45.2500 | 0.96456 | 0.99232 | 0.99999 | 0.99999 | 0.99999 | 0.99999 |
| 107 | Croatia | 19.8000 | 45.2000 | 0.99999 | 0.99999 | 0.99999 | 0.99999 | 0.99999 | 0.99999 |
| 108 | Croatia | 19.4000 | 45.1167 | 0.99999 | 0.99999 | 0.99999 | 0.99999 | 0.99999 | 0.99999 |
| 109 | Croatia | 20.0167 | 45.2167 | 0.99999 | 0.99999 | 0.99999 | 0.99999 | 0.99999 | 0.99999 |
| 110 | Croatia | 20.0333 | 45.6167 | 0.99999 | 0.99999 | 0.99999 | 0.99999 | 0.99999 | 0.99999 |
| 111 | Croatia | 19.1000 | 45.7667 | 0.99999 | 0.99999 | 0.99999 | 0.99999 | 0.99999 | 0.99999 |
| 112 | Croatia | 19.6667 | 46.1000 | 0.99999 | 0.99999 | 0.99999 | 0.99999 | 0.99999 | 0.99999 |
| 113 | Croatia | 19.6667 | 46.1000 | 0.99999 | 0.99999 | 0.99999 | 0.99999 | 0.99999 | 0.99999 |
