# Supplementary material for: Reduced SNP Panels for Genetic Identification and Introgression Analysis in the Dark Honey Bee (Apis mellifera mellifera)
Source: PLoS One. 2015 Apr 13;10(4):e0124365. doi: 10.1371/journal.pone.0124365 (PMC4395157; doi:10.1371/journal.pone.0124365)
Supplement: S4 Table — Values obtained from comparing individual admixture proportions estimated with the five AIMs panels (48-, 96-, 144-, 192-, 384-AIMs) and the initial 1183 SNP dataset using the holdout set. (DOCX) [file pone.0124365.s006.docx]

**S4 Table.** ***P*-values of Mann-Whitney pairwise several-sample-test.**

| Panel | 48-AIMs | 96-AIMs | 144-AIMs | 192-AIMs | 384-AIMs |
| --- | --- | --- | --- | --- | --- |
| 96-AIMs | 0.8837 |  |  |  |  |
| 144-AIMs | 0.9734 | 0.9199 |  |  |  |
| 192-AIMs | 0.9154 | 0.9983 | 0.9634 |  |  |
| 384-AIMs | 0.9065 | 0.9842 | 0.9327 | 0.9950 |  |
| 1183 SNPs | 0.9221 | 0.8200 | 0.9054 | 0.8450 | 0.8225 |
