# Supplementary material for: Reduced SNP Panels for Genetic Identification and Introgression Analysis in the Dark Honey Bee (Apis mellifera mellifera)
Source: PLoS One. 2015 Apr 13;10(4):e0124365. doi: 10.1371/journal.pone.0124365 (PMC4395157; doi:10.1371/journal.pone.0124365)
Supplement: S5 Table — Values obtained from comparing admixture proportions inferred from the five AIMs panels and the 1183 initial SNP dataset using the simulated set. The simulated set was generated with the program ONCOR (Kalinowski et al. 2007) using the function “simulate a single mixture”. Ten populations, each with 100 genotypes, were simulated using different levels of C-lineage introgression (0, 1, 5, 10, 20, 30, 40, 50, 75, and 90%). (DOCX) [file pone.0124365.s007.docx]

**S5 Table**. ***P*-values of Mann-Whitney pairwise several-sample-test.**

| Panel | 48-AIMs | 96-AIMs | 144-AIMs | 192-AIMs | 384-AIMs |
| --- | --- | --- | --- | --- | --- |
| 96-AIMs | 0.6580 |  |  |  |  |
| 144-AIMs | 0.8284 | 0.8207 |  |  |  |
| 192-AIMs | 0.9120 | 0.7392 | 0.9165 |  |  |
| 384-AIMs | 0.9742 | 0.6847 | 0.8625 | 0.9485 |  |
| 1183 SNPs | 0.4588 | 0.2313 | 0.3355 | 0.3943 | 0.4442 |
